# A Network Pharmacology Approach for Uncovering the Antitumor Effects and Potential Mechanisms of the Sijunzi Decoction for the Treatment of Gastric Cancer

**DOI:** 10.1155/2022/9364313

**Published:** 2022-04-12

**Authors:** Pengpeng Ding, Yutong Guo, Canghai Wang, Jianhong Chen, Chunmei Guo, Hong Liu, Qi Shi

**Affiliations:** ^1^Department of Gastroenterology, Beijing Shijitan Hospital of Capital Medical University, Beijing 100038, China; ^2^Ophthalmoptometry Class, Fourth Clinical Medical College, Nanjing Medical University, Nanjing, Jiangsu 210000, China; ^3^Department of Oncology, Beijing Shijitan Hospital of Capital Medical University, Beijing, China

## Abstract

**Background:**

Sijunzi decoction (SJZD), a classic Chinese formula, has been clinically used for the treatment of gastrointestinal disorders. However, few studies have uncovered its antitumor effects and its potential mechanisms against gastric cancer (GC). Therefore, this work aimed to identify the active compounds and putative targets of the SJZD and to further explore the potential mechanisms involved in the treatment of GC.

**Materials and Methods:**

The active compounds and potential targets of the SJZD and related genes for GC treatment were collected from a public database. Traditional Chinese medicine (TCM)-compound-target-disease networks, Venn diagrams, protein–protein interactions (PPIs), gene ontology (GO), and the Kyoto Encyclopedia of Genes and Genomes (KEGG) were used to obtain the bioactive compounds, key targets, and potential pathways. Next, the human gastric adenocarcinoma cell line NUGC-4 was inoculated subcutaneously into the right flank of NCG mice to build a tumor-bearing mouse model to further verify the findings.

**Results:**

There were 117 compounds in the SJZD in total. The SJZD and GC had 161 and 3288 potential targets, respectively, among which 123 targets overlapped. The network analysis showed that quercetin, kaempferol formononetin, ginsenoside, atractylenolide III, etc., were bioactive molecules. The tumor necrosis factor (TNF), interleukin-6 (IL-6), cellular tumor antigen p53 (TP53), transcription factor AP-1 (JUN), and vascular endothelial growth factor A (VEGFA) were potential targets. A KEGG pathway enrichment analysis revealed 110 pathways involved in the pathways for cancer, including the PI3K-AKT signaling pathway. Validation experiments showed that the SJZD inhibited tumor growth and induced apoptosis in tumor cells. In addition, the SJZD downregulated expressions of VEGFA, iNOS, COX-2, and Bax/Bcl2 and inhibited the expressions of p-PI3K and p-AKT.

**Conclusion:**

The SJZD treats GC by inhibiting blood vessel hyperplasia and inducing cell apoptosis by regulating the PI3K/AKT pathway.

## 1. Introduction

With its heterogenous nature, gastric cancer (GC) is a highly aggressive malignancy, and it is the fourth most common cancer in the world, with more than 70% of the cases occurring in the developing world [1, 2]. Due to increases in the standards of hygiene, in food conservation, and in the intake of fresh fruits, incidences of GC have decreased in most parts of the world [3, 4]. Smoking, drinking, *Helicobacter pylori* infections, etc., can still significantly increase the risk of developing GC [5, 6]. GC patients mainly receive treatments that include surgical resection, chemotherapy, and targeted therapy [7–9]. However, these therapy approaches impose physical, psychological, and financial burdens on GC patients [10]. Traditional Chinese medicine (TCM), which is low-cost and has low toxicity, has been utilized in China for a long time to prevent and treat GC and warrants further investigations [11, 12].

The Sijunzi decoction (SJZD) is a classic Chinese formula for “reinforcing asthenia qi” and nourishing the spleen; it consists of Renshen (Codonopsis pilosula), Baizhu (Atractylodes macrocephala), Fulin (Poria cocos), and Gancao (*Glycyrrhiza* uralensis) [13]. In clinical applications, researchers have recently observed that the SJZD has some therapeutic effects in the treatment of malignant digestive tumors [14]. Zhao Aiguang et al. also demonstrated that SJZD inhibited GC cell growth in vivo [15]. However, due to the multiple pathways by which TCM acts, the mechanism of the SJZD action for the treatment of GC remains to be explored.

**Figure 1:**
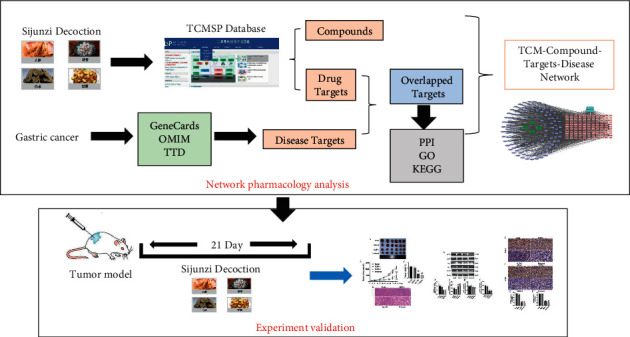
Workflow illustration of the network analysis.

Network pharmacology is an integrated discipline that combines systems biology, multidirectional pharmacology, computational biology, and network analysis to investigate the relationships between drugs and diseases to find drug targets [16]. Chinese herbs usually possess multiple components and targets, which are consistent with the characteristics of network pharmacology [17]. In recent years, substantial advances have been made in exploring the mechanisms of TCM action and searching for the active compounds and therapeutic targets involved by utilizing network pharmacology [18, 19]. Therefore, in this study, we used network pharmacology to identify the active compounds and putative targets of SJZD and further explored the potential mechanisms involved in the treatment of GC. Furthermore, we validated the results of network pharmacology in vivo by building a GC murine model (Figure 1).

## 2. Materials and Methods

### 2.1. Screening of Active Compounds and Potential Targets of the SJZD for the Treatment of GC

The keywords “Renshen” (Renshen is the root of the acanthaceae plant), “Baizhu” (Baizhu is the rhizome of Atractylodes macrocephala Koidz.), “Fulin” (Fulin is the dried sclerotia of Poria cocos (Schw.)Wolf), and “Gancao” (Gancao is the dried root and rhizome of *Glycyrrhiza* uralensis Fisch of legumes) were selected, and the herbs' compounds and targets were retrieved from the Traditional Chinese Medicine System Pharmacology Database (TCMSP, https://tcmspw.com/index.php). The compounds were screened according to the pharmacokinetic parameters “oral bioavailability (OB) ≥ 30%” and “drug likeness (DL) ≥ 0.18”. Then, the targets of GC were obtained from the GeneCards (https://www.genecards.org/), Online Mendelian Inheritance in Man (OMIM, https://omim.org/), and Therapeutic Target Database (TTD, https://db.idrblab.org/ttd/) databases with “Gastric Cancer” as the keyword. Next, the corresponding gene names were found in the UniProt Knowledgebase (UniProtKB) (https://www.UniProt.org/) by searching for “*Homo sapiens*” [20].

### 2.2. TCM-Compound-Target-Disease Network Construction

The intersecting targets of the SJZD and GC were searched to make a Venn diagram. The above targets and corresponding compounds as well as the Chinese herbs were imported into a Cytoscape 3.6.1 software, which is a useful tool for analyzing and visualizing biological networks, to generate a TCM-compound-target-disease network.

### 2.3. Protein–Protein Interaction (PPI) Network

The intersection targets of the SJZD and GC were uploaded to the STRING database (https://cn.string-db.org/cgi/) with the following limiting condition: species (*Homo sapiens*). Then, the PPI network was analyzed by using Cytoscape 3.6.1, which obtained “Nodes” representing the target protein molecules and “Edges” representing the mutual relationships.

### 2.4. GO and KEGG Pathway Enrichment Analysis

A gene ontology (GO) analysis including biological process (BP), cellular component (CC), and molecular function (MF) was performed in the DAVID database (https://david.ncifcrf.gov/home.jsp) by inputting the above targets. Then, the top 10 terms were selected to make graphical structures in GraphPad Prism 8.0. The results of the Kyoto Encyclopedia of Genes and Genomes (KEGG) pathway analysis were obtained from the DAVID database, and a bubble chart was made on imageGP (http://www.ehbio.com) to explore the correlated functional pathways of the selected genes.

### 2.5. Tumor Model and Therapeutic Experiments

The human gastric adenocarcinoma cell line NUGC-4 was maintained in a RPMI-1640 medium supplemented with 10% fetal bovine serum at 37°C in a humidified atmosphere containing 5% CO_2_. The NUGC-4 cell suspension for ultimate collection had a total of 1 × 108 NUGC-4 cells in 1 mL of PBS [21]. The cells were inoculated subcutaneously into the right flanks of NCG (NOD/ShiLtJGpt-Prkdcem26Il2rgem26/Gpt, male, 5 weeks of age) mice (100 *μ*L/mouse). The study was approved by the Medical Research Ethics Review Committee of the Beijing Shijitan Hospital of the Capital Medical University (Beijing, China). When the tumor size reached 100 mm^3^, the tumor-bearing mice were randomly divided into 4 groups: model group (*n* = 6), SJZD-L group (SJZD-low dose, 10 mg/kg, *n* = 6), SJZD-H group (SJZD high dose, 40 mg/kg, *n* = 6), and Urelumab group (20 mg/kg, *n* = 6). Mice in the SJZD groups were administered SZJD once a day, and mice in the Model group were given an equal volume of distilled water. The mice in the Urelumab group were injected with Urelumab twice a week. The tumor size was measured every week. After the administration of the drugs for 21 days, the mice were sacrificed, and the tumors were isolated, photographed, and weighed. The tumor growth inhibition weight value (TGIW) and tumor growth inhibition volume value (TGIV) were statistically analyzed. TGIW% = (1-Treatment group weight/Model group weight) × 100%; TGIV% =  (1-Treatment group RTV/Model group RTV) × 100%; Relative tumor volume (RTV) = V_21 day_/V_0 day_; Tumor volume = 0.5 × major axis × minor axis^2^.

### 2.6. H&E Staining

Tissues were fixed with 10% neutral buffered formalin overnight, embedded in paraffin, and cut into 4.5 *μ*m sections using a microtome. After dewaxing and rehydration, the sections were stained with hematoxylin & eosin solution (R20570, Yuanye, Shanghai, China). Tumors were identified on sections. These sections were sealed using neutral gum and photographed under a microscope (Nikon, Tokyo, Japan) at  × 200.

### 2.7. Western Blot

Tumors were lysed using a radioimmunoprecipitation assay (RIPA) buffer to obtain the proteins. The protein concentrations were detected by a BCA protein assay kit. The proteins were separated by SDS/PAGE and blotted onto polyvinylidene fluoride (PVDF) membranes. Next, the membranes were blocked in skim milk for 2 hours and then incubated with primary antibodies against Bax (1 : 1000, 50599-2-ig, Proteintech, Wuhan, China), Bcl2 (1 : 1000, 12789-1-AP, Proteintech, Wuhan, China), p-PI3K (1 : 1000, 67071-1-Ig, Proteintech, Wuhan, China), p-Akt (1 : 1000, 10716-2-AP, Proteintech, Wuhan, China), and VEGFA (1 : 1000, 19003-1-AP, Proteintech, Wuhan, China) at 4°C overnight, followed by HRP-conjugated secondary antibodies. Protein bands were detected using an ECL chemiluminescence system (Beyotime, Shanghai, China). *β*-actin (1 : 1000, 66009-1-Ig, Proteintech, Wuhan, China) was used as an internal control.

### 2.8. Immunohistochemistry

After dehydration, hydration, and antigen recovery, tumor biopsies were incubated with the primary antibodies at the following dilution ratios: iNOS (1 : 800, 18985-1-AP, Proteintech, Wuhan, China) and COX-2 (1 : 800, 66351-1-Ig, Proteintech, Wuhan, China). Subsequently, the sections were incubated with a secondary antibody for 2 hours at room temperature. Finally, these sections were sealed using neutral gum and photographed under a microscope at a magnification  × 200.

### 2.9. Statistical Analysis

Data are presented as the mean ± standard deviation. Differences between the two groups were investigated using Student's *t*-test. *P* < 0.05 was considered to indicate a statistically significant difference.

## 3. Results

### 3.1. The Active Compounds and Potential Targets of the SJZD for the Treatment of GC

**Figure 2:**
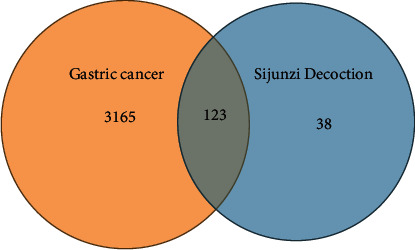
Venn diagram of the targets of gastric cancer and the Sijunzi Decoction. The orange and blue circles represent the GC and SJZD targets, respectively. TCM-compound-target-disease network analysis.

As is shown in the Supplement Table 1, there are 117 compounds in the SJZD in total (5 compounds in Baizhu, 6 compounds in Fulin, 90 compounds in Gancao, and 17 compounds in Renshen; Renshen and Gancao have one common compound). In addition, the SJZD and GC had 161 and 3288 potential targets, respectively. A total of 123 targets overlapped, which were the main related targets in the SJZD for the treatment of GC (Figure 2).

**Figure 3:**
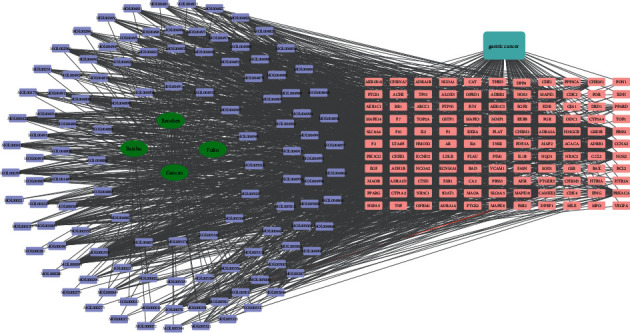
TCM compound-target-disease network. The purple rectangle represents the SJZD compounds. The green oval represents the four herbs in the SJZD. The orange and pink rectangles represent intersecting targets. The blue rectangle of the lake represents GC.

To perform a comprehensive analysis, a TCM-compound-target-disease network was constructed (Supplement Table 2 and Figure 3). The network had 245 nodes, including 1 disease, 4 Chinese herbs, 117 compounds, and 123 targets. Each compound had multiple effective targets, such as quercetin (MOL000098), kaempferol (MOL000422), 7-methoxy-2-methyl isoflavone (MOL003896), formononetin (MOL000392), and isorhamnetin (MOL 000354), with 60, 36, 34, 27, and 25 targets, respectively. On the other hand, each target also interacted with many compounds, such as the estrogen receptor (ESR1), calmodulin (CAMKK2), androgen receptor (AR), glycogen synthase kinase-3 beta (GSK3B), nuclear receptor coactivator 2 (NCOA2), coagulation factor Xa (F10), mitogen-activated protein kinase 14 (MAPK 14), and beta-2 adrenergic receptor (ADRB2), acting on 78, 73, 69, 56, 50, 52, 47, and 38 compounds, respectively. The results demonstrated that the SJZD treats GC with multiple compounds and multiple targets.

### 3.2. PPI Network Analysis

**Figure 4:**
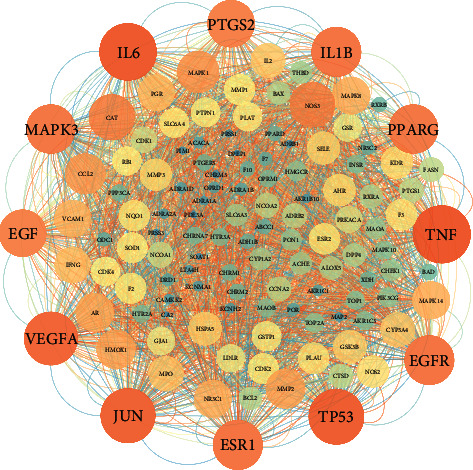
PPI network. The darker the color of the circle is, the larger the shape and the higher the degree value of the target. Gene ontology (GO) functional and KEGG pathway enrichment analyses.

The overlapping targets were input into Cytoscape software to achieve a visualized analysis. The network had 123 nodes and 1275 edges, and the average node degree was 20.7 (Supplement Table 3 and Figure 4). Then, after topology analysis, it was found that the tumor necrosis factor (TNF, degree value = 67), interleukin-6 (IL-6, degree value = 66), cellular tumor antigen p53 (TP53, degree value = 63), transcription factor AP-1 (JUN, degree value = 62), and vascular endothelial growth factor A (VEGFA, degree value = 60) had higher degree values than the other targets. These targets were mainly involved in the inflammatory response, malignant tumors, cell apoptosis, and proliferation.

**Figure 5:**
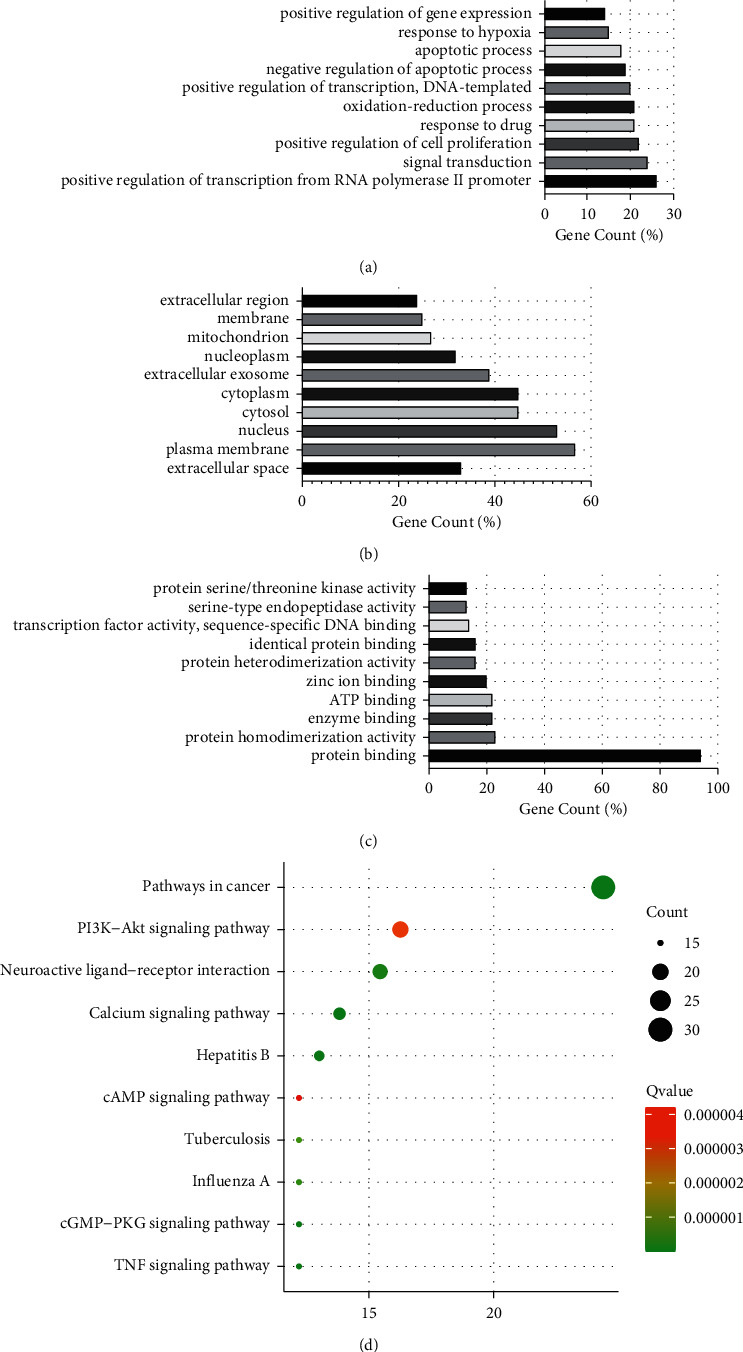
Gene function GO and KEGG pathway enrichment. The top 10 items of BP (a), CC (b), and MF (c) were selected. The *y*-axis shows significantly enriched categories of the targets, and the *x*-axis shows the enrichment scores of these terms (*P* < 0.01). KEGG pathway (d). The darker the circle is, the smaller the Q, and the larger the circle is, the larger the count. The effect of the SJZD on tumor growth.

To further explore the mechanisms of SJZD action for the treatment of GC, the overlapping targets were analyzed for GO functional and KEGG pathway enrichment. The enrichment results showed that there were 440 BP terms involved in the positive regulation of transcription from the RNA polymerase II promoter, signal transduction, and so on; 59 CC terms covered the extracellular space, plasma membrane, etc.; and 100 items of MF including protein binding, protein homodimerization activity, and so on were present. The top enrichment results are displayed in Figures 5(a)–5(c). The related pathways for SJZD action for the treatment of GC were obtained by KEGG pathway enrichment analysis. A total of 110 pathways were enriched (*P* < 0.05), including pathways in cancer and the PI3K-AKT signaling pathway (Figure 5(d)).

**Figure 6:**
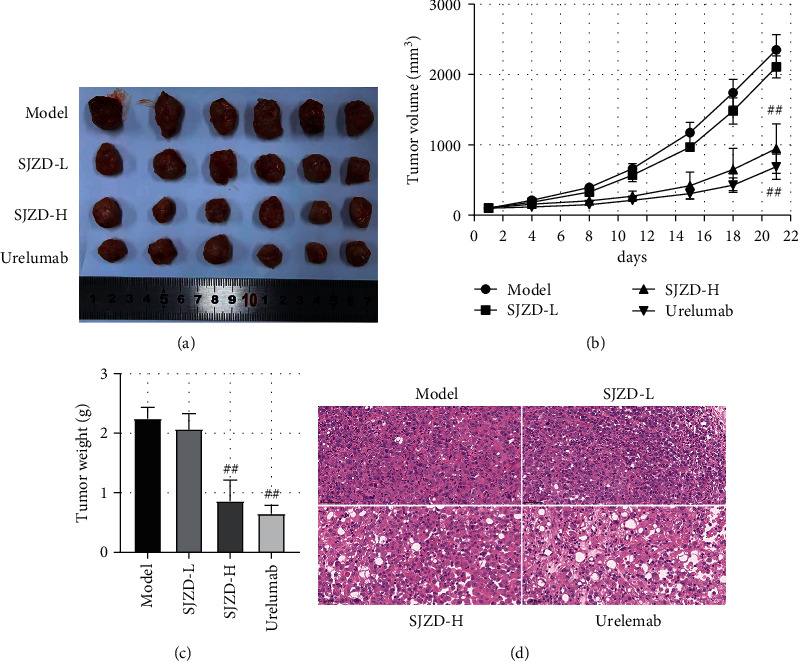
SJZD inhibited the growth of tumors. (a) Images of tumors. (b) Tumor volume. Tumor volume = 0.5 × major axis × minor axis2. (c) Tumor weight. (d) The tumors were stained with H&E (200x). Data are expressed as the means ± SD. Compared with the model group, ^##^*P* < 0.01.

After treatment with the SJZD for 21 days, the treated mice were sacrificed to isolate the tumor. The mice in the SJZD-H group had smaller and lighter tumors than the model group mice (*P* < 0.01, Figures 6(a)–6(c)). In addition, the TGIW and TGIV results indicated that the SJZD-H treatment substantially inhibited tumor growth (Table 1, *P* < 0.01). The H&E results showed that the tumor cells in the model group were closely arranged and grew well, with hyperchromatic nuclei and normal morphologies. After treatment with a high dose of the SJZD, the cells showed necrosis and exhibited diffuse and ambiguous distributions (Figures 6(b)–6(d)). The results suggested that the SJZD had an inhibitory effect on gastric tumor proliferation.

### 3.3. SJZD Regulated the PI3K/AKT Pathway and VEGFA

**Figure 7:**
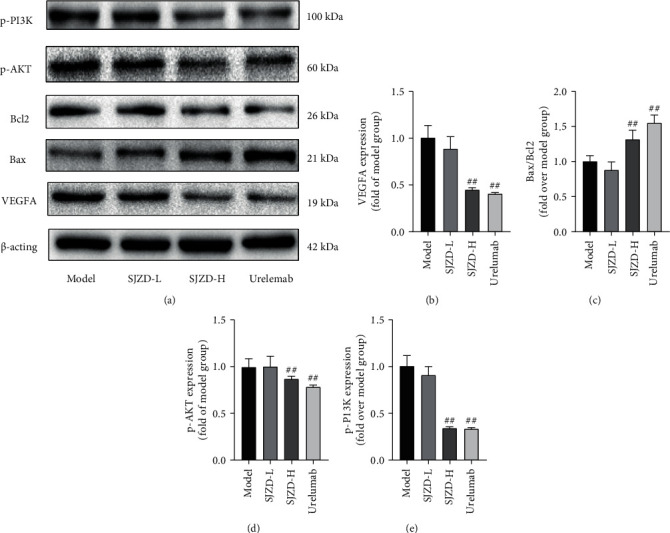
SJZD downregulated VEGFA and the PI3K/AKT pathway. (a) Representative images of WB. Image gel was used to quantify the expression of VEGFA (b) and the ratios of Bax/Bcl2 (c), p-AKT (d), and p-AKT (e). Data are expressed as the means ± SD. Compared with the model group, ^##^*P* < 0.01.

According to the results of the PPI network and KEGG pathway enrichment analyses, specific targets, including VEGFA, Bax, Bcl2, and the PI3K/AKT pathway, were involved in the SJZD treatment of GC. Therefore, we used WB to measure the expression of these proteins. As shown in Figures 7(a)–7(e), compared with the model group, the expression of VEGFA, p-PI3K, and p-AKT in the SJZD-H group was distinctly reduced along with an elevation in the ratio of Bax/Bcl2 (*P* < 0.01). The results suggested that the SJZD regulated the PI3K/AKT/VEGF pathway to treat GC.

### 3.4. Effect of the SJZD on Vascular Proliferation

**Figure 8:**
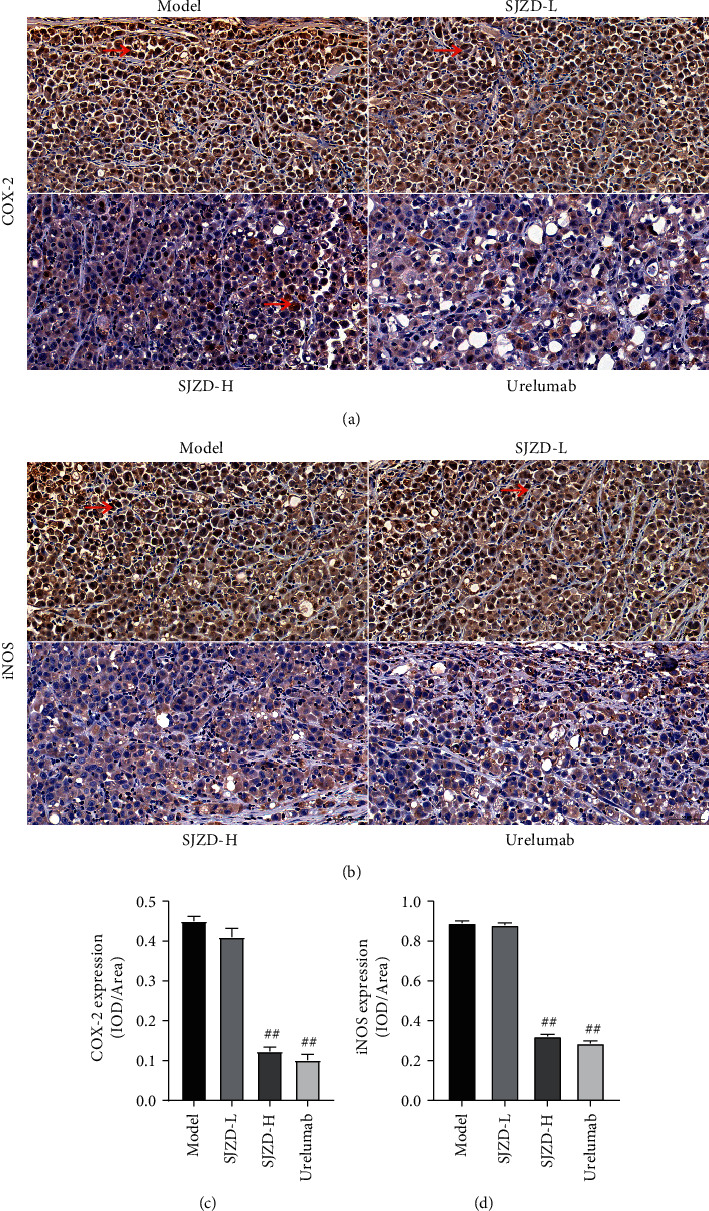
SJZD inhibited COX-2 and iNOX expression. (a)-(b) Representative immunohistochemistry images. Image-Pro was used to quantify the expression of COX-2 (c) and iNOS (d). Data are expressed as the means ± SD. Compared with the model group, ^##^*P* < 0.01.

Furthermore, the expression levels of iNOS and COX-2 were evaluated by immunohistochemistry. Figures 8(a) and 8(b) show that the expression levels of iNOS and COX-2 in the model group were distinctly higher than those in the SJZD-H group and Urelumab group (*P* < 0.01). These results demonstrated that SJZD treatment downregulated the iNOS and COX-2 levels in the gastric tumor models.

## 4. Discussion

In this study, network pharmacology and in vivo experiments were performed to reveal the bioactivity and mechanism of SJZD action for treating GC. The TCM-compound-target-disease network showed connections between multiple compounds and targets, which is consistent with the holism of TCM [22]. PPI results showed that TNF, IL-6, VEGFA, etc., played an important role in the SJZD treatment of GC. The GO functional and KEGG pathway enrichment results revealed the function of targets and potential mechanisms of the SJZD for the treatment of GC involving with pathways in cancer, such as the PI3K-AKT signaling pathway and so on. Furthermore, the experiment verified that the SJZD had a therapeutic effect on the gastric cancer model mice involving the VEGFA/PI3K/AKT pathways.

The SJZD, a traditional Chinese prescription, has been used for the clinical treatment of gastrointestinal disorders for over 2000 years [23]. Due to its high therapeutic value, in recent years, the bioactive compounds in the SJZD have attracted a considerable amount of attention. Zhibo Guan et al. demonstrated that formononetin, ginsenoside, atractylenolide III, and so on were the characteristic components in the SJZD [24]. As shown in the analysis of the TCM compound-target-disease network, formononetin (MOL000392), ginsenoside (MOL005344), and atractylenolide III (MOL000072) were also the major active compounds in the SJZD treatment of GC. Furthermore, it has been reported that formononetin, ginsenoside, and atractylenolide III have antineoplastic efficacy in GC cells [25–27]. Our results also showed that the SJZD had an marked inhibitory effect on tumor cell proliferation, as evidenced by the reduced tumor weight and volume. In addition, pathology results also confirmed the SJZD-induced apoptosis of tumor cells.

The PPI network and KEGG results indicated that VEGFA was the main target in the treatment with SZJD against GC. VEGF is a well-known angiogenic factor and plays an important role in the proliferation, migration, and invasion of vascular endothelial cells [28]. The angiogenic pathway is a sound target for obstructing the excessive proliferation of cells because nutrients and growth factors are supplied through blood vessels to tumor cells [29]. Previous studies have indicated that during the development of GC, COX-2 and increased proangiogenic growth factors, particularly VEGF, have close relationships in which the downregulation of COX-2 inhibits VEGF generation [30, 31]. In addition, it has been reported that COX-2 overexpression in tumors significantly correlates with iNOS overexpression [32]. The nitric oxide (NO) produced through iNOS might increase vascular permeability by increasing the enzymatic activity of COX-2, accelerating the nutrient supply to tumor tissues, and ultimately promoting tumor growth [33]. In our animal experiments, we found that the SJZD suppressed the expression of VEGFA, COX-2, and iNOS to prevent tumor cell proliferation during GC development, which was consistent with the results of Yang et al. [34].

To further explore the mechanisms of SJZD action for the treatment of GC, KEGG enrichment analysis was performed, which showed that the PI3K-AKT signaling pathway was the most enriched gene involved in the SJZD treatment of GC. The PI3K-AKT signaling pathway participates in many cellular activities, including cell growth, differentiation, apoptosis, and cytoskeletal rearrangement [35]. PI3K is an intracellular phosphatidylinositol kinase that can activate AKT, its downstream target, to regulate cell apoptosis during the development of human tumors [36]. LiRong et al. demonstrated that by targeting the PI3K/AKT pathway, the proliferation of GC in vivo and vitro can be inhibited, and cell apoptosis is induced by increasing the Bax/Bcl2 ratio [37]. Our results also emphasized that SZJD inhibited PI3K and AKT expression and increased the Bax/Bcl2 ratio in a GC model.

## 5. Conclusion

In summary, our study combined network pharmacology-based predictions and in vivo experiments to verify the antineoplastic efficacy and the potential mechanisms of SJZD action for the treatment of GC. We found that the SJZD exerted its anti-GC effects by downregulating the expression of VEGFA, iNOS, and COX-2 to inhibit blood vessel hyperplasia and by upregulating the Bax/Bcl2 ratio to induce cell apoptosis by regulating the PI3K/AKT pathway. However, there were still deficiencies in this study. The potential compounds involved in the SJZD treatment of GC need to be identified and validated in vivo or in vitro. In addition, the related targets should be further explored to provide a theory for the SJZD treatment of GC.

## Figures and Tables

**Table 1:** Tumor inhibition rate.

Group	TGIV%	TGIW%
Model	—	—
SJZD-L	9.9	7.8
SJZD-H	60.3^##^	61.6^##^
Urelumab	70.5^##^	70.8^##^

*Note.* TGIW% =  (1-Treatment group weight/Model group weight) × 100%; TGIV =  (1-Treatment group RTV/Model group RTV) × 100%; Relative tumor volume (RTV) = V_21 day_/V_0 day_, Compared with model group, ^##^*P* < 0.01.

## Data Availability

All data generated or analyzed during this study are included in this published article.

## Supplementary Materials

Supplementary Materials Supplement Table 1. The active compounds of SJZD. Supplement Table 2. TCM-TCM-compound-target-disease analysis. Supplemental Table 3. PPI analysis.
