# Supplementary material for: A Network Pharmacology Approach for Uncovering the Antitumor Effects and Potential Mechanisms of the Sijunzi Decoction for the Treatment of Gastric Cancer
Source: Evid Based Complement Alternat Med. 2022 Apr 12;2022:9364313. doi: 10.1155/2022/9364313 (PMC9019414; doi:10.1155/2022/9364313)
Supplement: Supplementary Materials — Supplement Table 1. The active compounds of SJZD. Supplement Table 2. TCM-TCM-compound-target-disease analysis. Supplemental Table 3. PPI analysis. [file 9364313.f1.zip › 9364313.f1/Supplement Table.1 (1).docx]

**Supplement. Table.1** The active compounds of SJZD

| **TCM** | **MOL** | **Compounds** |
| --- | --- | --- |
| Baizhu | MOL000018 | (+/-)-Isoborneol |
| Baizhu | MOL000022 | 14-acetyl-12-senecioyl-2E,8Z,10E-atractylentriol |
| Baizhu | MOL000033 | (3S,8S,9S,10R,13R,14S,17R)-10,13-dimethyl-17-[(2R,5S)-5-propan-2-yloctan-2-yl]-2,3,4,7,8,9,11,12,14,15,16,17-dodecahydro-1H-cyclopenta[a]phenanthren-3-ol |
| Baizhu | MOL000049 | 3β-acetoxyatractylone |
| Baizhu | MOL000072 | 8β-ethoxy atractylenolide Ⅲ |
| Fulin | MOL000273 | (2R)-2-[(3S,5R,10S,13R,14R,16R,17R)-3,16-dihydroxy-4,4,10,13,14-pentamethyl-2,3,5,6,12,15,16,17-octahydro-1H-cyclopenta[a]phenanthren-17-yl]-6-methylhept-5-enoic acid |
| Fulin | MOL000275 | trametenolic acid |
| Fulin | MOL000279 | Cerevisterol |
| Fulin | MOL000282 | ergosta-7,22E-dien-3beta-ol |
| Fulin | MOL000283 | Ergosterol peroxide |
| Fulin | MOL000296 | hederagenin |
| Gancao | MOL000098 | quercetin |
| Gancao | MOL000105 | protocatechuic acid |
| Gancao | MOL000211 | Mairin |
| Gancao | MOL000239 | Jaranol |
| Gancao | MOL000354 | isorhamnetin |
| Gancao | MOL000359 | sitosterol |
| Gancao | MOL000392 | formononetin |
| Gancao | MOL000417 | Calycosin |
| Gancao, Rhenshen | MOL000422 | kaempferol |
| Gancao | MOL000497 | licochalcone a |
| Gancao | MOL000500 | Vestitol |
| Gancao | MOL001484 | Inermine |
| Gancao | MOL001792 | DFV |
| Gancao | MOL002311 | Glycyrol |
| Gancao | MOL002565 | Medicarpin |
| Gancao | MOL002844 | Pinocembrin |
| Gancao | MOL003656 | Lupiwighteone |
| Gancao | MOL003896 | 7-Methoxy-2-methyl isoflavone |
| Gancao | MOL004328 | naringenin |
| Gancao | MOL004805 | (2S)-2-[4-hydroxy-3-(3-methylbut-2-enyl)phenyl]-8,8-dimethyl-2,3-dihydropyrano[2,3-f]chromen-4-one |
| Gancao | MOL004806 | euchrenone |
| Gancao | MOL004808 | glyasperin B |
| Gancao | MOL004810 | glyasperin F |
| Gancao | MOL004811 | Glyasperin C |
| Gancao | MOL004814 | Isotrifoliol |
| Gancao | MOL004815 | (E)-1-(2,4-dihydroxyphenyl)-3-(2,2-dimethylchromen-6-yl)prop-2-en-1-one |
| Gancao | MOL004820 | kanzonols W |
| Gancao | MOL004824 | (2S)-6-(2,4-dihydroxyphenyl)-2-(2-hydroxypropan-2-yl)-4-methoxy-2,3-dihydrofuro[3,2-g]chromen-7-one |
| Gancao | MOL004827 | Semilicoisoflavone B |
| Gancao | MOL004828 | Glepidotin A |
| Gancao | MOL004829 | Glepidotin B |
| Gancao | MOL004833 | Phaseolinisoflavan |
| Gancao | MOL004835 | Glypallichalcone |
| Gancao | MOL004838 | 8-(6-hydroxy-2-benzofuranyl)-2,2-dimethyl-5-chromenol |
| Gancao | MOL004841 | Licochalcone B |
| Gancao | MOL004848 | licochalcone G |
| Gancao | MOL004849 | 3-(2,4-dihydroxyphenyl)-8-(1,1-dimethylprop-2-enyl)-7-hydroxy-5-methoxy-coumarin |
| Gancao | MOL004855 | Licoricone |
| Gancao | MOL004856 | Gancaonin A |
| Gancao | MOL004857 | Gancaonin B |
| Gancao | MOL004863 | 3-(3,4-dihydroxyphenyl)-5,7-dihydroxy-8-(3-methylbut-2-enyl)chromone |
| Gancao | MOL004864 | 5,7-dihydroxy-3-(4-methoxyphenyl)-8-(3-methylbut-2-enyl)chromone |
| Gancao | MOL004866 | 2-(3,4-dihydroxyphenyl)-5,7-dihydroxy-6-(3-methylbut-2-enyl)chromone |
| Gancao | MOL004879 | Glycyrin |
| Gancao | MOL004882 | Licocoumarone |
| Gancao | MOL004883 | Licoisoflavone |
| Gancao | MOL004884 | Licoisoflavone B |
| Gancao | MOL004885 | licoisoflavanone |
| Gancao | MOL004891 | shinpterocarpin |
| Gancao | MOL004898 | (E)-3-[3,4-dihydroxy-5-(3-methylbut-2-enyl)phenyl]-1-(2,4-dihydroxyphenyl)prop-2-en-1-one |
| Gancao | MOL004903 | liquiritin |
| Gancao | MOL004904 | licopyranocoumarin |
| Gancao | MOL004907 | Glyzaglabrin |
| Gancao | MOL004908 | Glabridin |
| Gancao | MOL004910 | Glabranin |
| Gancao | MOL004911 | Glabrene |
| Gancao | MOL004912 | Glabrone |
| Gancao | MOL004913 | 1,3-dihydroxy-9-methoxy-6-benzofurano[3,2-c]chromenone |
| Gancao | MOL004914 | 1,3-dihydroxy-8,9-dimethoxy-6-benzofurano[3,2-c]chromenone |
| Gancao | MOL004915 | Eurycarpin A |
| Gancao | MOL004924 | (-)-Medicocarpin |
| Gancao | MOL004935 | Sigmoidin-B |
| Gancao | MOL004941 | (2R)-7-hydroxy-2-(4-hydroxyphenyl)chroman-4-one |
| Gancao | MOL004945 | (2S)-7-hydroxy-2-(4-hydroxyphenyl)-8-(3-methylbut-2-enyl)chroman-4-one |
| Gancao | MOL004948 | Isoglycyrol |
| Gancao | MOL004949 | Isolicoflavonol |
| Gancao | MOL004957 | HMO |
| Gancao | MOL004959 | 1-Methoxyphaseollidin |
| Gancao | MOL004961 | Quercetin der. |
| Gancao | MOL004966 | 3'-Hydroxy-4'-O-Methylglabridin |
| Gancao | MOL004974 | 3'-Methoxyglabridin |
| Gancao | MOL004978 | 2-[(3R)-8,8-dimethyl-3,4-dihydro-2H-pyrano[6,5-f]chromen-3-yl]-5-methoxyphenol |
| Gancao | MOL004980 | Inflacoumarin A |
| Gancao | MOL004985 | icos-5-enoic acid |
| Gancao | MOL004988 | Kanzonol F |
| Gancao | MOL004989 | 6-prenylated eriodictyol |
| Gancao | MOL004990 | 7,2',4'-trihydroxy－5-methoxy-3－arylcoumarin |
| Gancao | MOL004991 | 7-Acetoxy-2-methylisoflavone |
| Gancao | MOL004993 | 8-prenylated eriodictyol |
| Gancao | MOL004996 | gadelaidic acid |
| Gancao | MOL005000 | Gancaonin G |
| Gancao | MOL005001 | Gancaonin H |
| Gancao | MOL005003 | Licoagrocarpin |
| Gancao | MOL005007 | Glyasperins M |
| Gancao | MOL005008 | Glycyrrhiza flavonol A |
| Gancao | MOL005012 | Licoagroisoflavone |
| Gancao | MOL005016 | Odoratin |
| Gancao | MOL005017 | Phaseol |
| Gancao | MOL005018 | Xambioona |
| Gancao | MOL005020 | dehydroglyasperins C |
| Renshen | MOL000358 | beta-sitosterol |
| Renshen | MOL000449 | Stigmasterol |
| Renshen | MOL000787 | Fumarine |
| Renshen | MOL002879 | Diop |
| Renshen | MOL003648 | Inermin |
| Renshen | MOL005308 | Aposiopolamine |
| Renshen | MOL005317 | Deoxyharringtonine |
| Renshen | MOL005318 | Dianthramine |
| Renshen | MOL005320 | arachidonate |
| Renshen | MOL005321 | Frutinone A |
| Renshen | MOL005344 | ginsenoside rh2 |
| Renshen | MOL005348 | Ginsenoside-Rh4_qt |
| Renshen | MOL005356 | Girinimbin |
| Renshen | MOL005376 | Panaxadiol |
| Renshen | MOL005384 | suchilactone |
| Renshen | MOL005399 | alexandrin_qt |
