# Supplementary material for: A Network Pharmacology Approach for Uncovering the Antitumor Effects and Potential Mechanisms of the Sijunzi Decoction for the Treatment of Gastric Cancer
Source: Evid Based Complement Alternat Med. 2022 Apr 12;2022:9364313. doi: 10.1155/2022/9364313 (PMC9019414; doi:10.1155/2022/9364313)
Supplement: Supplementary Materials — Supplement Table 1. The active compounds of SJZD. Supplement Table 2. TCM-TCM-compound-target-disease analysis. Supplemental Table 3. PPI analysis. [file 9364313.f1.zip › 9364313.f1/Supplement Table2 (1).docx]

**Supplement Table.2 TCM-** **TCM-compound-target-disease analysis**

| **Herbs** | **MOL** | **Compounds** | **Gene** | **Disease** |
| --- | --- | --- | --- | --- |
| Baizhu | MOL000049 | 3β-acetoxyatractylone | ACHE | gastric cancer |
| Baizhu | MOL000018 | (+/-)-Isoborneol | ADRA1A | gastric cancer |
| Baizhu | MOL000049 | 3β-acetoxyatractylone | ADRA1A | gastric cancer |
| Baizhu | MOL000049 | 3β-acetoxyatractylone | ADRB2 | gastric cancer |
| Baizhu | MOL000049 | 3β-acetoxyatractylone | AR | gastric cancer |
| Baizhu | MOL000018 | (+/-)-Isoborneol | CHRM1 | gastric cancer |
| Baizhu | MOL000049 | 3β-acetoxyatractylone | CHRM1 | gastric cancer |
| Baizhu | MOL000018 | (+/-)-Isoborneol | CHRM2 | gastric cancer |
| Baizhu | MOL000049 | 3β-acetoxyatractylone | CHRM2 | gastric cancer |
| Baizhu | MOL000018 | (+/-)-Isoborneol | CHRM3 | gastric cancer |
| Baizhu | MOL000049 | 3β-acetoxyatractylone | CHRM3 | gastric cancer |
| Baizhu | MOL000049 | 3β-acetoxyatractylone | CHRNA7 | gastric cancer |
| Baizhu | MOL000072 | 8β-ethoxy atractylenolide Ⅲ | CHRNA7 | gastric cancer |
| Baizhu | MOL000049 | 3β-acetoxyatractylone | DPP4 | gastric cancer |
| Baizhu | MOL000072 | 8β-ethoxy atractylenolide Ⅲ | NCOA1 | gastric cancer |
| Baizhu | MOL000072 | 8β-ethoxy atractylenolide Ⅲ | NCOA2 | gastric cancer |
| Baizhu | MOL000049 | 3β-acetoxyatractylone | NOS3 | gastric cancer |
| Baizhu | MOL000049 | 3β-acetoxyatractylone | OPRM1 | gastric cancer |
| Baizhu | MOL000018 | (+/-)-Isoborneol | PGR | gastric cancer |
| Baizhu | MOL000033 | (3S,8S,9S,10R,13R,14S,17R)-10,13-dimethyl-17-[(2R,5S)-5-propan-2-yloctan-2-yl]-2,3,4,7,8,9,11,12,14,15,16,17-dodecahydro-1H-cyclopenta[a]phenanthren-3-ol | PGR | gastric cancer |
| Baizhu | MOL000018 | (+/-)-Isoborneol | PTGS2 | gastric cancer |
| Baizhu | MOL000022 | 14-acetyl-12-senecioyl-2E,8Z,10E-atractylentriol | PTGS2 | gastric cancer |
| Baizhu | MOL000049 | 3β-acetoxyatractylone | PTGS2 | gastric cancer |
| Baizhu | MOL000072 | 8β-ethoxy atractylenolide Ⅲ | PTGS2 | gastric cancer |
| Baizhu | MOL000049 | 3β-acetoxyatractylone | RXRA | gastric cancer |
| Fulin | MOL000296 | hederagenin | ADH1B | gastric cancer |
| Fulin | MOL000296 | hederagenin | ADRA1B | gastric cancer |
| Fulin | MOL000296 | hederagenin | CHRM1 | gastric cancer |
| Fulin | MOL000296 | hederagenin | CHRM2 | gastric cancer |
| Fulin | MOL000296 | hederagenin | CHRM3 | gastric cancer |
| Fulin | MOL000273 | (2R)-2-[(3S,5R,10S,13R,14R,16R,17R)-3,16-dihydroxy-4,4,10,13,14-pentamethyl-2,3,5,6,12,15,16,17-octahydro-1H-cyclopenta[a]phenanthren-17-yl]-6-methylhept-5-enoic acid | NCOA2 | gastric cancer |
| Fulin | MOL000296 | hederagenin | NCOA2 | gastric cancer |
| Fulin | MOL000273 | (2R)-2-[(3S,5R,10S,13R,14R,16R,17R)-3,16-dihydroxy-4,4,10,13,14-pentamethyl-2,3,5,6,12,15,16,17-octahydro-1H-cyclopenta[a]phenanthren-17-yl]-6-methylhept-5-enoic acid | NR3C2 | gastric cancer |
| Fulin | MOL000275 | trametenolic acid | NR3C2 | gastric cancer |
| Fulin | MOL000279 | Cerevisterol | NR3C2 | gastric cancer |
| Fulin | MOL000296 | hederagenin | PDE3A | gastric cancer |
| Fulin | MOL000282 | ergosta-7,22E-dien-3beta-ol | PGR | gastric cancer |
| Fulin | MOL000283 | Ergosterol peroxide | PGR | gastric cancer |
| Fulin | MOL000296 | hederagenin | PGR | gastric cancer |
| Fulin | MOL000296 | hederagenin | PTGS1 | gastric cancer |
| Fulin | MOL000296 | hederagenin | PTGS2 | gastric cancer |
| Fulin | MOL000296 | hederagenin | RXRA | gastric cancer |
| Gancao | MOL004328 | naringenin | ABCC1 | gastric cancer |
| Gancao | MOL000098 | quercetin | ACACA | gastric cancer |
| Gancao | MOL000098 | quercetin | ACHE | gastric cancer |
| Gancao | MOL000354 | isorhamnetin | ACHE | gastric cancer |
| Gancao | MOL000392 | formononetin | ACHE | gastric cancer |
| Gancao | MOL000422 | kaempferol | ACHE | gastric cancer |
| Gancao | MOL003896 | 7-Methoxy-2-methyl isoflavone | ACHE | gastric cancer |
| Gancao | MOL004808 | glyasperin B | ACHE | gastric cancer |
| Gancao | MOL004811 | Glyasperin C | ACHE | gastric cancer |
| Gancao | MOL004824 | (2S)-6-(2,4-dihydroxyphenyl)-2-(2-hydroxypropan-2-yl)-4-methoxy-2,3-dihydrofuro[3,2-g]chromen-7-one | ACHE | gastric cancer |
| Gancao | MOL004827 | Semilicoisoflavone B | ACHE | gastric cancer |
| Gancao | MOL004833 | Phaseolinisoflavan | ACHE | gastric cancer |
| Gancao | MOL004856 | Gancaonin A | ACHE | gastric cancer |
| Gancao | MOL004884 | Licoisoflavone B | ACHE | gastric cancer |
| Gancao | MOL004885 | licoisoflavanone | ACHE | gastric cancer |
| Gancao | MOL004904 | licopyranocoumarin | ACHE | gastric cancer |
| Gancao | MOL004908 | Glabridin | ACHE | gastric cancer |
| Gancao | MOL004912 | Glabrone | ACHE | gastric cancer |
| Gancao | MOL004924 | (-)-Medicocarpin | ACHE | gastric cancer |
| Gancao | MOL004974 | 3'-Methoxyglabridin | ACHE | gastric cancer |
| Gancao | MOL004978 | 2-[(3R)-8,8-dimethyl-3,4-dihydro-2H-pyrano[6,5-f]chromen-3-yl]-5-methoxyphenol | ACHE | gastric cancer |
| Gancao | MOL004991 | 7-Acetoxy-2-methylisoflavone | ACHE | gastric cancer |
| Gancao | MOL005003 | Licoagrocarpin | ACHE | gastric cancer |
| Gancao | MOL005007 | Glyasperins M | ACHE | gastric cancer |
| Gancao | MOL005008 | Glycyrrhiza flavonol A | ACHE | gastric cancer |
| Gancao | MOL000392 | formononetin | ADRA1A | gastric cancer |
| Gancao | MOL000500 | Vestitol | ADRA1A | gastric cancer |
| Gancao | MOL002565 | Medicarpin | ADRA1A | gastric cancer |
| Gancao | MOL000422 | kaempferol | ADRA1B | gastric cancer |
| Gancao | MOL000497 | licochalcone a | ADRA1B | gastric cancer |
| Gancao | MOL000500 | Vestitol | ADRA1B | gastric cancer |
| Gancao | MOL001484 | Inermine | ADRA1B | gastric cancer |
| Gancao | MOL002565 | Medicarpin | ADRA1B | gastric cancer |
| Gancao | MOL003896 | 7-Methoxy-2-methyl isoflavone | ADRA1B | gastric cancer |
| Gancao | MOL004815 | (E)-1-(2,4-dihydroxyphenyl)-3-(2,2-dimethylchromen-6-yl)prop-2-en-1-one | ADRA1B | gastric cancer |
| Gancao | MOL004829 | Glepidotin B | ADRA1B | gastric cancer |
| Gancao | MOL004833 | Phaseolinisoflavan | ADRA1B | gastric cancer |
| Gancao | MOL004835 | Glypallichalcone | ADRA1B | gastric cancer |
| Gancao | MOL004857 | Gancaonin B | ADRA1B | gastric cancer |
| Gancao | MOL004891 | shinpterocarpin | ADRA1B | gastric cancer |
| Gancao | MOL004908 | Glabridin | ADRA1B | gastric cancer |
| Gancao | MOL004945 | (2S)-7-hydroxy-2-(4-hydroxyphenyl)-8-(3-methylbut-2-enyl)chroman-4-one | ADRA1B | gastric cancer |
| Gancao | MOL004959 | 1-Methoxyphaseollidin | ADRA1B | gastric cancer |
| Gancao | MOL004966 | 3'-Hydroxy-4'-O-Methylglabridin | ADRA1B | gastric cancer |
| Gancao | MOL004974 | 3'-Methoxyglabridin | ADRA1B | gastric cancer |
| Gancao | MOL004978 | 2-[(3R)-8,8-dimethyl-3,4-dihydro-2H-pyrano[6,5-f]chromen-3-yl]-5-methoxyphenol | ADRA1B | gastric cancer |
| Gancao | MOL004991 | 7-Acetoxy-2-methylisoflavone | ADRA1B | gastric cancer |
| Gancao | MOL005003 | Licoagrocarpin | ADRA1B | gastric cancer |
| Gancao | MOL001484 | Inermine | ADRA1D | gastric cancer |
| Gancao | MOL002565 | Medicarpin | ADRA1D | gastric cancer |
| Gancao | MOL003896 | 7-Methoxy-2-methyl isoflavone | ADRA1D | gastric cancer |
| Gancao | MOL004891 | shinpterocarpin | ADRA1D | gastric cancer |
| Gancao | MOL004959 | 1-Methoxyphaseollidin | ADRA1D | gastric cancer |
| Gancao | MOL004991 | 7-Acetoxy-2-methylisoflavone | ADRA1D | gastric cancer |
| Gancao | MOL003896 | 7-Methoxy-2-methyl isoflavone | ADRB1 | gastric cancer |
| Gancao | MOL000098 | quercetin | ADRB2 | gastric cancer |
| Gancao | MOL000392 | formononetin | ADRB2 | gastric cancer |
| Gancao | MOL000417 | Calycosin | ADRB2 | gastric cancer |
| Gancao | MOL000497 | licochalcone a | ADRB2 | gastric cancer |
| Gancao | MOL000500 | Vestitol | ADRB2 | gastric cancer |
| Gancao | MOL001484 | Inermine | ADRB2 | gastric cancer |
| Gancao | MOL001792 | DFV | ADRB2 | gastric cancer |
| Gancao | MOL002565 | Medicarpin | ADRB2 | gastric cancer |
| Gancao | MOL002844 | Pinocembrin | ADRB2 | gastric cancer |
| Gancao | MOL003896 | 7-Methoxy-2-methyl isoflavone | ADRB2 | gastric cancer |
| Gancao | MOL004833 | Phaseolinisoflavan | ADRB2 | gastric cancer |
| Gancao | MOL004835 | Glypallichalcone | ADRB2 | gastric cancer |
| Gancao | MOL004841 | Licochalcone B | ADRB2 | gastric cancer |
| Gancao | MOL004857 | Gancaonin B | ADRB2 | gastric cancer |
| Gancao | MOL004891 | shinpterocarpin | ADRB2 | gastric cancer |
| Gancao | MOL004908 | Glabridin | ADRB2 | gastric cancer |
| Gancao | MOL004911 | Glabrene | ADRB2 | gastric cancer |
| Gancao | MOL004941 | (2R)-7-hydroxy-2-(4-hydroxyphenyl)chroman-4-one | ADRB2 | gastric cancer |
| Gancao | MOL004945 | (2S)-7-hydroxy-2-(4-hydroxyphenyl)-8-(3-methylbut-2-enyl)chroman-4-one | ADRB2 | gastric cancer |
| Gancao | MOL004957 | HMO | ADRB2 | gastric cancer |
| Gancao | MOL004959 | 1-Methoxyphaseollidin | ADRB2 | gastric cancer |
| Gancao | MOL004966 | 3'-Hydroxy-4'-O-Methylglabridin | ADRB2 | gastric cancer |
| Gancao | MOL004974 | 3'-Methoxyglabridin | ADRB2 | gastric cancer |
| Gancao | MOL004978 | 2-[(3R)-8,8-dimethyl-3,4-dihydro-2H-pyrano[6,5-f]chromen-3-yl]-5-methoxyphenol | ADRB2 | gastric cancer |
| Gancao | MOL004980 | Inflacoumarin A | ADRB2 | gastric cancer |
| Gancao | MOL004991 | 7-Acetoxy-2-methylisoflavone | ADRB2 | gastric cancer |
| Gancao | MOL005003 | Licoagrocarpin | ADRB2 | gastric cancer |
| Gancao | MOL005020 | dehydroglyasperins C | ADRB2 | gastric cancer |
| Gancao | MOL000098 | quercetin | AHR | gastric cancer |
| Gancao | MOL000422 | kaempferol | AHR | gastric cancer |
| Gancao | MOL000098 | quercetin | AKR1B10 | gastric cancer |
| Gancao | MOL000354 | isorhamnetin | AKR1B10 | gastric cancer |
| Gancao | MOL004328 | naringenin | AKR1C1 | gastric cancer |
| Gancao | MOL000422 | kaempferol | AKR1C3 | gastric cancer |
| Gancao | MOL000098 | quercetin | ALOX5 | gastric cancer |
| Gancao | MOL000105 | protocatechuic acid | ALOX5 | gastric cancer |
| Gancao | MOL000422 | kaempferol | ALOX5 | gastric cancer |
| Gancao | MOL000098 | quercetin | AR | gastric cancer |
| Gancao | MOL000239 | Jaranol | AR | gastric cancer |
| Gancao | MOL000354 | isorhamnetin | AR | gastric cancer |
| Gancao | MOL000392 | formononetin | AR | gastric cancer |
| Gancao | MOL000417 | Calycosin | AR | gastric cancer |
| Gancao | MOL000422 | kaempferol | AR | gastric cancer |
| Gancao | MOL000497 | licochalcone a | AR | gastric cancer |
| Gancao | MOL000500 | Vestitol | AR | gastric cancer |
| Gancao | MOL003656 | Lupiwighteone | AR | gastric cancer |
| Gancao | MOL003896 | 7-Methoxy-2-methyl isoflavone | AR | gastric cancer |
| Gancao | MOL004805 | (2S)-2-[4-hydroxy-3-(3-methylbut-2-enyl)phenyl]-8,8-dimethyl-2,3-dihydropyrano[2,3-f]chromen-4-one | AR | gastric cancer |
| Gancao | MOL004808 | glyasperin B | AR | gastric cancer |
| Gancao | MOL004810 | glyasperin F | AR | gastric cancer |
| Gancao | MOL004811 | Glyasperin C | AR | gastric cancer |
| Gancao | MOL004814 | Isotrifoliol | AR | gastric cancer |
| Gancao | MOL004815 | (E)-1-(2,4-dihydroxyphenyl)-3-(2,2-dimethylchromen-6-yl)prop-2-en-1-one | AR | gastric cancer |
| Gancao | MOL004820 | kanzonols W | AR | gastric cancer |
| Gancao | MOL004824 | (2S)-6-(2,4-dihydroxyphenyl)-2-(2-hydroxypropan-2-yl)-4-methoxy-2,3-dihydrofuro[3,2-g]chromen-7-one | AR | gastric cancer |
| Gancao | MOL004827 | Semilicoisoflavone B | AR | gastric cancer |
| Gancao | MOL004828 | Glepidotin A | AR | gastric cancer |
| Gancao | MOL004833 | Phaseolinisoflavan | AR | gastric cancer |
| Gancao | MOL004835 | Glypallichalcone | AR | gastric cancer |
| Gancao | MOL004841 | Licochalcone B | AR | gastric cancer |
| Gancao | MOL004848 | licochalcone G | AR | gastric cancer |
| Gancao | MOL004849 | 3-(2,4-dihydroxyphenyl)-8-(1,1-dimethylprop-2-enyl)-7-hydroxy-5-methoxy-coumarin | AR | gastric cancer |
| Gancao | MOL004855 | Licoricone | AR | gastric cancer |
| Gancao | MOL004856 | Gancaonin A | AR | gastric cancer |
| Gancao | MOL004857 | Gancaonin B | AR | gastric cancer |
| Gancao | MOL004863 | 3-(3,4-dihydroxyphenyl)-5,7-dihydroxy-8-(3-methylbut-2-enyl)chromone | AR | gastric cancer |
| Gancao | MOL004866 | 2-(3,4-dihydroxyphenyl)-5,7-dihydroxy-6-(3-methylbut-2-enyl)chromone | AR | gastric cancer |
| Gancao | MOL004879 | Glycyrin | AR | gastric cancer |
| Gancao | MOL004882 | Licocoumarone | AR | gastric cancer |
| Gancao | MOL004883 | Licoisoflavone | AR | gastric cancer |
| Gancao | MOL004884 | Licoisoflavone B | AR | gastric cancer |
| Gancao | MOL004885 | licoisoflavanone | AR | gastric cancer |
| Gancao | MOL004891 | shinpterocarpin | AR | gastric cancer |
| Gancao | MOL004898 | (E)-3-[3,4-dihydroxy-5-(3-methylbut-2-enyl)phenyl]-1-(2,4-dihydroxyphenyl)prop-2-en-1-one | AR | gastric cancer |
| Gancao | MOL004904 | licopyranocoumarin | AR | gastric cancer |
| Gancao | MOL004907 | Glyzaglabrin | AR | gastric cancer |
| Gancao | MOL004908 | Glabridin | AR | gastric cancer |
| Gancao | MOL004911 | Glabrene | AR | gastric cancer |
| Gancao | MOL004912 | Glabrone | AR | gastric cancer |
| Gancao | MOL004914 | 1,3-dihydroxy-8,9-dimethoxy-6-benzofurano[3,2-c]chromenone | AR | gastric cancer |
| Gancao | MOL004915 | Eurycarpin A | AR | gastric cancer |
| Gancao | MOL004948 | Isoglycyrol | AR | gastric cancer |
| Gancao | MOL004949 | Isolicoflavonol | AR | gastric cancer |
| Gancao | MOL004957 | HMO | AR | gastric cancer |
| Gancao | MOL004959 | 1-Methoxyphaseollidin | AR | gastric cancer |
| Gancao | MOL004961 | Quercetin der. | AR | gastric cancer |
| Gancao | MOL004966 | 3'-Hydroxy-4'-O-Methylglabridin | AR | gastric cancer |
| Gancao | MOL004974 | 3'-Methoxyglabridin | AR | gastric cancer |
| Gancao | MOL004978 | 2-[(3R)-8,8-dimethyl-3,4-dihydro-2H-pyrano[6,5-f]chromen-3-yl]-5-methoxyphenol | AR | gastric cancer |
| Gancao | MOL004980 | Inflacoumarin A | AR | gastric cancer |
| Gancao | MOL004988 | Kanzonol F | AR | gastric cancer |
| Gancao | MOL004990 | 7,2',4'-trihydroxy－5-methoxy-3－arylcoumarin | AR | gastric cancer |
| Gancao | MOL004991 | 7-Acetoxy-2-methylisoflavone | AR | gastric cancer |
| Gancao | MOL005000 | Gancaonin G | AR | gastric cancer |
| Gancao | MOL005001 | Gancaonin H | AR | gastric cancer |
| Gancao | MOL005003 | Licoagrocarpin | AR | gastric cancer |
| Gancao | MOL005007 | Glyasperins M | AR | gastric cancer |
| Gancao | MOL005008 | Glycyrrhiza flavonol A | AR | gastric cancer |
| Gancao | MOL005012 | Licoagroisoflavone | AR | gastric cancer |
| Gancao | MOL005016 | Odoratin | AR | gastric cancer |
| Gancao | MOL005017 | Phaseol | AR | gastric cancer |
| Gancao | MOL005020 | dehydroglyasperins C | AR | gastric cancer |
| Gancao | MOL004328 | naringenin | BAD | gastric cancer |
| Gancao | MOL000098 | quercetin | BCL2 | gastric cancer |
| Gancao | MOL000422 | kaempferol | BCL2 | gastric cancer |
| Gancao | MOL000497 | licochalcone a | BCL2 | gastric cancer |
| Gancao | MOL004328 | naringenin | BCL2 | gastric cancer |
| Gancao | MOL000497 | licochalcone a | CA2 | gastric cancer |
| Gancao | MOL004815 | (E)-1-(2,4-dihydroxyphenyl)-3-(2,2-dimethylchromen-6-yl)prop-2-en-1-one | CA2 | gastric cancer |
| Gancao | MOL004835 | Glypallichalcone | CA2 | gastric cancer |
| Gancao | MOL004841 | Licochalcone B | CA2 | gastric cancer |
| Gancao | MOL000239 | Jaranol | CAMKK2 | gastric cancer |
| Gancao | MOL000354 | isorhamnetin | CAMKK2 | gastric cancer |
| Gancao | MOL000392 | formononetin | CAMKK2 | gastric cancer |
| Gancao | MOL000417 | Calycosin | CAMKK2 | gastric cancer |
| Gancao | MOL000422 | kaempferol | CAMKK2 | gastric cancer |
| Gancao | MOL000497 | licochalcone a | CAMKK2 | gastric cancer |
| Gancao | MOL000500 | Vestitol | CAMKK2 | gastric cancer |
| Gancao | MOL001484 | Inermine | CAMKK2 | gastric cancer |
| Gancao | MOL002565 | Medicarpin | CAMKK2 | gastric cancer |
| Gancao | MOL003656 | Lupiwighteone | CAMKK2 | gastric cancer |
| Gancao | MOL003896 | 7-Methoxy-2-methyl isoflavone | CAMKK2 | gastric cancer |
| Gancao | MOL004805 | (2S)-2-[4-hydroxy-3-(3-methylbut-2-enyl)phenyl]-8,8-dimethyl-2,3-dihydropyrano[2,3-f]chromen-4-one | CAMKK2 | gastric cancer |
| Gancao | MOL004806 | euchrenone | CAMKK2 | gastric cancer |
| Gancao | MOL004808 | glyasperin B | CAMKK2 | gastric cancer |
| Gancao | MOL004810 | glyasperin F | CAMKK2 | gastric cancer |
| Gancao | MOL004811 | Glyasperin C | CAMKK2 | gastric cancer |
| Gancao | MOL004815 | (E)-1-(2,4-dihydroxyphenyl)-3-(2,2-dimethylchromen-6-yl)prop-2-en-1-one | CAMKK2 | gastric cancer |
| Gancao | MOL004820 | kanzonols W | CAMKK2 | gastric cancer |
| Gancao | MOL004824 | (2S)-6-(2,4-dihydroxyphenyl)-2-(2-hydroxypropan-2-yl)-4-methoxy-2,3-dihydrofuro[3,2-g]chromen-7-one | CAMKK2 | gastric cancer |
| Gancao | MOL004827 | Semilicoisoflavone B | CAMKK2 | gastric cancer |
| Gancao | MOL004828 | Glepidotin A | CAMKK2 | gastric cancer |
| Gancao | MOL004829 | Glepidotin B | CAMKK2 | gastric cancer |
| Gancao | MOL004833 | Phaseolinisoflavan | CAMKK2 | gastric cancer |
| Gancao | MOL004835 | Glypallichalcone | CAMKK2 | gastric cancer |
| Gancao | MOL004841 | Licochalcone B | CAMKK2 | gastric cancer |
| Gancao | MOL004848 | licochalcone G | CAMKK2 | gastric cancer |
| Gancao | MOL004849 | 3-(2,4-dihydroxyphenyl)-8-(1,1-dimethylprop-2-enyl)-7-hydroxy-5-methoxy-coumarin | CAMKK2 | gastric cancer |
| Gancao | MOL004855 | Licoricone | CAMKK2 | gastric cancer |
| Gancao | MOL004856 | Gancaonin A | CAMKK2 | gastric cancer |
| Gancao | MOL004857 | Gancaonin B | CAMKK2 | gastric cancer |
| Gancao | MOL004863 | 3-(3,4-dihydroxyphenyl)-5,7-dihydroxy-8-(3-methylbut-2-enyl)chromone | CAMKK2 | gastric cancer |
| Gancao | MOL004866 | 2-(3,4-dihydroxyphenyl)-5,7-dihydroxy-6-(3-methylbut-2-enyl)chromone | CAMKK2 | gastric cancer |
| Gancao | MOL004879 | Glycyrin | CAMKK2 | gastric cancer |
| Gancao | MOL004883 | Licoisoflavone | CAMKK2 | gastric cancer |
| Gancao | MOL004884 | Licoisoflavone B | CAMKK2 | gastric cancer |
| Gancao | MOL004885 | licoisoflavanone | CAMKK2 | gastric cancer |
| Gancao | MOL004891 | shinpterocarpin | CAMKK2 | gastric cancer |
| Gancao | MOL004898 | (E)-3-[3,4-dihydroxy-5-(3-methylbut-2-enyl)phenyl]-1-(2,4-dihydroxyphenyl)prop-2-en-1-one | CAMKK2 | gastric cancer |
| Gancao | MOL004903 | liquiritin | CAMKK2 | gastric cancer |
| Gancao | MOL004904 | licopyranocoumarin | CAMKK2 | gastric cancer |
| Gancao | MOL004908 | Glabridin | CAMKK2 | gastric cancer |
| Gancao | MOL004910 | Glabranin | CAMKK2 | gastric cancer |
| Gancao | MOL004911 | Glabrene | CAMKK2 | gastric cancer |
| Gancao | MOL004912 | Glabrone | CAMKK2 | gastric cancer |
| Gancao | MOL004915 | Eurycarpin A | CAMKK2 | gastric cancer |
| Gancao | MOL004935 | Sigmoidin-B | CAMKK2 | gastric cancer |
| Gancao | MOL004941 | (2R)-7-hydroxy-2-(4-hydroxyphenyl)chroman-4-one | CAMKK2 | gastric cancer |
| Gancao | MOL004945 | (2S)-7-hydroxy-2-(4-hydroxyphenyl)-8-(3-methylbut-2-enyl)chroman-4-one | CAMKK2 | gastric cancer |
| Gancao | MOL004949 | Isolicoflavonol | CAMKK2 | gastric cancer |
| Gancao | MOL004957 | HMO | CAMKK2 | gastric cancer |
| Gancao | MOL004959 | 1-Methoxyphaseollidin | CAMKK2 | gastric cancer |
| Gancao | MOL004961 | Quercetin der. | CAMKK2 | gastric cancer |
| Gancao | MOL004966 | 3'-Hydroxy-4'-O-Methylglabridin | CAMKK2 | gastric cancer |
| Gancao | MOL004974 | 3'-Methoxyglabridin | CAMKK2 | gastric cancer |
| Gancao | MOL004978 | 2-[(3R)-8,8-dimethyl-3,4-dihydro-2H-pyrano[6,5-f]chromen-3-yl]-5-methoxyphenol | CAMKK2 | gastric cancer |
| Gancao | MOL004980 | Inflacoumarin A | CAMKK2 | gastric cancer |
| Gancao | MOL004988 | Kanzonol F | CAMKK2 | gastric cancer |
| Gancao | MOL004989 | 6-prenylated eriodictyol | CAMKK2 | gastric cancer |
| Gancao | MOL004991 | 7-Acetoxy-2-methylisoflavone | CAMKK2 | gastric cancer |
| Gancao | MOL004993 | 8-prenylated eriodictyol | CAMKK2 | gastric cancer |
| Gancao | MOL005000 | Gancaonin G | CAMKK2 | gastric cancer |
| Gancao | MOL005001 | Gancaonin H | CAMKK2 | gastric cancer |
| Gancao | MOL005003 | Licoagrocarpin | CAMKK2 | gastric cancer |
| Gancao | MOL005007 | Glyasperins M | CAMKK2 | gastric cancer |
| Gancao | MOL005008 | Glycyrrhiza flavonol A | CAMKK2 | gastric cancer |
| Gancao | MOL005012 | Licoagroisoflavone | CAMKK2 | gastric cancer |
| Gancao | MOL005016 | Odoratin | CAMKK2 | gastric cancer |
| Gancao | MOL005018 | Xambioona | CAMKK2 | gastric cancer |
| Gancao | MOL005020 | dehydroglyasperins C | CAMKK2 | gastric cancer |
| Gancao | MOL004328 | naringenin | CAT | gastric cancer |
| Gancao | MOL000098 | quercetin | CCL2 | gastric cancer |
| Gancao | MOL000354 | isorhamnetin | CCNA2 | gastric cancer |
| Gancao | MOL000392 | formononetin | CCNA2 | gastric cancer |
| Gancao | MOL000417 | Calycosin | CCNA2 | gastric cancer |
| Gancao | MOL000497 | licochalcone a | CCNA2 | gastric cancer |
| Gancao | MOL000500 | Vestitol | CCNA2 | gastric cancer |
| Gancao | MOL002311 | Glycyrol | CCNA2 | gastric cancer |
| Gancao | MOL002565 | Medicarpin | CCNA2 | gastric cancer |
| Gancao | MOL003656 | Lupiwighteone | CCNA2 | gastric cancer |
| Gancao | MOL003896 | 7-Methoxy-2-methyl isoflavone | CCNA2 | gastric cancer |
| Gancao | MOL004808 | glyasperin B | CCNA2 | gastric cancer |
| Gancao | MOL004810 | glyasperin F | CCNA2 | gastric cancer |
| Gancao | MOL004811 | Glyasperin C | CCNA2 | gastric cancer |
| Gancao | MOL004814 | Isotrifoliol | CCNA2 | gastric cancer |
| Gancao | MOL004815 | (E)-1-(2,4-dihydroxyphenyl)-3-(2,2-dimethylchromen-6-yl)prop-2-en-1-one | CCNA2 | gastric cancer |
| Gancao | MOL004820 | kanzonols W | CCNA2 | gastric cancer |
| Gancao | MOL004824 | (2S)-6-(2,4-dihydroxyphenyl)-2-(2-hydroxypropan-2-yl)-4-methoxy-2,3-dihydrofuro[3,2-g]chromen-7-one | CCNA2 | gastric cancer |
| Gancao | MOL004828 | Glepidotin A | CCNA2 | gastric cancer |
| Gancao | MOL004833 | Phaseolinisoflavan | CCNA2 | gastric cancer |
| Gancao | MOL004835 | Glypallichalcone | CCNA2 | gastric cancer |
| Gancao | MOL004841 | Licochalcone B | CCNA2 | gastric cancer |
| Gancao | MOL004848 | licochalcone G | CCNA2 | gastric cancer |
| Gancao | MOL004856 | Gancaonin A | CCNA2 | gastric cancer |
| Gancao | MOL004857 | Gancaonin B | CCNA2 | gastric cancer |
| Gancao | MOL004863 | 3-(3,4-dihydroxyphenyl)-5,7-dihydroxy-8-(3-methylbut-2-enyl)chromone | CCNA2 | gastric cancer |
| Gancao | MOL004866 | 2-(3,4-dihydroxyphenyl)-5,7-dihydroxy-6-(3-methylbut-2-enyl)chromone | CCNA2 | gastric cancer |
| Gancao | MOL004882 | Licocoumarone | CCNA2 | gastric cancer |
| Gancao | MOL004883 | Licoisoflavone | CCNA2 | gastric cancer |
| Gancao | MOL004884 | Licoisoflavone B | CCNA2 | gastric cancer |
| Gancao | MOL004885 | licoisoflavanone | CCNA2 | gastric cancer |
| Gancao | MOL004891 | shinpterocarpin | CCNA2 | gastric cancer |
| Gancao | MOL004898 | (E)-3-[3,4-dihydroxy-5-(3-methylbut-2-enyl)phenyl]-1-(2,4-dihydroxyphenyl)prop-2-en-1-one | CCNA2 | gastric cancer |
| Gancao | MOL004904 | licopyranocoumarin | CCNA2 | gastric cancer |
| Gancao | MOL004907 | Glyzaglabrin | CCNA2 | gastric cancer |
| Gancao | MOL004908 | Glabridin | CCNA2 | gastric cancer |
| Gancao | MOL004912 | Glabrone | CCNA2 | gastric cancer |
| Gancao | MOL004913 | 1,3-dihydroxy-9-methoxy-6-benzofurano[3,2-c]chromenone | CCNA2 | gastric cancer |
| Gancao | MOL004915 | Eurycarpin A | CCNA2 | gastric cancer |
| Gancao | MOL004949 | Isolicoflavonol | CCNA2 | gastric cancer |
| Gancao | MOL004957 | HMO | CCNA2 | gastric cancer |
| Gancao | MOL004959 | 1-Methoxyphaseollidin | CCNA2 | gastric cancer |
| Gancao | MOL004966 | 3'-Hydroxy-4'-O-Methylglabridin | CCNA2 | gastric cancer |
| Gancao | MOL004974 | 3'-Methoxyglabridin | CCNA2 | gastric cancer |
| Gancao | MOL004978 | 2-[(3R)-8,8-dimethyl-3,4-dihydro-2H-pyrano[6,5-f]chromen-3-yl]-5-methoxyphenol | CCNA2 | gastric cancer |
| Gancao | MOL005000 | Gancaonin G | CCNA2 | gastric cancer |
| Gancao | MOL005001 | Gancaonin H | CCNA2 | gastric cancer |
| Gancao | MOL005003 | Licoagrocarpin | CCNA2 | gastric cancer |
| Gancao | MOL005007 | Glyasperins M | CCNA2 | gastric cancer |
| Gancao | MOL005008 | Glycyrrhiza flavonol A | CCNA2 | gastric cancer |
| Gancao | MOL005012 | Licoagroisoflavone | CCNA2 | gastric cancer |
| Gancao | MOL005016 | Odoratin | CCNA2 | gastric cancer |
| Gancao | MOL005017 | Phaseol | CCNA2 | gastric cancer |
| Gancao | MOL005020 | dehydroglyasperins C | CCNA2 | gastric cancer |
| Gancao | MOL000098 | quercetin | CDK1 | gastric cancer |
| Gancao | MOL000422 | kaempferol | CDK1 | gastric cancer |
| Gancao | MOL004808 | glyasperin B | CDK2 | gastric cancer |
| Gancao | MOL004810 | glyasperin F | CDK2 | gastric cancer |
| Gancao | MOL004811 | Glyasperin C | CDK2 | gastric cancer |
| Gancao | MOL004814 | Isotrifoliol | CDK2 | gastric cancer |
| Gancao | MOL004815 | (E)-1-(2,4-dihydroxyphenyl)-3-(2,2-dimethylchromen-6-yl)prop-2-en-1-one | CDK2 | gastric cancer |
| Gancao | MOL004820 | kanzonols W | CDK2 | gastric cancer |
| Gancao | MOL004824 | (2S)-6-(2,4-dihydroxyphenyl)-2-(2-hydroxypropan-2-yl)-4-methoxy-2,3-dihydrofuro[3,2-g]chromen-7-one | CDK2 | gastric cancer |
| Gancao | MOL004827 | Semilicoisoflavone B | CDK2 | gastric cancer |
| Gancao | MOL004828 | Glepidotin A | CDK2 | gastric cancer |
| Gancao | MOL004833 | Phaseolinisoflavan | CDK2 | gastric cancer |
| Gancao | MOL004835 | Glypallichalcone | CDK2 | gastric cancer |
| Gancao | MOL004841 | Licochalcone B | CDK2 | gastric cancer |
| Gancao | MOL004848 | licochalcone G | CDK2 | gastric cancer |
| Gancao | MOL004849 | 3-(2,4-dihydroxyphenyl)-8-(1,1-dimethylprop-2-enyl)-7-hydroxy-5-methoxy-coumarin | CDK2 | gastric cancer |
| Gancao | MOL004863 | 3-(3,4-dihydroxyphenyl)-5,7-dihydroxy-8-(3-methylbut-2-enyl)chromone | CDK2 | gastric cancer |
| Gancao | MOL004866 | 2-(3,4-dihydroxyphenyl)-5,7-dihydroxy-6-(3-methylbut-2-enyl)chromone | CDK2 | gastric cancer |
| Gancao | MOL004882 | Licocoumarone | CDK2 | gastric cancer |
| Gancao | MOL004883 | Licoisoflavone | CDK2 | gastric cancer |
| Gancao | MOL004884 | Licoisoflavone B | CDK2 | gastric cancer |
| Gancao | MOL004885 | licoisoflavanone | CDK2 | gastric cancer |
| Gancao | MOL004891 | shinpterocarpin | CDK2 | gastric cancer |
| Gancao | MOL004898 | (E)-3-[3,4-dihydroxy-5-(3-methylbut-2-enyl)phenyl]-1-(2,4-dihydroxyphenyl)prop-2-en-1-one | CDK2 | gastric cancer |
| Gancao | MOL004904 | licopyranocoumarin | CDK2 | gastric cancer |
| Gancao | MOL004907 | Glyzaglabrin | CDK2 | gastric cancer |
| Gancao | MOL004908 | Glabridin | CDK2 | gastric cancer |
| Gancao | MOL004911 | Glabrene | CDK2 | gastric cancer |
| Gancao | MOL004912 | Glabrone | CDK2 | gastric cancer |
| Gancao | MOL004913 | 1,3-dihydroxy-9-methoxy-6-benzofurano[3,2-c]chromenone | CDK2 | gastric cancer |
| Gancao | MOL004914 | 1,3-dihydroxy-8,9-dimethoxy-6-benzofurano[3,2-c]chromenone | CDK2 | gastric cancer |
| Gancao | MOL004915 | Eurycarpin A | CDK2 | gastric cancer |
| Gancao | MOL004949 | Isolicoflavonol | CDK2 | gastric cancer |
| Gancao | MOL004957 | HMO | CDK2 | gastric cancer |
| Gancao | MOL004959 | 1-Methoxyphaseollidin | CDK2 | gastric cancer |
| Gancao | MOL004961 | Quercetin der. | CDK2 | gastric cancer |
| Gancao | MOL004966 | 3'-Hydroxy-4'-O-Methylglabridin | CDK2 | gastric cancer |
| Gancao | MOL004974 | 3'-Methoxyglabridin | CDK2 | gastric cancer |
| Gancao | MOL004978 | 2-[(3R)-8,8-dimethyl-3,4-dihydro-2H-pyrano[6,5-f]chromen-3-yl]-5-methoxyphenol | CDK2 | gastric cancer |
| Gancao | MOL004990 | 7,2',4'-trihydroxy－5-methoxy-3－arylcoumarin | CDK2 | gastric cancer |
| Gancao | MOL004991 | 7-Acetoxy-2-methylisoflavone | CDK2 | gastric cancer |
| Gancao | MOL005003 | Licoagrocarpin | CDK2 | gastric cancer |
| Gancao | MOL005007 | Glyasperins M | CDK2 | gastric cancer |
| Gancao | MOL005008 | Glycyrrhiza flavonol A | CDK2 | gastric cancer |
| Gancao | MOL005012 | Licoagroisoflavone | CDK2 | gastric cancer |
| Gancao | MOL005016 | Odoratin | CDK2 | gastric cancer |
| Gancao | MOL005017 | Phaseol | CDK2 | gastric cancer |
| Gancao | MOL005020 | dehydroglyasperins C | CDK2 | gastric cancer |
| Gancao | MOL000497 | licochalcone a | CDK4 | gastric cancer |
| Gancao | MOL000239 | Jaranol | CHEK1 | gastric cancer |
| Gancao | MOL000354 | isorhamnetin | CHEK1 | gastric cancer |
| Gancao | MOL000392 | formononetin | CHEK1 | gastric cancer |
| Gancao | MOL000417 | Calycosin | CHEK1 | gastric cancer |
| Gancao | MOL000497 | licochalcone a | CHEK1 | gastric cancer |
| Gancao | MOL000500 | Vestitol | CHEK1 | gastric cancer |
| Gancao | MOL002311 | Glycyrol | CHEK1 | gastric cancer |
| Gancao | MOL003656 | Lupiwighteone | CHEK1 | gastric cancer |
| Gancao | MOL003896 | 7-Methoxy-2-methyl isoflavone | CHEK1 | gastric cancer |
| Gancao | MOL004811 | Glyasperin C | CHEK1 | gastric cancer |
| Gancao | MOL004814 | Isotrifoliol | CHEK1 | gastric cancer |
| Gancao | MOL004815 | (E)-1-(2,4-dihydroxyphenyl)-3-(2,2-dimethylchromen-6-yl)prop-2-en-1-one | CHEK1 | gastric cancer |
| Gancao | MOL004820 | kanzonols W | CHEK1 | gastric cancer |
| Gancao | MOL004824 | (2S)-6-(2,4-dihydroxyphenyl)-2-(2-hydroxypropan-2-yl)-4-methoxy-2,3-dihydrofuro[3,2-g]chromen-7-one | CHEK1 | gastric cancer |
| Gancao | MOL004827 | Semilicoisoflavone B | CHEK1 | gastric cancer |
| Gancao | MOL004828 | Glepidotin A | CHEK1 | gastric cancer |
| Gancao | MOL004833 | Phaseolinisoflavan | CHEK1 | gastric cancer |
| Gancao | MOL004835 | Glypallichalcone | CHEK1 | gastric cancer |
| Gancao | MOL004841 | Licochalcone B | CHEK1 | gastric cancer |
| Gancao | MOL004849 | 3-(2,4-dihydroxyphenyl)-8-(1,1-dimethylprop-2-enyl)-7-hydroxy-5-methoxy-coumarin | CHEK1 | gastric cancer |
| Gancao | MOL004855 | Licoricone | CHEK1 | gastric cancer |
| Gancao | MOL004856 | Gancaonin A | CHEK1 | gastric cancer |
| Gancao | MOL004857 | Gancaonin B | CHEK1 | gastric cancer |
| Gancao | MOL004863 | 3-(3,4-dihydroxyphenyl)-5,7-dihydroxy-8-(3-methylbut-2-enyl)chromone | CHEK1 | gastric cancer |
| Gancao | MOL004866 | 2-(3,4-dihydroxyphenyl)-5,7-dihydroxy-6-(3-methylbut-2-enyl)chromone | CHEK1 | gastric cancer |
| Gancao | MOL004879 | Glycyrin | CHEK1 | gastric cancer |
| Gancao | MOL004883 | Licoisoflavone | CHEK1 | gastric cancer |
| Gancao | MOL004884 | Licoisoflavone B | CHEK1 | gastric cancer |
| Gancao | MOL004907 | Glyzaglabrin | CHEK1 | gastric cancer |
| Gancao | MOL004908 | Glabridin | CHEK1 | gastric cancer |
| Gancao | MOL004912 | Glabrone | CHEK1 | gastric cancer |
| Gancao | MOL004913 | 1,3-dihydroxy-9-methoxy-6-benzofurano[3,2-c]chromenone | CHEK1 | gastric cancer |
| Gancao | MOL004914 | 1,3-dihydroxy-8,9-dimethoxy-6-benzofurano[3,2-c]chromenone | CHEK1 | gastric cancer |
| Gancao | MOL004915 | Eurycarpin A | CHEK1 | gastric cancer |
| Gancao | MOL004957 | HMO | CHEK1 | gastric cancer |
| Gancao | MOL004966 | 3'-Hydroxy-4'-O-Methylglabridin | CHEK1 | gastric cancer |
| Gancao | MOL004974 | 3'-Methoxyglabridin | CHEK1 | gastric cancer |
| Gancao | MOL004978 | 2-[(3R)-8,8-dimethyl-3,4-dihydro-2H-pyrano[6,5-f]chromen-3-yl]-5-methoxyphenol | CHEK1 | gastric cancer |
| Gancao | MOL004990 | 7,2',4'-trihydroxy－5-methoxy-3－arylcoumarin | CHEK1 | gastric cancer |
| Gancao | MOL004991 | 7-Acetoxy-2-methylisoflavone | CHEK1 | gastric cancer |
| Gancao | MOL005000 | Gancaonin G | CHEK1 | gastric cancer |
| Gancao | MOL005012 | Licoagroisoflavone | CHEK1 | gastric cancer |
| Gancao | MOL005016 | Odoratin | CHEK1 | gastric cancer |
| Gancao | MOL005017 | Phaseol | CHEK1 | gastric cancer |
| Gancao | MOL005020 | dehydroglyasperins C | CHEK1 | gastric cancer |
| Gancao | MOL000392 | formononetin | CHRM1 | gastric cancer |
| Gancao | MOL000422 | kaempferol | CHRM1 | gastric cancer |
| Gancao | MOL000497 | licochalcone a | CHRM1 | gastric cancer |
| Gancao | MOL000500 | Vestitol | CHRM1 | gastric cancer |
| Gancao | MOL001484 | Inermine | CHRM1 | gastric cancer |
| Gancao | MOL002565 | Medicarpin | CHRM1 | gastric cancer |
| Gancao | MOL003896 | 7-Methoxy-2-methyl isoflavone | CHRM1 | gastric cancer |
| Gancao | MOL004833 | Phaseolinisoflavan | CHRM1 | gastric cancer |
| Gancao | MOL004835 | Glypallichalcone | CHRM1 | gastric cancer |
| Gancao | MOL004891 | shinpterocarpin | CHRM1 | gastric cancer |
| Gancao | MOL004908 | Glabridin | CHRM1 | gastric cancer |
| Gancao | MOL004957 | HMO | CHRM1 | gastric cancer |
| Gancao | MOL004978 | 2-[(3R)-8,8-dimethyl-3,4-dihydro-2H-pyrano[6,5-f]chromen-3-yl]-5-methoxyphenol | CHRM1 | gastric cancer |
| Gancao | MOL005003 | Licoagrocarpin | CHRM1 | gastric cancer |
| Gancao | MOL000422 | kaempferol | CHRM2 | gastric cancer |
| Gancao | MOL002565 | Medicarpin | CHRM2 | gastric cancer |
| Gancao | MOL001484 | Inermine | CHRM3 | gastric cancer |
| Gancao | MOL002565 | Medicarpin | CHRM3 | gastric cancer |
| Gancao | MOL003896 | 7-Methoxy-2-methyl isoflavone | CHRM3 | gastric cancer |
| Gancao | MOL004891 | shinpterocarpin | CHRM3 | gastric cancer |
| Gancao | MOL004978 | 2-[(3R)-8,8-dimethyl-3,4-dihydro-2H-pyrano[6,5-f]chromen-3-yl]-5-methoxyphenol | CHRM3 | gastric cancer |
| Gancao | MOL005003 | Licoagrocarpin | CHRM3 | gastric cancer |
| Gancao | MOL002565 | Medicarpin | CHRNA7 | gastric cancer |
| Gancao | MOL003896 | 7-Methoxy-2-methyl isoflavone | CHRNA7 | gastric cancer |
| Gancao | MOL004891 | shinpterocarpin | CHRNA7 | gastric cancer |
| Gancao | MOL000098 | quercetin | CTSD | gastric cancer |
| Gancao | MOL000098 | quercetin | CYP1A2 | gastric cancer |
| Gancao | MOL000422 | kaempferol | CYP1A2 | gastric cancer |
| Gancao | MOL000098 | quercetin | CYP3A4 | gastric cancer |
| Gancao | MOL000422 | kaempferol | CYP3A4 | gastric cancer |
| Gancao | MOL000392 | formononetin | DPEP1 | gastric cancer |
| Gancao | MOL001792 | DFV | DPEP1 | gastric cancer |
| Gancao | MOL002844 | Pinocembrin | DPEP1 | gastric cancer |
| Gancao | MOL004328 | naringenin | DPEP1 | gastric cancer |
| Gancao | MOL003896 | 7-Methoxy-2-methyl isoflavone | DRD1 | gastric cancer |
| Gancao | MOL000098 | quercetin | EGF | gastric cancer |
| Gancao | MOL000098 | quercetin | EGFR | gastric cancer |
| Gancao | MOL000354 | isorhamnetin | ESR1 | gastric cancer |
| Gancao | MOL000392 | formononetin | ESR1 | gastric cancer |
| Gancao | MOL000417 | Calycosin | ESR1 | gastric cancer |
| Gancao | MOL000497 | licochalcone a | ESR1 | gastric cancer |
| Gancao | MOL000500 | Vestitol | ESR1 | gastric cancer |
| Gancao | MOL001792 | DFV | ESR1 | gastric cancer |
| Gancao | MOL002311 | Glycyrol | ESR1 | gastric cancer |
| Gancao | MOL002565 | Medicarpin | ESR1 | gastric cancer |
| Gancao | MOL002844 | Pinocembrin | ESR1 | gastric cancer |
| Gancao | MOL003656 | Lupiwighteone | ESR1 | gastric cancer |
| Gancao | MOL003896 | 7-Methoxy-2-methyl isoflavone | ESR1 | gastric cancer |
| Gancao | MOL004328 | naringenin | ESR1 | gastric cancer |
| Gancao | MOL004805 | (2S)-2-[4-hydroxy-3-(3-methylbut-2-enyl)phenyl]-8,8-dimethyl-2,3-dihydropyrano[2,3-f]chromen-4-one | ESR1 | gastric cancer |
| Gancao | MOL004806 | euchrenone | ESR1 | gastric cancer |
| Gancao | MOL004808 | glyasperin B | ESR1 | gastric cancer |
| Gancao | MOL004810 | glyasperin F | ESR1 | gastric cancer |
| Gancao | MOL004811 | Glyasperin C | ESR1 | gastric cancer |
| Gancao | MOL004814 | Isotrifoliol | ESR1 | gastric cancer |
| Gancao | MOL004815 | (E)-1-(2,4-dihydroxyphenyl)-3-(2,2-dimethylchromen-6-yl)prop-2-en-1-one | ESR1 | gastric cancer |
| Gancao | MOL004820 | kanzonols W | ESR1 | gastric cancer |
| Gancao | MOL004824 | (2S)-6-(2,4-dihydroxyphenyl)-2-(2-hydroxypropan-2-yl)-4-methoxy-2,3-dihydrofuro[3,2-g]chromen-7-one | ESR1 | gastric cancer |
| Gancao | MOL004827 | Semilicoisoflavone B | ESR1 | gastric cancer |
| Gancao | MOL004828 | Glepidotin A | ESR1 | gastric cancer |
| Gancao | MOL004829 | Glepidotin B | ESR1 | gastric cancer |
| Gancao | MOL004833 | Phaseolinisoflavan | ESR1 | gastric cancer |
| Gancao | MOL004835 | Glypallichalcone | ESR1 | gastric cancer |
| Gancao | MOL004838 | 8-(6-hydroxy-2-benzofuranyl)-2,2-dimethyl-5-chromenol | ESR1 | gastric cancer |
| Gancao | MOL004841 | Licochalcone B | ESR1 | gastric cancer |
| Gancao | MOL004848 | licochalcone G | ESR1 | gastric cancer |
| Gancao | MOL004849 | 3-(2,4-dihydroxyphenyl)-8-(1,1-dimethylprop-2-enyl)-7-hydroxy-5-methoxy-coumarin | ESR1 | gastric cancer |
| Gancao | MOL004855 | Licoricone | ESR1 | gastric cancer |
| Gancao | MOL004856 | Gancaonin A | ESR1 | gastric cancer |
| Gancao | MOL004857 | Gancaonin B | ESR1 | gastric cancer |
| Gancao | MOL004863 | 3-(3,4-dihydroxyphenyl)-5,7-dihydroxy-8-(3-methylbut-2-enyl)chromone | ESR1 | gastric cancer |
| Gancao | MOL004866 | 2-(3,4-dihydroxyphenyl)-5,7-dihydroxy-6-(3-methylbut-2-enyl)chromone | ESR1 | gastric cancer |
| Gancao | MOL004879 | Glycyrin | ESR1 | gastric cancer |
| Gancao | MOL004882 | Licocoumarone | ESR1 | gastric cancer |
| Gancao | MOL004883 | Licoisoflavone | ESR1 | gastric cancer |
| Gancao | MOL004884 | Licoisoflavone B | ESR1 | gastric cancer |
| Gancao | MOL004885 | licoisoflavanone | ESR1 | gastric cancer |
| Gancao | MOL004891 | shinpterocarpin | ESR1 | gastric cancer |
| Gancao | MOL004898 | (E)-3-[3,4-dihydroxy-5-(3-methylbut-2-enyl)phenyl]-1-(2,4-dihydroxyphenyl)prop-2-en-1-one | ESR1 | gastric cancer |
| Gancao | MOL004904 | licopyranocoumarin | ESR1 | gastric cancer |
| Gancao | MOL004907 | Glyzaglabrin | ESR1 | gastric cancer |
| Gancao | MOL004908 | Glabridin | ESR1 | gastric cancer |
| Gancao | MOL004910 | Glabranin | ESR1 | gastric cancer |
| Gancao | MOL004911 | Glabrene | ESR1 | gastric cancer |
| Gancao | MOL004912 | Glabrone | ESR1 | gastric cancer |
| Gancao | MOL004913 | 1,3-dihydroxy-9-methoxy-6-benzofurano[3,2-c]chromenone | ESR1 | gastric cancer |
| Gancao | MOL004914 | 1,3-dihydroxy-8,9-dimethoxy-6-benzofurano[3,2-c]chromenone | ESR1 | gastric cancer |
| Gancao | MOL004915 | Eurycarpin A | ESR1 | gastric cancer |
| Gancao | MOL004935 | Sigmoidin-B | ESR1 | gastric cancer |
| Gancao | MOL004941 | (2R)-7-hydroxy-2-(4-hydroxyphenyl)chroman-4-one | ESR1 | gastric cancer |
| Gancao | MOL004945 | (2S)-7-hydroxy-2-(4-hydroxyphenyl)-8-(3-methylbut-2-enyl)chroman-4-one | ESR1 | gastric cancer |
| Gancao | MOL004948 | Isoglycyrol | ESR1 | gastric cancer |
| Gancao | MOL004949 | Isolicoflavonol | ESR1 | gastric cancer |
| Gancao | MOL004957 | HMO | ESR1 | gastric cancer |
| Gancao | MOL004959 | 1-Methoxyphaseollidin | ESR1 | gastric cancer |
| Gancao | MOL004961 | Quercetin der. | ESR1 | gastric cancer |
| Gancao | MOL004966 | 3'-Hydroxy-4'-O-Methylglabridin | ESR1 | gastric cancer |
| Gancao | MOL004974 | 3'-Methoxyglabridin | ESR1 | gastric cancer |
| Gancao | MOL004978 | 2-[(3R)-8,8-dimethyl-3,4-dihydro-2H-pyrano[6,5-f]chromen-3-yl]-5-methoxyphenol | ESR1 | gastric cancer |
| Gancao | MOL004980 | Inflacoumarin A | ESR1 | gastric cancer |
| Gancao | MOL004988 | Kanzonol F | ESR1 | gastric cancer |
| Gancao | MOL004989 | 6-prenylated eriodictyol | ESR1 | gastric cancer |
| Gancao | MOL004990 | 7,2',4'-trihydroxy－5-methoxy-3－arylcoumarin | ESR1 | gastric cancer |
| Gancao | MOL004991 | 7-Acetoxy-2-methylisoflavone | ESR1 | gastric cancer |
| Gancao | MOL004993 | 8-prenylated eriodictyol | ESR1 | gastric cancer |
| Gancao | MOL005000 | Gancaonin G | ESR1 | gastric cancer |
| Gancao | MOL005001 | Gancaonin H | ESR1 | gastric cancer |
| Gancao | MOL005003 | Licoagrocarpin | ESR1 | gastric cancer |
| Gancao | MOL005007 | Glyasperins M | ESR1 | gastric cancer |
| Gancao | MOL005008 | Glycyrrhiza flavonol A | ESR1 | gastric cancer |
| Gancao | MOL005012 | Licoagroisoflavone | ESR1 | gastric cancer |
| Gancao | MOL005016 | Odoratin | ESR1 | gastric cancer |
| Gancao | MOL005017 | Phaseol | ESR1 | gastric cancer |
| Gancao | MOL005018 | Xambioona | ESR1 | gastric cancer |
| Gancao | MOL005020 | dehydroglyasperins C | ESR1 | gastric cancer |
| Gancao | MOL000239 | Jaranol | ESR2 | gastric cancer |
| Gancao | MOL000354 | isorhamnetin | ESR2 | gastric cancer |
| Gancao | MOL000392 | formononetin | ESR2 | gastric cancer |
| Gancao | MOL000417 | Calycosin | ESR2 | gastric cancer |
| Gancao | MOL000497 | licochalcone a | ESR2 | gastric cancer |
| Gancao | MOL000500 | Vestitol | ESR2 | gastric cancer |
| Gancao | MOL002565 | Medicarpin | ESR2 | gastric cancer |
| Gancao | MOL003656 | Lupiwighteone | ESR2 | gastric cancer |
| Gancao | MOL003896 | 7-Methoxy-2-methyl isoflavone | ESR2 | gastric cancer |
| Gancao | MOL004805 | (2S)-2-[4-hydroxy-3-(3-methylbut-2-enyl)phenyl]-8,8-dimethyl-2,3-dihydropyrano[2,3-f]chromen-4-one | ESR2 | gastric cancer |
| Gancao | MOL004806 | euchrenone | ESR2 | gastric cancer |
| Gancao | MOL004808 | glyasperin B | ESR2 | gastric cancer |
| Gancao | MOL004810 | glyasperin F | ESR2 | gastric cancer |
| Gancao | MOL004811 | Glyasperin C | ESR2 | gastric cancer |
| Gancao | MOL004814 | Isotrifoliol | ESR2 | gastric cancer |
| Gancao | MOL004815 | (E)-1-(2,4-dihydroxyphenyl)-3-(2,2-dimethylchromen-6-yl)prop-2-en-1-one | ESR2 | gastric cancer |
| Gancao | MOL004820 | kanzonols W | ESR2 | gastric cancer |
| Gancao | MOL004824 | (2S)-6-(2,4-dihydroxyphenyl)-2-(2-hydroxypropan-2-yl)-4-methoxy-2,3-dihydrofuro[3,2-g]chromen-7-one | ESR2 | gastric cancer |
| Gancao | MOL004833 | Phaseolinisoflavan | ESR2 | gastric cancer |
| Gancao | MOL004835 | Glypallichalcone | ESR2 | gastric cancer |
| Gancao | MOL004841 | Licochalcone B | ESR2 | gastric cancer |
| Gancao | MOL004848 | licochalcone G | ESR2 | gastric cancer |
| Gancao | MOL004849 | 3-(2,4-dihydroxyphenyl)-8-(1,1-dimethylprop-2-enyl)-7-hydroxy-5-methoxy-coumarin | ESR2 | gastric cancer |
| Gancao | MOL004856 | Gancaonin A | ESR2 | gastric cancer |
| Gancao | MOL004857 | Gancaonin B | ESR2 | gastric cancer |
| Gancao | MOL004866 | 2-(3,4-dihydroxyphenyl)-5,7-dihydroxy-6-(3-methylbut-2-enyl)chromone | ESR2 | gastric cancer |
| Gancao | MOL004879 | Glycyrin | ESR2 | gastric cancer |
| Gancao | MOL004882 | Licocoumarone | ESR2 | gastric cancer |
| Gancao | MOL004884 | Licoisoflavone B | ESR2 | gastric cancer |
| Gancao | MOL004885 | licoisoflavanone | ESR2 | gastric cancer |
| Gancao | MOL004891 | shinpterocarpin | ESR2 | gastric cancer |
| Gancao | MOL004907 | Glyzaglabrin | ESR2 | gastric cancer |
| Gancao | MOL004908 | Glabridin | ESR2 | gastric cancer |
| Gancao | MOL004911 | Glabrene | ESR2 | gastric cancer |
| Gancao | MOL004912 | Glabrone | ESR2 | gastric cancer |
| Gancao | MOL004913 | 1,3-dihydroxy-9-methoxy-6-benzofurano[3,2-c]chromenone | ESR2 | gastric cancer |
| Gancao | MOL004915 | Eurycarpin A | ESR2 | gastric cancer |
| Gancao | MOL004945 | (2S)-7-hydroxy-2-(4-hydroxyphenyl)-8-(3-methylbut-2-enyl)chroman-4-one | ESR2 | gastric cancer |
| Gancao | MOL004957 | HMO | ESR2 | gastric cancer |
| Gancao | MOL004959 | 1-Methoxyphaseollidin | ESR2 | gastric cancer |
| Gancao | MOL004961 | Quercetin der. | ESR2 | gastric cancer |
| Gancao | MOL004966 | 3'-Hydroxy-4'-O-Methylglabridin | ESR2 | gastric cancer |
| Gancao | MOL004974 | 3'-Methoxyglabridin | ESR2 | gastric cancer |
| Gancao | MOL004978 | 2-[(3R)-8,8-dimethyl-3,4-dihydro-2H-pyrano[6,5-f]chromen-3-yl]-5-methoxyphenol | ESR2 | gastric cancer |
| Gancao | MOL004988 | Kanzonol F | ESR2 | gastric cancer |
| Gancao | MOL004990 | 7,2',4'-trihydroxy－5-methoxy-3－arylcoumarin | ESR2 | gastric cancer |
| Gancao | MOL005000 | Gancaonin G | ESR2 | gastric cancer |
| Gancao | MOL005003 | Licoagrocarpin | ESR2 | gastric cancer |
| Gancao | MOL005007 | Glyasperins M | ESR2 | gastric cancer |
| Gancao | MOL005008 | Glycyrrhiza flavonol A | ESR2 | gastric cancer |
| Gancao | MOL005012 | Licoagroisoflavone | ESR2 | gastric cancer |
| Gancao | MOL005016 | Odoratin | ESR2 | gastric cancer |
| Gancao | MOL005018 | Xambioona | ESR2 | gastric cancer |
| Gancao | MOL005020 | dehydroglyasperins C | ESR2 | gastric cancer |
| Gancao | MOL000098 | quercetin | F10 | gastric cancer |
| Gancao | MOL000497 | licochalcone a | F10 | gastric cancer |
| Gancao | MOL003656 | Lupiwighteone | F10 | gastric cancer |
| Gancao | MOL004805 | (2S)-2-[4-hydroxy-3-(3-methylbut-2-enyl)phenyl]-8,8-dimethyl-2,3-dihydropyrano[2,3-f]chromen-4-one | F10 | gastric cancer |
| Gancao | MOL004806 | euchrenone | F10 | gastric cancer |
| Gancao | MOL004808 | glyasperin B | F10 | gastric cancer |
| Gancao | MOL004810 | glyasperin F | F10 | gastric cancer |
| Gancao | MOL004811 | Glyasperin C | F10 | gastric cancer |
| Gancao | MOL004815 | (E)-1-(2,4-dihydroxyphenyl)-3-(2,2-dimethylchromen-6-yl)prop-2-en-1-one | F10 | gastric cancer |
| Gancao | MOL004820 | kanzonols W | F10 | gastric cancer |
| Gancao | MOL004824 | (2S)-6-(2,4-dihydroxyphenyl)-2-(2-hydroxypropan-2-yl)-4-methoxy-2,3-dihydrofuro[3,2-g]chromen-7-one | F10 | gastric cancer |
| Gancao | MOL004827 | Semilicoisoflavone B | F10 | gastric cancer |
| Gancao | MOL004828 | Glepidotin A | F10 | gastric cancer |
| Gancao | MOL004829 | Glepidotin B | F10 | gastric cancer |
| Gancao | MOL004833 | Phaseolinisoflavan | F10 | gastric cancer |
| Gancao | MOL004848 | licochalcone G | F10 | gastric cancer |
| Gancao | MOL004849 | 3-(2,4-dihydroxyphenyl)-8-(1,1-dimethylprop-2-enyl)-7-hydroxy-5-methoxy-coumarin | F10 | gastric cancer |
| Gancao | MOL004855 | Licoricone | F10 | gastric cancer |
| Gancao | MOL004856 | Gancaonin A | F10 | gastric cancer |
| Gancao | MOL004857 | Gancaonin B | F10 | gastric cancer |
| Gancao | MOL004863 | 3-(3,4-dihydroxyphenyl)-5,7-dihydroxy-8-(3-methylbut-2-enyl)chromone | F10 | gastric cancer |
| Gancao | MOL004866 | 2-(3,4-dihydroxyphenyl)-5,7-dihydroxy-6-(3-methylbut-2-enyl)chromone | F10 | gastric cancer |
| Gancao | MOL004879 | Glycyrin | F10 | gastric cancer |
| Gancao | MOL004883 | Licoisoflavone | F10 | gastric cancer |
| Gancao | MOL004884 | Licoisoflavone B | F10 | gastric cancer |
| Gancao | MOL004885 | licoisoflavanone | F10 | gastric cancer |
| Gancao | MOL004903 | liquiritin | F10 | gastric cancer |
| Gancao | MOL004904 | licopyranocoumarin | F10 | gastric cancer |
| Gancao | MOL004910 | Glabranin | F10 | gastric cancer |
| Gancao | MOL004911 | Glabrene | F10 | gastric cancer |
| Gancao | MOL004912 | Glabrone | F10 | gastric cancer |
| Gancao | MOL004915 | Eurycarpin A | F10 | gastric cancer |
| Gancao | MOL004935 | Sigmoidin-B | F10 | gastric cancer |
| Gancao | MOL004945 | (2S)-7-hydroxy-2-(4-hydroxyphenyl)-8-(3-methylbut-2-enyl)chroman-4-one | F10 | gastric cancer |
| Gancao | MOL004949 | Isolicoflavonol | F10 | gastric cancer |
| Gancao | MOL004959 | 1-Methoxyphaseollidin | F10 | gastric cancer |
| Gancao | MOL004966 | 3'-Hydroxy-4'-O-Methylglabridin | F10 | gastric cancer |
| Gancao | MOL004974 | 3'-Methoxyglabridin | F10 | gastric cancer |
| Gancao | MOL004978 | 2-[(3R)-8,8-dimethyl-3,4-dihydro-2H-pyrano[6,5-f]chromen-3-yl]-5-methoxyphenol | F10 | gastric cancer |
| Gancao | MOL004980 | Inflacoumarin A | F10 | gastric cancer |
| Gancao | MOL004988 | Kanzonol F | F10 | gastric cancer |
| Gancao | MOL004989 | 6-prenylated eriodictyol | F10 | gastric cancer |
| Gancao | MOL004993 | 8-prenylated eriodictyol | F10 | gastric cancer |
| Gancao | MOL005000 | Gancaonin G | F10 | gastric cancer |
| Gancao | MOL005001 | Gancaonin H | F10 | gastric cancer |
| Gancao | MOL005003 | Licoagrocarpin | F10 | gastric cancer |
| Gancao | MOL005007 | Glyasperins M | F10 | gastric cancer |
| Gancao | MOL005008 | Glycyrrhiza flavonol A | F10 | gastric cancer |
| Gancao | MOL005012 | Licoagroisoflavone | F10 | gastric cancer |
| Gancao | MOL005018 | Xambioona | F10 | gastric cancer |
| Gancao | MOL005020 | dehydroglyasperins C | F10 | gastric cancer |
| Gancao | MOL004808 | glyasperin B | F2 | gastric cancer |
| Gancao | MOL004811 | Glyasperin C | F2 | gastric cancer |
| Gancao | MOL004824 | (2S)-6-(2,4-dihydroxyphenyl)-2-(2-hydroxypropan-2-yl)-4-methoxy-2,3-dihydrofuro[3,2-g]chromen-7-one | F2 | gastric cancer |
| Gancao | MOL004827 | Semilicoisoflavone B | F2 | gastric cancer |
| Gancao | MOL004828 | Glepidotin A | F2 | gastric cancer |
| Gancao | MOL004849 | 3-(2,4-dihydroxyphenyl)-8-(1,1-dimethylprop-2-enyl)-7-hydroxy-5-methoxy-coumarin | F2 | gastric cancer |
| Gancao | MOL004855 | Licoricone | F2 | gastric cancer |
| Gancao | MOL004856 | Gancaonin A | F2 | gastric cancer |
| Gancao | MOL004857 | Gancaonin B | F2 | gastric cancer |
| Gancao | MOL004863 | 3-(3,4-dihydroxyphenyl)-5,7-dihydroxy-8-(3-methylbut-2-enyl)chromone | F2 | gastric cancer |
| Gancao | MOL004879 | Glycyrin | F2 | gastric cancer |
| Gancao | MOL004883 | Licoisoflavone | F2 | gastric cancer |
| Gancao | MOL004884 | Licoisoflavone B | F2 | gastric cancer |
| Gancao | MOL004904 | licopyranocoumarin | F2 | gastric cancer |
| Gancao | MOL004912 | Glabrone | F2 | gastric cancer |
| Gancao | MOL004915 | Eurycarpin A | F2 | gastric cancer |
| Gancao | MOL004949 | Isolicoflavonol | F2 | gastric cancer |
| Gancao | MOL004959 | 1-Methoxyphaseollidin | F2 | gastric cancer |
| Gancao | MOL004980 | Inflacoumarin A | F2 | gastric cancer |
| Gancao | MOL004991 | 7-Acetoxy-2-methylisoflavone | F2 | gastric cancer |
| Gancao | MOL005000 | Gancaonin G | F2 | gastric cancer |
| Gancao | MOL005003 | Licoagrocarpin | F2 | gastric cancer |
| Gancao | MOL005012 | Licoagroisoflavone | F2 | gastric cancer |
| Gancao | MOL005017 | Phaseol | F2 | gastric cancer |
| Gancao | MOL000098 | quercetin | F3 | gastric cancer |
| Gancao | MOL000098 | quercetin | F7 | gastric cancer |
| Gancao | MOL000354 | isorhamnetin | F7 | gastric cancer |
| Gancao | MOL000422 | kaempferol | F7 | gastric cancer |
| Gancao | MOL004808 | glyasperin B | F7 | gastric cancer |
| Gancao | MOL004824 | (2S)-6-(2,4-dihydroxyphenyl)-2-(2-hydroxypropan-2-yl)-4-methoxy-2,3-dihydrofuro[3,2-g]chromen-7-one | F7 | gastric cancer |
| Gancao | MOL004827 | Semilicoisoflavone B | F7 | gastric cancer |
| Gancao | MOL004828 | Glepidotin A | F7 | gastric cancer |
| Gancao | MOL004829 | Glepidotin B | F7 | gastric cancer |
| Gancao | MOL004849 | 3-(2,4-dihydroxyphenyl)-8-(1,1-dimethylprop-2-enyl)-7-hydroxy-5-methoxy-coumarin | F7 | gastric cancer |
| Gancao | MOL004857 | Gancaonin B | F7 | gastric cancer |
| Gancao | MOL004885 | licoisoflavanone | F7 | gastric cancer |
| Gancao | MOL004903 | liquiritin | F7 | gastric cancer |
| Gancao | MOL004904 | licopyranocoumarin | F7 | gastric cancer |
| Gancao | MOL004966 | 3'-Hydroxy-4'-O-Methylglabridin | F7 | gastric cancer |
| Gancao | MOL004974 | 3'-Methoxyglabridin | F7 | gastric cancer |
| Gancao | MOL004989 | 6-prenylated eriodictyol | F7 | gastric cancer |
| Gancao | MOL004993 | 8-prenylated eriodictyol | F7 | gastric cancer |
| Gancao | MOL005007 | Glyasperins M | F7 | gastric cancer |
| Gancao | MOL005008 | Glycyrrhiza flavonol A | F7 | gastric cancer |
| Gancao | MOL004328 | naringenin | FASN | gastric cancer |
| Gancao | MOL000098 | quercetin | GJA1 | gastric cancer |
| Gancao | MOL000354 | isorhamnetin | GSK3B | gastric cancer |
| Gancao | MOL000392 | formononetin | GSK3B | gastric cancer |
| Gancao | MOL000417 | Calycosin | GSK3B | gastric cancer |
| Gancao | MOL000497 | licochalcone a | GSK3B | gastric cancer |
| Gancao | MOL000500 | Vestitol | GSK3B | gastric cancer |
| Gancao | MOL002311 | Glycyrol | GSK3B | gastric cancer |
| Gancao | MOL003656 | Lupiwighteone | GSK3B | gastric cancer |
| Gancao | MOL003896 | 7-Methoxy-2-methyl isoflavone | GSK3B | gastric cancer |
| Gancao | MOL004805 | (2S)-2-[4-hydroxy-3-(3-methylbut-2-enyl)phenyl]-8,8-dimethyl-2,3-dihydropyrano[2,3-f]chromen-4-one | GSK3B | gastric cancer |
| Gancao | MOL004808 | glyasperin B | GSK3B | gastric cancer |
| Gancao | MOL004810 | glyasperin F | GSK3B | gastric cancer |
| Gancao | MOL004811 | Glyasperin C | GSK3B | gastric cancer |
| Gancao | MOL004814 | Isotrifoliol | GSK3B | gastric cancer |
| Gancao | MOL004815 | (E)-1-(2,4-dihydroxyphenyl)-3-(2,2-dimethylchromen-6-yl)prop-2-en-1-one | GSK3B | gastric cancer |
| Gancao | MOL004820 | kanzonols W | GSK3B | gastric cancer |
| Gancao | MOL004824 | (2S)-6-(2,4-dihydroxyphenyl)-2-(2-hydroxypropan-2-yl)-4-methoxy-2,3-dihydrofuro[3,2-g]chromen-7-one | GSK3B | gastric cancer |
| Gancao | MOL004827 | Semilicoisoflavone B | GSK3B | gastric cancer |
| Gancao | MOL004828 | Glepidotin A | GSK3B | gastric cancer |
| Gancao | MOL004833 | Phaseolinisoflavan | GSK3B | gastric cancer |
| Gancao | MOL004835 | Glypallichalcone | GSK3B | gastric cancer |
| Gancao | MOL004841 | Licochalcone B | GSK3B | gastric cancer |
| Gancao | MOL004848 | licochalcone G | GSK3B | gastric cancer |
| Gancao | MOL004849 | 3-(2,4-dihydroxyphenyl)-8-(1,1-dimethylprop-2-enyl)-7-hydroxy-5-methoxy-coumarin | GSK3B | gastric cancer |
| Gancao | MOL004856 | Gancaonin A | GSK3B | gastric cancer |
| Gancao | MOL004857 | Gancaonin B | GSK3B | gastric cancer |
| Gancao | MOL004863 | 3-(3,4-dihydroxyphenyl)-5,7-dihydroxy-8-(3-methylbut-2-enyl)chromone | GSK3B | gastric cancer |
| Gancao | MOL004866 | 2-(3,4-dihydroxyphenyl)-5,7-dihydroxy-6-(3-methylbut-2-enyl)chromone | GSK3B | gastric cancer |
| Gancao | MOL004882 | Licocoumarone | GSK3B | gastric cancer |
| Gancao | MOL004884 | Licoisoflavone B | GSK3B | gastric cancer |
| Gancao | MOL004885 | licoisoflavanone | GSK3B | gastric cancer |
| Gancao | MOL004891 | shinpterocarpin | GSK3B | gastric cancer |
| Gancao | MOL004898 | (E)-3-[3,4-dihydroxy-5-(3-methylbut-2-enyl)phenyl]-1-(2,4-dihydroxyphenyl)prop-2-en-1-one | GSK3B | gastric cancer |
| Gancao | MOL004907 | Glyzaglabrin | GSK3B | gastric cancer |
| Gancao | MOL004908 | Glabridin | GSK3B | gastric cancer |
| Gancao | MOL004911 | Glabrene | GSK3B | gastric cancer |
| Gancao | MOL004912 | Glabrone | GSK3B | gastric cancer |
| Gancao | MOL004913 | 1,3-dihydroxy-9-methoxy-6-benzofurano[3,2-c]chromenone | GSK3B | gastric cancer |
| Gancao | MOL004914 | 1,3-dihydroxy-8,9-dimethoxy-6-benzofurano[3,2-c]chromenone | GSK3B | gastric cancer |
| Gancao | MOL004915 | Eurycarpin A | GSK3B | gastric cancer |
| Gancao | MOL004948 | Isoglycyrol | GSK3B | gastric cancer |
| Gancao | MOL004949 | Isolicoflavonol | GSK3B | gastric cancer |
| Gancao | MOL004957 | HMO | GSK3B | gastric cancer |
| Gancao | MOL004959 | 1-Methoxyphaseollidin | GSK3B | gastric cancer |
| Gancao | MOL004961 | Quercetin der. | GSK3B | gastric cancer |
| Gancao | MOL004966 | 3'-Hydroxy-4'-O-Methylglabridin | GSK3B | gastric cancer |
| Gancao | MOL004974 | 3'-Methoxyglabridin | GSK3B | gastric cancer |
| Gancao | MOL004978 | 2-[(3R)-8,8-dimethyl-3,4-dihydro-2H-pyrano[6,5-f]chromen-3-yl]-5-methoxyphenol | GSK3B | gastric cancer |
| Gancao | MOL004990 | 7,2',4'-trihydroxy－5-methoxy-3－arylcoumarin | GSK3B | gastric cancer |
| Gancao | MOL004991 | 7-Acetoxy-2-methylisoflavone | GSK3B | gastric cancer |
| Gancao | MOL005000 | Gancaonin G | GSK3B | gastric cancer |
| Gancao | MOL005003 | Licoagrocarpin | GSK3B | gastric cancer |
| Gancao | MOL005007 | Glyasperins M | GSK3B | gastric cancer |
| Gancao | MOL005008 | Glycyrrhiza flavonol A | GSK3B | gastric cancer |
| Gancao | MOL005012 | Licoagroisoflavone | GSK3B | gastric cancer |
| Gancao | MOL005016 | Odoratin | GSK3B | gastric cancer |
| Gancao | MOL005017 | Phaseol | GSK3B | gastric cancer |
| Gancao | MOL004328 | naringenin | GSR | gastric cancer |
| Gancao | MOL000098 | quercetin | GSTP1 | gastric cancer |
| Gancao | MOL000422 | kaempferol | GSTP1 | gastric cancer |
| Gancao | MOL004328 | naringenin | GSTP1 | gastric cancer |
| Gancao | MOL004328 | naringenin | HMGCR | gastric cancer |
| Gancao | MOL000098 | quercetin | HMOX1 | gastric cancer |
| Gancao | MOL000422 | kaempferol | HMOX1 | gastric cancer |
| Gancao | MOL000098 | quercetin | HSPA5 | gastric cancer |
| Gancao | MOL000500 | Vestitol | HTR2A | gastric cancer |
| Gancao | MOL002565 | Medicarpin | HTR2A | gastric cancer |
| Gancao | MOL001484 | Inermine | HTR3A | gastric cancer |
| Gancao | MOL004891 | shinpterocarpin | HTR3A | gastric cancer |
| Gancao | MOL000098 | quercetin | IFNG | gastric cancer |
| Gancao | MOL000098 | quercetin | IL1B | gastric cancer |
| Gancao | MOL000098 | quercetin | IL2 | gastric cancer |
| Gancao | MOL000098 | quercetin | IL6 | gastric cancer |
| Gancao | MOL000098 | quercetin | INSR | gastric cancer |
| Gancao | MOL000422 | kaempferol | INSR | gastric cancer |
| Gancao | MOL000098 | quercetin | JUN | gastric cancer |
| Gancao | MOL000392 | formononetin | JUN | gastric cancer |
| Gancao | MOL000422 | kaempferol | JUN | gastric cancer |
| Gancao | MOL000098 | quercetin | KCNH2 | gastric cancer |
| Gancao | MOL004805 | (2S)-2-[4-hydroxy-3-(3-methylbut-2-enyl)phenyl]-8,8-dimethyl-2,3-dihydropyrano[2,3-f]chromen-4-one | KCNH2 | gastric cancer |
| Gancao | MOL004806 | euchrenone | KCNH2 | gastric cancer |
| Gancao | MOL004811 | Glyasperin C | KCNH2 | gastric cancer |
| Gancao | MOL004849 | 3-(2,4-dihydroxyphenyl)-8-(1,1-dimethylprop-2-enyl)-7-hydroxy-5-methoxy-coumarin | KCNH2 | gastric cancer |
| Gancao | MOL004855 | Licoricone | KCNH2 | gastric cancer |
| Gancao | MOL004866 | 2-(3,4-dihydroxyphenyl)-5,7-dihydroxy-6-(3-methylbut-2-enyl)chromone | KCNH2 | gastric cancer |
| Gancao | MOL004879 | Glycyrin | KCNH2 | gastric cancer |
| Gancao | MOL004891 | shinpterocarpin | KCNH2 | gastric cancer |
| Gancao | MOL004959 | 1-Methoxyphaseollidin | KCNH2 | gastric cancer |
| Gancao | MOL004966 | 3'-Hydroxy-4'-O-Methylglabridin | KCNH2 | gastric cancer |
| Gancao | MOL004974 | 3'-Methoxyglabridin | KCNH2 | gastric cancer |
| Gancao | MOL004978 | 2-[(3R)-8,8-dimethyl-3,4-dihydro-2H-pyrano[6,5-f]chromen-3-yl]-5-methoxyphenol | KCNH2 | gastric cancer |
| Gancao | MOL005003 | Licoagrocarpin | KCNH2 | gastric cancer |
| Gancao | MOL005007 | Glyasperins M | KCNH2 | gastric cancer |
| Gancao | MOL004966 | 3'-Hydroxy-4'-O-Methylglabridin | KCNMA1 | gastric cancer |
| Gancao | MOL004974 | 3'-Methoxyglabridin | KCNMA1 | gastric cancer |
| Gancao | MOL004978 | 2-[(3R)-8,8-dimethyl-3,4-dihydro-2H-pyrano[6,5-f]chromen-3-yl]-5-methoxyphenol | KCNMA1 | gastric cancer |
| Gancao | MOL005007 | Glyasperins M | KCNMA1 | gastric cancer |
| Gancao | MOL002311 | Glycyrol | KDR | gastric cancer |
| Gancao | MOL004808 | glyasperin B | KDR | gastric cancer |
| Gancao | MOL004824 | (2S)-6-(2,4-dihydroxyphenyl)-2-(2-hydroxypropan-2-yl)-4-methoxy-2,3-dihydrofuro[3,2-g]chromen-7-one | KDR | gastric cancer |
| Gancao | MOL004828 | Glepidotin A | KDR | gastric cancer |
| Gancao | MOL004848 | licochalcone G | KDR | gastric cancer |
| Gancao | MOL004849 | 3-(2,4-dihydroxyphenyl)-8-(1,1-dimethylprop-2-enyl)-7-hydroxy-5-methoxy-coumarin | KDR | gastric cancer |
| Gancao | MOL004855 | Licoricone | KDR | gastric cancer |
| Gancao | MOL004857 | Gancaonin B | KDR | gastric cancer |
| Gancao | MOL004879 | Glycyrin | KDR | gastric cancer |
| Gancao | MOL004883 | Licoisoflavone | KDR | gastric cancer |
| Gancao | MOL004903 | liquiritin | KDR | gastric cancer |
| Gancao | MOL004904 | licopyranocoumarin | KDR | gastric cancer |
| Gancao | MOL004935 | Sigmoidin-B | KDR | gastric cancer |
| Gancao | MOL004959 | 1-Methoxyphaseollidin | KDR | gastric cancer |
| Gancao | MOL004966 | 3'-Hydroxy-4'-O-Methylglabridin | KDR | gastric cancer |
| Gancao | MOL005001 | Gancaonin H | KDR | gastric cancer |
| Gancao | MOL005007 | Glyasperins M | KDR | gastric cancer |
| Gancao | MOL005017 | Phaseol | KDR | gastric cancer |
| Gancao | MOL004328 | naringenin | LDLR | gastric cancer |
| Gancao | MOL003896 | 7-Methoxy-2-methyl isoflavone | LTA4H | gastric cancer |
| Gancao | MOL004835 | Glypallichalcone | LTA4H | gastric cancer |
| Gancao | MOL000098 | quercetin | MAOB | gastric cancer |
| Gancao | MOL000105 | protocatechuic acid | MAOB | gastric cancer |
| Gancao | MOL000354 | isorhamnetin | MAOB | gastric cancer |
| Gancao | MOL000392 | formononetin | MAOB | gastric cancer |
| Gancao | MOL001792 | DFV | MAOB | gastric cancer |
| Gancao | MOL002844 | Pinocembrin | MAOB | gastric cancer |
| Gancao | MOL003896 | 7-Methoxy-2-methyl isoflavone | MAOB | gastric cancer |
| Gancao | MOL004835 | Glypallichalcone | MAOB | gastric cancer |
| Gancao | MOL004941 | (2R)-7-hydroxy-2-(4-hydroxyphenyl)chroman-4-one | MAOB | gastric cancer |
| Gancao | MOL004957 | HMO | MAOB | gastric cancer |
| Gancao | MOL000098 | quercetin | MAPK1 | gastric cancer |
| Gancao | MOL000497 | licochalcone a | MAPK1 | gastric cancer |
| Gancao | MOL004328 | naringenin | MAPK1 | gastric cancer |
| Gancao | MOL002565 | Medicarpin | MAPK10 | gastric cancer |
| Gancao | MOL000354 | isorhamnetin | MAPK14 | gastric cancer |
| Gancao | MOL000392 | formononetin | MAPK14 | gastric cancer |
| Gancao | MOL000417 | Calycosin | MAPK14 | gastric cancer |
| Gancao | MOL000497 | licochalcone a | MAPK14 | gastric cancer |
| Gancao | MOL000500 | Vestitol | MAPK14 | gastric cancer |
| Gancao | MOL002311 | Glycyrol | MAPK14 | gastric cancer |
| Gancao | MOL003656 | Lupiwighteone | MAPK14 | gastric cancer |
| Gancao | MOL003896 | 7-Methoxy-2-methyl isoflavone | MAPK14 | gastric cancer |
| Gancao | MOL004805 | (2S)-2-[4-hydroxy-3-(3-methylbut-2-enyl)phenyl]-8,8-dimethyl-2,3-dihydropyrano[2,3-f]chromen-4-one | MAPK14 | gastric cancer |
| Gancao | MOL004810 | glyasperin F | MAPK14 | gastric cancer |
| Gancao | MOL004811 | Glyasperin C | MAPK14 | gastric cancer |
| Gancao | MOL004814 | Isotrifoliol | MAPK14 | gastric cancer |
| Gancao | MOL004815 | (E)-1-(2,4-dihydroxyphenyl)-3-(2,2-dimethylchromen-6-yl)prop-2-en-1-one | MAPK14 | gastric cancer |
| Gancao | MOL004820 | kanzonols W | MAPK14 | gastric cancer |
| Gancao | MOL004824 | (2S)-6-(2,4-dihydroxyphenyl)-2-(2-hydroxypropan-2-yl)-4-methoxy-2,3-dihydrofuro[3,2-g]chromen-7-one | MAPK14 | gastric cancer |
| Gancao | MOL004828 | Glepidotin A | MAPK14 | gastric cancer |
| Gancao | MOL004833 | Phaseolinisoflavan | MAPK14 | gastric cancer |
| Gancao | MOL004835 | Glypallichalcone | MAPK14 | gastric cancer |
| Gancao | MOL004841 | Licochalcone B | MAPK14 | gastric cancer |
| Gancao | MOL004848 | licochalcone G | MAPK14 | gastric cancer |
| Gancao | MOL004849 | 3-(2,4-dihydroxyphenyl)-8-(1,1-dimethylprop-2-enyl)-7-hydroxy-5-methoxy-coumarin | MAPK14 | gastric cancer |
| Gancao | MOL004863 | 3-(3,4-dihydroxyphenyl)-5,7-dihydroxy-8-(3-methylbut-2-enyl)chromone | MAPK14 | gastric cancer |
| Gancao | MOL004866 | 2-(3,4-dihydroxyphenyl)-5,7-dihydroxy-6-(3-methylbut-2-enyl)chromone | MAPK14 | gastric cancer |
| Gancao | MOL004883 | Licoisoflavone | MAPK14 | gastric cancer |
| Gancao | MOL004891 | shinpterocarpin | MAPK14 | gastric cancer |
| Gancao | MOL004898 | (E)-3-[3,4-dihydroxy-5-(3-methylbut-2-enyl)phenyl]-1-(2,4-dihydroxyphenyl)prop-2-en-1-one | MAPK14 | gastric cancer |
| Gancao | MOL004907 | Glyzaglabrin | MAPK14 | gastric cancer |
| Gancao | MOL004908 | Glabridin | MAPK14 | gastric cancer |
| Gancao | MOL004911 | Glabrene | MAPK14 | gastric cancer |
| Gancao | MOL004912 | Glabrone | MAPK14 | gastric cancer |
| Gancao | MOL004913 | 1,3-dihydroxy-9-methoxy-6-benzofurano[3,2-c]chromenone | MAPK14 | gastric cancer |
| Gancao | MOL004914 | 1,3-dihydroxy-8,9-dimethoxy-6-benzofurano[3,2-c]chromenone | MAPK14 | gastric cancer |
| Gancao | MOL004915 | Eurycarpin A | MAPK14 | gastric cancer |
| Gancao | MOL004957 | HMO | MAPK14 | gastric cancer |
| Gancao | MOL004959 | 1-Methoxyphaseollidin | MAPK14 | gastric cancer |
| Gancao | MOL004961 | Quercetin der. | MAPK14 | gastric cancer |
| Gancao | MOL004966 | 3'-Hydroxy-4'-O-Methylglabridin | MAPK14 | gastric cancer |
| Gancao | MOL004974 | 3'-Methoxyglabridin | MAPK14 | gastric cancer |
| Gancao | MOL004978 | 2-[(3R)-8,8-dimethyl-3,4-dihydro-2H-pyrano[6,5-f]chromen-3-yl]-5-methoxyphenol | MAPK14 | gastric cancer |
| Gancao | MOL004990 | 7,2',4'-trihydroxy－5-methoxy-3－arylcoumarin | MAPK14 | gastric cancer |
| Gancao | MOL004991 | 7-Acetoxy-2-methylisoflavone | MAPK14 | gastric cancer |
| Gancao | MOL005000 | Gancaonin G | MAPK14 | gastric cancer |
| Gancao | MOL005003 | Licoagrocarpin | MAPK14 | gastric cancer |
| Gancao | MOL005012 | Licoagroisoflavone | MAPK14 | gastric cancer |
| Gancao | MOL005016 | Odoratin | MAPK14 | gastric cancer |
| Gancao | MOL005017 | Phaseol | MAPK14 | gastric cancer |
| Gancao | MOL005020 | dehydroglyasperins C | MAPK14 | gastric cancer |
| Gancao | MOL004328 | naringenin | MAPK3 | gastric cancer |
| Gancao | MOL000422 | kaempferol | MAPK8 | gastric cancer |
| Gancao | MOL000098 | quercetin | MMP1 | gastric cancer |
| Gancao | MOL000422 | kaempferol | MMP1 | gastric cancer |
| Gancao | MOL000098 | quercetin | MPO | gastric cancer |
| Gancao | MOL000354 | isorhamnetin | NCOA1 | gastric cancer |
| Gancao | MOL003896 | 7-Methoxy-2-methyl isoflavone | NCOA1 | gastric cancer |
| Gancao | MOL004820 | kanzonols W | NCOA1 | gastric cancer |
| Gancao | MOL004829 | Glepidotin B | NCOA1 | gastric cancer |
| Gancao | MOL004833 | Phaseolinisoflavan | NCOA1 | gastric cancer |
| Gancao | MOL004835 | Glypallichalcone | NCOA1 | gastric cancer |
| Gancao | MOL004849 | 3-(2,4-dihydroxyphenyl)-8-(1,1-dimethylprop-2-enyl)-7-hydroxy-5-methoxy-coumarin | NCOA1 | gastric cancer |
| Gancao | MOL004885 | licoisoflavanone | NCOA1 | gastric cancer |
| Gancao | MOL004891 | shinpterocarpin | NCOA1 | gastric cancer |
| Gancao | MOL004908 | Glabridin | NCOA1 | gastric cancer |
| Gancao | MOL004959 | 1-Methoxyphaseollidin | NCOA1 | gastric cancer |
| Gancao | MOL004966 | 3'-Hydroxy-4'-O-Methylglabridin | NCOA1 | gastric cancer |
| Gancao | MOL004974 | 3'-Methoxyglabridin | NCOA1 | gastric cancer |
| Gancao | MOL004978 | 2-[(3R)-8,8-dimethyl-3,4-dihydro-2H-pyrano[6,5-f]chromen-3-yl]-5-methoxyphenol | NCOA1 | gastric cancer |
| Gancao | MOL004993 | 8-prenylated eriodictyol | NCOA1 | gastric cancer |
| Gancao | MOL005007 | Glyasperins M | NCOA1 | gastric cancer |
| Gancao | MOL000098 | quercetin | NCOA2 | gastric cancer |
| Gancao | MOL000239 | Jaranol | NCOA2 | gastric cancer |
| Gancao | MOL000354 | isorhamnetin | NCOA2 | gastric cancer |
| Gancao | MOL000359 | sitosterol | NCOA2 | gastric cancer |
| Gancao | MOL000417 | Calycosin | NCOA2 | gastric cancer |
| Gancao | MOL000422 | kaempferol | NCOA2 | gastric cancer |
| Gancao | MOL000497 | licochalcone a | NCOA2 | gastric cancer |
| Gancao | MOL003656 | Lupiwighteone | NCOA2 | gastric cancer |
| Gancao | MOL003896 | 7-Methoxy-2-methyl isoflavone | NCOA2 | gastric cancer |
| Gancao | MOL004808 | glyasperin B | NCOA2 | gastric cancer |
| Gancao | MOL004811 | Glyasperin C | NCOA2 | gastric cancer |
| Gancao | MOL004815 | (E)-1-(2,4-dihydroxyphenyl)-3-(2,2-dimethylchromen-6-yl)prop-2-en-1-one | NCOA2 | gastric cancer |
| Gancao | MOL004820 | kanzonols W | NCOA2 | gastric cancer |
| Gancao | MOL004848 | licochalcone G | NCOA2 | gastric cancer |
| Gancao | MOL004849 | 3-(2,4-dihydroxyphenyl)-8-(1,1-dimethylprop-2-enyl)-7-hydroxy-5-methoxy-coumarin | NCOA2 | gastric cancer |
| Gancao | MOL004855 | Licoricone | NCOA2 | gastric cancer |
| Gancao | MOL004856 | Gancaonin A | NCOA2 | gastric cancer |
| Gancao | MOL004857 | Gancaonin B | NCOA2 | gastric cancer |
| Gancao | MOL004863 | 3-(3,4-dihydroxyphenyl)-5,7-dihydroxy-8-(3-methylbut-2-enyl)chromone | NCOA2 | gastric cancer |
| Gancao | MOL004866 | 2-(3,4-dihydroxyphenyl)-5,7-dihydroxy-6-(3-methylbut-2-enyl)chromone | NCOA2 | gastric cancer |
| Gancao | MOL004879 | Glycyrin | NCOA2 | gastric cancer |
| Gancao | MOL004883 | Licoisoflavone | NCOA2 | gastric cancer |
| Gancao | MOL004898 | (E)-3-[3,4-dihydroxy-5-(3-methylbut-2-enyl)phenyl]-1-(2,4-dihydroxyphenyl)prop-2-en-1-one | NCOA2 | gastric cancer |
| Gancao | MOL004908 | Glabridin | NCOA2 | gastric cancer |
| Gancao | MOL004911 | Glabrene | NCOA2 | gastric cancer |
| Gancao | MOL004949 | Isolicoflavonol | NCOA2 | gastric cancer |
| Gancao | MOL004959 | 1-Methoxyphaseollidin | NCOA2 | gastric cancer |
| Gancao | MOL004961 | Quercetin der. | NCOA2 | gastric cancer |
| Gancao | MOL004966 | 3'-Hydroxy-4'-O-Methylglabridin | NCOA2 | gastric cancer |
| Gancao | MOL004974 | 3'-Methoxyglabridin | NCOA2 | gastric cancer |
| Gancao | MOL004978 | 2-[(3R)-8,8-dimethyl-3,4-dihydro-2H-pyrano[6,5-f]chromen-3-yl]-5-methoxyphenol | NCOA2 | gastric cancer |
| Gancao | MOL004980 | Inflacoumarin A | NCOA2 | gastric cancer |
| Gancao | MOL004985 | icos-5-enoic acid | NCOA2 | gastric cancer |
| Gancao | MOL004988 | Kanzonol F | NCOA2 | gastric cancer |
| Gancao | MOL004991 | 7-Acetoxy-2-methylisoflavone | NCOA2 | gastric cancer |
| Gancao | MOL004996 | gadelaidic acid | NCOA2 | gastric cancer |
| Gancao | MOL005000 | Gancaonin G | NCOA2 | gastric cancer |
| Gancao | MOL005001 | Gancaonin H | NCOA2 | gastric cancer |
| Gancao | MOL005003 | Licoagrocarpin | NCOA2 | gastric cancer |
| Gancao | MOL005007 | Glyasperins M | NCOA2 | gastric cancer |
| Gancao | MOL005016 | Odoratin | NCOA2 | gastric cancer |
| Gancao | MOL005018 | Xambioona | NCOA2 | gastric cancer |
| Gancao | MOL005020 | dehydroglyasperins C | NCOA2 | gastric cancer |
| Gancao | MOL000239 | Jaranol | NOS2 | gastric cancer |
| Gancao | MOL000354 | isorhamnetin | NOS2 | gastric cancer |
| Gancao | MOL000392 | formononetin | NOS2 | gastric cancer |
| Gancao | MOL000417 | Calycosin | NOS2 | gastric cancer |
| Gancao | MOL000422 | kaempferol | NOS2 | gastric cancer |
| Gancao | MOL000497 | licochalcone a | NOS2 | gastric cancer |
| Gancao | MOL000500 | Vestitol | NOS2 | gastric cancer |
| Gancao | MOL002311 | Glycyrol | NOS2 | gastric cancer |
| Gancao | MOL002565 | Medicarpin | NOS2 | gastric cancer |
| Gancao | MOL003656 | Lupiwighteone | NOS2 | gastric cancer |
| Gancao | MOL003896 | 7-Methoxy-2-methyl isoflavone | NOS2 | gastric cancer |
| Gancao | MOL004805 | (2S)-2-[4-hydroxy-3-(3-methylbut-2-enyl)phenyl]-8,8-dimethyl-2,3-dihydropyrano[2,3-f]chromen-4-one | NOS2 | gastric cancer |
| Gancao | MOL004806 | euchrenone | NOS2 | gastric cancer |
| Gancao | MOL004808 | glyasperin B | NOS2 | gastric cancer |
| Gancao | MOL004810 | glyasperin F | NOS2 | gastric cancer |
| Gancao | MOL004811 | Glyasperin C | NOS2 | gastric cancer |
| Gancao | MOL004814 | Isotrifoliol | NOS2 | gastric cancer |
| Gancao | MOL004815 | (E)-1-(2,4-dihydroxyphenyl)-3-(2,2-dimethylchromen-6-yl)prop-2-en-1-one | NOS2 | gastric cancer |
| Gancao | MOL004820 | kanzonols W | NOS2 | gastric cancer |
| Gancao | MOL004824 | (2S)-6-(2,4-dihydroxyphenyl)-2-(2-hydroxypropan-2-yl)-4-methoxy-2,3-dihydrofuro[3,2-g]chromen-7-one | NOS2 | gastric cancer |
| Gancao | MOL004827 | Semilicoisoflavone B | NOS2 | gastric cancer |
| Gancao | MOL004828 | Glepidotin A | NOS2 | gastric cancer |
| Gancao | MOL004833 | Phaseolinisoflavan | NOS2 | gastric cancer |
| Gancao | MOL004835 | Glypallichalcone | NOS2 | gastric cancer |
| Gancao | MOL004838 | 8-(6-hydroxy-2-benzofuranyl)-2,2-dimethyl-5-chromenol | NOS2 | gastric cancer |
| Gancao | MOL004841 | Licochalcone B | NOS2 | gastric cancer |
| Gancao | MOL004848 | licochalcone G | NOS2 | gastric cancer |
| Gancao | MOL004849 | 3-(2,4-dihydroxyphenyl)-8-(1,1-dimethylprop-2-enyl)-7-hydroxy-5-methoxy-coumarin | NOS2 | gastric cancer |
| Gancao | MOL004855 | Licoricone | NOS2 | gastric cancer |
| Gancao | MOL004856 | Gancaonin A | NOS2 | gastric cancer |
| Gancao | MOL004857 | Gancaonin B | NOS2 | gastric cancer |
| Gancao | MOL004863 | 3-(3,4-dihydroxyphenyl)-5,7-dihydroxy-8-(3-methylbut-2-enyl)chromone | NOS2 | gastric cancer |
| Gancao | MOL004864 | 5,7-dihydroxy-3-(4-methoxyphenyl)-8-(3-methylbut-2-enyl)chromone | NOS2 | gastric cancer |
| Gancao | MOL004879 | Glycyrin | NOS2 | gastric cancer |
| Gancao | MOL004883 | Licoisoflavone | NOS2 | gastric cancer |
| Gancao | MOL004884 | Licoisoflavone B | NOS2 | gastric cancer |
| Gancao | MOL004885 | licoisoflavanone | NOS2 | gastric cancer |
| Gancao | MOL004891 | shinpterocarpin | NOS2 | gastric cancer |
| Gancao | MOL004904 | licopyranocoumarin | NOS2 | gastric cancer |
| Gancao | MOL004907 | Glyzaglabrin | NOS2 | gastric cancer |
| Gancao | MOL004908 | Glabridin | NOS2 | gastric cancer |
| Gancao | MOL004910 | Glabranin | NOS2 | gastric cancer |
| Gancao | MOL004911 | Glabrene | NOS2 | gastric cancer |
| Gancao | MOL004912 | Glabrone | NOS2 | gastric cancer |
| Gancao | MOL004915 | Eurycarpin A | NOS2 | gastric cancer |
| Gancao | MOL004948 | Isoglycyrol | NOS2 | gastric cancer |
| Gancao | MOL004949 | Isolicoflavonol | NOS2 | gastric cancer |
| Gancao | MOL004957 | HMO | NOS2 | gastric cancer |
| Gancao | MOL004959 | 1-Methoxyphaseollidin | NOS2 | gastric cancer |
| Gancao | MOL004961 | Quercetin der. | NOS2 | gastric cancer |
| Gancao | MOL004966 | 3'-Hydroxy-4'-O-Methylglabridin | NOS2 | gastric cancer |
| Gancao | MOL004974 | 3'-Methoxyglabridin | NOS2 | gastric cancer |
| Gancao | MOL004978 | 2-[(3R)-8,8-dimethyl-3,4-dihydro-2H-pyrano[6,5-f]chromen-3-yl]-5-methoxyphenol | NOS2 | gastric cancer |
| Gancao | MOL004989 | 6-prenylated eriodictyol | NOS2 | gastric cancer |
| Gancao | MOL004990 | 7,2',4'-trihydroxy－5-methoxy-3－arylcoumarin | NOS2 | gastric cancer |
| Gancao | MOL004991 | 7-Acetoxy-2-methylisoflavone | NOS2 | gastric cancer |
| Gancao | MOL005000 | Gancaonin G | NOS2 | gastric cancer |
| Gancao | MOL005003 | Licoagrocarpin | NOS2 | gastric cancer |
| Gancao | MOL005007 | Glyasperins M | NOS2 | gastric cancer |
| Gancao | MOL005008 | Glycyrrhiza flavonol A | NOS2 | gastric cancer |
| Gancao | MOL005012 | Licoagroisoflavone | NOS2 | gastric cancer |
| Gancao | MOL005016 | Odoratin | NOS2 | gastric cancer |
| Gancao | MOL005018 | Xambioona | NOS2 | gastric cancer |
| Gancao | MOL005020 | dehydroglyasperins C | NOS2 | gastric cancer |
| Gancao | MOL000098 | quercetin | NOS3 | gastric cancer |
| Gancao | MOL000354 | isorhamnetin | NOS3 | gastric cancer |
| Gancao | MOL000392 | formononetin | NOS3 | gastric cancer |
| Gancao | MOL000422 | kaempferol | NOS3 | gastric cancer |
| Gancao | MOL003896 | 7-Methoxy-2-methyl isoflavone | NOS3 | gastric cancer |
| Gancao | MOL004828 | Glepidotin A | NOS3 | gastric cancer |
| Gancao | MOL004829 | Glepidotin B | NOS3 | gastric cancer |
| Gancao | MOL004910 | Glabranin | NOS3 | gastric cancer |
| Gancao | MOL004959 | 1-Methoxyphaseollidin | NOS3 | gastric cancer |
| Gancao | MOL004978 | 2-[(3R)-8,8-dimethyl-3,4-dihydro-2H-pyrano[6,5-f]chromen-3-yl]-5-methoxyphenol | NOS3 | gastric cancer |
| Gancao | MOL004991 | 7-Acetoxy-2-methylisoflavone | NOS3 | gastric cancer |
| Gancao | MOL005000 | Gancaonin G | NOS3 | gastric cancer |
| Gancao | MOL005003 | Licoagrocarpin | NOS3 | gastric cancer |
| Gancao | MOL000098 | quercetin | NQO1 | gastric cancer |
| Gancao | MOL000359 | sitosterol | NR3C2 | gastric cancer |
| Gancao | MOL000098 | quercetin | ODC1 | gastric cancer |
| Gancao | MOL002565 | Medicarpin | OPRD1 | gastric cancer |
| Gancao | MOL004891 | shinpterocarpin | OPRD1 | gastric cancer |
| Gancao | MOL001484 | Inermine | OPRM1 | gastric cancer |
| Gancao | MOL002565 | Medicarpin | OPRM1 | gastric cancer |
| Gancao | MOL003896 | 7-Methoxy-2-methyl isoflavone | OPRM1 | gastric cancer |
| Gancao | MOL004891 | shinpterocarpin | OPRM1 | gastric cancer |
| Gancao | MOL000392 | formononetin | PDE3A | gastric cancer |
| Gancao | MOL000417 | Calycosin | PDE3A | gastric cancer |
| Gancao | MOL000500 | Vestitol | PDE3A | gastric cancer |
| Gancao | MOL002565 | Medicarpin | PDE3A | gastric cancer |
| Gancao | MOL002844 | Pinocembrin | PDE3A | gastric cancer |
| Gancao | MOL003896 | 7-Methoxy-2-methyl isoflavone | PDE3A | gastric cancer |
| Gancao | MOL004828 | Glepidotin A | PDE3A | gastric cancer |
| Gancao | MOL004829 | Glepidotin B | PDE3A | gastric cancer |
| Gancao | MOL004835 | Glypallichalcone | PDE3A | gastric cancer |
| Gancao | MOL004841 | Licochalcone B | PDE3A | gastric cancer |
| Gancao | MOL004910 | Glabranin | PDE3A | gastric cancer |
| Gancao | MOL004941 | (2R)-7-hydroxy-2-(4-hydroxyphenyl)chroman-4-one | PDE3A | gastric cancer |
| Gancao | MOL004945 | (2S)-7-hydroxy-2-(4-hydroxyphenyl)-8-(3-methylbut-2-enyl)chroman-4-one | PDE3A | gastric cancer |
| Gancao | MOL004957 | HMO | PDE3A | gastric cancer |
| Gancao | MOL004991 | 7-Acetoxy-2-methylisoflavone | PDE3A | gastric cancer |
| Gancao | MOL000211 | Mairin | PGR | gastric cancer |
| Gancao | MOL000359 | sitosterol | PGR | gastric cancer |
| Gancao | MOL000422 | kaempferol | PGR | gastric cancer |
| Gancao | MOL000354 | isorhamnetin | PIK3CG | gastric cancer |
| Gancao | MOL000422 | kaempferol | PIK3CG | gastric cancer |
| Gancao | MOL001484 | Inermine | PIK3CG | gastric cancer |
| Gancao | MOL001792 | DFV | PIK3CG | gastric cancer |
| Gancao | MOL002565 | Medicarpin | PIK3CG | gastric cancer |
| Gancao | MOL002844 | Pinocembrin | PIK3CG | gastric cancer |
| Gancao | MOL004328 | naringenin | PIK3CG | gastric cancer |
| Gancao | MOL004814 | Isotrifoliol | PIK3CG | gastric cancer |
| Gancao | MOL004838 | 8-(6-hydroxy-2-benzofuranyl)-2,2-dimethyl-5-chromenol | PIK3CG | gastric cancer |
| Gancao | MOL004891 | shinpterocarpin | PIK3CG | gastric cancer |
| Gancao | MOL004907 | Glyzaglabrin | PIK3CG | gastric cancer |
| Gancao | MOL004941 | (2R)-7-hydroxy-2-(4-hydroxyphenyl)chroman-4-one | PIK3CG | gastric cancer |
| Gancao | MOL004959 | 1-Methoxyphaseollidin | PIK3CG | gastric cancer |
| Gancao | MOL000354 | isorhamnetin | PIM1 | gastric cancer |
| Gancao | MOL000392 | formononetin | PIM1 | gastric cancer |
| Gancao | MOL000417 | Calycosin | PIM1 | gastric cancer |
| Gancao | MOL000497 | licochalcone a | PIM1 | gastric cancer |
| Gancao | MOL000500 | Vestitol | PIM1 | gastric cancer |
| Gancao | MOL002311 | Glycyrol | PIM1 | gastric cancer |
| Gancao | MOL002565 | Medicarpin | PIM1 | gastric cancer |
| Gancao | MOL003656 | Lupiwighteone | PIM1 | gastric cancer |
| Gancao | MOL003896 | 7-Methoxy-2-methyl isoflavone | PIM1 | gastric cancer |
| Gancao | MOL004805 | (2S)-2-[4-hydroxy-3-(3-methylbut-2-enyl)phenyl]-8,8-dimethyl-2,3-dihydropyrano[2,3-f]chromen-4-one | PIM1 | gastric cancer |
| Gancao | MOL004806 | euchrenone | PIM1 | gastric cancer |
| Gancao | MOL004808 | glyasperin B | PIM1 | gastric cancer |
| Gancao | MOL004810 | glyasperin F | PIM1 | gastric cancer |
| Gancao | MOL004811 | Glyasperin C | PIM1 | gastric cancer |
| Gancao | MOL004814 | Isotrifoliol | PIM1 | gastric cancer |
| Gancao | MOL004815 | (E)-1-(2,4-dihydroxyphenyl)-3-(2,2-dimethylchromen-6-yl)prop-2-en-1-one | PIM1 | gastric cancer |
| Gancao | MOL004820 | kanzonols W | PIM1 | gastric cancer |
| Gancao | MOL004824 | (2S)-6-(2,4-dihydroxyphenyl)-2-(2-hydroxypropan-2-yl)-4-methoxy-2,3-dihydrofuro[3,2-g]chromen-7-one | PIM1 | gastric cancer |
| Gancao | MOL004828 | Glepidotin A | PIM1 | gastric cancer |
| Gancao | MOL004833 | Phaseolinisoflavan | PIM1 | gastric cancer |
| Gancao | MOL004841 | Licochalcone B | PIM1 | gastric cancer |
| Gancao | MOL004848 | licochalcone G | PIM1 | gastric cancer |
| Gancao | MOL004849 | 3-(2,4-dihydroxyphenyl)-8-(1,1-dimethylprop-2-enyl)-7-hydroxy-5-methoxy-coumarin | PIM1 | gastric cancer |
| Gancao | MOL004855 | Licoricone | PIM1 | gastric cancer |
| Gancao | MOL004856 | Gancaonin A | PIM1 | gastric cancer |
| Gancao | MOL004857 | Gancaonin B | PIM1 | gastric cancer |
| Gancao | MOL004863 | 3-(3,4-dihydroxyphenyl)-5,7-dihydroxy-8-(3-methylbut-2-enyl)chromone | PIM1 | gastric cancer |
| Gancao | MOL004866 | 2-(3,4-dihydroxyphenyl)-5,7-dihydroxy-6-(3-methylbut-2-enyl)chromone | PIM1 | gastric cancer |
| Gancao | MOL004879 | Glycyrin | PIM1 | gastric cancer |
| Gancao | MOL004883 | Licoisoflavone | PIM1 | gastric cancer |
| Gancao | MOL004884 | Licoisoflavone B | PIM1 | gastric cancer |
| Gancao | MOL004885 | licoisoflavanone | PIM1 | gastric cancer |
| Gancao | MOL004891 | shinpterocarpin | PIM1 | gastric cancer |
| Gancao | MOL004898 | (E)-3-[3,4-dihydroxy-5-(3-methylbut-2-enyl)phenyl]-1-(2,4-dihydroxyphenyl)prop-2-en-1-one | PIM1 | gastric cancer |
| Gancao | MOL004904 | licopyranocoumarin | PIM1 | gastric cancer |
| Gancao | MOL004907 | Glyzaglabrin | PIM1 | gastric cancer |
| Gancao | MOL004908 | Glabridin | PIM1 | gastric cancer |
| Gancao | MOL004911 | Glabrene | PIM1 | gastric cancer |
| Gancao | MOL004912 | Glabrone | PIM1 | gastric cancer |
| Gancao | MOL004915 | Eurycarpin A | PIM1 | gastric cancer |
| Gancao | MOL004948 | Isoglycyrol | PIM1 | gastric cancer |
| Gancao | MOL004949 | Isolicoflavonol | PIM1 | gastric cancer |
| Gancao | MOL004957 | HMO | PIM1 | gastric cancer |
| Gancao | MOL004959 | 1-Methoxyphaseollidin | PIM1 | gastric cancer |
| Gancao | MOL004966 | 3'-Hydroxy-4'-O-Methylglabridin | PIM1 | gastric cancer |
| Gancao | MOL004974 | 3'-Methoxyglabridin | PIM1 | gastric cancer |
| Gancao | MOL004978 | 2-[(3R)-8,8-dimethyl-3,4-dihydro-2H-pyrano[6,5-f]chromen-3-yl]-5-methoxyphenol | PIM1 | gastric cancer |
| Gancao | MOL004980 | Inflacoumarin A | PIM1 | gastric cancer |
| Gancao | MOL004988 | Kanzonol F | PIM1 | gastric cancer |
| Gancao | MOL004990 | 7,2',4'-trihydroxy－5-methoxy-3－arylcoumarin | PIM1 | gastric cancer |
| Gancao | MOL005000 | Gancaonin G | PIM1 | gastric cancer |
| Gancao | MOL005001 | Gancaonin H | PIM1 | gastric cancer |
| Gancao | MOL005003 | Licoagrocarpin | PIM1 | gastric cancer |
| Gancao | MOL005007 | Glyasperins M | PIM1 | gastric cancer |
| Gancao | MOL005008 | Glycyrrhiza flavonol A | PIM1 | gastric cancer |
| Gancao | MOL005012 | Licoagroisoflavone | PIM1 | gastric cancer |
| Gancao | MOL005016 | Odoratin | PIM1 | gastric cancer |
| Gancao | MOL005017 | Phaseol | PIM1 | gastric cancer |
| Gancao | MOL005018 | Xambioona | PIM1 | gastric cancer |
| Gancao | MOL005020 | dehydroglyasperins C | PIM1 | gastric cancer |
| Gancao | MOL000098 | quercetin | PLAT | gastric cancer |
| Gancao | MOL000098 | quercetin | PLAU | gastric cancer |
| Gancao | MOL000098 | quercetin | PON1 | gastric cancer |
| Gancao | MOL000098 | quercetin | POR | gastric cancer |
| Gancao | MOL000354 | isorhamnetin | PPARD | gastric cancer |
| Gancao | MOL000392 | formononetin | PPARD | gastric cancer |
| Gancao | MOL000417 | Calycosin | PPARD | gastric cancer |
| Gancao | MOL000422 | kaempferol | PPARD | gastric cancer |
| Gancao | MOL000497 | licochalcone a | PPARD | gastric cancer |
| Gancao | MOL000500 | Vestitol | PPARD | gastric cancer |
| Gancao | MOL002311 | Glycyrol | PPARD | gastric cancer |
| Gancao | MOL003656 | Lupiwighteone | PPARD | gastric cancer |
| Gancao | MOL003896 | 7-Methoxy-2-methyl isoflavone | PPARD | gastric cancer |
| Gancao | MOL005007 | Glyasperins M | PPARD | gastric cancer |
| Gancao | MOL000098 | quercetin | PPARG | gastric cancer |
| Gancao | MOL000354 | isorhamnetin | PPARG | gastric cancer |
| Gancao | MOL004805 | (2S)-2-[4-hydroxy-3-(3-methylbut-2-enyl)phenyl]-8,8-dimethyl-2,3-dihydropyrano[2,3-f]chromen-4-one | PPARG | gastric cancer |
| Gancao | MOL004808 | glyasperin B | PPARG | gastric cancer |
| Gancao | MOL004810 | glyasperin F | PPARG | gastric cancer |
| Gancao | MOL004811 | Glyasperin C | PPARG | gastric cancer |
| Gancao | MOL004815 | (E)-1-(2,4-dihydroxyphenyl)-3-(2,2-dimethylchromen-6-yl)prop-2-en-1-one | PPARG | gastric cancer |
| Gancao | MOL004820 | kanzonols W | PPARG | gastric cancer |
| Gancao | MOL004824 | (2S)-6-(2,4-dihydroxyphenyl)-2-(2-hydroxypropan-2-yl)-4-methoxy-2,3-dihydrofuro[3,2-g]chromen-7-one | PPARG | gastric cancer |
| Gancao | MOL004827 | Semilicoisoflavone B | PPARG | gastric cancer |
| Gancao | MOL004828 | Glepidotin A | PPARG | gastric cancer |
| Gancao | MOL004833 | Phaseolinisoflavan | PPARG | gastric cancer |
| Gancao | MOL004835 | Glypallichalcone | PPARG | gastric cancer |
| Gancao | MOL004841 | Licochalcone B | PPARG | gastric cancer |
| Gancao | MOL004848 | licochalcone G | PPARG | gastric cancer |
| Gancao | MOL004849 | 3-(2,4-dihydroxyphenyl)-8-(1,1-dimethylprop-2-enyl)-7-hydroxy-5-methoxy-coumarin | PPARG | gastric cancer |
| Gancao | MOL004855 | Licoricone | PPARG | gastric cancer |
| Gancao | MOL004856 | Gancaonin A | PPARG | gastric cancer |
| Gancao | MOL004857 | Gancaonin B | PPARG | gastric cancer |
| Gancao | MOL004863 | 3-(3,4-dihydroxyphenyl)-5,7-dihydroxy-8-(3-methylbut-2-enyl)chromone | PPARG | gastric cancer |
| Gancao | MOL004866 | 2-(3,4-dihydroxyphenyl)-5,7-dihydroxy-6-(3-methylbut-2-enyl)chromone | PPARG | gastric cancer |
| Gancao | MOL004879 | Glycyrin | PPARG | gastric cancer |
| Gancao | MOL004883 | Licoisoflavone | PPARG | gastric cancer |
| Gancao | MOL004884 | Licoisoflavone B | PPARG | gastric cancer |
| Gancao | MOL004885 | licoisoflavanone | PPARG | gastric cancer |
| Gancao | MOL004891 | shinpterocarpin | PPARG | gastric cancer |
| Gancao | MOL004898 | (E)-3-[3,4-dihydroxy-5-(3-methylbut-2-enyl)phenyl]-1-(2,4-dihydroxyphenyl)prop-2-en-1-one | PPARG | gastric cancer |
| Gancao | MOL004904 | licopyranocoumarin | PPARG | gastric cancer |
| Gancao | MOL004907 | Glyzaglabrin | PPARG | gastric cancer |
| Gancao | MOL004908 | Glabridin | PPARG | gastric cancer |
| Gancao | MOL004911 | Glabrene | PPARG | gastric cancer |
| Gancao | MOL004912 | Glabrone | PPARG | gastric cancer |
| Gancao | MOL004913 | 1,3-dihydroxy-9-methoxy-6-benzofurano[3,2-c]chromenone | PPARG | gastric cancer |
| Gancao | MOL004914 | 1,3-dihydroxy-8,9-dimethoxy-6-benzofurano[3,2-c]chromenone | PPARG | gastric cancer |
| Gancao | MOL004915 | Eurycarpin A | PPARG | gastric cancer |
| Gancao | MOL004949 | Isolicoflavonol | PPARG | gastric cancer |
| Gancao | MOL004957 | HMO | PPARG | gastric cancer |
| Gancao | MOL004959 | 1-Methoxyphaseollidin | PPARG | gastric cancer |
| Gancao | MOL004961 | Quercetin der. | PPARG | gastric cancer |
| Gancao | MOL004966 | 3'-Hydroxy-4'-O-Methylglabridin | PPARG | gastric cancer |
| Gancao | MOL004974 | 3'-Methoxyglabridin | PPARG | gastric cancer |
| Gancao | MOL004978 | 2-[(3R)-8,8-dimethyl-3,4-dihydro-2H-pyrano[6,5-f]chromen-3-yl]-5-methoxyphenol | PPARG | gastric cancer |
| Gancao | MOL004980 | Inflacoumarin A | PPARG | gastric cancer |
| Gancao | MOL004990 | 7,2',4'-trihydroxy－5-methoxy-3－arylcoumarin | PPARG | gastric cancer |
| Gancao | MOL004991 | 7-Acetoxy-2-methylisoflavone | PPARG | gastric cancer |
| Gancao | MOL005000 | Gancaonin G | PPARG | gastric cancer |
| Gancao | MOL005003 | Licoagrocarpin | PPARG | gastric cancer |
| Gancao | MOL005007 | Glyasperins M | PPARG | gastric cancer |
| Gancao | MOL005012 | Licoagroisoflavone | PPARG | gastric cancer |
| Gancao | MOL005016 | Odoratin | PPARG | gastric cancer |
| Gancao | MOL005017 | Phaseol | PPARG | gastric cancer |
| Gancao | MOL005020 | dehydroglyasperins C | PPARG | gastric cancer |
| Gancao | MOL000422 | kaempferol | PPP3CA | gastric cancer |
| Gancao | MOL000098 | quercetin | PRKACA | gastric cancer |
| Gancao | MOL000354 | isorhamnetin | PRKACA | gastric cancer |
| Gancao | MOL000392 | formononetin | PRKACA | gastric cancer |
| Gancao | MOL000417 | Calycosin | PRKACA | gastric cancer |
| Gancao | MOL000422 | kaempferol | PRKACA | gastric cancer |
| Gancao | MOL000500 | Vestitol | PRKACA | gastric cancer |
| Gancao | MOL001484 | Inermine | PRKACA | gastric cancer |
| Gancao | MOL001792 | DFV | PRKACA | gastric cancer |
| Gancao | MOL002565 | Medicarpin | PRKACA | gastric cancer |
| Gancao | MOL002844 | Pinocembrin | PRKACA | gastric cancer |
| Gancao | MOL003896 | 7-Methoxy-2-methyl isoflavone | PRKACA | gastric cancer |
| Gancao | MOL004328 | naringenin | PRKACA | gastric cancer |
| Gancao | MOL004814 | Isotrifoliol | PRKACA | gastric cancer |
| Gancao | MOL004835 | Glypallichalcone | PRKACA | gastric cancer |
| Gancao | MOL004841 | Licochalcone B | PRKACA | gastric cancer |
| Gancao | MOL004891 | shinpterocarpin | PRKACA | gastric cancer |
| Gancao | MOL004907 | Glyzaglabrin | PRKACA | gastric cancer |
| Gancao | MOL004908 | Glabridin | PRKACA | gastric cancer |
| Gancao | MOL004910 | Glabranin | PRKACA | gastric cancer |
| Gancao | MOL004913 | 1,3-dihydroxy-9-methoxy-6-benzofurano[3,2-c]chromenone | PRKACA | gastric cancer |
| Gancao | MOL004914 | 1,3-dihydroxy-8,9-dimethoxy-6-benzofurano[3,2-c]chromenone | PRKACA | gastric cancer |
| Gancao | MOL004941 | (2R)-7-hydroxy-2-(4-hydroxyphenyl)chroman-4-one | PRKACA | gastric cancer |
| Gancao | MOL004957 | HMO | PRKACA | gastric cancer |
| Gancao | MOL004966 | 3'-Hydroxy-4'-O-Methylglabridin | PRKACA | gastric cancer |
| Gancao | MOL004978 | 2-[(3R)-8,8-dimethyl-3,4-dihydro-2H-pyrano[6,5-f]chromen-3-yl]-5-methoxyphenol | PRKACA | gastric cancer |
| Gancao | MOL004990 | 7,2',4'-trihydroxy－5-methoxy-3－arylcoumarin | PRKACA | gastric cancer |
| Gancao | MOL005007 | Glyasperins M | PRKACA | gastric cancer |
| Gancao | MOL005017 | Phaseol | PRKACA | gastric cancer |
| Gancao | MOL000098 | quercetin | PRSS1 | gastric cancer |
| Gancao | MOL000239 | Jaranol | PRSS1 | gastric cancer |
| Gancao | MOL000354 | isorhamnetin | PRSS1 | gastric cancer |
| Gancao | MOL000392 | formononetin | PRSS1 | gastric cancer |
| Gancao | MOL000417 | Calycosin | PRSS1 | gastric cancer |
| Gancao | MOL000422 | kaempferol | PRSS1 | gastric cancer |
| Gancao | MOL000500 | Vestitol | PRSS1 | gastric cancer |
| Gancao | MOL001484 | Inermine | PRSS1 | gastric cancer |
| Gancao | MOL002565 | Medicarpin | PRSS1 | gastric cancer |
| Gancao | MOL003656 | Lupiwighteone | PRSS1 | gastric cancer |
| Gancao | MOL003896 | 7-Methoxy-2-methyl isoflavone | PRSS1 | gastric cancer |
| Gancao | MOL004808 | glyasperin B | PRSS1 | gastric cancer |
| Gancao | MOL004810 | glyasperin F | PRSS1 | gastric cancer |
| Gancao | MOL004811 | Glyasperin C | PRSS1 | gastric cancer |
| Gancao | MOL004820 | kanzonols W | PRSS1 | gastric cancer |
| Gancao | MOL004824 | (2S)-6-(2,4-dihydroxyphenyl)-2-(2-hydroxypropan-2-yl)-4-methoxy-2,3-dihydrofuro[3,2-g]chromen-7-one | PRSS1 | gastric cancer |
| Gancao | MOL004827 | Semilicoisoflavone B | PRSS1 | gastric cancer |
| Gancao | MOL004828 | Glepidotin A | PRSS1 | gastric cancer |
| Gancao | MOL004833 | Phaseolinisoflavan | PRSS1 | gastric cancer |
| Gancao | MOL004849 | 3-(2,4-dihydroxyphenyl)-8-(1,1-dimethylprop-2-enyl)-7-hydroxy-5-methoxy-coumarin | PRSS1 | gastric cancer |
| Gancao | MOL004855 | Licoricone | PRSS1 | gastric cancer |
| Gancao | MOL004856 | Gancaonin A | PRSS1 | gastric cancer |
| Gancao | MOL004857 | Gancaonin B | PRSS1 | gastric cancer |
| Gancao | MOL004863 | 3-(3,4-dihydroxyphenyl)-5,7-dihydroxy-8-(3-methylbut-2-enyl)chromone | PRSS1 | gastric cancer |
| Gancao | MOL004866 | 2-(3,4-dihydroxyphenyl)-5,7-dihydroxy-6-(3-methylbut-2-enyl)chromone | PRSS1 | gastric cancer |
| Gancao | MOL004879 | Glycyrin | PRSS1 | gastric cancer |
| Gancao | MOL004883 | Licoisoflavone | PRSS1 | gastric cancer |
| Gancao | MOL004884 | Licoisoflavone B | PRSS1 | gastric cancer |
| Gancao | MOL004885 | licoisoflavanone | PRSS1 | gastric cancer |
| Gancao | MOL004891 | shinpterocarpin | PRSS1 | gastric cancer |
| Gancao | MOL004904 | licopyranocoumarin | PRSS1 | gastric cancer |
| Gancao | MOL004907 | Glyzaglabrin | PRSS1 | gastric cancer |
| Gancao | MOL004908 | Glabridin | PRSS1 | gastric cancer |
| Gancao | MOL004911 | Glabrene | PRSS1 | gastric cancer |
| Gancao | MOL004912 | Glabrone | PRSS1 | gastric cancer |
| Gancao | MOL004915 | Eurycarpin A | PRSS1 | gastric cancer |
| Gancao | MOL004949 | Isolicoflavonol | PRSS1 | gastric cancer |
| Gancao | MOL004957 | HMO | PRSS1 | gastric cancer |
| Gancao | MOL004959 | 1-Methoxyphaseollidin | PRSS1 | gastric cancer |
| Gancao | MOL004961 | Quercetin der. | PRSS1 | gastric cancer |
| Gancao | MOL004966 | 3'-Hydroxy-4'-O-Methylglabridin | PRSS1 | gastric cancer |
| Gancao | MOL004974 | 3'-Methoxyglabridin | PRSS1 | gastric cancer |
| Gancao | MOL004978 | 2-[(3R)-8,8-dimethyl-3,4-dihydro-2H-pyrano[6,5-f]chromen-3-yl]-5-methoxyphenol | PRSS1 | gastric cancer |
| Gancao | MOL004980 | Inflacoumarin A | PRSS1 | gastric cancer |
| Gancao | MOL004991 | 7-Acetoxy-2-methylisoflavone | PRSS1 | gastric cancer |
| Gancao | MOL005000 | Gancaonin G | PRSS1 | gastric cancer |
| Gancao | MOL005001 | Gancaonin H | PRSS1 | gastric cancer |
| Gancao | MOL005003 | Licoagrocarpin | PRSS1 | gastric cancer |
| Gancao | MOL005007 | Glyasperins M | PRSS1 | gastric cancer |
| Gancao | MOL005008 | Glycyrrhiza flavonol A | PRSS1 | gastric cancer |
| Gancao | MOL005012 | Licoagroisoflavone | PRSS1 | gastric cancer |
| Gancao | MOL005016 | Odoratin | PRSS1 | gastric cancer |
| Gancao | MOL005020 | dehydroglyasperins C | PRSS1 | gastric cancer |
| Gancao | MOL000105 | protocatechuic acid | PRSS3 | gastric cancer |
| Gancao | MOL000098 | quercetin | PTGER3 | gastric cancer |
| Gancao | MOL000098 | quercetin | PTGS1 | gastric cancer |
| Gancao | MOL000105 | protocatechuic acid | PTGS1 | gastric cancer |
| Gancao | MOL000239 | Jaranol | PTGS1 | gastric cancer |
| Gancao | MOL000354 | isorhamnetin | PTGS1 | gastric cancer |
| Gancao | MOL000392 | formononetin | PTGS1 | gastric cancer |
| Gancao | MOL000417 | Calycosin | PTGS1 | gastric cancer |
| Gancao | MOL000422 | kaempferol | PTGS1 | gastric cancer |
| Gancao | MOL000497 | licochalcone a | PTGS1 | gastric cancer |
| Gancao | MOL000500 | Vestitol | PTGS1 | gastric cancer |
| Gancao | MOL001484 | Inermine | PTGS1 | gastric cancer |
| Gancao | MOL001792 | DFV | PTGS1 | gastric cancer |
| Gancao | MOL002565 | Medicarpin | PTGS1 | gastric cancer |
| Gancao | MOL002844 | Pinocembrin | PTGS1 | gastric cancer |
| Gancao | MOL003896 | 7-Methoxy-2-methyl isoflavone | PTGS1 | gastric cancer |
| Gancao | MOL004328 | naringenin | PTGS1 | gastric cancer |
| Gancao | MOL004810 | glyasperin F | PTGS1 | gastric cancer |
| Gancao | MOL004815 | (E)-1-(2,4-dihydroxyphenyl)-3-(2,2-dimethylchromen-6-yl)prop-2-en-1-one | PTGS1 | gastric cancer |
| Gancao | MOL004820 | kanzonols W | PTGS1 | gastric cancer |
| Gancao | MOL004828 | Glepidotin A | PTGS1 | gastric cancer |
| Gancao | MOL004829 | Glepidotin B | PTGS1 | gastric cancer |
| Gancao | MOL004835 | Glypallichalcone | PTGS1 | gastric cancer |
| Gancao | MOL004841 | Licochalcone B | PTGS1 | gastric cancer |
| Gancao | MOL004885 | licoisoflavanone | PTGS1 | gastric cancer |
| Gancao | MOL004891 | shinpterocarpin | PTGS1 | gastric cancer |
| Gancao | MOL004907 | Glyzaglabrin | PTGS1 | gastric cancer |
| Gancao | MOL004910 | Glabranin | PTGS1 | gastric cancer |
| Gancao | MOL004911 | Glabrene | PTGS1 | gastric cancer |
| Gancao | MOL004912 | Glabrone | PTGS1 | gastric cancer |
| Gancao | MOL004941 | (2R)-7-hydroxy-2-(4-hydroxyphenyl)chroman-4-one | PTGS1 | gastric cancer |
| Gancao | MOL004945 | (2S)-7-hydroxy-2-(4-hydroxyphenyl)-8-(3-methylbut-2-enyl)chroman-4-one | PTGS1 | gastric cancer |
| Gancao | MOL004957 | HMO | PTGS1 | gastric cancer |
| Gancao | MOL004959 | 1-Methoxyphaseollidin | PTGS1 | gastric cancer |
| Gancao | MOL004961 | Quercetin der. | PTGS1 | gastric cancer |
| Gancao | MOL004966 | 3'-Hydroxy-4'-O-Methylglabridin | PTGS1 | gastric cancer |
| Gancao | MOL004974 | 3'-Methoxyglabridin | PTGS1 | gastric cancer |
| Gancao | MOL004978 | 2-[(3R)-8,8-dimethyl-3,4-dihydro-2H-pyrano[6,5-f]chromen-3-yl]-5-methoxyphenol | PTGS1 | gastric cancer |
| Gancao | MOL004980 | Inflacoumarin A | PTGS1 | gastric cancer |
| Gancao | MOL004990 | 7,2',4'-trihydroxy－5-methoxy-3－arylcoumarin | PTGS1 | gastric cancer |
| Gancao | MOL004991 | 7-Acetoxy-2-methylisoflavone | PTGS1 | gastric cancer |
| Gancao | MOL005003 | Licoagrocarpin | PTGS1 | gastric cancer |
| Gancao | MOL005007 | Glyasperins M | PTGS1 | gastric cancer |
| Gancao | MOL005016 | Odoratin | PTGS1 | gastric cancer |
| Gancao | MOL000098 | quercetin | PTGS2 | gastric cancer |
| Gancao | MOL000105 | protocatechuic acid | PTGS2 | gastric cancer |
| Gancao | MOL000239 | Jaranol | PTGS2 | gastric cancer |
| Gancao | MOL000354 | isorhamnetin | PTGS2 | gastric cancer |
| Gancao | MOL000392 | formononetin | PTGS2 | gastric cancer |
| Gancao | MOL000417 | Calycosin | PTGS2 | gastric cancer |
| Gancao | MOL000422 | kaempferol | PTGS2 | gastric cancer |
| Gancao | MOL000497 | licochalcone a | PTGS2 | gastric cancer |
| Gancao | MOL000500 | Vestitol | PTGS2 | gastric cancer |
| Gancao | MOL001484 | Inermine | PTGS2 | gastric cancer |
| Gancao | MOL001792 | DFV | PTGS2 | gastric cancer |
| Gancao | MOL002311 | Glycyrol | PTGS2 | gastric cancer |
| Gancao | MOL002565 | Medicarpin | PTGS2 | gastric cancer |
| Gancao | MOL002844 | Pinocembrin | PTGS2 | gastric cancer |
| Gancao | MOL003656 | Lupiwighteone | PTGS2 | gastric cancer |
| Gancao | MOL003896 | 7-Methoxy-2-methyl isoflavone | PTGS2 | gastric cancer |
| Gancao | MOL004328 | naringenin | PTGS2 | gastric cancer |
| Gancao | MOL004805 | (2S)-2-[4-hydroxy-3-(3-methylbut-2-enyl)phenyl]-8,8-dimethyl-2,3-dihydropyrano[2,3-f]chromen-4-one | PTGS2 | gastric cancer |
| Gancao | MOL004806 | euchrenone | PTGS2 | gastric cancer |
| Gancao | MOL004808 | glyasperin B | PTGS2 | gastric cancer |
| Gancao | MOL004810 | glyasperin F | PTGS2 | gastric cancer |
| Gancao | MOL004811 | Glyasperin C | PTGS2 | gastric cancer |
| Gancao | MOL004814 | Isotrifoliol | PTGS2 | gastric cancer |
| Gancao | MOL004815 | (E)-1-(2,4-dihydroxyphenyl)-3-(2,2-dimethylchromen-6-yl)prop-2-en-1-one | PTGS2 | gastric cancer |
| Gancao | MOL004820 | kanzonols W | PTGS2 | gastric cancer |
| Gancao | MOL004824 | (2S)-6-(2,4-dihydroxyphenyl)-2-(2-hydroxypropan-2-yl)-4-methoxy-2,3-dihydrofuro[3,2-g]chromen-7-one | PTGS2 | gastric cancer |
| Gancao | MOL004827 | Semilicoisoflavone B | PTGS2 | gastric cancer |
| Gancao | MOL004828 | Glepidotin A | PTGS2 | gastric cancer |
| Gancao | MOL004829 | Glepidotin B | PTGS2 | gastric cancer |
| Gancao | MOL004833 | Phaseolinisoflavan | PTGS2 | gastric cancer |
| Gancao | MOL004835 | Glypallichalcone | PTGS2 | gastric cancer |
| Gancao | MOL004838 | 8-(6-hydroxy-2-benzofuranyl)-2,2-dimethyl-5-chromenol | PTGS2 | gastric cancer |
| Gancao | MOL004841 | Licochalcone B | PTGS2 | gastric cancer |
| Gancao | MOL004848 | licochalcone G | PTGS2 | gastric cancer |
| Gancao | MOL004849 | 3-(2,4-dihydroxyphenyl)-8-(1,1-dimethylprop-2-enyl)-7-hydroxy-5-methoxy-coumarin | PTGS2 | gastric cancer |
| Gancao | MOL004855 | Licoricone | PTGS2 | gastric cancer |
| Gancao | MOL004856 | Gancaonin A | PTGS2 | gastric cancer |
| Gancao | MOL004857 | Gancaonin B | PTGS2 | gastric cancer |
| Gancao | MOL004863 | 3-(3,4-dihydroxyphenyl)-5,7-dihydroxy-8-(3-methylbut-2-enyl)chromone | PTGS2 | gastric cancer |
| Gancao | MOL004866 | 2-(3,4-dihydroxyphenyl)-5,7-dihydroxy-6-(3-methylbut-2-enyl)chromone | PTGS2 | gastric cancer |
| Gancao | MOL004879 | Glycyrin | PTGS2 | gastric cancer |
| Gancao | MOL004883 | Licoisoflavone | PTGS2 | gastric cancer |
| Gancao | MOL004884 | Licoisoflavone B | PTGS2 | gastric cancer |
| Gancao | MOL004885 | licoisoflavanone | PTGS2 | gastric cancer |
| Gancao | MOL004891 | shinpterocarpin | PTGS2 | gastric cancer |
| Gancao | MOL004898 | (E)-3-[3,4-dihydroxy-5-(3-methylbut-2-enyl)phenyl]-1-(2,4-dihydroxyphenyl)prop-2-en-1-one | PTGS2 | gastric cancer |
| Gancao | MOL004903 | liquiritin | PTGS2 | gastric cancer |
| Gancao | MOL004904 | licopyranocoumarin | PTGS2 | gastric cancer |
| Gancao | MOL004907 | Glyzaglabrin | PTGS2 | gastric cancer |
| Gancao | MOL004908 | Glabridin | PTGS2 | gastric cancer |
| Gancao | MOL004910 | Glabranin | PTGS2 | gastric cancer |
| Gancao | MOL004911 | Glabrene | PTGS2 | gastric cancer |
| Gancao | MOL004912 | Glabrone | PTGS2 | gastric cancer |
| Gancao | MOL004915 | Eurycarpin A | PTGS2 | gastric cancer |
| Gancao | MOL004924 | (-)-Medicocarpin | PTGS2 | gastric cancer |
| Gancao | MOL004935 | Sigmoidin-B | PTGS2 | gastric cancer |
| Gancao | MOL004941 | (2R)-7-hydroxy-2-(4-hydroxyphenyl)chroman-4-one | PTGS2 | gastric cancer |
| Gancao | MOL004945 | (2S)-7-hydroxy-2-(4-hydroxyphenyl)-8-(3-methylbut-2-enyl)chroman-4-one | PTGS2 | gastric cancer |
| Gancao | MOL004948 | Isoglycyrol | PTGS2 | gastric cancer |
| Gancao | MOL004949 | Isolicoflavonol | PTGS2 | gastric cancer |
| Gancao | MOL004957 | HMO | PTGS2 | gastric cancer |
| Gancao | MOL004959 | 1-Methoxyphaseollidin | PTGS2 | gastric cancer |
| Gancao | MOL004961 | Quercetin der. | PTGS2 | gastric cancer |
| Gancao | MOL004966 | 3'-Hydroxy-4'-O-Methylglabridin | PTGS2 | gastric cancer |
| Gancao | MOL004974 | 3'-Methoxyglabridin | PTGS2 | gastric cancer |
| Gancao | MOL004978 | 2-[(3R)-8,8-dimethyl-3,4-dihydro-2H-pyrano[6,5-f]chromen-3-yl]-5-methoxyphenol | PTGS2 | gastric cancer |
| Gancao | MOL004980 | Inflacoumarin A | PTGS2 | gastric cancer |
| Gancao | MOL004988 | Kanzonol F | PTGS2 | gastric cancer |
| Gancao | MOL004989 | 6-prenylated eriodictyol | PTGS2 | gastric cancer |
| Gancao | MOL004990 | 7,2',4'-trihydroxy－5-methoxy-3－arylcoumarin | PTGS2 | gastric cancer |
| Gancao | MOL004991 | 7-Acetoxy-2-methylisoflavone | PTGS2 | gastric cancer |
| Gancao | MOL004993 | 8-prenylated eriodictyol | PTGS2 | gastric cancer |
| Gancao | MOL005000 | Gancaonin G | PTGS2 | gastric cancer |
| Gancao | MOL005001 | Gancaonin H | PTGS2 | gastric cancer |
| Gancao | MOL005003 | Licoagrocarpin | PTGS2 | gastric cancer |
| Gancao | MOL005007 | Glyasperins M | PTGS2 | gastric cancer |
| Gancao | MOL005008 | Glycyrrhiza flavonol A | PTGS2 | gastric cancer |
| Gancao | MOL005012 | Licoagroisoflavone | PTGS2 | gastric cancer |
| Gancao | MOL005016 | Odoratin | PTGS2 | gastric cancer |
| Gancao | MOL005017 | Phaseol | PTGS2 | gastric cancer |
| Gancao | MOL005018 | Xambioona | PTGS2 | gastric cancer |
| Gancao | MOL005020 | dehydroglyasperins C | PTGS2 | gastric cancer |
| Gancao | MOL004863 | 3-(3,4-dihydroxyphenyl)-5,7-dihydroxy-8-(3-methylbut-2-enyl)chromone | PTPN1 | gastric cancer |
| Gancao | MOL004961 | Quercetin der. | PTPN1 | gastric cancer |
| Gancao | MOL000098 | quercetin | RB1 | gastric cancer |
| Gancao | MOL000497 | licochalcone a | RB1 | gastric cancer |
| Gancao | MOL000098 | quercetin | RXRA | gastric cancer |
| Gancao | MOL000392 | formononetin | RXRA | gastric cancer |
| Gancao | MOL000417 | Calycosin | RXRA | gastric cancer |
| Gancao | MOL000500 | Vestitol | RXRA | gastric cancer |
| Gancao | MOL001484 | Inermine | RXRA | gastric cancer |
| Gancao | MOL001792 | DFV | RXRA | gastric cancer |
| Gancao | MOL002565 | Medicarpin | RXRA | gastric cancer |
| Gancao | MOL002844 | Pinocembrin | RXRA | gastric cancer |
| Gancao | MOL003896 | 7-Methoxy-2-methyl isoflavone | RXRA | gastric cancer |
| Gancao | MOL004811 | Glyasperin C | RXRA | gastric cancer |
| Gancao | MOL004815 | (E)-1-(2,4-dihydroxyphenyl)-3-(2,2-dimethylchromen-6-yl)prop-2-en-1-one | RXRA | gastric cancer |
| Gancao | MOL004820 | kanzonols W | RXRA | gastric cancer |
| Gancao | MOL004828 | Glepidotin A | RXRA | gastric cancer |
| Gancao | MOL004829 | Glepidotin B | RXRA | gastric cancer |
| Gancao | MOL004833 | Phaseolinisoflavan | RXRA | gastric cancer |
| Gancao | MOL004838 | 8-(6-hydroxy-2-benzofuranyl)-2,2-dimethyl-5-chromenol | RXRA | gastric cancer |
| Gancao | MOL004891 | shinpterocarpin | RXRA | gastric cancer |
| Gancao | MOL004908 | Glabridin | RXRA | gastric cancer |
| Gancao | MOL004911 | Glabrene | RXRA | gastric cancer |
| Gancao | MOL004912 | Glabrone | RXRA | gastric cancer |
| Gancao | MOL004941 | (2R)-7-hydroxy-2-(4-hydroxyphenyl)chroman-4-one | RXRA | gastric cancer |
| Gancao | MOL004957 | HMO | RXRA | gastric cancer |
| Gancao | MOL004959 | 1-Methoxyphaseollidin | RXRA | gastric cancer |
| Gancao | MOL004974 | 3'-Methoxyglabridin | RXRA | gastric cancer |
| Gancao | MOL004978 | 2-[(3R)-8,8-dimethyl-3,4-dihydro-2H-pyrano[6,5-f]chromen-3-yl]-5-methoxyphenol | RXRA | gastric cancer |
| Gancao | MOL004991 | 7-Acetoxy-2-methylisoflavone | RXRA | gastric cancer |
| Gancao | MOL005003 | Licoagrocarpin | RXRA | gastric cancer |
| Gancao | MOL005016 | Odoratin | RXRA | gastric cancer |
| Gancao | MOL004891 | shinpterocarpin | RXRB | gastric cancer |
| Gancao | MOL004908 | Glabridin | RXRB | gastric cancer |
| Gancao | MOL004978 | 2-[(3R)-8,8-dimethyl-3,4-dihydro-2H-pyrano[6,5-f]chromen-3-yl]-5-methoxyphenol | RXRB | gastric cancer |
| Gancao | MOL005003 | Licoagrocarpin | RXRB | gastric cancer |
| Gancao | MOL000098 | quercetin | SELE | gastric cancer |
| Gancao | MOL000422 | kaempferol | SELE | gastric cancer |
| Gancao | MOL000392 | formononetin | SLC6A3 | gastric cancer |
| Gancao | MOL000497 | licochalcone a | SLC6A3 | gastric cancer |
| Gancao | MOL000500 | Vestitol | SLC6A3 | gastric cancer |
| Gancao | MOL002565 | Medicarpin | SLC6A3 | gastric cancer |
| Gancao | MOL003896 | 7-Methoxy-2-methyl isoflavone | SLC6A3 | gastric cancer |
| Gancao | MOL004835 | Glypallichalcone | SLC6A3 | gastric cancer |
| Gancao | MOL004957 | HMO | SLC6A3 | gastric cancer |
| Gancao | MOL004978 | 2-[(3R)-8,8-dimethyl-3,4-dihydro-2H-pyrano[6,5-f]chromen-3-yl]-5-methoxyphenol | SLC6A3 | gastric cancer |
| Gancao | MOL000392 | formononetin | SLC6A4 | gastric cancer |
| Gancao | MOL000500 | Vestitol | SLC6A4 | gastric cancer |
| Gancao | MOL001792 | DFV | SLC6A4 | gastric cancer |
| Gancao | MOL002565 | Medicarpin | SLC6A4 | gastric cancer |
| Gancao | MOL002844 | Pinocembrin | SLC6A4 | gastric cancer |
| Gancao | MOL003896 | 7-Methoxy-2-methyl isoflavone | SLC6A4 | gastric cancer |
| Gancao | MOL004835 | Glypallichalcone | SLC6A4 | gastric cancer |
| Gancao | MOL004941 | (2R)-7-hydroxy-2-(4-hydroxyphenyl)chroman-4-one | SLC6A4 | gastric cancer |
| Gancao | MOL004957 | HMO | SLC6A4 | gastric cancer |
| Gancao | MOL004328 | naringenin | SOAT1 | gastric cancer |
| Gancao | MOL000098 | quercetin | SOD1 | gastric cancer |
| Gancao | MOL004328 | naringenin | SOD1 | gastric cancer |
| Gancao | MOL004903 | liquiritin | SOD1 | gastric cancer |
| Gancao | MOL000098 | quercetin | THBD | gastric cancer |
| Gancao | MOL000098 | quercetin | TNF | gastric cancer |
| Gancao | MOL000422 | kaempferol | TNF | gastric cancer |
| Gancao | MOL000098 | quercetin | TOP1 | gastric cancer |
| Gancao | MOL000098 | quercetin | TOP2A | gastric cancer |
| Gancao | MOL000098 | quercetin | TOP2A | gastric cancer |
| Gancao | MOL000422 | kaempferol | TOP2A | gastric cancer |
| Gancao | MOL000098 | quercetin | TP53 | gastric cancer |
| Gancao | MOL000098 | quercetin | VCAM1 | gastric cancer |
| Gancao | MOL000422 | kaempferol | VCAM1 | gastric cancer |
| Gancao | MOL000098 | quercetin | VEGFA | gastric cancer |
| Gancao | MOL000098 | quercetin | XDH | gastric cancer |
| Gancao | MOL000354 | isorhamnetin | XDH | gastric cancer |
| Gancao | MOL000422 | kaempferol | XDH | gastric cancer |
| Renshen | MOL000422 | kaempferol | ACHE | gastric cancer |
| Renshen | MOL005321 | Frutinone A | ACHE | gastric cancer |
| Renshen | MOL000358 | beta-sitosterol | ADRA1A | gastric cancer |
| Renshen | MOL000449 | Stigmasterol | ADRA1A | gastric cancer |
| Renshen | MOL000358 | beta-sitosterol | ADRA1B | gastric cancer |
| Renshen | MOL000422 | kaempferol | ADRA1B | gastric cancer |
| Renshen | MOL000449 | Stigmasterol | ADRA1B | gastric cancer |
| Renshen | MOL000787 | Fumarine | ADRA1B | gastric cancer |
| Renshen | MOL003648 | Inermin | ADRA1B | gastric cancer |
| Renshen | MOL000787 | Fumarine | ADRA1D | gastric cancer |
| Renshen | MOL003648 | Inermin | ADRA1D | gastric cancer |
| Renshen | MOL005384 | suchilactone | ADRA1D | gastric cancer |
| Renshen | MOL000449 | Stigmasterol | ADRA2A | gastric cancer |
| Renshen | MOL000449 | Stigmasterol | ADRB1 | gastric cancer |
| Renshen | MOL000358 | beta-sitosterol | ADRB2 | gastric cancer |
| Renshen | MOL000449 | Stigmasterol | ADRB2 | gastric cancer |
| Renshen | MOL000787 | Fumarine | ADRB2 | gastric cancer |
| Renshen | MOL002879 | Diop | ADRB2 | gastric cancer |
| Renshen | MOL003648 | Inermin | ADRB2 | gastric cancer |
| Renshen | MOL005308 | Aposiopolamine | ADRB2 | gastric cancer |
| Renshen | MOL005321 | Frutinone A | ADRB2 | gastric cancer |
| Renshen | MOL005356 | Girinimbin | ADRB2 | gastric cancer |
| Renshen | MOL005384 | suchilactone | ADRB2 | gastric cancer |
| Renshen | MOL000422 | kaempferol | AHR | gastric cancer |
| Renshen | MOL000449 | Stigmasterol | AKR1B10 | gastric cancer |
| Renshen | MOL000422 | kaempferol | AKR1C3 | gastric cancer |
| Renshen | MOL000422 | kaempferol | ALOX5 | gastric cancer |
| Renshen | MOL000422 | kaempferol | AR | gastric cancer |
| Renshen | MOL005317 | Deoxyharringtonine | AR | gastric cancer |
| Renshen | MOL005321 | Frutinone A | AR | gastric cancer |
| Renshen | MOL000358 | beta-sitosterol | BAX | gastric cancer |
| Renshen | MOL000358 | beta-sitosterol | BCL2 | gastric cancer |
| Renshen | MOL000422 | kaempferol | BCL2 | gastric cancer |
| Renshen | MOL000422 | kaempferol | CAMKK2 | gastric cancer |
| Renshen | MOL000787 | Fumarine | CAMKK2 | gastric cancer |
| Renshen | MOL003648 | Inermin | CAMKK2 | gastric cancer |
| Renshen | MOL005384 | suchilactone | CAMKK2 | gastric cancer |
| Renshen | MOL000422 | kaempferol | CDK1 | gastric cancer |
| Renshen | MOL000358 | beta-sitosterol | CHRM1 | gastric cancer |
| Renshen | MOL000422 | kaempferol | CHRM1 | gastric cancer |
| Renshen | MOL000449 | Stigmasterol | CHRM1 | gastric cancer |
| Renshen | MOL000787 | Fumarine | CHRM1 | gastric cancer |
| Renshen | MOL005308 | Aposiopolamine | CHRM1 | gastric cancer |
| Renshen | MOL000358 | beta-sitosterol | CHRM2 | gastric cancer |
| Renshen | MOL000422 | kaempferol | CHRM2 | gastric cancer |
| Renshen | MOL000449 | Stigmasterol | CHRM2 | gastric cancer |
| Renshen | MOL000358 | beta-sitosterol | CHRM3 | gastric cancer |
| Renshen | MOL000449 | Stigmasterol | CHRM3 | gastric cancer |
| Renshen | MOL000787 | Fumarine | CHRM3 | gastric cancer |
| Renshen | MOL002879 | Diop | CHRM3 | gastric cancer |
| Renshen | MOL003648 | Inermin | CHRM3 | gastric cancer |
| Renshen | MOL005308 | Aposiopolamine | CHRM3 | gastric cancer |
| Renshen | MOL000358 | beta-sitosterol | CHRNA7 | gastric cancer |
| Renshen | MOL000449 | Stigmasterol | CHRNA7 | gastric cancer |
| Renshen | MOL003648 | Inermin | CHRNA7 | gastric cancer |
| Renshen | MOL005321 | Frutinone A | CHRNA7 | gastric cancer |
| Renshen | MOL005356 | Girinimbin | CHRNA7 | gastric cancer |
| Renshen | MOL000422 | kaempferol | CYP1A2 | gastric cancer |
| Renshen | MOL000422 | kaempferol | CYP3A4 | gastric cancer |
| Renshen | MOL000358 | beta-sitosterol | DRD1 | gastric cancer |
| Renshen | MOL000787 | Fumarine | DRD1 | gastric cancer |
| Renshen | MOL000787 | Fumarine | F10 | gastric cancer |
| Renshen | MOL005384 | suchilactone | F10 | gastric cancer |
| Renshen | MOL000422 | kaempferol | F7 | gastric cancer |
| Renshen | MOL000787 | Fumarine | F7 | gastric cancer |
| Renshen | MOL005384 | suchilactone | F7 | gastric cancer |
| Renshen | MOL000422 | kaempferol | GSTP1 | gastric cancer |
| Renshen | MOL000422 | kaempferol | HMOX1 | gastric cancer |
| Renshen | MOL000358 | beta-sitosterol | HTR2A | gastric cancer |
| Renshen | MOL000449 | Stigmasterol | HTR2A | gastric cancer |
| Renshen | MOL000787 | Fumarine | HTR2A | gastric cancer |
| Renshen | MOL000787 | Fumarine | HTR3A | gastric cancer |
| Renshen | MOL003648 | Inermin | HTR3A | gastric cancer |
| Renshen | MOL005344 | ginsenoside rh2 | IFNG | gastric cancer |
| Renshen | MOL005344 | ginsenoside rh2 | IL1B | gastric cancer |
| Renshen | MOL000422 | kaempferol | INSR | gastric cancer |
| Renshen | MOL000358 | beta-sitosterol | JUN | gastric cancer |
| Renshen | MOL000422 | kaempferol | JUN | gastric cancer |
| Renshen | MOL000358 | beta-sitosterol | KCNH2 | gastric cancer |
| Renshen | MOL000787 | Fumarine | KCNH2 | gastric cancer |
| Renshen | MOL005384 | suchilactone | KCNH2 | gastric cancer |
| Renshen | MOL005384 | suchilactone | KCNMA1 | gastric cancer |
| Renshen | MOL000787 | Fumarine | KDR | gastric cancer |
| Renshen | MOL000449 | Stigmasterol | LTA4H | gastric cancer |
| Renshen | MOL000449 | Stigmasterol | MAOA | gastric cancer |
| Renshen | MOL000449 | Stigmasterol | MAOB | gastric cancer |
| Renshen | MOL000358 | beta-sitosterol | MAP2 | gastric cancer |
| Renshen | MOL000422 | kaempferol | MAPK8 | gastric cancer |
| Renshen | MOL000422 | kaempferol | MMP1 | gastric cancer |
| Renshen | MOL000449 | Stigmasterol | NCOA1 | gastric cancer |
| Renshen | MOL003648 | Inermin | NCOA1 | gastric cancer |
| Renshen | MOL005384 | suchilactone | NCOA1 | gastric cancer |
| Renshen | MOL000358 | beta-sitosterol | NCOA2 | gastric cancer |
| Renshen | MOL000422 | kaempferol | NCOA2 | gastric cancer |
| Renshen | MOL000449 | Stigmasterol | NCOA2 | gastric cancer |
| Renshen | MOL005320 | arachidonate | NCOA2 | gastric cancer |
| Renshen | MOL005348 | Ginsenoside-Rh4_qt | NCOA2 | gastric cancer |
| Renshen | MOL005356 | Girinimbin | NCOA2 | gastric cancer |
| Renshen | MOL000422 | kaempferol | NOS2 | gastric cancer |
| Renshen | MOL000422 | kaempferol | NOS3 | gastric cancer |
| Renshen | MOL005376 | Panaxadiol | NR3C1 | gastric cancer |
| Renshen | MOL000449 | Stigmasterol | NR3C2 | gastric cancer |
| Renshen | MOL005317 | Deoxyharringtonine | NR3C2 | gastric cancer |
| Renshen | MOL005348 | Ginsenoside-Rh4_qt | NR3C2 | gastric cancer |
| Renshen | MOL000787 | Fumarine | OPRD1 | gastric cancer |
| Renshen | MOL000358 | beta-sitosterol | OPRM1 | gastric cancer |
| Renshen | MOL000787 | Fumarine | OPRM1 | gastric cancer |
| Renshen | MOL000358 | beta-sitosterol | PDE3A | gastric cancer |
| Renshen | MOL000787 | Fumarine | PDE3A | gastric cancer |
| Renshen | MOL000787 | Fumarine | PDE3A | gastric cancer |
| Renshen | MOL005321 | Frutinone A | PDE3A | gastric cancer |
| Renshen | MOL005384 | suchilactone | PDE3A | gastric cancer |
| Renshen | MOL000358 | beta-sitosterol | PGR | gastric cancer |
| Renshen | MOL000422 | kaempferol | PGR | gastric cancer |
| Renshen | MOL000449 | Stigmasterol | PGR | gastric cancer |
| Renshen | MOL005399 | alexandrin_qt | PGR | gastric cancer |
| Renshen | MOL000358 | beta-sitosterol | PIK3CG | gastric cancer |
| Renshen | MOL000422 | kaempferol | PIK3CG | gastric cancer |
| Renshen | MOL003648 | Inermin | PIK3CG | gastric cancer |
| Renshen | MOL005321 | Frutinone A | PIK3CG | gastric cancer |
| Renshen | MOL005356 | Girinimbin | PIK3CG | gastric cancer |
| Renshen | MOL000449 | Stigmasterol | PLAU | gastric cancer |
| Renshen | MOL000358 | beta-sitosterol | PON1 | gastric cancer |
| Renshen | MOL000422 | kaempferol | PPARD | gastric cancer |
| Renshen | MOL005321 | Frutinone A | PPARD | gastric cancer |
| Renshen | MOL000422 | kaempferol | PPP3CA | gastric cancer |
| Renshen | MOL000358 | beta-sitosterol | PRKACA | gastric cancer |
| Renshen | MOL000422 | kaempferol | PRKACA | gastric cancer |
| Renshen | MOL000449 | Stigmasterol | PRKACA | gastric cancer |
| Renshen | MOL000787 | Fumarine | PRKACA | gastric cancer |
| Renshen | MOL003648 | Inermin | PRKACA | gastric cancer |
| Renshen | MOL005321 | Frutinone A | PRKACA | gastric cancer |
| Renshen | MOL005356 | Girinimbin | PRKACA | gastric cancer |
| Renshen | MOL005384 | suchilactone | PRKACA | gastric cancer |
| Renshen | MOL005384 | suchilactone | PRKACA | gastric cancer |
| Renshen | MOL000422 | kaempferol | PRSS1 | gastric cancer |
| Renshen | MOL003648 | Inermin | PRSS1 | gastric cancer |
| Renshen | MOL000358 | beta-sitosterol | PTGS1 | gastric cancer |
| Renshen | MOL000422 | kaempferol | PTGS1 | gastric cancer |
| Renshen | MOL000449 | Stigmasterol | PTGS1 | gastric cancer |
| Renshen | MOL000787 | Fumarine | PTGS1 | gastric cancer |
| Renshen | MOL003648 | Inermin | PTGS1 | gastric cancer |
| Renshen | MOL005318 | Dianthramine | PTGS1 | gastric cancer |
| Renshen | MOL005320 | arachidonate | PTGS1 | gastric cancer |
| Renshen | MOL005321 | Frutinone A | PTGS1 | gastric cancer |
| Renshen | MOL005356 | Girinimbin | PTGS1 | gastric cancer |
| Renshen | MOL005384 | suchilactone | PTGS1 | gastric cancer |
| Renshen | MOL000358 | beta-sitosterol | PTGS2 | gastric cancer |
| Renshen | MOL000422 | kaempferol | PTGS2 | gastric cancer |
| Renshen | MOL000449 | Stigmasterol | PTGS2 | gastric cancer |
| Renshen | MOL000787 | Fumarine | PTGS2 | gastric cancer |
| Renshen | MOL003648 | Inermin | PTGS2 | gastric cancer |
| Renshen | MOL005318 | Dianthramine | PTGS2 | gastric cancer |
| Renshen | MOL005320 | arachidonate | PTGS2 | gastric cancer |
| Renshen | MOL005321 | Frutinone A | PTGS2 | gastric cancer |
| Renshen | MOL005344 | ginsenoside rh2 | PTGS2 | gastric cancer |
| Renshen | MOL005356 | Girinimbin | PTGS2 | gastric cancer |
| Renshen | MOL005384 | suchilactone | PTGS2 | gastric cancer |
| Renshen | MOL000449 | Stigmasterol | RXRA | gastric cancer |
| Renshen | MOL003648 | Inermin | RXRA | gastric cancer |
| Renshen | MOL005321 | Frutinone A | RXRA | gastric cancer |
| Renshen | MOL005356 | Girinimbin | RXRA | gastric cancer |
| Renshen | MOL005384 | suchilactone | RXRA | gastric cancer |
| Renshen | MOL000422 | kaempferol | SELE | gastric cancer |
| Renshen | MOL000449 | Stigmasterol | SLC6A3 | gastric cancer |
| Renshen | MOL000787 | Fumarine | SLC6A3 | gastric cancer |
| Renshen | MOL005308 | Aposiopolamine | SLC6A3 | gastric cancer |
| Renshen | MOL000358 | beta-sitosterol | SLC6A4 | gastric cancer |
| Renshen | MOL000787 | Fumarine | SLC6A4 | gastric cancer |
| Renshen | MOL003648 | Inermin | SLC6A4 | gastric cancer |
| Renshen | MOL005308 | Aposiopolamine | SLC6A4 | gastric cancer |
| Renshen | MOL000422 | kaempferol | TNF | gastric cancer |
| Renshen | MOL005344 | ginsenoside rh2 | TNF | gastric cancer |
| Renshen | MOL000422 | kaempferol | TOP2A | gastric cancer |
| Renshen | MOL000787 | Fumarine | TOP2A | gastric cancer |
| Renshen | MOL000422 | kaempferol | VCAM1 | gastric cancer |
| Renshen | MOL000422 | kaempferol | XDH | gastric cancer |
