# Supplementary material for: A Network Pharmacology Approach for Uncovering the Antitumor Effects and Potential Mechanisms of the Sijunzi Decoction for the Treatment of Gastric Cancer
Source: Evid Based Complement Alternat Med. 2022 Apr 12;2022:9364313. doi: 10.1155/2022/9364313 (PMC9019414; doi:10.1155/2022/9364313)
Supplement: Supplementary Materials — Supplement Table 1. The active compounds of SJZD. Supplement Table 2. TCM-TCM-compound-target-disease analysis. Supplemental Table 3. PPI analysis. [file 9364313.f1.zip › 9364313.f1/Supplement Table3 (1).docx]

**Supplemental Table.3 PPI analysis**

| #node1 | node2 | node1_string_id | node2_string_id | neighborhood_on_chromosome | gene_fusion | phylogenetic_cooccurrence | homology | coexpression | experimentally_determined_interaction | database_annotated | automated_textmining | combined_score |
| --- | --- | --- | --- | --- | --- | --- | --- | --- | --- | --- | --- | --- |
| ABCC1 | GSR | 9606.ENSP00000382342 | 9606.ENSP00000221130 | 0 | 0 | 0 | 0 | 0.094 | 0.137 | 0 | 0.385 | 0.477 |
| ABCC1 | TP53 | 9606.ENSP00000382342 | 9606.ENSP00000269305 | 0 | 0 | 0 | 0 | 0 | 0 | 0 | 0.46 | 0.459 |
| ABCC1 | SOD1 | 9606.ENSP00000382342 | 9606.ENSP00000270142 | 0 | 0 | 0 | 0 | 0 | 0.131 | 0 | 0.387 | 0.445 |
| ABCC1 | EGFR | 9606.ENSP00000382342 | 9606.ENSP00000275493 | 0 | 0 | 0 | 0 | 0 | 0 | 0 | 0.478 | 0.478 |
| ABCC1 | CYP3A4 | 9606.ENSP00000382342 | 9606.ENSP00000337915 | 0 | 0 | 0 | 0 | 0.063 | 0 | 0 | 0.528 | 0.539 |
| ABCC1 | DPP4 | 9606.ENSP00000382342 | 9606.ENSP00000353731 | 0 | 0 | 0 | 0 | 0.062 | 0 | 0 | 0.433 | 0.445 |
| ABCC1 | TOP1 | 9606.ENSP00000382342 | 9606.ENSP00000354522 | 0 | 0 | 0 | 0 | 0 | 0.076 | 0 | 0.408 | 0.43 |
| ABCC1 | GSTP1 | 9606.ENSP00000382342 | 9606.ENSP00000381607 | 0 | 0 | 0 | 0 | 0.054 | 0 | 0 | 0.483 | 0.49 |
| ABCC1 | TNF | 9606.ENSP00000382342 | 9606.ENSP00000398698 | 0 | 0 | 0 | 0 | 0 | 0 | 0 | 0.447 | 0.447 |
| ACACA | PPARG | 9606.ENSP00000483300 | 9606.ENSP00000287820 | 0 | 0 | 0 | 0 | 0.049 | 0 | 0 | 0.682 | 0.685 |
| ACACA | HMGCR | 9606.ENSP00000483300 | 9606.ENSP00000287936 | 0.05 | 0 | 0 | 0 | 0.069 | 0 | 0 | 0.768 | 0.777 |
| ACACA | FASN | 9606.ENSP00000483300 | 9606.ENSP00000304592 | 0.056 | 0 | 0 | 0 | 0.684 | 0.068 | 0.9 | 0.922 | 0.997 |
| ACACA | CAMKK2 | 9606.ENSP00000483300 | 9606.ENSP00000312741 | 0 | 0 | 0 | 0 | 0 | 0 | 0 | 0.424 | 0.424 |
| ACACA | AKR1B10 | 9606.ENSP00000483300 | 9606.ENSP00000352584 | 0 | 0 | 0 | 0 | 0.062 | 0.27 | 0 | 0.452 | 0.592 |
| ACACA | LDLR | 9606.ENSP00000483300 | 9606.ENSP00000454071 | 0 | 0 | 0 | 0 | 0 | 0 | 0 | 0.465 | 0.465 |
| ACHE | GSR | 9606.ENSP00000303211 | 9606.ENSP00000221130 | 0 | 0 | 0 | 0 | 0 | 0 | 0 | 0.4 | 0.4 |
| ACHE | PON1 | 9606.ENSP00000303211 | 9606.ENSP00000222381 | 0 | 0 | 0 | 0 | 0.063 | 0 | 0 | 0.659 | 0.667 |
| ACHE | CAT | 9606.ENSP00000303211 | 9606.ENSP00000241052 | 0 | 0 | 0 | 0 | 0.049 | 0 | 0 | 0.674 | 0.677 |
| ACHE | SLC6A4 | 9606.ENSP00000303211 | 9606.ENSP00000261707 | 0 | 0 | 0 | 0 | 0 | 0 | 0 | 0.417 | 0.417 |
| ACHE | IL1B | 9606.ENSP00000303211 | 9606.ENSP00000263341 | 0 | 0 | 0 | 0 | 0 | 0 | 0 | 0.508 | 0.508 |
| ACHE | SLC6A3 | 9606.ENSP00000303211 | 9606.ENSP00000270349 | 0 | 0 | 0 | 0 | 0.062 | 0 | 0 | 0.442 | 0.454 |
| ACHE | GSK3B | 9606.ENSP00000303211 | 9606.ENSP00000324806 | 0 | 0 | 0 | 0 | 0.048 | 0.062 | 0 | 0.408 | 0.425 |
| ACHE | CHRM2 | 9606.ENSP00000303211 | 9606.ENSP00000399745 | 0 | 0 | 0 | 0 | 0.065 | 0 | 0 | 0.461 | 0.474 |
| ACHE | PTGS2 | 9606.ENSP00000303211 | 9606.ENSP00000356438 | 0 | 0 | 0 | 0 | 0 | 0.059 | 0 | 0.467 | 0.477 |
| ACHE | TNF | 9606.ENSP00000303211 | 9606.ENSP00000398698 | 0 | 0 | 0 | 0 | 0 | 0 | 0 | 0.521 | 0.521 |
| ACHE | IL6 | 9606.ENSP00000303211 | 9606.ENSP00000385675 | 0 | 0 | 0 | 0 | 0 | 0 | 0 | 0.536 | 0.536 |
| ACHE | CHRNA7 | 9606.ENSP00000303211 | 9606.ENSP00000407546 | 0 | 0 | 0 | 0 | 0.062 | 0 | 0 | 0.553 | 0.563 |
| ACHE | CHRM1 | 9606.ENSP00000303211 | 9606.ENSP00000306490 | 0 | 0 | 0 | 0 | 0.146 | 0 | 0 | 0.556 | 0.605 |
| ACHE | MAOA | 9606.ENSP00000303211 | 9606.ENSP00000340684 | 0.047 | 0 | 0 | 0 | 0.096 | 0 | 0 | 0.603 | 0.628 |
| ACHE | MAOB | 9606.ENSP00000303211 | 9606.ENSP00000367309 | 0.047 | 0 | 0 | 0 | 0.107 | 0 | 0 | 0.662 | 0.687 |
| ADH1B | OPRD1 | 9606.ENSP00000306606 | 9606.ENSP00000234961 | 0 | 0 | 0 | 0 | 0 | 0 | 0 | 0.49 | 0.489 |
| ADH1B | CAT | 9606.ENSP00000306606 | 9606.ENSP00000241052 | 0 | 0 | 0 | 0 | 0.062 | 0 | 0 | 0.387 | 0.4 |
| ADH1B | SLC6A4 | 9606.ENSP00000306606 | 9606.ENSP00000261707 | 0 | 0 | 0 | 0 | 0 | 0 | 0 | 0.423 | 0.422 |
| ADH1B | CHRM2 | 9606.ENSP00000306606 | 9606.ENSP00000399745 | 0 | 0 | 0 | 0 | 0 | 0 | 0 | 0.412 | 0.412 |
| ADH1B | OPRM1 | 9606.ENSP00000306606 | 9606.ENSP00000394624 | 0 | 0 | 0 | 0 | 0 | 0 | 0 | 0.46 | 0.459 |
| ADH1B | AKR1B10 | 9606.ENSP00000306606 | 9606.ENSP00000352584 | 0 | 0 | 0 | 0 | 0.063 | 0.16 | 0 | 0.414 | 0.498 |
| ADH1B | MAOB | 9606.ENSP00000306606 | 9606.ENSP00000367309 | 0 | 0 | 0 | 0 | 0.104 | 0 | 0.65 | 0.203 | 0.728 |
| ADH1B | GSTP1 | 9606.ENSP00000306606 | 9606.ENSP00000381607 | 0 | 0 | 0 | 0 | 0 | 0.166 | 0.65 | 0.325 | 0.785 |
| ADH1B | MAOA | 9606.ENSP00000306606 | 9606.ENSP00000340684 | 0 | 0 | 0 | 0 | 0.104 | 0 | 0.9 | 0.409 | 0.942 |
| ADRA1A | SLC6A4 | 9606.ENSP00000369960 | 9606.ENSP00000261707 | 0 | 0 | 0 | 0 | 0 | 0.057 | 0 | 0.471 | 0.48 |
| ADRA1A | SLC6A3 | 9606.ENSP00000369960 | 9606.ENSP00000270349 | 0 | 0 | 0 | 0 | 0 | 0.057 | 0 | 0.457 | 0.466 |
| ADRA1A | ADRA1B | 9606.ENSP00000369960 | 9606.ENSP00000306662 | 0 | 0 | 0 | 0.936 | 0.062 | 0.676 | 0.8 | 0.883 | 0.937 |
| ADRA1A | ADRA1D | 9606.ENSP00000369960 | 9606.ENSP00000368766 | 0 | 0 | 0 | 0.932 | 0.062 | 0.213 | 0.8 | 0.543 | 0.845 |
| ADRA1B | SLC6A4 | 9606.ENSP00000306662 | 9606.ENSP00000261707 | 0 | 0 | 0 | 0 | 0.062 | 0.057 | 0 | 0.429 | 0.451 |
| ADRA1B | SLC6A3 | 9606.ENSP00000306662 | 9606.ENSP00000270349 | 0 | 0 | 0 | 0 | 0 | 0.057 | 0 | 0.41 | 0.42 |
| ADRA1B | ADRB2 | 9606.ENSP00000306662 | 9606.ENSP00000305372 | 0 | 0 | 0 | 0.801 | 0 | 0 | 0.9 | 0.767 | 0.915 |
| ADRA1B | CHRM1 | 9606.ENSP00000306662 | 9606.ENSP00000306490 | 0 | 0 | 0 | 0.676 | 0.076 | 0 | 0.9 | 0.506 | 0.918 |
| ADRA1B | ADRA1D | 9606.ENSP00000306662 | 9606.ENSP00000368766 | 0 | 0 | 0 | 0.932 | 0.111 | 0.27 | 0.8 | 0.812 | 0.866 |
| ADRA1B | F2 | 9606.ENSP00000306662 | 9606.ENSP00000308541 | 0 | 0 | 0 | 0 | 0.066 | 0.097 | 0.9 | 0 | 0.908 |
| ADRA1B | ADRA1A | 9606.ENSP00000306662 | 9606.ENSP00000369960 | 0 | 0 | 0 | 0.936 | 0.062 | 0.676 | 0.8 | 0.883 | 0.937 |
| ADRA1D | SLC6A4 | 9606.ENSP00000368766 | 9606.ENSP00000261707 | 0 | 0 | 0 | 0 | 0 | 0.057 | 0 | 0.477 | 0.485 |
| ADRA1D | SLC6A3 | 9606.ENSP00000368766 | 9606.ENSP00000270349 | 0 | 0 | 0 | 0 | 0 | 0.057 | 0 | 0.469 | 0.478 |
| ADRA1D | ADRA1B | 9606.ENSP00000368766 | 9606.ENSP00000306662 | 0 | 0 | 0 | 0.932 | 0.111 | 0.27 | 0.8 | 0.812 | 0.866 |
| ADRA1D | HTR3A | 9606.ENSP00000368766 | 9606.ENSP00000347754 | 0 | 0 | 0 | 0 | 0 | 0 | 0 | 0.411 | 0.411 |
| ADRA1D | CHRNA7 | 9606.ENSP00000368766 | 9606.ENSP00000407546 | 0 | 0 | 0 | 0 | 0.062 | 0 | 0 | 0.432 | 0.445 |
| ADRA1D | ADRA1A | 9606.ENSP00000368766 | 9606.ENSP00000369960 | 0 | 0 | 0 | 0.932 | 0.062 | 0.213 | 0.8 | 0.543 | 0.845 |
| ADRA2A | SLC6A4 | 9606.ENSP00000280155 | 9606.ENSP00000261707 | 0 | 0 | 0 | 0 | 0 | 0.057 | 0 | 0.564 | 0.571 |
| ADRA2A | SLC6A3 | 9606.ENSP00000280155 | 9606.ENSP00000270349 | 0 | 0 | 0 | 0 | 0 | 0.057 | 0 | 0.556 | 0.563 |
| ADRA2A | HTR3A | 9606.ENSP00000280155 | 9606.ENSP00000347754 | 0 | 0 | 0 | 0 | 0.062 | 0 | 0 | 0.422 | 0.434 |
| ADRA2A | CHRNA7 | 9606.ENSP00000280155 | 9606.ENSP00000407546 | 0 | 0 | 0 | 0 | 0.062 | 0 | 0 | 0.424 | 0.436 |
| ADRA2A | MAOA | 9606.ENSP00000280155 | 9606.ENSP00000340684 | 0 | 0 | 0 | 0 | 0.065 | 0 | 0 | 0.426 | 0.44 |
| ADRB1 | EGFR | 9606.ENSP00000358301 | 9606.ENSP00000275493 | 0 | 0 | 0 | 0 | 0 | 0.122 | 0 | 0.684 | 0.711 |
| ADRB1 | NOS3 | 9606.ENSP00000358301 | 9606.ENSP00000297494 | 0 | 0 | 0 | 0 | 0.062 | 0 | 0 | 0.413 | 0.426 |
| ADRB1 | ADRB2 | 9606.ENSP00000358301 | 9606.ENSP00000305372 | 0 | 0 | 0 | 0.937 | 0 | 0.182 | 0.8 | 0.985 | 0.839 |
| ADRB2 | NR3C1 | 9606.ENSP00000305372 | 9606.ENSP00000231509 | 0 | 0 | 0 | 0 | 0 | 0.056 | 0 | 0.543 | 0.55 |
| ADRB2 | OPRD1 | 9606.ENSP00000305372 | 9606.ENSP00000234961 | 0 | 0 | 0 | 0.674 | 0 | 0.312 | 0 | 0.487 | 0.416 |
| ADRB2 | SLC6A4 | 9606.ENSP00000305372 | 9606.ENSP00000261707 | 0 | 0 | 0 | 0 | 0 | 0.057 | 0 | 0.456 | 0.465 |
| ADRB2 | MAPK3 | 9606.ENSP00000305372 | 9606.ENSP00000263025 | 0 | 0 | 0 | 0 | 0 | 0 | 0 | 0.505 | 0.505 |
| ADRB2 | IL1B | 9606.ENSP00000305372 | 9606.ENSP00000263341 | 0 | 0 | 0 | 0 | 0.088 | 0.213 | 0 | 0.413 | 0.541 |
| ADRB2 | EGFR | 9606.ENSP00000305372 | 9606.ENSP00000275493 | 0 | 0 | 0 | 0 | 0 | 0.122 | 0 | 0.491 | 0.534 |
| ADRB2 | PPARG | 9606.ENSP00000305372 | 9606.ENSP00000287820 | 0 | 0 | 0 | 0 | 0 | 0.056 | 0 | 0.457 | 0.465 |
| ADRB2 | NOS3 | 9606.ENSP00000305372 | 9606.ENSP00000297494 | 0 | 0 | 0 | 0 | 0.062 | 0 | 0 | 0.508 | 0.518 |
| ADRB2 | INSR | 9606.ENSP00000305372 | 9606.ENSP00000303830 | 0 | 0 | 0 | 0 | 0 | 0.225 | 0 | 0.703 | 0.76 |
| ADRB2 | PTGER3 | 9606.ENSP00000305372 | 9606.ENSP00000349003 | 0 | 0 | 0 | 0 | 0 | 0 | 0 | 0.475 | 0.475 |
| ADRB2 | IL6 | 9606.ENSP00000305372 | 9606.ENSP00000385675 | 0 | 0 | 0 | 0 | 0.063 | 0 | 0 | 0.47 | 0.482 |
| ADRB2 | TNF | 9606.ENSP00000305372 | 9606.ENSP00000398698 | 0 | 0 | 0 | 0 | 0.065 | 0 | 0 | 0.554 | 0.566 |
| ADRB2 | CHRM2 | 9606.ENSP00000305372 | 9606.ENSP00000399745 | 0 | 0 | 0 | 0.652 | 0.052 | 0 | 0.5 | 0.522 | 0.592 |
| ADRB2 | ADRB1 | 9606.ENSP00000305372 | 9606.ENSP00000358301 | 0 | 0 | 0 | 0.937 | 0 | 0.182 | 0.8 | 0.985 | 0.839 |
| ADRB2 | CHRM1 | 9606.ENSP00000305372 | 9606.ENSP00000306490 | 0 | 0 | 0 | 0.681 | 0.049 | 0 | 0.9 | 0.498 | 0.915 |
| ADRB2 | ADRA1B | 9606.ENSP00000305372 | 9606.ENSP00000306662 | 0 | 0 | 0 | 0.801 | 0 | 0 | 0.9 | 0.767 | 0.915 |
| ADRB2 | F2 | 9606.ENSP00000305372 | 9606.ENSP00000308541 | 0 | 0 | 0 | 0 | 0 | 0.097 | 0.9 | 0.173 | 0.918 |
| AHR | HMOX1 | 9606.ENSP00000242057 | 9606.ENSP00000216117 | 0 | 0 | 0 | 0 | 0.062 | 0 | 0 | 0.51 | 0.52 |
| AHR | IL2 | 9606.ENSP00000242057 | 9606.ENSP00000226730 | 0 | 0 | 0 | 0 | 0 | 0 | 0 | 0.503 | 0.503 |
| AHR | IFNG | 9606.ENSP00000242057 | 9606.ENSP00000229135 | 0 | 0 | 0 | 0 | 0 | 0 | 0 | 0.486 | 0.486 |
| AHR | NR3C1 | 9606.ENSP00000242057 | 9606.ENSP00000231509 | 0 | 0 | 0 | 0 | 0.059 | 0.057 | 0 | 0.724 | 0.733 |
| AHR | CAT | 9606.ENSP00000242057 | 9606.ENSP00000241052 | 0 | 0 | 0 | 0 | 0.062 | 0 | 0 | 0.391 | 0.404 |
| AHR | EGFR | 9606.ENSP00000242057 | 9606.ENSP00000275493 | 0 | 0 | 0 | 0 | 0.062 | 0 | 0 | 0.412 | 0.425 |
| AHR | PGR | 9606.ENSP00000242057 | 9606.ENSP00000325120 | 0 | 0 | 0 | 0 | 0 | 0.057 | 0 | 0.43 | 0.44 |
| AHR | EGF | 9606.ENSP00000242057 | 9606.ENSP00000265171 | 0 | 0 | 0 | 0 | 0 | 0 | 0 | 0.444 | 0.444 |
| AHR | VEGFA | 9606.ENSP00000242057 | 9606.ENSP00000478570 | 0 | 0 | 0 | 0 | 0 | 0 | 0 | 0.457 | 0.457 |
| AHR | GSTP1 | 9606.ENSP00000242057 | 9606.ENSP00000381607 | 0 | 0 | 0 | 0 | 0 | 0 | 0 | 0.477 | 0.477 |
| AHR | PTGS2 | 9606.ENSP00000242057 | 9606.ENSP00000356438 | 0 | 0 | 0 | 0 | 0.098 | 0 | 0 | 0.469 | 0.5 |
| AHR | TP53 | 9606.ENSP00000242057 | 9606.ENSP00000269305 | 0 | 0 | 0 | 0 | 0 | 0 | 0 | 0.518 | 0.518 |
| AHR | PPARG | 9606.ENSP00000242057 | 9606.ENSP00000287820 | 0 | 0 | 0 | 0 | 0 | 0.057 | 0 | 0.565 | 0.573 |
| AHR | JUN | 9606.ENSP00000242057 | 9606.ENSP00000360266 | 0 | 0 | 0 | 0 | 0 | 0 | 0 | 0.588 | 0.588 |
| AHR | IL1B | 9606.ENSP00000242057 | 9606.ENSP00000263341 | 0 | 0 | 0 | 0 | 0.071 | 0 | 0 | 0.6 | 0.612 |
| AHR | NCOA2 | 9606.ENSP00000242057 | 9606.ENSP00000399968 | 0 | 0 | 0 | 0.546 | 0 | 0.599 | 0 | 0.334 | 0.654 |
| AHR | IL6 | 9606.ENSP00000242057 | 9606.ENSP00000385675 | 0 | 0 | 0 | 0 | 0.069 | 0 | 0 | 0.655 | 0.665 |
| AHR | TNF | 9606.ENSP00000242057 | 9606.ENSP00000398698 | 0 | 0 | 0 | 0 | 0 | 0 | 0 | 0.673 | 0.674 |
| AHR | ESR2 | 9606.ENSP00000242057 | 9606.ENSP00000343925 | 0 | 0 | 0 | 0 | 0 | 0.057 | 0 | 0.668 | 0.674 |
| AHR | CYP3A4 | 9606.ENSP00000242057 | 9606.ENSP00000337915 | 0 | 0 | 0 | 0 | 0.062 | 0 | 0 | 0.677 | 0.684 |
| AHR | NQO1 | 9606.ENSP00000242057 | 9606.ENSP00000319788 | 0 | 0 | 0 | 0 | 0 | 0 | 0 | 0.688 | 0.688 |
| AHR | NCOA1 | 9606.ENSP00000242057 | 9606.ENSP00000385216 | 0 | 0 | 0 | 0.547 | 0.062 | 0.599 | 0 | 0.579 | 0.707 |
| AHR | CYP1A2 | 9606.ENSP00000242057 | 9606.ENSP00000342007 | 0 | 0 | 0 | 0 | 0.062 | 0 | 0 | 0.747 | 0.752 |
| AHR | RB1 | 9606.ENSP00000242057 | 9606.ENSP00000267163 | 0 | 0 | 0 | 0 | 0 | 0.857 | 0 | 0.134 | 0.871 |
| AHR | AR | 9606.ENSP00000242057 | 9606.ENSP00000363822 | 0 | 0 | 0 | 0 | 0 | 0.47 | 0 | 0.833 | 0.908 |
| AHR | ESR1 | 9606.ENSP00000242057 | 9606.ENSP00000405330 | 0 | 0 | 0 | 0 | 0 | 0.873 | 0 | 0.99 | 0.998 |
| AKR1B10 | ADH1B | 9606.ENSP00000352584 | 9606.ENSP00000306606 | 0 | 0 | 0 | 0 | 0.063 | 0.16 | 0 | 0.414 | 0.498 |
| AKR1B10 | NQO1 | 9606.ENSP00000352584 | 9606.ENSP00000319788 | 0.072 | 0 | 0 | 0 | 0.169 | 0 | 0 | 0.427 | 0.52 |
| AKR1B10 | ACACA | 9606.ENSP00000352584 | 9606.ENSP00000483300 | 0 | 0 | 0 | 0 | 0.062 | 0.27 | 0 | 0.452 | 0.592 |
| AKR1B10 | AKR1C3 | 9606.ENSP00000352584 | 9606.ENSP00000369927 | 0 | 0 | 0.449 | 0.933 | 0.102 | 0 | 0.9 | 0.683 | 0.913 |
| AKR1C1 | HMOX1 | 9606.ENSP00000370254 | 9606.ENSP00000216117 | 0 | 0 | 0 | 0 | 0 | 0 | 0 | 0.47 | 0.47 |
| AKR1C1 | GSR | 9606.ENSP00000370254 | 9606.ENSP00000221130 | 0 | 0 | 0 | 0 | 0.062 | 0.057 | 0 | 0.414 | 0.436 |
| AKR1C1 | NQO1 | 9606.ENSP00000370254 | 9606.ENSP00000319788 | 0.072 | 0 | 0 | 0 | 0.163 | 0 | 0 | 0.612 | 0.673 |
| AKR1C1 | CYP3A4 | 9606.ENSP00000370254 | 9606.ENSP00000337915 | 0 | 0 | 0 | 0 | 0.062 | 0 | 0.9 | 0.272 | 0.925 |
| AKR1C1 | ESR2 | 9606.ENSP00000370254 | 9606.ENSP00000343925 | 0 | 0 | 0 | 0 | 0 | 0.056 | 0 | 0.446 | 0.454 |
| AKR1C1 | AKR1C3 | 9606.ENSP00000370254 | 9606.ENSP00000369927 | 0 | 0 | 0.449 | 0.983 | 0.214 | 0.872 | 0.9 | 0.934 | 0.989 |
| AKR1C3 | PPARG | 9606.ENSP00000369927 | 9606.ENSP00000287820 | 0 | 0 | 0 | 0 | 0 | 0.056 | 0 | 0.4 | 0.41 |
| AKR1C3 | NQO1 | 9606.ENSP00000369927 | 9606.ENSP00000319788 | 0.072 | 0 | 0 | 0 | 0.163 | 0 | 0 | 0.469 | 0.552 |
| AKR1C3 | PGR | 9606.ENSP00000369927 | 9606.ENSP00000325120 | 0 | 0 | 0 | 0 | 0 | 0.056 | 0 | 0.392 | 0.401 |
| AKR1C3 | CYP3A4 | 9606.ENSP00000369927 | 9606.ENSP00000337915 | 0 | 0 | 0 | 0 | 0.062 | 0 | 0.9 | 0.336 | 0.932 |
| AKR1C3 | CYP1A2 | 9606.ENSP00000369927 | 9606.ENSP00000342007 | 0 | 0 | 0 | 0 | 0.062 | 0 | 0.9 | 0.198 | 0.918 |
| AKR1C3 | AKR1B10 | 9606.ENSP00000369927 | 9606.ENSP00000352584 | 0 | 0 | 0.449 | 0.933 | 0.102 | 0 | 0.9 | 0.683 | 0.913 |
| AKR1C3 | PTGS1 | 9606.ENSP00000369927 | 9606.ENSP00000354612 | 0 | 0 | 0 | 0 | 0.061 | 0 | 0 | 0.471 | 0.482 |
| AKR1C3 | PTGS2 | 9606.ENSP00000369927 | 9606.ENSP00000356438 | 0 | 0 | 0 | 0 | 0 | 0 | 0 | 0.548 | 0.548 |
| AKR1C3 | AR | 9606.ENSP00000369927 | 9606.ENSP00000363822 | 0 | 0 | 0 | 0 | 0 | 0.056 | 0 | 0.745 | 0.749 |
| AKR1C3 | POR | 9606.ENSP00000369927 | 9606.ENSP00000419970 | 0 | 0 | 0 | 0 | 0 | 0 | 0 | 0.402 | 0.402 |
| AKR1C3 | ESR1 | 9606.ENSP00000369927 | 9606.ENSP00000405330 | 0 | 0 | 0 | 0 | 0 | 0.056 | 0 | 0.407 | 0.416 |
| AKR1C3 | AKR1C1 | 9606.ENSP00000369927 | 9606.ENSP00000370254 | 0 | 0 | 0.449 | 0.983 | 0.214 | 0.872 | 0.9 | 0.934 | 0.989 |
| ALOX5 | HMOX1 | 9606.ENSP00000363512 | 9606.ENSP00000216117 | 0 | 0 | 0 | 0 | 0.06 | 0 | 0 | 0.404 | 0.415 |
| ALOX5 | MMP2 | 9606.ENSP00000363512 | 9606.ENSP00000219070 | 0 | 0 | 0 | 0 | 0 | 0 | 0 | 0.503 | 0.503 |
| ALOX5 | MPO | 9606.ENSP00000363512 | 9606.ENSP00000225275 | 0 | 0 | 0 | 0 | 0.121 | 0 | 0 | 0.478 | 0.522 |
| ALOX5 | CCL2 | 9606.ENSP00000363512 | 9606.ENSP00000225831 | 0 | 0 | 0 | 0 | 0 | 0 | 0 | 0.466 | 0.465 |
| ALOX5 | LTA4H | 9606.ENSP00000363512 | 9606.ENSP00000228740 | 0 | 0 | 0 | 0 | 0 | 0 | 0.9 | 0.79 | 0.978 |
| ALOX5 | CAT | 9606.ENSP00000363512 | 9606.ENSP00000241052 | 0 | 0 | 0 | 0 | 0 | 0 | 0 | 0.411 | 0.411 |
| ALOX5 | IL1B | 9606.ENSP00000363512 | 9606.ENSP00000263341 | 0 | 0 | 0 | 0 | 0.098 | 0 | 0 | 0.606 | 0.629 |
| ALOX5 | TP53 | 9606.ENSP00000363512 | 9606.ENSP00000269305 | 0 | 0 | 0 | 0 | 0 | 0 | 0 | 0.475 | 0.475 |
| ALOX5 | PPARG | 9606.ENSP00000363512 | 9606.ENSP00000287820 | 0 | 0 | 0 | 0 | 0 | 0 | 0 | 0.676 | 0.676 |
| ALOX5 | VCAM1 | 9606.ENSP00000363512 | 9606.ENSP00000294728 | 0 | 0 | 0 | 0 | 0.062 | 0 | 0 | 0.392 | 0.405 |
| ALOX5 | PTGER3 | 9606.ENSP00000363512 | 9606.ENSP00000349003 | 0 | 0 | 0 | 0 | 0 | 0 | 0 | 0.47 | 0.47 |
| ALOX5 | PTGS1 | 9606.ENSP00000363512 | 9606.ENSP00000354612 | 0 | 0 | 0 | 0 | 0.096 | 0 | 0.9 | 0.728 | 0.973 |
| ALOX5 | PTGS2 | 9606.ENSP00000363512 | 9606.ENSP00000356438 | 0 | 0 | 0 | 0 | 0.081 | 0 | 0.9 | 0.828 | 0.982 |
| ALOX5 | VEGFA | 9606.ENSP00000363512 | 9606.ENSP00000478570 | 0 | 0 | 0 | 0 | 0 | 0 | 0 | 0.454 | 0.454 |
| ALOX5 | IL6 | 9606.ENSP00000363512 | 9606.ENSP00000385675 | 0 | 0 | 0 | 0 | 0 | 0 | 0 | 0.601 | 0.601 |
| ALOX5 | TNF | 9606.ENSP00000363512 | 9606.ENSP00000398698 | 0 | 0 | 0 | 0 | 0.121 | 0 | 0 | 0.62 | 0.652 |
| AR | MAPK1 | 9606.ENSP00000363822 | 9606.ENSP00000215832 | 0 | 0 | 0 | 0 | 0 | 0.497 | 0 | 0.324 | 0.645 |
| AR | MMP2 | 9606.ENSP00000363822 | 9606.ENSP00000219070 | 0 | 0 | 0 | 0 | 0.061 | 0 | 0 | 0.403 | 0.415 |
| AR | MAPK14 | 9606.ENSP00000363822 | 9606.ENSP00000229795 | 0 | 0 | 0 | 0 | 0 | 0.104 | 0.9 | 0.289 | 0.93 |
| AR | NR3C1 | 9606.ENSP00000363822 | 9606.ENSP00000231509 | 0 | 0 | 0 | 0.816 | 0 | 0.213 | 0.9 | 0.834 | 0.93 |
| AR | ODC1 | 9606.ENSP00000363822 | 9606.ENSP00000234111 | 0 | 0 | 0 | 0 | 0 | 0 | 0 | 0.474 | 0.474 |
| AR | AHR | 9606.ENSP00000363822 | 9606.ENSP00000242057 | 0 | 0 | 0 | 0 | 0 | 0.47 | 0 | 0.833 | 0.908 |
| AR | CDK4 | 9606.ENSP00000363822 | 9606.ENSP00000257904 | 0 | 0 | 0 | 0 | 0 | 0.057 | 0 | 0.523 | 0.53 |
| AR | MAPK3 | 9606.ENSP00000363822 | 9606.ENSP00000263025 | 0 | 0 | 0 | 0 | 0 | 0.104 | 0 | 0.517 | 0.548 |
| AR | EGF | 9606.ENSP00000363822 | 9606.ENSP00000265171 | 0 | 0 | 0 | 0 | 0 | 0.056 | 0 | 0.72 | 0.724 |
| AR | CDK2 | 9606.ENSP00000363822 | 9606.ENSP00000266970 | 0 | 0 | 0 | 0 | 0 | 0.057 | 0 | 0.531 | 0.539 |
| AR | RB1 | 9606.ENSP00000363822 | 9606.ENSP00000267163 | 0 | 0 | 0 | 0 | 0 | 0.687 | 0.65 | 0.489 | 0.939 |
| AR | TP53 | 9606.ENSP00000363822 | 9606.ENSP00000269305 | 0 | 0 | 0 | 0 | 0 | 0.474 | 0 | 0.776 | 0.877 |
| AR | CCNA2 | 9606.ENSP00000363822 | 9606.ENSP00000274026 | 0 | 0 | 0 | 0 | 0 | 0.056 | 0 | 0.577 | 0.583 |
| AR | EGFR | 9606.ENSP00000363822 | 9606.ENSP00000275493 | 0 | 0 | 0 | 0 | 0.063 | 0.698 | 0 | 0.966 | 0.989 |
| AR | KCNMA1 | 9606.ENSP00000363822 | 9606.ENSP00000286628 | 0 | 0 | 0 | 0 | 0 | 0 | 0 | 0.422 | 0.422 |
| AR | FASN | 9606.ENSP00000363822 | 9606.ENSP00000304592 | 0 | 0 | 0 | 0 | 0 | 0 | 0 | 0.42 | 0.42 |
| AR | PRKACA | 9606.ENSP00000363822 | 9606.ENSP00000309591 | 0 | 0 | 0 | 0 | 0 | 0 | 0.9 | 0.094 | 0.905 |
| AR | CAMKK2 | 9606.ENSP00000363822 | 9606.ENSP00000312741 | 0 | 0 | 0 | 0 | 0 | 0.282 | 0 | 0.415 | 0.563 |
| AR | HSPA5 | 9606.ENSP00000363822 | 9606.ENSP00000324173 | 0 | 0 | 0 | 0 | 0 | 0.182 | 0 | 0.301 | 0.403 |
| AR | GSK3B | 9606.ENSP00000363822 | 9606.ENSP00000324806 | 0 | 0 | 0 | 0 | 0 | 0.482 | 0.9 | 0.405 | 0.966 |
| AR | PGR | 9606.ENSP00000363822 | 9606.ENSP00000325120 | 0 | 0 | 0 | 0.813 | 0.069 | 0 | 0.6 | 0.908 | 0.677 |
| AR | CYP3A4 | 9606.ENSP00000363822 | 9606.ENSP00000337915 | 0 | 0 | 0 | 0 | 0 | 0.073 | 0 | 0.455 | 0.473 |
| AR | NR3C2 | 9606.ENSP00000363822 | 9606.ENSP00000350815 | 0 | 0 | 0 | 0.779 | 0.062 | 0.213 | 0.6 | 0.705 | 0.728 |
| AR | TOP1 | 9606.ENSP00000363822 | 9606.ENSP00000354522 | 0 | 0 | 0 | 0 | 0.062 | 0 | 0 | 0.436 | 0.448 |
| AR | PTGS2 | 9606.ENSP00000363822 | 9606.ENSP00000356438 | 0 | 0 | 0 | 0 | 0 | 0.056 | 0 | 0.412 | 0.421 |
| AR | JUN | 9606.ENSP00000363822 | 9606.ENSP00000360266 | 0 | 0 | 0 | 0 | 0 | 0.499 | 0 | 0.928 | 0.962 |
| AR | GSTP1 | 9606.ENSP00000363822 | 9606.ENSP00000381607 | 0 | 0 | 0 | 0 | 0 | 0.062 | 0 | 0.456 | 0.467 |
| AR | TNF | 9606.ENSP00000363822 | 9606.ENSP00000398698 | 0 | 0 | 0 | 0 | 0 | 0 | 0 | 0.469 | 0.469 |
| AR | ESR1 | 9606.ENSP00000363822 | 9606.ENSP00000405330 | 0 | 0 | 0 | 0.656 | 0.062 | 0.213 | 0 | 0.984 | 0.49 |
| AR | IL6 | 9606.ENSP00000363822 | 9606.ENSP00000385675 | 0 | 0 | 0 | 0 | 0 | 0 | 0 | 0.56 | 0.56 |
| AR | VEGFA | 9606.ENSP00000363822 | 9606.ENSP00000478570 | 0 | 0 | 0 | 0 | 0 | 0 | 0 | 0.6 | 0.6 |
| AR | AKR1C3 | 9606.ENSP00000363822 | 9606.ENSP00000369927 | 0 | 0 | 0 | 0 | 0 | 0.056 | 0 | 0.745 | 0.749 |
| AR | CDK1 | 9606.ENSP00000363822 | 9606.ENSP00000378699 | 0 | 0 | 0 | 0 | 0 | 0.226 | 0 | 0.819 | 0.854 |
| AR | MAPK8 | 9606.ENSP00000363822 | 9606.ENSP00000378974 | 0 | 0 | 0 | 0 | 0 | 0 | 0.9 | 0.285 | 0.925 |
| AR | NCOA2 | 9606.ENSP00000363822 | 9606.ENSP00000399968 | 0 | 0 | 0 | 0 | 0 | 0.977 | 0.9 | 0.954 | 0.999 |
| AR | NCOA1 | 9606.ENSP00000363822 | 9606.ENSP00000385216 | 0 | 0 | 0 | 0 | 0 | 0.895 | 0.9 | 0.964 | 0.999 |
| BAD | MAPK1 | 9606.ENSP00000378040 | 9606.ENSP00000215832 | 0 | 0 | 0 | 0 | 0.062 | 0 | 0.9 | 0.169 | 0.915 |
| BAD | MAPK3 | 9606.ENSP00000378040 | 9606.ENSP00000263025 | 0 | 0 | 0 | 0 | 0.062 | 0 | 0.9 | 0.298 | 0.928 |
| BAD | TP53 | 9606.ENSP00000378040 | 9606.ENSP00000269305 | 0 | 0 | 0 | 0 | 0 | 0 | 0 | 0.64 | 0.64 |
| BAD | BAX | 9606.ENSP00000378040 | 9606.ENSP00000293288 | 0 | 0 | 0 | 0 | 0 | 0.535 | 0 | 0.681 | 0.845 |
| BAD | PRKACA | 9606.ENSP00000378040 | 9606.ENSP00000309591 | 0 | 0 | 0 | 0 | 0 | 0.729 | 0.9 | 0.232 | 0.977 |
| BAD | MAPK10 | 9606.ENSP00000378040 | 9606.ENSP00000352157 | 0 | 0 | 0 | 0 | 0 | 0 | 0.9 | 0.093 | 0.905 |
| BAD | PIM1 | 9606.ENSP00000378040 | 9606.ENSP00000362608 | 0 | 0 | 0 | 0 | 0 | 0.463 | 0 | 0.292 | 0.603 |
| BAD | PPP3CA | 9606.ENSP00000378040 | 9606.ENSP00000378323 | 0 | 0 | 0 | 0 | 0 | 0.213 | 0.8 | 0 | 0.835 |
| BAD | MAPK8 | 9606.ENSP00000378040 | 9606.ENSP00000378974 | 0 | 0 | 0 | 0 | 0 | 0.682 | 0.9 | 0.244 | 0.973 |
| BAD | BCL2 | 9606.ENSP00000378040 | 9606.ENSP00000381185 | 0 | 0 | 0 | 0 | 0 | 0.905 | 0.9 | 0.559 | 0.995 |
| BAX | HMOX1 | 9606.ENSP00000293288 | 9606.ENSP00000216117 | 0 | 0 | 0 | 0 | 0.063 | 0 | 0 | 0.503 | 0.514 |
| BAX | CAT | 9606.ENSP00000293288 | 9606.ENSP00000241052 | 0 | 0 | 0 | 0 | 0 | 0 | 0 | 0.518 | 0.518 |
| BAX | IL1B | 9606.ENSP00000293288 | 9606.ENSP00000263341 | 0 | 0 | 0 | 0 | 0.049 | 0 | 0 | 0.465 | 0.469 |
| BAX | CDK2 | 9606.ENSP00000293288 | 9606.ENSP00000266970 | 0 | 0 | 0 | 0 | 0 | 0.056 | 0 | 0.391 | 0.4 |
| BAX | TP53 | 9606.ENSP00000293288 | 9606.ENSP00000269305 | 0 | 0 | 0 | 0 | 0.051 | 0.738 | 0.9 | 0.681 | 0.991 |
| BAX | PTGS2 | 9606.ENSP00000293288 | 9606.ENSP00000356438 | 0 | 0 | 0 | 0 | 0 | 0 | 0 | 0.458 | 0.457 |
| BAX | IL6 | 9606.ENSP00000293288 | 9606.ENSP00000385675 | 0 | 0 | 0 | 0 | 0 | 0 | 0 | 0.459 | 0.459 |
| BAX | HSPA5 | 9606.ENSP00000293288 | 9606.ENSP00000324173 | 0 | 0 | 0 | 0 | 0 | 0 | 0 | 0.459 | 0.459 |
| BAX | VEGFA | 9606.ENSP00000293288 | 9606.ENSP00000478570 | 0 | 0 | 0 | 0 | 0 | 0 | 0 | 0.467 | 0.467 |
| BAX | TNF | 9606.ENSP00000293288 | 9606.ENSP00000398698 | 0 | 0 | 0 | 0 | 0 | 0.059 | 0 | 0.457 | 0.467 |
| BAX | GSK3B | 9606.ENSP00000293288 | 9606.ENSP00000324806 | 0 | 0 | 0 | 0 | 0.059 | 0.27 | 0 | 0.301 | 0.477 |
| BAX | JUN | 9606.ENSP00000293288 | 9606.ENSP00000360266 | 0 | 0 | 0 | 0 | 0 | 0 | 0 | 0.558 | 0.558 |
| BAX | PRKACA | 9606.ENSP00000293288 | 9606.ENSP00000309591 | 0 | 0 | 0 | 0 | 0.045 | 0 | 0.8 | 0 | 0.8 |
| BAX | BAD | 9606.ENSP00000293288 | 9606.ENSP00000378040 | 0 | 0 | 0 | 0 | 0 | 0.535 | 0 | 0.681 | 0.845 |
| BAX | MAPK8 | 9606.ENSP00000293288 | 9606.ENSP00000378974 | 0 | 0 | 0 | 0 | 0 | 0.281 | 0.9 | 0.394 | 0.952 |
| BAX | BCL2 | 9606.ENSP00000293288 | 9606.ENSP00000381185 | 0 | 0 | 0 | 0.645 | 0 | 0.981 | 0.9 | 0.758 | 0.998 |
| BCL2 | MAPK1 | 9606.ENSP00000381185 | 9606.ENSP00000215832 | 0 | 0 | 0 | 0 | 0 | 0.462 | 0.9 | 0.222 | 0.954 |
| BCL2 | CTSD | 9606.ENSP00000381185 | 9606.ENSP00000236671 | 0 | 0 | 0 | 0 | 0 | 0 | 0.6 | 0.061 | 0.608 |
| BCL2 | MAPK3 | 9606.ENSP00000381185 | 9606.ENSP00000263025 | 0 | 0 | 0 | 0 | 0 | 0.213 | 0.9 | 0.297 | 0.939 |
| BCL2 | CDK2 | 9606.ENSP00000381185 | 9606.ENSP00000266970 | 0 | 0 | 0 | 0 | 0 | 0.329 | 0 | 0.263 | 0.484 |
| BCL2 | TP53 | 9606.ENSP00000381185 | 9606.ENSP00000269305 | 0 | 0 | 0 | 0 | 0.051 | 0.884 | 0.9 | 0.628 | 0.995 |
| BCL2 | SOD1 | 9606.ENSP00000381185 | 9606.ENSP00000270142 | 0 | 0 | 0 | 0 | 0 | 0.297 | 0.8 | 0.15 | 0.87 |
| BCL2 | BAX | 9606.ENSP00000381185 | 9606.ENSP00000293288 | 0 | 0 | 0 | 0.645 | 0 | 0.981 | 0.9 | 0.758 | 0.998 |
| BCL2 | CAMKK2 | 9606.ENSP00000381185 | 9606.ENSP00000312741 | 0 | 0 | 0 | 0 | 0 | 0 | 0 | 0.431 | 0.431 |
| BCL2 | MAPK10 | 9606.ENSP00000381185 | 9606.ENSP00000352157 | 0 | 0 | 0 | 0 | 0 | 0.056 | 0.8 | 0.083 | 0.811 |
| BCL2 | RXRB | 9606.ENSP00000381185 | 9606.ENSP00000363817 | 0 | 0 | 0 | 0 | 0.062 | 0.057 | 0.9 | 0 | 0.903 |
| BCL2 | BAD | 9606.ENSP00000381185 | 9606.ENSP00000378040 | 0 | 0 | 0 | 0 | 0 | 0.905 | 0.9 | 0.559 | 0.995 |
| BCL2 | PPP3CA | 9606.ENSP00000381185 | 9606.ENSP00000378323 | 0 | 0 | 0 | 0 | 0 | 0.687 | 0 | 0.073 | 0.698 |
| BCL2 | CDK1 | 9606.ENSP00000381185 | 9606.ENSP00000378699 | 0 | 0 | 0 | 0 | 0 | 0.68 | 0 | 0.146 | 0.715 |
| BCL2 | MAPK8 | 9606.ENSP00000381185 | 9606.ENSP00000378974 | 0 | 0 | 0 | 0 | 0 | 0.471 | 0.9 | 0.258 | 0.957 |
| BCL2 | RXRA | 9606.ENSP00000381185 | 9606.ENSP00000419692 | 0 | 0 | 0 | 0 | 0 | 0.057 | 0.9 | 0 | 0.901 |
| BCL2 | ESR1 | 9606.ENSP00000381185 | 9606.ENSP00000405330 | 0 | 0 | 0 | 0 | 0.069 | 0.057 | 0.9 | 0.244 | 0.924 |
| CA2 | JUN | 9606.ENSP00000285379 | 9606.ENSP00000360266 | 0 | 0 | 0 | 0 | 0 | 0.129 | 0 | 0.413 | 0.467 |
| CAMKK2 | MAPK14 | 9606.ENSP00000312741 | 9606.ENSP00000229795 | 0 | 0 | 0 | 0.63 | 0.063 | 0.396 | 0 | 0.195 | 0.445 |
| CAMKK2 | NOS3 | 9606.ENSP00000312741 | 9606.ENSP00000297494 | 0 | 0 | 0 | 0 | 0 | 0.214 | 0 | 0.402 | 0.509 |
| CAMKK2 | ACACA | 9606.ENSP00000312741 | 9606.ENSP00000483300 | 0 | 0 | 0 | 0 | 0 | 0 | 0 | 0.424 | 0.424 |
| CAMKK2 | BCL2 | 9606.ENSP00000312741 | 9606.ENSP00000381185 | 0 | 0 | 0 | 0 | 0 | 0 | 0 | 0.431 | 0.431 |
| CAMKK2 | AR | 9606.ENSP00000312741 | 9606.ENSP00000363822 | 0 | 0 | 0 | 0 | 0 | 0.282 | 0 | 0.415 | 0.563 |
| CAT | MAPK1 | 9606.ENSP00000241052 | 9606.ENSP00000215832 | 0 | 0 | 0 | 0 | 0 | 0.1 | 0 | 0.455 | 0.488 |
| CAT | HMOX1 | 9606.ENSP00000241052 | 9606.ENSP00000216117 | 0 | 0 | 0 | 0 | 0.062 | 0.101 | 0 | 0.882 | 0.892 |
| CAT | MMP2 | 9606.ENSP00000241052 | 9606.ENSP00000219070 | 0 | 0 | 0 | 0 | 0 | 0 | 0 | 0.46 | 0.459 |
| CAT | GSR | 9606.ENSP00000241052 | 9606.ENSP00000221130 | 0 | 0 | 0 | 0 | 0.218 | 0.26 | 0 | 0.953 | 0.97 |
| CAT | PON1 | 9606.ENSP00000241052 | 9606.ENSP00000222381 | 0 | 0 | 0 | 0 | 0.062 | 0 | 0 | 0.596 | 0.604 |
| CAT | MPO | 9606.ENSP00000241052 | 9606.ENSP00000225275 | 0 | 0 | 0 | 0 | 0.061 | 0.241 | 0 | 0.702 | 0.769 |
| CAT | CCL2 | 9606.ENSP00000241052 | 9606.ENSP00000225831 | 0 | 0 | 0 | 0 | 0 | 0 | 0 | 0.564 | 0.564 |
| CAT | IL2 | 9606.ENSP00000241052 | 9606.ENSP00000226730 | 0 | 0 | 0 | 0 | 0 | 0 | 0 | 0.467 | 0.467 |
| CAT | IFNG | 9606.ENSP00000241052 | 9606.ENSP00000229135 | 0 | 0 | 0 | 0 | 0 | 0 | 0 | 0.514 | 0.514 |
| CAT | MAPK14 | 9606.ENSP00000241052 | 9606.ENSP00000229795 | 0 | 0 | 0 | 0 | 0 | 0.117 | 0 | 0.507 | 0.546 |
| CAT | ODC1 | 9606.ENSP00000241052 | 9606.ENSP00000234111 | 0.043 | 0 | 0 | 0 | 0 | 0 | 0 | 0.481 | 0.482 |
| CAT | CTSD | 9606.ENSP00000241052 | 9606.ENSP00000236671 | 0 | 0 | 0 | 0 | 0.086 | 0.138 | 0 | 0.381 | 0.47 |
| CAT | ADH1B | 9606.ENSP00000241052 | 9606.ENSP00000306606 | 0 | 0 | 0 | 0 | 0.062 | 0 | 0 | 0.387 | 0.4 |
| CAT | CYP1A2 | 9606.ENSP00000241052 | 9606.ENSP00000342007 | 0.047 | 0 | 0 | 0 | 0 | 0 | 0 | 0.398 | 0.401 |
| CAT | AHR | 9606.ENSP00000241052 | 9606.ENSP00000242057 | 0 | 0 | 0 | 0 | 0.062 | 0 | 0 | 0.391 | 0.404 |
| CAT | ALOX5 | 9606.ENSP00000241052 | 9606.ENSP00000363512 | 0 | 0 | 0 | 0 | 0 | 0 | 0 | 0.411 | 0.411 |
| CAT | FASN | 9606.ENSP00000241052 | 9606.ENSP00000304592 | 0 | 0 | 0 | 0 | 0 | 0.143 | 0 | 0.346 | 0.415 |
| CAT | SELE | 9606.ENSP00000241052 | 9606.ENSP00000331736 | 0 | 0 | 0 | 0 | 0.049 | 0 | 0 | 0.41 | 0.415 |
| CAT | CYP3A4 | 9606.ENSP00000241052 | 9606.ENSP00000337915 | 0.047 | 0 | 0 | 0 | 0.049 | 0 | 0 | 0.43 | 0.438 |
| CAT | EGFR | 9606.ENSP00000241052 | 9606.ENSP00000275493 | 0 | 0 | 0 | 0 | 0.062 | 0 | 0 | 0.431 | 0.443 |
| CAT | PTGS1 | 9606.ENSP00000241052 | 9606.ENSP00000354612 | 0 | 0 | 0 | 0 | 0.065 | 0.178 | 0 | 0.342 | 0.45 |
| CAT | POR | 9606.ENSP00000241052 | 9606.ENSP00000419970 | 0 | 0 | 0 | 0 | 0.049 | 0 | 0 | 0.452 | 0.457 |
| CAT | MAOA | 9606.ENSP00000241052 | 9606.ENSP00000340684 | 0 | 0 | 0 | 0 | 0.062 | 0 | 0 | 0.446 | 0.459 |
| CAT | EGF | 9606.ENSP00000241052 | 9606.ENSP00000265171 | 0 | 0 | 0 | 0 | 0 | 0 | 0 | 0.465 | 0.465 |
| CAT | PTPN1 | 9606.ENSP00000241052 | 9606.ENSP00000360683 | 0 | 0 | 0 | 0 | 0.053 | 0 | 0 | 0.468 | 0.475 |
| CAT | MAPK8 | 9606.ENSP00000241052 | 9606.ENSP00000378974 | 0 | 0 | 0 | 0 | 0 | 0 | 0 | 0.501 | 0.501 |
| CAT | GSK3B | 9606.ENSP00000241052 | 9606.ENSP00000324806 | 0.044 | 0 | 0 | 0 | 0.062 | 0.144 | 0 | 0.429 | 0.504 |
| CAT | MAOB | 9606.ENSP00000241052 | 9606.ENSP00000367309 | 0 | 0 | 0 | 0 | 0.062 | 0 | 0 | 0.503 | 0.514 |
| CAT | BAX | 9606.ENSP00000241052 | 9606.ENSP00000293288 | 0 | 0 | 0 | 0 | 0 | 0 | 0 | 0.518 | 0.518 |
| CAT | VCAM1 | 9606.ENSP00000241052 | 9606.ENSP00000294728 | 0 | 0 | 0 | 0 | 0 | 0 | 0 | 0.522 | 0.522 |
| CAT | ESR1 | 9606.ENSP00000241052 | 9606.ENSP00000405330 | 0 | 0 | 0 | 0 | 0.062 | 0.27 | 0 | 0.4 | 0.553 |
| CAT | VEGFA | 9606.ENSP00000241052 | 9606.ENSP00000478570 | 0 | 0 | 0 | 0 | 0 | 0 | 0 | 0.601 | 0.601 |
| CAT | GSTP1 | 9606.ENSP00000241052 | 9606.ENSP00000381607 | 0 | 0 | 0 | 0 | 0.053 | 0.178 | 0 | 0.535 | 0.606 |
| CAT | HSPA5 | 9606.ENSP00000241052 | 9606.ENSP00000324173 | 0 | 0 | 0 | 0 | 0.062 | 0 | 0 | 0.618 | 0.626 |
| CAT | PPARG | 9606.ENSP00000241052 | 9606.ENSP00000287820 | 0 | 0 | 0 | 0 | 0 | 0 | 0 | 0.655 | 0.655 |
| CAT | JUN | 9606.ENSP00000241052 | 9606.ENSP00000360266 | 0 | 0 | 0 | 0 | 0 | 0 | 0 | 0.66 | 0.66 |
| CAT | ACHE | 9606.ENSP00000241052 | 9606.ENSP00000303211 | 0 | 0 | 0 | 0 | 0.049 | 0 | 0 | 0.674 | 0.677 |
| CAT | TP53 | 9606.ENSP00000241052 | 9606.ENSP00000269305 | 0 | 0 | 0 | 0 | 0 | 0 | 0 | 0.689 | 0.689 |
| CAT | MAPK3 | 9606.ENSP00000241052 | 9606.ENSP00000263025 | 0 | 0 | 0 | 0 | 0 | 0.1 | 0 | 0.673 | 0.693 |
| CAT | XDH | 9606.ENSP00000241052 | 9606.ENSP00000368727 | 0 | 0 | 0 | 0 | 0.062 | 0 | 0 | 0.697 | 0.703 |
| CAT | PTGS2 | 9606.ENSP00000241052 | 9606.ENSP00000356438 | 0 | 0 | 0 | 0 | 0 | 0.178 | 0 | 0.675 | 0.721 |
| CAT | IL1B | 9606.ENSP00000241052 | 9606.ENSP00000263341 | 0 | 0 | 0 | 0 | 0 | 0 | 0 | 0.734 | 0.734 |
| CAT | NOS3 | 9606.ENSP00000241052 | 9606.ENSP00000297494 | 0 | 0 | 0 | 0 | 0.049 | 0 | 0 | 0.744 | 0.746 |
| CAT | IL6 | 9606.ENSP00000241052 | 9606.ENSP00000385675 | 0 | 0 | 0 | 0 | 0 | 0 | 0 | 0.756 | 0.756 |
| CAT | NQO1 | 9606.ENSP00000241052 | 9606.ENSP00000319788 | 0 | 0 | 0 | 0 | 0.062 | 0 | 0 | 0.755 | 0.76 |
| CAT | TNF | 9606.ENSP00000241052 | 9606.ENSP00000398698 | 0 | 0 | 0 | 0 | 0 | 0 | 0 | 0.779 | 0.779 |
| CAT | NOS2 | 9606.ENSP00000241052 | 9606.ENSP00000327251 | 0 | 0 | 0 | 0 | 0.049 | 0 | 0.9 | 0.558 | 0.954 |
| CAT | SOD1 | 9606.ENSP00000241052 | 9606.ENSP00000270142 | 0.111 | 0 | 0 | 0 | 0.124 | 0.52 | 0.9 | 0.923 | 0.996 |
| CCL2 | HMOX1 | 9606.ENSP00000225831 | 9606.ENSP00000216117 | 0 | 0 | 0 | 0 | 0.095 | 0 | 0 | 0.668 | 0.686 |
| CCL2 | MMP2 | 9606.ENSP00000225831 | 9606.ENSP00000219070 | 0 | 0 | 0 | 0 | 0.155 | 0 | 0 | 0.667 | 0.706 |
| CCL2 | PLAT | 9606.ENSP00000225831 | 9606.ENSP00000220809 | 0 | 0 | 0 | 0 | 0.073 | 0 | 0 | 0.415 | 0.434 |
| CCL2 | PON1 | 9606.ENSP00000225831 | 9606.ENSP00000222381 | 0 | 0 | 0 | 0 | 0 | 0 | 0 | 0.452 | 0.452 |
| CCL2 | MPO | 9606.ENSP00000225831 | 9606.ENSP00000225275 | 0 | 0 | 0 | 0 | 0 | 0 | 0 | 0.682 | 0.682 |
| CCL2 | NR3C1 | 9606.ENSP00000225831 | 9606.ENSP00000231509 | 0 | 0 | 0 | 0 | 0.055 | 0 | 0 | 0.406 | 0.414 |
| CCL2 | F2 | 9606.ENSP00000225831 | 9606.ENSP00000308541 | 0 | 0 | 0 | 0 | 0 | 0 | 0 | 0.416 | 0.416 |
| CCL2 | PTGS1 | 9606.ENSP00000225831 | 9606.ENSP00000354612 | 0 | 0 | 0 | 0 | 0.097 | 0 | 0 | 0.389 | 0.424 |
| CCL2 | SOD1 | 9606.ENSP00000225831 | 9606.ENSP00000270142 | 0 | 0 | 0 | 0 | 0 | 0 | 0 | 0.427 | 0.426 |
| CCL2 | GJA1 | 9606.ENSP00000225831 | 9606.ENSP00000282561 | 0 | 0 | 0 | 0 | 0.086 | 0 | 0 | 0.413 | 0.44 |
| CCL2 | MAPK14 | 9606.ENSP00000225831 | 9606.ENSP00000229795 | 0 | 0 | 0 | 0 | 0 | 0 | 0 | 0.465 | 0.465 |
| CCL2 | ALOX5 | 9606.ENSP00000225831 | 9606.ENSP00000363512 | 0 | 0 | 0 | 0 | 0 | 0 | 0 | 0.466 | 0.465 |
| CCL2 | ESR1 | 9606.ENSP00000225831 | 9606.ENSP00000405330 | 0 | 0 | 0 | 0 | 0.055 | 0 | 0 | 0.46 | 0.467 |
| CCL2 | DPP4 | 9606.ENSP00000225831 | 9606.ENSP00000353731 | 0 | 0 | 0 | 0 | 0 | 0 | 0 | 0.495 | 0.495 |
| CCL2 | LDLR | 9606.ENSP00000225831 | 9606.ENSP00000454071 | 0 | 0 | 0 | 0 | 0 | 0 | 0 | 0.517 | 0.517 |
| CCL2 | MAPK8 | 9606.ENSP00000225831 | 9606.ENSP00000378974 | 0 | 0 | 0 | 0 | 0 | 0 | 0 | 0.518 | 0.518 |
| CCL2 | PLAU | 9606.ENSP00000225831 | 9606.ENSP00000361850 | 0 | 0 | 0 | 0 | 0.122 | 0 | 0 | 0.475 | 0.519 |
| CCL2 | EGFR | 9606.ENSP00000225831 | 9606.ENSP00000275493 | 0 | 0 | 0 | 0 | 0.062 | 0 | 0 | 0.517 | 0.527 |
| CCL2 | THBD | 9606.ENSP00000225831 | 9606.ENSP00000366307 | 0 | 0 | 0 | 0 | 0.095 | 0 | 0 | 0.504 | 0.532 |
| CCL2 | KDR | 9606.ENSP00000225831 | 9606.ENSP00000263923 | 0 | 0 | 0 | 0 | 0.065 | 0 | 0 | 0.52 | 0.532 |
| CCL2 | CAT | 9606.ENSP00000225831 | 9606.ENSP00000241052 | 0 | 0 | 0 | 0 | 0 | 0 | 0 | 0.564 | 0.564 |
| CCL2 | NOS2 | 9606.ENSP00000225831 | 9606.ENSP00000327251 | 0 | 0 | 0 | 0 | 0 | 0 | 0 | 0.624 | 0.624 |
| CCL2 | MAPK3 | 9606.ENSP00000225831 | 9606.ENSP00000263025 | 0 | 0 | 0 | 0 | 0 | 0 | 0 | 0.634 | 0.634 |
| CCL2 | TP53 | 9606.ENSP00000225831 | 9606.ENSP00000269305 | 0 | 0 | 0 | 0 | 0 | 0 | 0 | 0.644 | 0.644 |
| CCL2 | F3 | 9606.ENSP00000225831 | 9606.ENSP00000334145 | 0 | 0 | 0 | 0 | 0.083 | 0 | 0 | 0.66 | 0.675 |
| CCL2 | EGF | 9606.ENSP00000225831 | 9606.ENSP00000265171 | 0 | 0 | 0 | 0 | 0 | 0 | 0 | 0.681 | 0.681 |
| CCL2 | NOS3 | 9606.ENSP00000225831 | 9606.ENSP00000297494 | 0 | 0 | 0 | 0 | 0 | 0 | 0 | 0.681 | 0.681 |
| CCL2 | MMP1 | 9606.ENSP00000225831 | 9606.ENSP00000322788 | 0 | 0 | 0 | 0 | 0.109 | 0.213 | 0 | 0.614 | 0.705 |
| CCL2 | PPARG | 9606.ENSP00000225831 | 9606.ENSP00000287820 | 0 | 0 | 0 | 0 | 0.055 | 0 | 0 | 0.705 | 0.709 |
| CCL2 | PTGS2 | 9606.ENSP00000225831 | 9606.ENSP00000356438 | 0 | 0 | 0 | 0 | 0.117 | 0 | 0 | 0.754 | 0.774 |
| CCL2 | SELE | 9606.ENSP00000225831 | 9606.ENSP00000331736 | 0 | 0 | 0 | 0 | 0.109 | 0 | 0 | 0.767 | 0.784 |
| CCL2 | MMP3 | 9606.ENSP00000225831 | 9606.ENSP00000299855 | 0 | 0 | 0 | 0 | 0.107 | 0.213 | 0 | 0.731 | 0.795 |
| CCL2 | IL2 | 9606.ENSP00000225831 | 9606.ENSP00000226730 | 0 | 0 | 0 | 0 | 0 | 0 | 0 | 0.81 | 0.81 |
| CCL2 | IFNG | 9606.ENSP00000225831 | 9606.ENSP00000229135 | 0 | 0 | 0 | 0 | 0.063 | 0 | 0 | 0.835 | 0.839 |
| CCL2 | VEGFA | 9606.ENSP00000225831 | 9606.ENSP00000478570 | 0 | 0 | 0 | 0 | 0 | 0 | 0 | 0.856 | 0.856 |
| CCL2 | VCAM1 | 9606.ENSP00000225831 | 9606.ENSP00000294728 | 0 | 0 | 0 | 0 | 0.202 | 0 | 0 | 0.879 | 0.899 |
| CCL2 | JUN | 9606.ENSP00000225831 | 9606.ENSP00000360266 | 0 | 0 | 0 | 0 | 0.062 | 0 | 0.9 | 0.686 | 0.968 |
| CCL2 | TNF | 9606.ENSP00000225831 | 9606.ENSP00000398698 | 0 | 0 | 0 | 0 | 0.062 | 0 | 0.9 | 0.93 | 0.992 |
| CCL2 | IL1B | 9606.ENSP00000225831 | 9606.ENSP00000263341 | 0 | 0 | 0 | 0 | 0.127 | 0 | 0.9 | 0.927 | 0.993 |
| CCL2 | IL6 | 9606.ENSP00000225831 | 9606.ENSP00000385675 | 0 | 0 | 0 | 0 | 0.267 | 0 | 0.9 | 0.93 | 0.994 |
| CCNA2 | MAPK1 | 9606.ENSP00000274026 | 9606.ENSP00000215832 | 0 | 0.002 | 0 | 0 | 0.064 | 0.07 | 0 | 0.375 | 0.408 |
| CCNA2 | CDK4 | 9606.ENSP00000274026 | 9606.ENSP00000257904 | 0 | 0.003 | 0 | 0 | 0.183 | 0.587 | 0.9 | 0.946 | 0.997 |
| CCNA2 | MAPK3 | 9606.ENSP00000274026 | 9606.ENSP00000263025 | 0 | 0.001 | 0 | 0 | 0.064 | 0.07 | 0 | 0.526 | 0.551 |
| CCNA2 | EGF | 9606.ENSP00000274026 | 9606.ENSP00000265171 | 0 | 0 | 0 | 0 | 0.062 | 0 | 0 | 0.502 | 0.512 |
| CCNA2 | CDK2 | 9606.ENSP00000274026 | 9606.ENSP00000266970 | 0 | 0.006 | 0 | 0 | 0.401 | 0.999 | 0.9 | 0.995 | 0.999 |
| CCNA2 | RB1 | 9606.ENSP00000274026 | 9606.ENSP00000267163 | 0 | 0 | 0 | 0 | 0.108 | 0.885 | 0.9 | 0.733 | 0.996 |
| CCNA2 | TP53 | 9606.ENSP00000274026 | 9606.ENSP00000269305 | 0 | 0 | 0 | 0 | 0.092 | 0.283 | 0.9 | 0.944 | 0.995 |
| CCNA2 | TOP1 | 9606.ENSP00000274026 | 9606.ENSP00000354522 | 0 | 0 | 0 | 0 | 0.086 | 0.061 | 0 | 0.39 | 0.431 |
| CCNA2 | VEGFA | 9606.ENSP00000274026 | 9606.ENSP00000478570 | 0 | 0 | 0 | 0 | 0 | 0 | 0 | 0.47 | 0.47 |
| CCNA2 | PGR | 9606.ENSP00000274026 | 9606.ENSP00000325120 | 0 | 0 | 0 | 0 | 0 | 0.225 | 0 | 0.405 | 0.519 |
| CCNA2 | JUN | 9606.ENSP00000274026 | 9606.ENSP00000360266 | 0 | 0 | 0 | 0 | 0 | 0 | 0 | 0.559 | 0.559 |
| CCNA2 | ESR1 | 9606.ENSP00000274026 | 9606.ENSP00000405330 | 0 | 0 | 0 | 0 | 0 | 0.056 | 0 | 0.558 | 0.564 |
| CCNA2 | AR | 9606.ENSP00000274026 | 9606.ENSP00000363822 | 0 | 0 | 0 | 0 | 0 | 0.056 | 0 | 0.577 | 0.583 |
| CCNA2 | EGFR | 9606.ENSP00000274026 | 9606.ENSP00000275493 | 0 | 0 | 0 | 0 | 0 | 0.103 | 0 | 0.566 | 0.594 |
| CCNA2 | CHEK1 | 9606.ENSP00000274026 | 9606.ENSP00000388648 | 0 | 0 | 0 | 0 | 0.852 | 0.058 | 0 | 0.702 | 0.955 |
| CCNA2 | TOP2A | 9606.ENSP00000274026 | 9606.ENSP00000411532 | 0 | 0 | 0 | 0 | 0.967 | 0.056 | 0 | 0.575 | 0.985 |
| CCNA2 | CDK1 | 9606.ENSP00000274026 | 9606.ENSP00000378699 | 0 | 0.007 | 0 | 0 | 0.985 | 0.929 | 0.9 | 0.992 | 0.999 |
| CDK1 | CDK4 | 9606.ENSP00000378699 | 9606.ENSP00000257904 | 0 | 0 | 0.436 | 0.905 | 0.213 | 0.119 | 0.9 | 0.913 | 0.933 |
| CDK1 | EGF | 9606.ENSP00000378699 | 9606.ENSP00000265171 | 0 | 0 | 0 | 0 | 0.062 | 0 | 0 | 0.46 | 0.471 |
| CDK1 | CDK2 | 9606.ENSP00000378699 | 9606.ENSP00000266970 | 0 | 0 | 0.441 | 0.968 | 0.68 | 0.617 | 0.9 | 0.978 | 0.987 |
| CDK1 | RB1 | 9606.ENSP00000378699 | 9606.ENSP00000267163 | 0 | 0 | 0 | 0 | 0.064 | 0.895 | 0.9 | 0.709 | 0.996 |
| CDK1 | TP53 | 9606.ENSP00000378699 | 9606.ENSP00000269305 | 0 | 0 | 0 | 0 | 0.096 | 0.68 | 0 | 0.927 | 0.977 |
| CDK1 | CCNA2 | 9606.ENSP00000378699 | 9606.ENSP00000274026 | 0 | 0.007 | 0 | 0 | 0.985 | 0.929 | 0.9 | 0.992 | 0.999 |
| CDK1 | EGFR | 9606.ENSP00000378699 | 9606.ENSP00000275493 | 0 | 0 | 0 | 0.62 | 0 | 0.78 | 0 | 0.593 | 0.828 |
| CDK1 | GJA1 | 9606.ENSP00000378699 | 9606.ENSP00000282561 | 0 | 0 | 0 | 0 | 0 | 0 | 0.8 | 0.235 | 0.84 |
| CDK1 | TOP1 | 9606.ENSP00000378699 | 9606.ENSP00000354522 | 0 | 0 | 0 | 0 | 0.09 | 0.056 | 0 | 0.49 | 0.524 |
| CDK1 | JUN | 9606.ENSP00000378699 | 9606.ENSP00000360266 | 0 | 0 | 0 | 0 | 0.049 | 0.281 | 0 | 0.583 | 0.69 |
| CDK1 | PTPN1 | 9606.ENSP00000378699 | 9606.ENSP00000360683 | 0 | 0 | 0 | 0 | 0.062 | 0.3 | 0 | 0.248 | 0.464 |
| CDK1 | AR | 9606.ENSP00000378699 | 9606.ENSP00000363822 | 0 | 0 | 0 | 0 | 0 | 0.226 | 0 | 0.819 | 0.854 |
| CDK1 | VEGFA | 9606.ENSP00000378699 | 9606.ENSP00000478570 | 0 | 0 | 0 | 0 | 0 | 0.105 | 0 | 0.413 | 0.452 |
| CDK1 | ESR1 | 9606.ENSP00000378699 | 9606.ENSP00000405330 | 0 | 0 | 0 | 0 | 0 | 0.121 | 0 | 0.502 | 0.544 |
| CDK1 | BCL2 | 9606.ENSP00000378699 | 9606.ENSP00000381185 | 0 | 0 | 0 | 0 | 0 | 0.68 | 0 | 0.146 | 0.715 |
| CDK1 | RXRA | 9606.ENSP00000378699 | 9606.ENSP00000419692 | 0 | 0 | 0 | 0 | 0 | 0.057 | 0.9 | 0.099 | 0.907 |
| CDK1 | CHEK1 | 9606.ENSP00000378699 | 9606.ENSP00000388648 | 0 | 0 | 0.287 | 0.726 | 0.855 | 0.483 | 0 | 0.921 | 0.945 |
| CDK1 | TOP2A | 9606.ENSP00000378699 | 9606.ENSP00000411532 | 0 | 0 | 0 | 0 | 0.988 | 0.225 | 0 | 0.672 | 0.996 |
| CDK2 | MAPK1 | 9606.ENSP00000266970 | 9606.ENSP00000215832 | 0 | 0 | 0.399 | 0.863 | 0.049 | 0.235 | 0.9 | 0.615 | 0.931 |
| CDK2 | MMP2 | 9606.ENSP00000266970 | 9606.ENSP00000219070 | 0 | 0 | 0 | 0 | 0 | 0 | 0 | 0.469 | 0.469 |
| CDK2 | CDK4 | 9606.ENSP00000266970 | 9606.ENSP00000257904 | 0 | 0 | 0.446 | 0.911 | 0.119 | 0.076 | 0.9 | 0.995 | 0.922 |
| CDK2 | MAPK3 | 9606.ENSP00000266970 | 9606.ENSP00000263025 | 0 | 0 | 0.405 | 0.872 | 0.049 | 0.235 | 0.9 | 0.664 | 0.931 |
| CDK2 | EGF | 9606.ENSP00000266970 | 9606.ENSP00000265171 | 0 | 0 | 0 | 0 | 0.062 | 0 | 0 | 0.573 | 0.582 |
| CDK2 | BAX | 9606.ENSP00000266970 | 9606.ENSP00000293288 | 0 | 0 | 0 | 0 | 0 | 0.056 | 0 | 0.391 | 0.4 |
| CDK2 | PTGS2 | 9606.ENSP00000266970 | 9606.ENSP00000356438 | 0 | 0 | 0 | 0 | 0 | 0 | 0 | 0.403 | 0.403 |
| CDK2 | PPARG | 9606.ENSP00000266970 | 9606.ENSP00000287820 | 0 | 0 | 0 | 0 | 0 | 0.057 | 0 | 0.41 | 0.42 |
| CDK2 | IL6 | 9606.ENSP00000266970 | 9606.ENSP00000385675 | 0 | 0 | 0 | 0 | 0 | 0 | 0 | 0.465 | 0.465 |
| CDK2 | BCL2 | 9606.ENSP00000266970 | 9606.ENSP00000381185 | 0 | 0 | 0 | 0 | 0 | 0.329 | 0 | 0.263 | 0.484 |
| CDK2 | TNF | 9606.ENSP00000266970 | 9606.ENSP00000398698 | 0 | 0 | 0 | 0 | 0 | 0.065 | 0 | 0.474 | 0.487 |
| CDK2 | AR | 9606.ENSP00000266970 | 9606.ENSP00000363822 | 0 | 0 | 0 | 0 | 0 | 0.057 | 0 | 0.531 | 0.539 |
| CDK2 | VEGFA | 9606.ENSP00000266970 | 9606.ENSP00000478570 | 0 | 0 | 0 | 0 | 0 | 0.105 | 0 | 0.522 | 0.554 |
| CDK2 | TOP1 | 9606.ENSP00000266970 | 9606.ENSP00000354522 | 0 | 0 | 0 | 0 | 0.091 | 0.078 | 0 | 0.513 | 0.557 |
| CDK2 | TOP2A | 9606.ENSP00000266970 | 9606.ENSP00000411532 | 0 | 0 | 0 | 0 | 0.376 | 0.056 | 0 | 0.454 | 0.65 |
| CDK2 | GJA1 | 9606.ENSP00000266970 | 9606.ENSP00000282561 | 0 | 0 | 0 | 0 | 0 | 0 | 0 | 0.705 | 0.706 |
| CDK2 | ESR1 | 9606.ENSP00000266970 | 9606.ENSP00000405330 | 0 | 0 | 0 | 0 | 0 | 0.282 | 0 | 0.62 | 0.715 |
| CDK2 | JUN | 9606.ENSP00000266970 | 9606.ENSP00000360266 | 0 | 0 | 0 | 0 | 0.049 | 0.239 | 0 | 0.7 | 0.765 |
| CDK2 | PGR | 9606.ENSP00000266970 | 9606.ENSP00000325120 | 0 | 0 | 0 | 0 | 0 | 0.494 | 0 | 0.677 | 0.83 |
| CDK2 | CHEK1 | 9606.ENSP00000266970 | 9606.ENSP00000388648 | 0 | 0 | 0.308 | 0.719 | 0.321 | 0.36 | 0.65 | 0.823 | 0.882 |
| CDK2 | CDK1 | 9606.ENSP00000266970 | 9606.ENSP00000378699 | 0 | 0 | 0.441 | 0.968 | 0.68 | 0.617 | 0.9 | 0.978 | 0.987 |
| CDK2 | RB1 | 9606.ENSP00000266970 | 9606.ENSP00000267163 | 0 | 0 | 0 | 0 | 0.082 | 0.929 | 0.9 | 0.71 | 0.997 |
| CDK2 | CCNA2 | 9606.ENSP00000266970 | 9606.ENSP00000274026 | 0 | 0.006 | 0 | 0 | 0.401 | 0.999 | 0.9 | 0.995 | 0.999 |
| CDK2 | TP53 | 9606.ENSP00000266970 | 9606.ENSP00000269305 | 0 | 0 | 0 | 0 | 0.095 | 0.79 | 0.9 | 0.97 | 0.999 |
| CDK4 | MAPK1 | 9606.ENSP00000257904 | 9606.ENSP00000215832 | 0 | 0 | 0.389 | 0.832 | 0.062 | 0.373 | 0 | 0.442 | 0.465 |
| CDK4 | MMP2 | 9606.ENSP00000257904 | 9606.ENSP00000219070 | 0 | 0 | 0 | 0 | 0 | 0 | 0 | 0.503 | 0.503 |
| CDK4 | PPARG | 9606.ENSP00000257904 | 9606.ENSP00000287820 | 0 | 0 | 0 | 0 | 0 | 0.057 | 0 | 0.414 | 0.423 |
| CDK4 | TNF | 9606.ENSP00000257904 | 9606.ENSP00000398698 | 0 | 0 | 0 | 0 | 0 | 0.065 | 0 | 0.429 | 0.443 |
| CDK4 | PPP3CA | 9606.ENSP00000257904 | 9606.ENSP00000378323 | 0 | 0 | 0 | 0 | 0.063 | 0.149 | 0 | 0.374 | 0.457 |
| CDK4 | IL6 | 9606.ENSP00000257904 | 9606.ENSP00000385675 | 0 | 0 | 0 | 0 | 0 | 0 | 0 | 0.458 | 0.457 |
| CDK4 | TOP1 | 9606.ENSP00000257904 | 9606.ENSP00000354522 | 0 | 0 | 0 | 0 | 0.063 | 0.192 | 0 | 0.361 | 0.474 |
| CDK4 | PGR | 9606.ENSP00000257904 | 9606.ENSP00000325120 | 0 | 0 | 0 | 0 | 0 | 0.057 | 0 | 0.468 | 0.476 |
| CDK4 | CHEK1 | 9606.ENSP00000257904 | 9606.ENSP00000388648 | 0 | 0 | 0.323 | 0.65 | 0.127 | 0.251 | 0 | 0.668 | 0.528 |
| CDK4 | AR | 9606.ENSP00000257904 | 9606.ENSP00000363822 | 0 | 0 | 0 | 0 | 0 | 0.057 | 0 | 0.523 | 0.53 |
| CDK4 | TOP2A | 9606.ENSP00000257904 | 9606.ENSP00000411532 | 0 | 0 | 0 | 0 | 0.212 | 0.056 | 0 | 0.418 | 0.53 |
| CDK4 | EGF | 9606.ENSP00000257904 | 9606.ENSP00000265171 | 0 | 0 | 0 | 0 | 0 | 0 | 0 | 0.573 | 0.573 |
| CDK4 | VEGFA | 9606.ENSP00000257904 | 9606.ENSP00000478570 | 0 | 0 | 0 | 0 | 0 | 0.155 | 0 | 0.565 | 0.616 |
| CDK4 | ESR1 | 9606.ENSP00000257904 | 9606.ENSP00000405330 | 0 | 0 | 0 | 0 | 0 | 0.057 | 0 | 0.681 | 0.686 |
| CDK4 | JUN | 9606.ENSP00000257904 | 9606.ENSP00000360266 | 0 | 0 | 0 | 0 | 0.062 | 0.239 | 0 | 0.624 | 0.708 |
| CDK4 | CDK2 | 9606.ENSP00000257904 | 9606.ENSP00000266970 | 0 | 0 | 0.446 | 0.911 | 0.119 | 0.076 | 0.9 | 0.995 | 0.922 |
| CDK4 | GSK3B | 9606.ENSP00000257904 | 9606.ENSP00000324806 | 0 | 0 | 0.347 | 0.735 | 0.064 | 0.061 | 0.9 | 0.497 | 0.923 |
| CDK4 | CDK1 | 9606.ENSP00000257904 | 9606.ENSP00000378699 | 0 | 0 | 0.436 | 0.905 | 0.213 | 0.119 | 0.9 | 0.913 | 0.933 |
| CDK4 | TP53 | 9606.ENSP00000257904 | 9606.ENSP00000269305 | 0 | 0 | 0 | 0 | 0.093 | 0.498 | 0 | 0.871 | 0.936 |
| CDK4 | CCNA2 | 9606.ENSP00000257904 | 9606.ENSP00000274026 | 0 | 0.003 | 0 | 0 | 0.183 | 0.587 | 0.9 | 0.946 | 0.997 |
| CDK4 | RB1 | 9606.ENSP00000257904 | 9606.ENSP00000267163 | 0 | 0 | 0 | 0 | 0.064 | 0.993 | 0.9 | 0.924 | 0.999 |
| CHEK1 | MAPK1 | 9606.ENSP00000388648 | 9606.ENSP00000215832 | 0 | 0 | 0.303 | 0.633 | 0.062 | 0.355 | 0 | 0.365 | 0.503 |
| CHEK1 | CDK4 | 9606.ENSP00000388648 | 9606.ENSP00000257904 | 0 | 0 | 0.323 | 0.65 | 0.127 | 0.251 | 0 | 0.668 | 0.528 |
| CHEK1 | CDK2 | 9606.ENSP00000388648 | 9606.ENSP00000266970 | 0 | 0 | 0.308 | 0.719 | 0.321 | 0.36 | 0.65 | 0.823 | 0.882 |
| CHEK1 | RB1 | 9606.ENSP00000388648 | 9606.ENSP00000267163 | 0 | 0 | 0 | 0 | 0.062 | 0.27 | 0 | 0.402 | 0.554 |
| CHEK1 | TP53 | 9606.ENSP00000388648 | 9606.ENSP00000269305 | 0 | 0 | 0 | 0 | 0.096 | 0.875 | 0.9 | 0.861 | 0.998 |
| CHEK1 | CCNA2 | 9606.ENSP00000388648 | 9606.ENSP00000274026 | 0 | 0 | 0 | 0 | 0.852 | 0.058 | 0 | 0.702 | 0.955 |
| CHEK1 | EGFR | 9606.ENSP00000388648 | 9606.ENSP00000275493 | 0 | 0 | 0 | 0 | 0 | 0 | 0 | 0.534 | 0.534 |
| CHEK1 | PIK3CG | 9606.ENSP00000388648 | 9606.ENSP00000352121 | 0 | 0 | 0 | 0 | 0 | 0.245 | 0 | 0.264 | 0.421 |
| CHEK1 | TOP1 | 9606.ENSP00000388648 | 9606.ENSP00000354522 | 0 | 0 | 0 | 0 | 0.062 | 0.205 | 0 | 0.82 | 0.854 |
| CHEK1 | JUN | 9606.ENSP00000388648 | 9606.ENSP00000360266 | 0 | 0 | 0 | 0 | 0 | 0.129 | 0 | 0.433 | 0.485 |
| CHEK1 | CDK1 | 9606.ENSP00000388648 | 9606.ENSP00000378699 | 0 | 0 | 0.287 | 0.726 | 0.855 | 0.483 | 0 | 0.921 | 0.945 |
| CHEK1 | VEGFA | 9606.ENSP00000388648 | 9606.ENSP00000478570 | 0 | 0 | 0 | 0 | 0 | 0.129 | 0 | 0.366 | 0.424 |
| CHEK1 | ESR1 | 9606.ENSP00000388648 | 9606.ENSP00000405330 | 0 | 0 | 0 | 0 | 0.062 | 0 | 0 | 0.422 | 0.434 |
| CHEK1 | TOP2A | 9606.ENSP00000388648 | 9606.ENSP00000411532 | 0 | 0 | 0 | 0 | 0.803 | 0.205 | 0 | 0.678 | 0.945 |
| CHRM1 | CHRM3 | 9606.ENSP00000306490 | 9606.ENSP00000255380 | 0 | 0 | 0 | 0.95 | 0.119 | 0 | 0.8 | 0.812 | 0.823 |
| CHRM1 | ACHE | 9606.ENSP00000306490 | 9606.ENSP00000303211 | 0 | 0 | 0 | 0 | 0.146 | 0 | 0 | 0.556 | 0.605 |
| CHRM1 | ADRB2 | 9606.ENSP00000306490 | 9606.ENSP00000305372 | 0 | 0 | 0 | 0.681 | 0.049 | 0 | 0.9 | 0.498 | 0.915 |
| CHRM1 | DRD1 | 9606.ENSP00000306490 | 9606.ENSP00000377353 | 0 | 0 | 0 | 0.65 | 0.292 | 0 | 0 | 0.573 | 0.429 |
| CHRM1 | CHRNA7 | 9606.ENSP00000306490 | 9606.ENSP00000407546 | 0 | 0 | 0 | 0 | 0.062 | 0 | 0 | 0.517 | 0.528 |
| CHRM1 | PIK3CG | 9606.ENSP00000306490 | 9606.ENSP00000352121 | 0 | 0 | 0 | 0 | 0 | 0 | 0.6 | 0.05 | 0.604 |
| CHRM1 | CHRM2 | 9606.ENSP00000306490 | 9606.ENSP00000399745 | 0 | 0 | 0 | 0.923 | 0.059 | 0 | 0.8 | 0.805 | 0.815 |
| CHRM1 | F2 | 9606.ENSP00000306490 | 9606.ENSP00000308541 | 0 | 0 | 0 | 0 | 0 | 0 | 0.9 | 0 | 0.9 |
| CHRM1 | JUN | 9606.ENSP00000306490 | 9606.ENSP00000360266 | 0 | 0 | 0 | 0 | 0 | 0 | 0.9 | 0.062 | 0.902 |
| CHRM1 | ADRA1B | 9606.ENSP00000306490 | 9606.ENSP00000306662 | 0 | 0 | 0 | 0.676 | 0.076 | 0 | 0.9 | 0.506 | 0.918 |
| CHRM2 | SLC6A4 | 9606.ENSP00000399745 | 9606.ENSP00000261707 | 0 | 0 | 0 | 0 | 0.062 | 0 | 0 | 0.471 | 0.483 |
| CHRM2 | ACHE | 9606.ENSP00000399745 | 9606.ENSP00000303211 | 0 | 0 | 0 | 0 | 0.065 | 0 | 0 | 0.461 | 0.474 |
| CHRM2 | ADRB2 | 9606.ENSP00000399745 | 9606.ENSP00000305372 | 0 | 0 | 0 | 0.652 | 0.052 | 0 | 0.5 | 0.522 | 0.592 |
| CHRM2 | CHRM1 | 9606.ENSP00000399745 | 9606.ENSP00000306490 | 0 | 0 | 0 | 0.923 | 0.059 | 0 | 0.8 | 0.805 | 0.815 |
| CHRM2 | ADH1B | 9606.ENSP00000399745 | 9606.ENSP00000306606 | 0 | 0 | 0 | 0 | 0 | 0 | 0 | 0.412 | 0.412 |
| CHRM2 | HTR3A | 9606.ENSP00000399745 | 9606.ENSP00000347754 | 0 | 0 | 0 | 0 | 0.062 | 0 | 0 | 0.417 | 0.43 |
| CHRM2 | PIK3CG | 9606.ENSP00000399745 | 9606.ENSP00000352121 | 0 | 0 | 0 | 0 | 0 | 0 | 0.6 | 0.073 | 0.613 |
| CHRM2 | MAP2 | 9606.ENSP00000399745 | 9606.ENSP00000353508 | 0 | 0 | 0 | 0 | 0.078 | 0 | 0 | 0.56 | 0.577 |
| CHRM2 | MAOB | 9606.ENSP00000399745 | 9606.ENSP00000367309 | 0 | 0 | 0 | 0 | 0.06 | 0 | 0 | 0.398 | 0.409 |
| CHRM2 | CHRNA7 | 9606.ENSP00000399745 | 9606.ENSP00000407546 | 0 | 0 | 0 | 0 | 0.063 | 0 | 0 | 0.534 | 0.545 |
| CHRM3 | IL6 | 9606.ENSP00000255380 | 9606.ENSP00000385675 | 0 | 0 | 0 | 0 | 0 | 0 | 0 | 0.411 | 0.411 |
| CHRM3 | CHRNA7 | 9606.ENSP00000255380 | 9606.ENSP00000407546 | 0 | 0 | 0 | 0 | 0.062 | 0 | 0 | 0.667 | 0.674 |
| CHRM3 | CHRM1 | 9606.ENSP00000255380 | 9606.ENSP00000306490 | 0 | 0 | 0 | 0.95 | 0.119 | 0 | 0.8 | 0.812 | 0.823 |
| CHRNA7 | OPRD1 | 9606.ENSP00000407546 | 9606.ENSP00000234961 | 0 | 0 | 0 | 0 | 0.074 | 0 | 0 | 0.444 | 0.464 |
| CHRNA7 | CHRM3 | 9606.ENSP00000407546 | 9606.ENSP00000255380 | 0 | 0 | 0 | 0 | 0.062 | 0 | 0 | 0.667 | 0.674 |
| CHRNA7 | SLC6A4 | 9606.ENSP00000407546 | 9606.ENSP00000261707 | 0 | 0 | 0 | 0 | 0 | 0 | 0 | 0.461 | 0.461 |
| CHRNA7 | SLC6A3 | 9606.ENSP00000407546 | 9606.ENSP00000270349 | 0 | 0 | 0 | 0 | 0 | 0 | 0 | 0.427 | 0.427 |
| CHRNA7 | ADRA2A | 9606.ENSP00000407546 | 9606.ENSP00000280155 | 0 | 0 | 0 | 0 | 0.062 | 0 | 0 | 0.424 | 0.436 |
| CHRNA7 | ACHE | 9606.ENSP00000407546 | 9606.ENSP00000303211 | 0 | 0 | 0 | 0 | 0.062 | 0 | 0 | 0.553 | 0.563 |
| CHRNA7 | CHRM1 | 9606.ENSP00000407546 | 9606.ENSP00000306490 | 0 | 0 | 0 | 0 | 0.062 | 0 | 0 | 0.517 | 0.528 |
| CHRNA7 | ADRA1D | 9606.ENSP00000407546 | 9606.ENSP00000368766 | 0 | 0 | 0 | 0 | 0.062 | 0 | 0 | 0.432 | 0.445 |
| CHRNA7 | DRD1 | 9606.ENSP00000407546 | 9606.ENSP00000377353 | 0 | 0 | 0 | 0 | 0.062 | 0 | 0 | 0.556 | 0.566 |
| CHRNA7 | CHRM2 | 9606.ENSP00000407546 | 9606.ENSP00000399745 | 0 | 0 | 0 | 0 | 0.063 | 0 | 0 | 0.534 | 0.545 |
| CHRNA7 | HTR2A | 9606.ENSP00000407546 | 9606.ENSP00000437737 | 0 | 0 | 0 | 0 | 0 | 0 | 0 | 0.506 | 0.506 |
| CTSD | MMP2 | 9606.ENSP00000236671 | 9606.ENSP00000219070 | 0 | 0 | 0 | 0 | 0.052 | 0 | 0 | 0.476 | 0.482 |
| CTSD | EGF | 9606.ENSP00000236671 | 9606.ENSP00000265171 | 0 | 0 | 0 | 0 | 0 | 0 | 0 | 0.401 | 0.4 |
| CTSD | VEGFA | 9606.ENSP00000236671 | 9606.ENSP00000478570 | 0 | 0 | 0 | 0 | 0 | 0 | 0 | 0.405 | 0.405 |
| CTSD | IL1B | 9606.ENSP00000236671 | 9606.ENSP00000263341 | 0 | 0 | 0 | 0 | 0.062 | 0 | 0 | 0.395 | 0.408 |
| CTSD | MMP3 | 9606.ENSP00000236671 | 9606.ENSP00000299855 | 0 | 0 | 0 | 0 | 0.052 | 0 | 0 | 0.419 | 0.426 |
| CTSD | ESR2 | 9606.ENSP00000236671 | 9606.ENSP00000343925 | 0 | 0 | 0 | 0 | 0 | 0.056 | 0 | 0.421 | 0.43 |
| CTSD | CAT | 9606.ENSP00000236671 | 9606.ENSP00000241052 | 0 | 0 | 0 | 0 | 0.086 | 0.138 | 0 | 0.381 | 0.47 |
| CTSD | PLAU | 9606.ENSP00000236671 | 9606.ENSP00000361850 | 0 | 0 | 0 | 0 | 0.065 | 0 | 0 | 0.459 | 0.472 |
| CTSD | TP53 | 9606.ENSP00000236671 | 9606.ENSP00000269305 | 0 | 0 | 0 | 0 | 0 | 0 | 0 | 0.518 | 0.518 |
| CTSD | TNF | 9606.ENSP00000236671 | 9606.ENSP00000398698 | 0 | 0 | 0 | 0 | 0.062 | 0 | 0 | 0.536 | 0.547 |
| CTSD | EGFR | 9606.ENSP00000236671 | 9606.ENSP00000275493 | 0 | 0 | 0 | 0 | 0 | 0.103 | 0 | 0.567 | 0.595 |
| CTSD | BCL2 | 9606.ENSP00000236671 | 9606.ENSP00000381185 | 0 | 0 | 0 | 0 | 0 | 0 | 0.6 | 0.061 | 0.608 |
| CTSD | PGR | 9606.ENSP00000236671 | 9606.ENSP00000325120 | 0 | 0 | 0 | 0 | 0 | 0.056 | 0 | 0.602 | 0.608 |
| CTSD | HSPA5 | 9606.ENSP00000236671 | 9606.ENSP00000324173 | 0 | 0 | 0 | 0 | 0.062 | 0.153 | 0 | 0.565 | 0.624 |
| CTSD | NCOA1 | 9606.ENSP00000236671 | 9606.ENSP00000385216 | 0 | 0 | 0 | 0 | 0 | 0 | 0.9 | 0.089 | 0.905 |
| CTSD | NCOA2 | 9606.ENSP00000236671 | 9606.ENSP00000399968 | 0 | 0 | 0 | 0 | 0 | 0 | 0.9 | 0.114 | 0.907 |
| CTSD | ESR1 | 9606.ENSP00000236671 | 9606.ENSP00000405330 | 0 | 0 | 0 | 0 | 0.088 | 0.056 | 0.9 | 0.606 | 0.961 |
| CYP1A2 | PON1 | 9606.ENSP00000342007 | 9606.ENSP00000222381 | 0 | 0 | 0 | 0 | 0 | 0 | 0 | 0.459 | 0.459 |
| CYP1A2 | CAT | 9606.ENSP00000342007 | 9606.ENSP00000241052 | 0.047 | 0 | 0 | 0 | 0 | 0 | 0 | 0.398 | 0.401 |
| CYP1A2 | AHR | 9606.ENSP00000342007 | 9606.ENSP00000242057 | 0 | 0 | 0 | 0 | 0.062 | 0 | 0 | 0.747 | 0.752 |
| CYP1A2 | SLC6A4 | 9606.ENSP00000342007 | 9606.ENSP00000261707 | 0 | 0 | 0 | 0 | 0 | 0 | 0 | 0.46 | 0.459 |
| CYP1A2 | KCNH2 | 9606.ENSP00000342007 | 9606.ENSP00000262186 | 0 | 0 | 0 | 0 | 0 | 0 | 0 | 0.475 | 0.475 |
| CYP1A2 | NQO1 | 9606.ENSP00000342007 | 9606.ENSP00000319788 | 0 | 0 | 0 | 0 | 0 | 0 | 0 | 0.529 | 0.529 |
| CYP1A2 | CYP3A4 | 9606.ENSP00000342007 | 9606.ENSP00000337915 | 0 | 0 | 0.442 | 0.604 | 0.139 | 0.077 | 0.9 | 0.927 | 0.954 |
| CYP1A2 | MAOA | 9606.ENSP00000342007 | 9606.ENSP00000340684 | 0 | 0 | 0 | 0 | 0.063 | 0 | 0 | 0.423 | 0.436 |
| CYP1A2 | MAOB | 9606.ENSP00000342007 | 9606.ENSP00000367309 | 0 | 0 | 0 | 0 | 0.063 | 0 | 0 | 0.391 | 0.405 |
| CYP1A2 | ESR1 | 9606.ENSP00000342007 | 9606.ENSP00000405330 | 0 | 0 | 0 | 0 | 0 | 0.073 | 0 | 0.391 | 0.411 |
| CYP1A2 | HTR2A | 9606.ENSP00000342007 | 9606.ENSP00000437737 | 0 | 0 | 0 | 0 | 0 | 0 | 0 | 0.494 | 0.493 |
| CYP1A2 | GSTP1 | 9606.ENSP00000342007 | 9606.ENSP00000381607 | 0 | 0 | 0 | 0 | 0 | 0.057 | 0.65 | 0.563 | 0.843 |
| CYP1A2 | POR | 9606.ENSP00000342007 | 9606.ENSP00000419970 | 0.042 | 0 | 0 | 0 | 0 | 0.494 | 0 | 0.77 | 0.879 |
| CYP1A2 | XDH | 9606.ENSP00000342007 | 9606.ENSP00000368727 | 0 | 0 | 0 | 0 | 0.051 | 0 | 0.9 | 0.134 | 0.91 |
| CYP1A2 | AKR1C3 | 9606.ENSP00000342007 | 9606.ENSP00000369927 | 0 | 0 | 0 | 0 | 0.062 | 0 | 0.9 | 0.198 | 0.918 |
| CYP3A4 | PON1 | 9606.ENSP00000337915 | 9606.ENSP00000222381 | 0 | 0 | 0 | 0 | 0.098 | 0 | 0 | 0.451 | 0.483 |
| CYP3A4 | NR3C1 | 9606.ENSP00000337915 | 9606.ENSP00000231509 | 0 | 0 | 0 | 0 | 0 | 0.073 | 0 | 0.582 | 0.596 |
| CYP3A4 | CAT | 9606.ENSP00000337915 | 9606.ENSP00000241052 | 0.047 | 0 | 0 | 0 | 0.049 | 0 | 0 | 0.43 | 0.438 |
| CYP3A4 | AHR | 9606.ENSP00000337915 | 9606.ENSP00000242057 | 0 | 0 | 0 | 0 | 0.062 | 0 | 0 | 0.677 | 0.684 |
| CYP3A4 | KCNH2 | 9606.ENSP00000337915 | 9606.ENSP00000262186 | 0 | 0 | 0 | 0 | 0 | 0 | 0 | 0.557 | 0.557 |
| CYP3A4 | TP53 | 9606.ENSP00000337915 | 9606.ENSP00000269305 | 0 | 0 | 0 | 0 | 0 | 0.058 | 0 | 0.417 | 0.427 |
| CYP3A4 | EGFR | 9606.ENSP00000337915 | 9606.ENSP00000275493 | 0 | 0 | 0 | 0 | 0 | 0 | 0 | 0.545 | 0.545 |
| CYP3A4 | PPARG | 9606.ENSP00000337915 | 9606.ENSP00000287820 | 0 | 0 | 0 | 0 | 0.053 | 0.073 | 0 | 0.413 | 0.439 |
| CYP3A4 | HMGCR | 9606.ENSP00000337915 | 9606.ENSP00000287936 | 0 | 0 | 0 | 0 | 0.064 | 0 | 0 | 0.502 | 0.514 |
| CYP3A4 | F2 | 9606.ENSP00000337915 | 9606.ENSP00000308541 | 0 | 0 | 0 | 0 | 0.087 | 0.061 | 0 | 0.502 | 0.535 |
| CYP3A4 | NQO1 | 9606.ENSP00000337915 | 9606.ENSP00000319788 | 0 | 0 | 0 | 0 | 0 | 0 | 0 | 0.454 | 0.454 |
| CYP3A4 | PGR | 9606.ENSP00000337915 | 9606.ENSP00000325120 | 0 | 0 | 0 | 0 | 0 | 0.073 | 0 | 0.398 | 0.418 |
| CYP3A4 | VEGFA | 9606.ENSP00000337915 | 9606.ENSP00000478570 | 0 | 0 | 0 | 0 | 0 | 0 | 0 | 0.404 | 0.404 |
| CYP3A4 | F10 | 9606.ENSP00000337915 | 9606.ENSP00000364709 | 0 | 0 | 0 | 0 | 0.062 | 0.061 | 0 | 0.391 | 0.416 |
| CYP3A4 | RXRA | 9606.ENSP00000337915 | 9606.ENSP00000419692 | 0 | 0 | 0 | 0 | 0 | 0.073 | 0 | 0.42 | 0.439 |
| CYP3A4 | IL6 | 9606.ENSP00000337915 | 9606.ENSP00000385675 | 0 | 0 | 0 | 0 | 0 | 0 | 0 | 0.457 | 0.457 |
| CYP3A4 | NCOA1 | 9606.ENSP00000337915 | 9606.ENSP00000385216 | 0 | 0 | 0 | 0 | 0 | 0 | 0 | 0.458 | 0.457 |
| CYP3A4 | DPP4 | 9606.ENSP00000337915 | 9606.ENSP00000353731 | 0 | 0 | 0 | 0 | 0 | 0 | 0 | 0.459 | 0.459 |
| CYP3A4 | AR | 9606.ENSP00000337915 | 9606.ENSP00000363822 | 0 | 0 | 0 | 0 | 0 | 0.073 | 0 | 0.455 | 0.473 |
| CYP3A4 | HTR2A | 9606.ENSP00000337915 | 9606.ENSP00000437737 | 0 | 0 | 0 | 0 | 0 | 0 | 0 | 0.503 | 0.503 |
| CYP3A4 | OPRM1 | 9606.ENSP00000337915 | 9606.ENSP00000394624 | 0 | 0 | 0 | 0 | 0 | 0 | 0 | 0.518 | 0.518 |
| CYP3A4 | ABCC1 | 9606.ENSP00000337915 | 9606.ENSP00000382342 | 0 | 0 | 0 | 0 | 0.063 | 0 | 0 | 0.528 | 0.539 |
| CYP3A4 | ESR1 | 9606.ENSP00000337915 | 9606.ENSP00000405330 | 0 | 0 | 0 | 0 | 0.049 | 0.073 | 0 | 0.621 | 0.637 |
| CYP3A4 | GSTP1 | 9606.ENSP00000337915 | 9606.ENSP00000381607 | 0 | 0 | 0 | 0 | 0 | 0.056 | 0.65 | 0.563 | 0.843 |
| CYP3A4 | AKR1C1 | 9606.ENSP00000337915 | 9606.ENSP00000370254 | 0 | 0 | 0 | 0 | 0.062 | 0 | 0.9 | 0.272 | 0.925 |
| CYP3A4 | MAOB | 9606.ENSP00000337915 | 9606.ENSP00000367309 | 0 | 0 | 0 | 0 | 0.064 | 0 | 0.9 | 0.285 | 0.927 |
| CYP3A4 | AKR1C3 | 9606.ENSP00000337915 | 9606.ENSP00000369927 | 0 | 0 | 0 | 0 | 0.062 | 0 | 0.9 | 0.336 | 0.932 |
| CYP3A4 | MAOA | 9606.ENSP00000337915 | 9606.ENSP00000340684 | 0 | 0 | 0 | 0 | 0.064 | 0 | 0.9 | 0.366 | 0.935 |
| CYP3A4 | CYP1A2 | 9606.ENSP00000337915 | 9606.ENSP00000342007 | 0 | 0 | 0.442 | 0.604 | 0.139 | 0.077 | 0.9 | 0.927 | 0.954 |
| CYP3A4 | POR | 9606.ENSP00000337915 | 9606.ENSP00000419970 | 0.042 | 0.384 | 0 | 0 | 0 | 0.282 | 0 | 0.941 | 0.972 |
| DPEP1 | DPP4 | 9606.ENSP00000376807 | 9606.ENSP00000353731 | 0.06 | 0 | 0 | 0 | 0.062 | 0 | 0 | 0.487 | 0.508 |
| DPP4 | MMP2 | 9606.ENSP00000353731 | 9606.ENSP00000219070 | 0 | 0 | 0 | 0 | 0.063 | 0 | 0 | 0.391 | 0.404 |
| DPP4 | CCL2 | 9606.ENSP00000353731 | 9606.ENSP00000225831 | 0 | 0 | 0 | 0 | 0 | 0 | 0 | 0.495 | 0.495 |
| DPP4 | IL2 | 9606.ENSP00000353731 | 9606.ENSP00000226730 | 0 | 0 | 0 | 0 | 0 | 0 | 0 | 0.459 | 0.459 |
| DPP4 | IFNG | 9606.ENSP00000353731 | 9606.ENSP00000229135 | 0 | 0 | 0 | 0 | 0 | 0 | 0 | 0.403 | 0.403 |
| DPP4 | IL1B | 9606.ENSP00000353731 | 9606.ENSP00000263341 | 0 | 0 | 0 | 0 | 0.062 | 0 | 0 | 0.465 | 0.476 |
| DPP4 | TP53 | 9606.ENSP00000353731 | 9606.ENSP00000269305 | 0 | 0 | 0 | 0 | 0 | 0 | 0 | 0.928 | 0.928 |
| DPP4 | PPARG | 9606.ENSP00000353731 | 9606.ENSP00000287820 | 0 | 0 | 0 | 0 | 0.062 | 0.078 | 0 | 0.566 | 0.591 |
| DPP4 | VCAM1 | 9606.ENSP00000353731 | 9606.ENSP00000294728 | 0 | 0 | 0 | 0 | 0 | 0 | 0 | 0.428 | 0.428 |
| DPP4 | NOS3 | 9606.ENSP00000353731 | 9606.ENSP00000297494 | 0.048 | 0 | 0 | 0 | 0 | 0 | 0 | 0.501 | 0.504 |
| DPP4 | CYP3A4 | 9606.ENSP00000353731 | 9606.ENSP00000337915 | 0 | 0 | 0 | 0 | 0 | 0 | 0 | 0.459 | 0.459 |
| DPP4 | ABCC1 | 9606.ENSP00000353731 | 9606.ENSP00000382342 | 0 | 0 | 0 | 0 | 0.062 | 0 | 0 | 0.433 | 0.445 |
| DPP4 | VEGFA | 9606.ENSP00000353731 | 9606.ENSP00000478570 | 0 | 0 | 0 | 0 | 0 | 0 | 0 | 0.451 | 0.451 |
| DPP4 | PTPN1 | 9606.ENSP00000353731 | 9606.ENSP00000360683 | 0 | 0 | 0 | 0 | 0 | 0 | 0 | 0.472 | 0.472 |
| DPP4 | DPEP1 | 9606.ENSP00000353731 | 9606.ENSP00000376807 | 0.06 | 0 | 0 | 0 | 0.062 | 0 | 0 | 0.487 | 0.508 |
| DPP4 | TNF | 9606.ENSP00000353731 | 9606.ENSP00000398698 | 0 | 0 | 0 | 0 | 0 | 0 | 0 | 0.532 | 0.532 |
| DPP4 | IL6 | 9606.ENSP00000353731 | 9606.ENSP00000385675 | 0 | 0 | 0 | 0 | 0 | 0 | 0 | 0.566 | 0.566 |
| DRD1 | SLC6A4 | 9606.ENSP00000377353 | 9606.ENSP00000261707 | 0 | 0 | 0 | 0 | 0.057 | 0.057 | 0 | 0.722 | 0.731 |
| DRD1 | SLC6A3 | 9606.ENSP00000377353 | 9606.ENSP00000270349 | 0 | 0 | 0 | 0 | 0.057 | 0.057 | 0 | 0.823 | 0.829 |
| DRD1 | CHRM1 | 9606.ENSP00000377353 | 9606.ENSP00000306490 | 0 | 0 | 0 | 0.65 | 0.292 | 0 | 0 | 0.573 | 0.429 |
| DRD1 | MAOA | 9606.ENSP00000377353 | 9606.ENSP00000340684 | 0 | 0 | 0 | 0 | 0 | 0 | 0 | 0.614 | 0.614 |
| DRD1 | HTR3A | 9606.ENSP00000377353 | 9606.ENSP00000347754 | 0 | 0 | 0 | 0 | 0.085 | 0 | 0 | 0.405 | 0.432 |
| DRD1 | MAOB | 9606.ENSP00000377353 | 9606.ENSP00000367309 | 0 | 0 | 0 | 0 | 0.123 | 0 | 0 | 0.525 | 0.566 |
| DRD1 | CHRNA7 | 9606.ENSP00000377353 | 9606.ENSP00000407546 | 0 | 0 | 0 | 0 | 0.062 | 0 | 0 | 0.556 | 0.566 |
| EGF | MAPK1 | 9606.ENSP00000265171 | 9606.ENSP00000215832 | 0 | 0 | 0 | 0 | 0 | 0 | 0 | 0.625 | 0.625 |
| EGF | HMOX1 | 9606.ENSP00000265171 | 9606.ENSP00000216117 | 0 | 0 | 0 | 0 | 0 | 0 | 0 | 0.4 | 0.4 |
| EGF | MMP2 | 9606.ENSP00000265171 | 9606.ENSP00000219070 | 0 | 0 | 0 | 0 | 0 | 0 | 0 | 0.671 | 0.671 |
| EGF | PLAT | 9606.ENSP00000265171 | 9606.ENSP00000220809 | 0 | 0 | 0 | 0 | 0.062 | 0.056 | 0 | 0.473 | 0.493 |
| EGF | MPO | 9606.ENSP00000265171 | 9606.ENSP00000225275 | 0 | 0 | 0 | 0 | 0.062 | 0.069 | 0 | 0.386 | 0.417 |
| EGF | CCL2 | 9606.ENSP00000265171 | 9606.ENSP00000225831 | 0 | 0 | 0 | 0 | 0 | 0 | 0 | 0.681 | 0.681 |
| EGF | IL2 | 9606.ENSP00000265171 | 9606.ENSP00000226730 | 0 | 0 | 0 | 0 | 0 | 0 | 0 | 0.615 | 0.615 |
| EGF | IFNG | 9606.ENSP00000265171 | 9606.ENSP00000229135 | 0 | 0 | 0 | 0 | 0 | 0 | 0 | 0.607 | 0.607 |
| EGF | MAPK14 | 9606.ENSP00000265171 | 9606.ENSP00000229795 | 0 | 0 | 0 | 0 | 0 | 0 | 0 | 0.562 | 0.562 |
| EGF | ODC1 | 9606.ENSP00000265171 | 9606.ENSP00000234111 | 0 | 0 | 0 | 0 | 0 | 0 | 0 | 0.424 | 0.424 |
| EGF | CTSD | 9606.ENSP00000265171 | 9606.ENSP00000236671 | 0 | 0 | 0 | 0 | 0 | 0 | 0 | 0.401 | 0.4 |
| EGF | CAT | 9606.ENSP00000265171 | 9606.ENSP00000241052 | 0 | 0 | 0 | 0 | 0 | 0 | 0 | 0.465 | 0.465 |
| EGF | AHR | 9606.ENSP00000265171 | 9606.ENSP00000242057 | 0 | 0 | 0 | 0 | 0 | 0 | 0 | 0.444 | 0.444 |
| EGF | CDK4 | 9606.ENSP00000265171 | 9606.ENSP00000257904 | 0 | 0 | 0 | 0 | 0 | 0 | 0 | 0.573 | 0.573 |
| EGF | MAPK3 | 9606.ENSP00000265171 | 9606.ENSP00000263025 | 0 | 0 | 0 | 0 | 0 | 0 | 0 | 0.82 | 0.82 |
| EGF | IL1B | 9606.ENSP00000265171 | 9606.ENSP00000263341 | 0 | 0 | 0 | 0 | 0 | 0 | 0 | 0.71 | 0.71 |
| EGF | KDR | 9606.ENSP00000265171 | 9606.ENSP00000263923 | 0 | 0 | 0 | 0 | 0 | 0.057 | 0.6 | 0.734 | 0.891 |
| EGF | PTGS1 | 9606.ENSP00000265171 | 9606.ENSP00000354612 | 0 | 0 | 0 | 0 | 0.062 | 0.069 | 0 | 0.376 | 0.408 |
| EGF | F10 | 9606.ENSP00000265171 | 9606.ENSP00000364709 | 0 | 0 | 0 | 0 | 0 | 0.056 | 0 | 0.436 | 0.445 |
| EGF | CDK1 | 9606.ENSP00000265171 | 9606.ENSP00000378699 | 0 | 0 | 0 | 0 | 0.062 | 0 | 0 | 0.46 | 0.471 |
| EGF | HSPA5 | 9606.ENSP00000265171 | 9606.ENSP00000324173 | 0 | 0 | 0 | 0 | 0 | 0 | 0 | 0.497 | 0.497 |
| EGF | MAP2 | 9606.ENSP00000265171 | 9606.ENSP00000353508 | 0 | 0 | 0 | 0 | 0.065 | 0 | 0 | 0.491 | 0.503 |
| EGF | SELE | 9606.ENSP00000265171 | 9606.ENSP00000331736 | 0 | 0 | 0 | 0 | 0 | 0 | 0 | 0.505 | 0.505 |
| EGF | CCNA2 | 9606.ENSP00000265171 | 9606.ENSP00000274026 | 0 | 0 | 0 | 0 | 0.062 | 0 | 0 | 0.502 | 0.512 |
| EGF | GJA1 | 9606.ENSP00000265171 | 9606.ENSP00000282561 | 0 | 0 | 0 | 0 | 0.062 | 0 | 0 | 0.503 | 0.513 |
| EGF | F7 | 9606.ENSP00000265171 | 9606.ENSP00000364731 | 0 | 0 | 0 | 0 | 0 | 0.056 | 0 | 0.517 | 0.525 |
| EGF | NOS3 | 9606.ENSP00000265171 | 9606.ENSP00000297494 | 0 | 0 | 0 | 0 | 0.062 | 0 | 0 | 0.517 | 0.527 |
| EGF | PPARG | 9606.ENSP00000265171 | 9606.ENSP00000287820 | 0 | 0 | 0 | 0 | 0 | 0.056 | 0 | 0.522 | 0.529 |
| EGF | MMP3 | 9606.ENSP00000265171 | 9606.ENSP00000299855 | 0 | 0 | 0 | 0 | 0 | 0 | 0 | 0.548 | 0.548 |
| EGF | MAPK8 | 9606.ENSP00000265171 | 9606.ENSP00000378974 | 0 | 0 | 0 | 0 | 0.061 | 0.076 | 0 | 0.523 | 0.549 |
| EGF | VCAM1 | 9606.ENSP00000265171 | 9606.ENSP00000294728 | 0 | 0 | 0 | 0 | 0 | 0 | 0 | 0.557 | 0.557 |
| EGF | PLAU | 9606.ENSP00000265171 | 9606.ENSP00000361850 | 0 | 0 | 0 | 0 | 0 | 0.056 | 0 | 0.56 | 0.566 |
| EGF | CDK2 | 9606.ENSP00000265171 | 9606.ENSP00000266970 | 0 | 0 | 0 | 0 | 0.062 | 0 | 0 | 0.573 | 0.582 |
| EGF | ESR2 | 9606.ENSP00000265171 | 9606.ENSP00000343925 | 0 | 0 | 0 | 0 | 0 | 0.056 | 0 | 0.579 | 0.585 |
| EGF | GSK3B | 9606.ENSP00000265171 | 9606.ENSP00000324806 | 0 | 0 | 0 | 0 | 0 | 0.056 | 0 | 0.602 | 0.609 |
| EGF | MMP1 | 9606.ENSP00000265171 | 9606.ENSP00000322788 | 0 | 0 | 0 | 0 | 0 | 0 | 0 | 0.626 | 0.626 |
| EGF | PTGS2 | 9606.ENSP00000265171 | 9606.ENSP00000356438 | 0 | 0 | 0 | 0 | 0.062 | 0.069 | 0 | 0.623 | 0.642 |
| EGF | PGR | 9606.ENSP00000265171 | 9606.ENSP00000325120 | 0 | 0 | 0 | 0 | 0 | 0.056 | 0 | 0.687 | 0.692 |
| EGF | JUN | 9606.ENSP00000265171 | 9606.ENSP00000360266 | 0 | 0 | 0 | 0 | 0 | 0 | 0 | 0.701 | 0.701 |
| EGF | AR | 9606.ENSP00000265171 | 9606.ENSP00000363822 | 0 | 0 | 0 | 0 | 0 | 0.056 | 0 | 0.72 | 0.724 |
| EGF | F2 | 9606.ENSP00000265171 | 9606.ENSP00000308541 | 0 | 0 | 0 | 0 | 0 | 0.056 | 0 | 0.747 | 0.751 |
| EGF | IL6 | 9606.ENSP00000265171 | 9606.ENSP00000385675 | 0 | 0 | 0 | 0 | 0 | 0 | 0 | 0.763 | 0.763 |
| EGF | TP53 | 9606.ENSP00000265171 | 9606.ENSP00000269305 | 0 | 0 | 0 | 0 | 0 | 0 | 0 | 0.766 | 0.766 |
| EGF | ESR1 | 9606.ENSP00000265171 | 9606.ENSP00000405330 | 0 | 0 | 0 | 0 | 0 | 0.056 | 0 | 0.782 | 0.785 |
| EGF | TNF | 9606.ENSP00000265171 | 9606.ENSP00000398698 | 0 | 0 | 0 | 0 | 0 | 0.059 | 0 | 0.783 | 0.787 |
| EGF | INSR | 9606.ENSP00000265171 | 9606.ENSP00000303830 | 0 | 0 | 0 | 0 | 0 | 0.056 | 0.8 | 0.323 | 0.861 |
| EGF | VEGFA | 9606.ENSP00000265171 | 9606.ENSP00000478570 | 0 | 0 | 0 | 0 | 0 | 0 | 0 | 0.878 | 0.878 |
| EGF | PTPN1 | 9606.ENSP00000265171 | 9606.ENSP00000360683 | 0 | 0 | 0 | 0 | 0 | 0.06 | 0.9 | 0.576 | 0.956 |
| EGF | F3 | 9606.ENSP00000265171 | 9606.ENSP00000334145 | 0 | 0 | 0 | 0 | 0 | 0 | 0 | 0.966 | 0.966 |
| EGF | EGFR | 9606.ENSP00000265171 | 9606.ENSP00000275493 | 0 | 0 | 0 | 0 | 0.16 | 0.982 | 0.9 | 0.991 | 0.999 |
| EGFR | MAPK1 | 9606.ENSP00000275493 | 9606.ENSP00000215832 | 0 | 0 | 0 | 0.577 | 0 | 0.44 | 0.9 | 0.832 | 0.962 |
| EGFR | MMP2 | 9606.ENSP00000275493 | 9606.ENSP00000219070 | 0 | 0 | 0 | 0 | 0.11 | 0 | 0 | 0.684 | 0.707 |
| EGFR | CCL2 | 9606.ENSP00000275493 | 9606.ENSP00000225831 | 0 | 0 | 0 | 0 | 0.062 | 0 | 0 | 0.517 | 0.527 |
| EGFR | IL2 | 9606.ENSP00000275493 | 9606.ENSP00000226730 | 0 | 0 | 0 | 0 | 0 | 0 | 0.6 | 0.556 | 0.815 |
| EGFR | IFNG | 9606.ENSP00000275493 | 9606.ENSP00000229135 | 0 | 0 | 0 | 0 | 0 | 0 | 0.6 | 0.515 | 0.798 |
| EGFR | MAPK14 | 9606.ENSP00000275493 | 9606.ENSP00000229795 | 0 | 0 | 0 | 0.577 | 0 | 0.736 | 0 | 0.647 | 0.807 |
| EGFR | NR3C1 | 9606.ENSP00000275493 | 9606.ENSP00000231509 | 0 | 0 | 0 | 0 | 0 | 0.664 | 0 | 0.394 | 0.788 |
| EGFR | CTSD | 9606.ENSP00000275493 | 9606.ENSP00000236671 | 0 | 0 | 0 | 0 | 0 | 0.103 | 0 | 0.567 | 0.595 |
| EGFR | CAT | 9606.ENSP00000275493 | 9606.ENSP00000241052 | 0 | 0 | 0 | 0 | 0.062 | 0 | 0 | 0.431 | 0.443 |
| EGFR | AHR | 9606.ENSP00000275493 | 9606.ENSP00000242057 | 0 | 0 | 0 | 0 | 0.062 | 0 | 0 | 0.412 | 0.425 |
| EGFR | MAPK3 | 9606.ENSP00000275493 | 9606.ENSP00000263025 | 0 | 0 | 0 | 0.58 | 0 | 0.413 | 0.9 | 0.859 | 0.96 |
| EGFR | IL1B | 9606.ENSP00000275493 | 9606.ENSP00000263341 | 0 | 0 | 0 | 0 | 0 | 0 | 0 | 0.561 | 0.561 |
| EGFR | KDR | 9606.ENSP00000275493 | 9606.ENSP00000263923 | 0 | 0 | 0 | 0.559 | 0.077 | 0.356 | 0 | 0.913 | 0.629 |
| EGFR | EGF | 9606.ENSP00000275493 | 9606.ENSP00000265171 | 0 | 0 | 0 | 0 | 0.16 | 0.982 | 0.9 | 0.991 | 0.999 |
| EGFR | RB1 | 9606.ENSP00000275493 | 9606.ENSP00000267163 | 0 | 0 | 0 | 0 | 0 | 0.056 | 0 | 0.513 | 0.52 |
| EGFR | TP53 | 9606.ENSP00000275493 | 9606.ENSP00000269305 | 0 | 0 | 0 | 0 | 0 | 0.279 | 0 | 0.877 | 0.908 |
| EGFR | CCNA2 | 9606.ENSP00000275493 | 9606.ENSP00000274026 | 0 | 0 | 0 | 0 | 0 | 0.103 | 0 | 0.566 | 0.594 |
| EGFR | SELE | 9606.ENSP00000275493 | 9606.ENSP00000331736 | 0 | 0 | 0 | 0 | 0 | 0 | 0 | 0.425 | 0.425 |
| EGFR | LDLR | 9606.ENSP00000275493 | 9606.ENSP00000454071 | 0 | 0 | 0 | 0 | 0.063 | 0.072 | 0 | 0.406 | 0.439 |
| EGFR | F3 | 9606.ENSP00000275493 | 9606.ENSP00000334145 | 0 | 0 | 0 | 0 | 0.11 | 0 | 0 | 0.404 | 0.446 |
| EGFR | NOS3 | 9606.ENSP00000275493 | 9606.ENSP00000297494 | 0 | 0 | 0 | 0 | 0 | 0 | 0 | 0.459 | 0.459 |
| EGFR | GJA1 | 9606.ENSP00000275493 | 9606.ENSP00000282561 | 0 | 0 | 0 | 0 | 0.158 | 0 | 0 | 0.39 | 0.464 |
| EGFR | VCAM1 | 9606.ENSP00000275493 | 9606.ENSP00000294728 | 0 | 0 | 0 | 0 | 0.076 | 0 | 0 | 0.453 | 0.472 |
| EGFR | ABCC1 | 9606.ENSP00000275493 | 9606.ENSP00000382342 | 0 | 0 | 0 | 0 | 0 | 0 | 0 | 0.478 | 0.478 |
| EGFR | PRKACA | 9606.ENSP00000275493 | 9606.ENSP00000309591 | 0 | 0 | 0 | 0.594 | 0 | 0.487 | 0 | 0.132 | 0.506 |
| EGFR | TOP2A | 9606.ENSP00000275493 | 9606.ENSP00000411532 | 0 | 0 | 0 | 0 | 0 | 0 | 0 | 0.515 | 0.515 |
| EGFR | ADRB2 | 9606.ENSP00000275493 | 9606.ENSP00000305372 | 0 | 0 | 0 | 0 | 0 | 0.122 | 0 | 0.491 | 0.534 |
| EGFR | CHEK1 | 9606.ENSP00000275493 | 9606.ENSP00000388648 | 0 | 0 | 0 | 0 | 0 | 0 | 0 | 0.534 | 0.534 |
| EGFR | MMP1 | 9606.ENSP00000275493 | 9606.ENSP00000322788 | 0 | 0 | 0 | 0 | 0.078 | 0 | 0 | 0.516 | 0.535 |
| EGFR | CYP3A4 | 9606.ENSP00000275493 | 9606.ENSP00000337915 | 0 | 0 | 0 | 0 | 0 | 0 | 0 | 0.545 | 0.545 |
| EGFR | PIK3CG | 9606.ENSP00000275493 | 9606.ENSP00000352121 | 0 | 0 | 0 | 0 | 0 | 0.058 | 0 | 0.537 | 0.545 |
| EGFR | FASN | 9606.ENSP00000275493 | 9606.ENSP00000304592 | 0 | 0 | 0 | 0 | 0.066 | 0 | 0 | 0.583 | 0.595 |
| EGFR | NCOA2 | 9606.ENSP00000275493 | 9606.ENSP00000399968 | 0 | 0 | 0 | 0 | 0.062 | 0 | 0 | 0.643 | 0.65 |
| EGFR | INSR | 9606.ENSP00000275493 | 9606.ENSP00000303830 | 0 | 0 | 0 | 0.574 | 0.062 | 0.107 | 0.5 | 0.567 | 0.651 |
| EGFR | PPARG | 9606.ENSP00000275493 | 9606.ENSP00000287820 | 0 | 0 | 0 | 0 | 0.062 | 0.316 | 0 | 0.517 | 0.663 |
| EGFR | PIM1 | 9606.ENSP00000275493 | 9606.ENSP00000362608 | 0 | 0 | 0 | 0.582 | 0.048 | 0.642 | 0 | 0.164 | 0.663 |
| EGFR | TOP1 | 9606.ENSP00000275493 | 9606.ENSP00000354522 | 0 | 0 | 0 | 0 | 0 | 0.27 | 0 | 0.602 | 0.697 |
| EGFR | GSTP1 | 9606.ENSP00000275493 | 9606.ENSP00000381607 | 0 | 0 | 0 | 0 | 0.068 | 0.487 | 0 | 0.422 | 0.699 |
| EGFR | PTGS2 | 9606.ENSP00000275493 | 9606.ENSP00000356438 | 0 | 0 | 0 | 0 | 0.062 | 0 | 0 | 0.7 | 0.706 |
| EGFR | ADRB1 | 9606.ENSP00000275493 | 9606.ENSP00000358301 | 0 | 0 | 0 | 0 | 0 | 0.122 | 0 | 0.684 | 0.711 |
| EGFR | TNF | 9606.ENSP00000275493 | 9606.ENSP00000398698 | 0 | 0 | 0 | 0 | 0.063 | 0 | 0 | 0.704 | 0.712 |
| EGFR | MAPK8 | 9606.ENSP00000275493 | 9606.ENSP00000378974 | 0 | 0 | 0 | 0.563 | 0 | 0.642 | 0 | 0.591 | 0.731 |
| EGFR | ESR2 | 9606.ENSP00000275493 | 9606.ENSP00000343925 | 0 | 0 | 0 | 0 | 0 | 0.316 | 0 | 0.663 | 0.759 |
| EGFR | JUN | 9606.ENSP00000275493 | 9606.ENSP00000360266 | 0 | 0 | 0 | 0 | 0.06 | 0.139 | 0 | 0.732 | 0.764 |
| EGFR | CDK1 | 9606.ENSP00000275493 | 9606.ENSP00000378699 | 0 | 0 | 0 | 0.62 | 0 | 0.78 | 0 | 0.593 | 0.828 |
| EGFR | MMP3 | 9606.ENSP00000275493 | 9606.ENSP00000299855 | 0 | 0 | 0 | 0 | 0.076 | 0 | 0.65 | 0.541 | 0.838 |
| EGFR | PGR | 9606.ENSP00000275493 | 9606.ENSP00000325120 | 0 | 0 | 0 | 0 | 0 | 0.102 | 0 | 0.833 | 0.844 |
| EGFR | IL6 | 9606.ENSP00000275493 | 9606.ENSP00000385675 | 0 | 0 | 0 | 0 | 0 | 0 | 0.6 | 0.683 | 0.867 |
| EGFR | VEGFA | 9606.ENSP00000275493 | 9606.ENSP00000478570 | 0 | 0 | 0 | 0 | 0.089 | 0 | 0.6 | 0.861 | 0.944 |
| EGFR | PLAU | 9606.ENSP00000275493 | 9606.ENSP00000361850 | 0 | 0 | 0 | 0 | 0.088 | 0 | 0.9 | 0.626 | 0.962 |
| EGFR | HSPA5 | 9606.ENSP00000275493 | 9606.ENSP00000324173 | 0 | 0 | 0 | 0 | 0 | 0.682 | 0 | 0.955 | 0.985 |
| EGFR | AR | 9606.ENSP00000275493 | 9606.ENSP00000363822 | 0 | 0 | 0 | 0 | 0.063 | 0.698 | 0 | 0.966 | 0.989 |
| EGFR | ESR1 | 9606.ENSP00000275493 | 9606.ENSP00000405330 | 0 | 0 | 0 | 0 | 0 | 0.696 | 0 | 0.988 | 0.996 |
| EGFR | PTPN1 | 9606.ENSP00000275493 | 9606.ENSP00000360683 | 0 | 0 | 0 | 0 | 0 | 0.893 | 0.9 | 0.988 | 0.999 |
| ESR1 | MAPK1 | 9606.ENSP00000405330 | 9606.ENSP00000215832 | 0 | 0 | 0 | 0 | 0 | 0.699 | 0.9 | 0.532 | 0.984 |
| ESR1 | MMP2 | 9606.ENSP00000405330 | 9606.ENSP00000219070 | 0 | 0 | 0 | 0 | 0 | 0 | 0 | 0.56 | 0.56 |
| ESR1 | CCL2 | 9606.ENSP00000405330 | 9606.ENSP00000225831 | 0 | 0 | 0 | 0 | 0.055 | 0 | 0 | 0.46 | 0.467 |
| ESR1 | IFNG | 9606.ENSP00000405330 | 9606.ENSP00000229135 | 0 | 0 | 0 | 0 | 0 | 0 | 0 | 0.415 | 0.415 |
| ESR1 | MAPK14 | 9606.ENSP00000405330 | 9606.ENSP00000229795 | 0 | 0 | 0 | 0 | 0 | 0.264 | 0.9 | 0.458 | 0.956 |
| ESR1 | NR3C1 | 9606.ENSP00000405330 | 9606.ENSP00000231509 | 0 | 0 | 0 | 0.66 | 0 | 0 | 0.9 | 0.89 | 0.93 |
| ESR1 | CTSD | 9606.ENSP00000405330 | 9606.ENSP00000236671 | 0 | 0 | 0 | 0 | 0.088 | 0.056 | 0.9 | 0.606 | 0.961 |
| ESR1 | CAT | 9606.ENSP00000405330 | 9606.ENSP00000241052 | 0 | 0 | 0 | 0 | 0.062 | 0.27 | 0 | 0.4 | 0.553 |
| ESR1 | AHR | 9606.ENSP00000405330 | 9606.ENSP00000242057 | 0 | 0 | 0 | 0 | 0 | 0.873 | 0 | 0.99 | 0.998 |
| ESR1 | CDK4 | 9606.ENSP00000405330 | 9606.ENSP00000257904 | 0 | 0 | 0 | 0 | 0 | 0.057 | 0 | 0.681 | 0.686 |
| ESR1 | SLC6A4 | 9606.ENSP00000405330 | 9606.ENSP00000261707 | 0 | 0 | 0 | 0 | 0.062 | 0.057 | 0 | 0.391 | 0.414 |
| ESR1 | MAPK3 | 9606.ENSP00000405330 | 9606.ENSP00000263025 | 0 | 0 | 0 | 0 | 0 | 0.264 | 0.9 | 0.683 | 0.974 |
| ESR1 | IL1B | 9606.ENSP00000405330 | 9606.ENSP00000263341 | 0 | 0 | 0 | 0 | 0 | 0 | 0 | 0.729 | 0.729 |
| ESR1 | KDR | 9606.ENSP00000405330 | 9606.ENSP00000263923 | 0 | 0 | 0 | 0 | 0 | 0.057 | 0 | 0.468 | 0.476 |
| ESR1 | EGF | 9606.ENSP00000405330 | 9606.ENSP00000265171 | 0 | 0 | 0 | 0 | 0 | 0.056 | 0 | 0.782 | 0.785 |
| ESR1 | CDK2 | 9606.ENSP00000405330 | 9606.ENSP00000266970 | 0 | 0 | 0 | 0 | 0 | 0.282 | 0 | 0.62 | 0.715 |
| ESR1 | RB1 | 9606.ENSP00000405330 | 9606.ENSP00000267163 | 0 | 0 | 0 | 0 | 0 | 0.077 | 0 | 0.782 | 0.79 |
| ESR1 | TP53 | 9606.ENSP00000405330 | 9606.ENSP00000269305 | 0 | 0 | 0 | 0 | 0 | 0.835 | 0 | 0.968 | 0.994 |
| ESR1 | SOD1 | 9606.ENSP00000405330 | 9606.ENSP00000270142 | 0 | 0 | 0 | 0 | 0.062 | 0 | 0 | 0.703 | 0.71 |
| ESR1 | SLC6A3 | 9606.ENSP00000405330 | 9606.ENSP00000270349 | 0 | 0 | 0 | 0 | 0.053 | 0.057 | 0 | 0.453 | 0.469 |
| ESR1 | CCNA2 | 9606.ENSP00000405330 | 9606.ENSP00000274026 | 0 | 0 | 0 | 0 | 0 | 0.056 | 0 | 0.558 | 0.564 |
| ESR1 | EGFR | 9606.ENSP00000405330 | 9606.ENSP00000275493 | 0 | 0 | 0 | 0 | 0 | 0.696 | 0 | 0.988 | 0.996 |
| ESR1 | GJA1 | 9606.ENSP00000405330 | 9606.ENSP00000282561 | 0 | 0 | 0 | 0 | 0 | 0 | 0 | 0.424 | 0.424 |
| ESR1 | NOS3 | 9606.ENSP00000405330 | 9606.ENSP00000297494 | 0 | 0 | 0 | 0 | 0 | 0.345 | 0.9 | 0.985 | 0.998 |
| ESR1 | MMP3 | 9606.ENSP00000405330 | 9606.ENSP00000299855 | 0 | 0 | 0 | 0 | 0 | 0 | 0 | 0.435 | 0.435 |
| ESR1 | INSR | 9606.ENSP00000405330 | 9606.ENSP00000303830 | 0 | 0 | 0 | 0 | 0 | 0.077 | 0 | 0.391 | 0.413 |
| ESR1 | FASN | 9606.ENSP00000405330 | 9606.ENSP00000304592 | 0 | 0 | 0 | 0 | 0 | 0.072 | 0 | 0.391 | 0.41 |
| ESR1 | PRKACA | 9606.ENSP00000405330 | 9606.ENSP00000309591 | 0 | 0 | 0 | 0 | 0 | 0.209 | 0.8 | 0.073 | 0.84 |
| ESR1 | NQO1 | 9606.ENSP00000405330 | 9606.ENSP00000319788 | 0 | 0 | 0 | 0 | 0 | 0 | 0 | 0.486 | 0.486 |
| ESR1 | MMP1 | 9606.ENSP00000405330 | 9606.ENSP00000322788 | 0 | 0 | 0 | 0 | 0 | 0 | 0 | 0.418 | 0.418 |
| ESR1 | HSPA5 | 9606.ENSP00000405330 | 9606.ENSP00000324173 | 0 | 0 | 0 | 0 | 0 | 0.309 | 0 | 0.404 | 0.571 |
| ESR1 | GSK3B | 9606.ENSP00000405330 | 9606.ENSP00000324806 | 0 | 0 | 0 | 0 | 0 | 0.077 | 0 | 0.618 | 0.633 |
| ESR1 | PGR | 9606.ENSP00000405330 | 9606.ENSP00000325120 | 0 | 0 | 0 | 0.679 | 0.102 | 0.825 | 0.6 | 0.965 | 0.952 |
| ESR1 | CYP3A4 | 9606.ENSP00000405330 | 9606.ENSP00000337915 | 0 | 0 | 0 | 0 | 0.049 | 0.073 | 0 | 0.621 | 0.637 |
| ESR1 | CYP1A2 | 9606.ENSP00000405330 | 9606.ENSP00000342007 | 0 | 0 | 0 | 0 | 0 | 0.073 | 0 | 0.391 | 0.411 |
| ESR1 | ESR2 | 9606.ENSP00000405330 | 9606.ENSP00000343925 | 0 | 0 | 0 | 0.925 | 0 | 0.873 | 0.9 | 0.991 | 0.987 |
| ESR1 | MAPK10 | 9606.ENSP00000405330 | 9606.ENSP00000352157 | 0 | 0 | 0 | 0 | 0 | 0 | 0.8 | 0.158 | 0.824 |
| ESR1 | PTGS2 | 9606.ENSP00000405330 | 9606.ENSP00000356438 | 0 | 0 | 0 | 0 | 0 | 0.056 | 0 | 0.632 | 0.637 |
| ESR1 | JUN | 9606.ENSP00000405330 | 9606.ENSP00000360266 | 0 | 0 | 0 | 0 | 0 | 0.684 | 0.9 | 0.988 | 0.999 |
| ESR1 | PTPN1 | 9606.ENSP00000405330 | 9606.ENSP00000360683 | 0 | 0 | 0 | 0 | 0 | 0.291 | 0 | 0.3 | 0.482 |
| ESR1 | PLAU | 9606.ENSP00000405330 | 9606.ENSP00000361850 | 0 | 0 | 0 | 0 | 0.062 | 0 | 0 | 0.452 | 0.463 |
| ESR1 | AR | 9606.ENSP00000405330 | 9606.ENSP00000363822 | 0 | 0 | 0 | 0.656 | 0.062 | 0.213 | 0 | 0.984 | 0.49 |
| ESR1 | AKR1C3 | 9606.ENSP00000405330 | 9606.ENSP00000369927 | 0 | 0 | 0 | 0 | 0 | 0.056 | 0 | 0.407 | 0.416 |
| ESR1 | CDK1 | 9606.ENSP00000405330 | 9606.ENSP00000378699 | 0 | 0 | 0 | 0 | 0 | 0.121 | 0 | 0.502 | 0.544 |
| ESR1 | MAPK8 | 9606.ENSP00000405330 | 9606.ENSP00000378974 | 0 | 0 | 0 | 0 | 0 | 0 | 0.8 | 0.456 | 0.886 |
| ESR1 | BCL2 | 9606.ENSP00000405330 | 9606.ENSP00000381185 | 0 | 0 | 0 | 0 | 0.069 | 0.057 | 0.9 | 0.244 | 0.924 |
| ESR1 | GSTP1 | 9606.ENSP00000405330 | 9606.ENSP00000381607 | 0 | 0 | 0 | 0 | 0 | 0.062 | 0 | 0.56 | 0.569 |
| ESR1 | NCOA1 | 9606.ENSP00000405330 | 9606.ENSP00000385216 | 0 | 0 | 0 | 0 | 0 | 0.984 | 0.9 | 0.989 | 0.999 |
| ESR1 | IL6 | 9606.ENSP00000405330 | 9606.ENSP00000385675 | 0 | 0 | 0 | 0 | 0 | 0 | 0 | 0.677 | 0.677 |
| ESR1 | CHEK1 | 9606.ENSP00000405330 | 9606.ENSP00000388648 | 0 | 0 | 0 | 0 | 0.062 | 0 | 0 | 0.422 | 0.434 |
| ESR1 | TNF | 9606.ENSP00000405330 | 9606.ENSP00000398698 | 0 | 0 | 0 | 0 | 0 | 0 | 0 | 0.622 | 0.622 |
| ESR1 | NCOA2 | 9606.ENSP00000405330 | 9606.ENSP00000399968 | 0 | 0 | 0 | 0 | 0.062 | 0.989 | 0.9 | 0.965 | 0.999 |
| ESR1 | TOP2A | 9606.ENSP00000405330 | 9606.ENSP00000411532 | 0 | 0 | 0 | 0 | 0.062 | 0.044 | 0 | 0.469 | 0.482 |
| ESR1 | VEGFA | 9606.ENSP00000405330 | 9606.ENSP00000478570 | 0 | 0 | 0 | 0 | 0.062 | 0 | 0 | 0.685 | 0.691 |
| ESR2 | MAPK1 | 9606.ENSP00000343925 | 9606.ENSP00000215832 | 0 | 0 | 0 | 0 | 0 | 0.317 | 0.8 | 0.304 | 0.896 |
| ESR2 | MAPK14 | 9606.ENSP00000343925 | 9606.ENSP00000229795 | 0 | 0 | 0 | 0 | 0.062 | 0.104 | 0.8 | 0.244 | 0.855 |
| ESR2 | CTSD | 9606.ENSP00000343925 | 9606.ENSP00000236671 | 0 | 0 | 0 | 0 | 0 | 0.056 | 0 | 0.421 | 0.43 |
| ESR2 | AHR | 9606.ENSP00000343925 | 9606.ENSP00000242057 | 0 | 0 | 0 | 0 | 0 | 0.057 | 0 | 0.668 | 0.674 |
| ESR2 | MAPK3 | 9606.ENSP00000343925 | 9606.ENSP00000263025 | 0 | 0 | 0 | 0 | 0 | 0.104 | 0.8 | 0.57 | 0.916 |
| ESR2 | IL1B | 9606.ENSP00000343925 | 9606.ENSP00000263341 | 0 | 0 | 0 | 0 | 0 | 0 | 0 | 0.46 | 0.459 |
| ESR2 | EGF | 9606.ENSP00000343925 | 9606.ENSP00000265171 | 0 | 0 | 0 | 0 | 0 | 0.056 | 0 | 0.579 | 0.585 |
| ESR2 | RB1 | 9606.ENSP00000343925 | 9606.ENSP00000267163 | 0 | 0 | 0 | 0 | 0 | 0.077 | 0 | 0.519 | 0.537 |
| ESR2 | TP53 | 9606.ENSP00000343925 | 9606.ENSP00000269305 | 0 | 0 | 0 | 0 | 0 | 0 | 0 | 0.881 | 0.881 |
| ESR2 | EGFR | 9606.ENSP00000343925 | 9606.ENSP00000275493 | 0 | 0 | 0 | 0 | 0 | 0.316 | 0 | 0.663 | 0.759 |
| ESR2 | NOS3 | 9606.ENSP00000343925 | 9606.ENSP00000297494 | 0 | 0 | 0 | 0 | 0 | 0.067 | 0.9 | 0.851 | 0.984 |
| ESR2 | PRKACA | 9606.ENSP00000343925 | 9606.ENSP00000309591 | 0 | 0 | 0 | 0 | 0 | 0 | 0.8 | 0.06 | 0.804 |
| ESR2 | GSTP1 | 9606.ENSP00000343925 | 9606.ENSP00000381607 | 0 | 0 | 0 | 0 | 0 | 0.062 | 0 | 0.391 | 0.404 |
| ESR2 | AKR1C1 | 9606.ENSP00000343925 | 9606.ENSP00000370254 | 0 | 0 | 0 | 0 | 0 | 0.056 | 0 | 0.446 | 0.454 |
| ESR2 | TNF | 9606.ENSP00000343925 | 9606.ENSP00000398698 | 0 | 0 | 0 | 0 | 0 | 0 | 0 | 0.514 | 0.514 |
| ESR2 | PTGS2 | 9606.ENSP00000343925 | 9606.ENSP00000356438 | 0 | 0 | 0 | 0 | 0 | 0.056 | 0 | 0.52 | 0.527 |
| ESR2 | IL6 | 9606.ENSP00000343925 | 9606.ENSP00000385675 | 0 | 0 | 0 | 0 | 0 | 0 | 0 | 0.535 | 0.535 |
| ESR2 | VEGFA | 9606.ENSP00000343925 | 9606.ENSP00000478570 | 0 | 0 | 0 | 0 | 0 | 0 | 0 | 0.556 | 0.556 |
| ESR2 | MAPK10 | 9606.ENSP00000343925 | 9606.ENSP00000352157 | 0 | 0 | 0 | 0 | 0.062 | 0 | 0.8 | 0.07 | 0.81 |
| ESR2 | MAPK8 | 9606.ENSP00000343925 | 9606.ENSP00000378974 | 0 | 0 | 0 | 0 | 0.062 | 0 | 0.8 | 0.232 | 0.843 |
| ESR2 | JUN | 9606.ENSP00000343925 | 9606.ENSP00000360266 | 0 | 0 | 0 | 0 | 0 | 0.066 | 0.8 | 0.795 | 0.958 |
| ESR2 | ESR1 | 9606.ENSP00000343925 | 9606.ENSP00000405330 | 0 | 0 | 0 | 0.925 | 0 | 0.873 | 0.9 | 0.991 | 0.987 |
| ESR2 | NCOA1 | 9606.ENSP00000343925 | 9606.ENSP00000385216 | 0 | 0 | 0 | 0 | 0 | 0.977 | 0.9 | 0.971 | 0.999 |
| ESR2 | NCOA2 | 9606.ENSP00000343925 | 9606.ENSP00000399968 | 0 | 0 | 0 | 0 | 0 | 0.847 | 0.9 | 0.952 | 0.999 |
| F10 | PLAT | 9606.ENSP00000364709 | 9606.ENSP00000220809 | 0 | 0 | 0.408 | 0.632 | 0.061 | 0.298 | 0 | 0.67 | 0.552 |
| F10 | EGF | 9606.ENSP00000364709 | 9606.ENSP00000265171 | 0 | 0 | 0 | 0 | 0 | 0.056 | 0 | 0.436 | 0.445 |
| F10 | F2 | 9606.ENSP00000364709 | 9606.ENSP00000308541 | 0 | 0 | 0.391 | 0.763 | 0.199 | 0 | 0.9 | 0.979 | 0.941 |
| F10 | F3 | 9606.ENSP00000364709 | 9606.ENSP00000334145 | 0 | 0 | 0 | 0 | 0 | 0.213 | 0.6 | 0.988 | 0.996 |
| F10 | CYP3A4 | 9606.ENSP00000364709 | 9606.ENSP00000337915 | 0 | 0 | 0 | 0 | 0.062 | 0.061 | 0 | 0.391 | 0.416 |
| F10 | IL6 | 9606.ENSP00000364709 | 9606.ENSP00000385675 | 0 | 0 | 0 | 0 | 0.062 | 0 | 0 | 0.395 | 0.408 |
| F10 | TNF | 9606.ENSP00000364709 | 9606.ENSP00000398698 | 0 | 0 | 0 | 0 | 0.062 | 0 | 0 | 0.513 | 0.524 |
| F10 | THBD | 9606.ENSP00000364709 | 9606.ENSP00000366307 | 0 | 0 | 0 | 0 | 0 | 0 | 0.65 | 0.639 | 0.868 |
| F10 | F7 | 9606.ENSP00000364709 | 9606.ENSP00000364731 | 0 | 0 | 0.423 | 0.897 | 0.419 | 0.27 | 0.8 | 0.927 | 0.92 |
| F2 | MAPK1 | 9606.ENSP00000308541 | 9606.ENSP00000215832 | 0 | 0 | 0 | 0 | 0 | 0.056 | 0.9 | 0.204 | 0.918 |
| F2 | PLAT | 9606.ENSP00000308541 | 9606.ENSP00000220809 | 0 | 0 | 0.395 | 0.642 | 0 | 0.653 | 0.9 | 0.739 | 0.976 |
| F2 | MPO | 9606.ENSP00000308541 | 9606.ENSP00000225275 | 0 | 0 | 0 | 0 | 0 | 0.056 | 0 | 0.503 | 0.51 |
| F2 | CCL2 | 9606.ENSP00000308541 | 9606.ENSP00000225831 | 0 | 0 | 0 | 0 | 0 | 0 | 0 | 0.416 | 0.416 |
| F2 | IFNG | 9606.ENSP00000308541 | 9606.ENSP00000229135 | 0 | 0 | 0 | 0 | 0 | 0 | 0 | 0.402 | 0.402 |
| F2 | MAPK14 | 9606.ENSP00000308541 | 9606.ENSP00000229795 | 0 | 0 | 0 | 0 | 0 | 0.056 | 0 | 0.444 | 0.452 |
| F2 | MAPK3 | 9606.ENSP00000308541 | 9606.ENSP00000263025 | 0 | 0 | 0 | 0 | 0 | 0.056 | 0.9 | 0.348 | 0.933 |
| F2 | IL1B | 9606.ENSP00000308541 | 9606.ENSP00000263341 | 0 | 0 | 0 | 0 | 0 | 0 | 0 | 0.556 | 0.556 |
| F2 | EGF | 9606.ENSP00000308541 | 9606.ENSP00000265171 | 0 | 0 | 0 | 0 | 0 | 0.056 | 0 | 0.747 | 0.751 |
| F2 | VCAM1 | 9606.ENSP00000308541 | 9606.ENSP00000294728 | 0 | 0 | 0 | 0 | 0 | 0 | 0 | 0.508 | 0.508 |
| F2 | NOS3 | 9606.ENSP00000308541 | 9606.ENSP00000297494 | 0 | 0 | 0 | 0 | 0 | 0 | 0 | 0.405 | 0.405 |
| F2 | ADRB2 | 9606.ENSP00000308541 | 9606.ENSP00000305372 | 0 | 0 | 0 | 0 | 0 | 0.097 | 0.9 | 0.173 | 0.918 |
| F2 | CHRM1 | 9606.ENSP00000308541 | 9606.ENSP00000306490 | 0 | 0 | 0 | 0 | 0 | 0 | 0.9 | 0 | 0.9 |
| F2 | ADRA1B | 9606.ENSP00000308541 | 9606.ENSP00000306662 | 0 | 0 | 0 | 0 | 0.066 | 0.097 | 0.9 | 0 | 0.908 |
| F2 | F7 | 9606.ENSP00000308541 | 9606.ENSP00000364731 | 0 | 0 | 0.376 | 0.705 | 0.265 | 0 | 0 | 0.956 | 0.526 |
| F2 | CYP3A4 | 9606.ENSP00000308541 | 9606.ENSP00000337915 | 0 | 0 | 0 | 0 | 0.087 | 0.061 | 0 | 0.502 | 0.535 |
| F2 | SELE | 9606.ENSP00000308541 | 9606.ENSP00000331736 | 0 | 0 | 0 | 0 | 0.049 | 0 | 0 | 0.553 | 0.556 |
| F2 | VEGFA | 9606.ENSP00000308541 | 9606.ENSP00000478570 | 0 | 0 | 0 | 0 | 0 | 0 | 0 | 0.583 | 0.583 |
| F2 | TNF | 9606.ENSP00000308541 | 9606.ENSP00000398698 | 0 | 0 | 0 | 0 | 0 | 0 | 0 | 0.627 | 0.627 |
| F2 | IL6 | 9606.ENSP00000308541 | 9606.ENSP00000385675 | 0 | 0 | 0 | 0 | 0 | 0 | 0 | 0.633 | 0.633 |
| F2 | PLAU | 9606.ENSP00000308541 | 9606.ENSP00000361850 | 0 | 0 | 0.415 | 0.645 | 0 | 0.213 | 0.9 | 0.467 | 0.94 |
| F2 | F10 | 9606.ENSP00000308541 | 9606.ENSP00000364709 | 0 | 0 | 0.391 | 0.763 | 0.199 | 0 | 0.9 | 0.979 | 0.941 |
| F2 | F3 | 9606.ENSP00000308541 | 9606.ENSP00000334145 | 0 | 0 | 0 | 0 | 0 | 0 | 0.9 | 0.921 | 0.991 |
| F2 | THBD | 9606.ENSP00000308541 | 9606.ENSP00000366307 | 0 | 0 | 0 | 0 | 0 | 0.962 | 0.8 | 0.925 | 0.999 |
| F3 | MMP2 | 9606.ENSP00000334145 | 9606.ENSP00000219070 | 0 | 0 | 0 | 0 | 0.062 | 0 | 0 | 0.421 | 0.433 |
| F3 | PLAT | 9606.ENSP00000334145 | 9606.ENSP00000220809 | 0 | 0 | 0 | 0 | 0.076 | 0 | 0 | 0.801 | 0.808 |
| F3 | MPO | 9606.ENSP00000334145 | 9606.ENSP00000225275 | 0 | 0 | 0 | 0 | 0 | 0 | 0 | 0.594 | 0.594 |
| F3 | CCL2 | 9606.ENSP00000334145 | 9606.ENSP00000225831 | 0 | 0 | 0 | 0 | 0.083 | 0 | 0 | 0.66 | 0.675 |
| F3 | IL2 | 9606.ENSP00000334145 | 9606.ENSP00000226730 | 0 | 0 | 0 | 0 | 0 | 0 | 0 | 0.414 | 0.414 |
| F3 | IFNG | 9606.ENSP00000334145 | 9606.ENSP00000229135 | 0 | 0 | 0 | 0 | 0 | 0 | 0 | 0.474 | 0.474 |
| F3 | IL1B | 9606.ENSP00000334145 | 9606.ENSP00000263341 | 0 | 0 | 0 | 0 | 0.062 | 0 | 0 | 0.653 | 0.66 |
| F3 | KDR | 9606.ENSP00000334145 | 9606.ENSP00000263923 | 0 | 0 | 0 | 0 | 0 | 0 | 0 | 0.421 | 0.421 |
| F3 | EGF | 9606.ENSP00000334145 | 9606.ENSP00000265171 | 0 | 0 | 0 | 0 | 0 | 0 | 0 | 0.966 | 0.966 |
| F3 | EGFR | 9606.ENSP00000334145 | 9606.ENSP00000275493 | 0 | 0 | 0 | 0 | 0.11 | 0 | 0 | 0.404 | 0.446 |
| F3 | VCAM1 | 9606.ENSP00000334145 | 9606.ENSP00000294728 | 0 | 0 | 0 | 0 | 0.062 | 0 | 0 | 0.707 | 0.713 |
| F3 | NOS3 | 9606.ENSP00000334145 | 9606.ENSP00000297494 | 0 | 0 | 0 | 0 | 0 | 0 | 0 | 0.6 | 0.6 |
| F3 | F2 | 9606.ENSP00000334145 | 9606.ENSP00000308541 | 0 | 0 | 0 | 0 | 0 | 0 | 0.9 | 0.921 | 0.991 |
| F3 | HSPA5 | 9606.ENSP00000334145 | 9606.ENSP00000324173 | 0 | 0 | 0 | 0 | 0 | 0 | 0 | 0.46 | 0.46 |
| F3 | SELE | 9606.ENSP00000334145 | 9606.ENSP00000331736 | 0 | 0 | 0 | 0 | 0 | 0 | 0 | 0.715 | 0.715 |
| F3 | PTGS1 | 9606.ENSP00000334145 | 9606.ENSP00000354612 | 0 | 0 | 0 | 0 | 0.062 | 0 | 0 | 0.39 | 0.403 |
| F3 | JUN | 9606.ENSP00000334145 | 9606.ENSP00000360266 | 0 | 0 | 0 | 0 | 0 | 0 | 0 | 0.416 | 0.416 |
| F3 | PTGS2 | 9606.ENSP00000334145 | 9606.ENSP00000356438 | 0 | 0 | 0 | 0 | 0.085 | 0 | 0 | 0.463 | 0.487 |
| F3 | PLAU | 9606.ENSP00000334145 | 9606.ENSP00000361850 | 0 | 0 | 0 | 0 | 0.076 | 0 | 0 | 0.556 | 0.572 |
| F3 | VEGFA | 9606.ENSP00000334145 | 9606.ENSP00000478570 | 0 | 0 | 0 | 0 | 0.062 | 0 | 0 | 0.681 | 0.687 |
| F3 | TNF | 9606.ENSP00000334145 | 9606.ENSP00000398698 | 0 | 0 | 0 | 0 | 0.063 | 0 | 0 | 0.698 | 0.705 |
| F3 | IL6 | 9606.ENSP00000334145 | 9606.ENSP00000385675 | 0 | 0 | 0 | 0 | 0.088 | 0 | 0 | 0.705 | 0.719 |
| F3 | THBD | 9606.ENSP00000334145 | 9606.ENSP00000366307 | 0 | 0 | 0 | 0 | 0.062 | 0 | 0 | 0.864 | 0.867 |
| F3 | F10 | 9606.ENSP00000334145 | 9606.ENSP00000364709 | 0 | 0 | 0 | 0 | 0 | 0.213 | 0.6 | 0.988 | 0.996 |
| F3 | F7 | 9606.ENSP00000334145 | 9606.ENSP00000364731 | 0 | 0 | 0 | 0 | 0 | 0.978 | 0.9 | 0.99 | 0.999 |
| F7 | PLAT | 9606.ENSP00000364731 | 9606.ENSP00000220809 | 0 | 0 | 0.416 | 0.62 | 0 | 0.091 | 0 | 0.703 | 0.429 |
| F7 | EGF | 9606.ENSP00000364731 | 9606.ENSP00000265171 | 0 | 0 | 0 | 0 | 0 | 0.056 | 0 | 0.517 | 0.525 |
| F7 | F2 | 9606.ENSP00000364731 | 9606.ENSP00000308541 | 0 | 0 | 0.376 | 0.705 | 0.265 | 0 | 0 | 0.956 | 0.526 |
| F7 | F3 | 9606.ENSP00000364731 | 9606.ENSP00000334145 | 0 | 0 | 0 | 0 | 0 | 0.978 | 0.9 | 0.99 | 0.999 |
| F7 | F10 | 9606.ENSP00000364731 | 9606.ENSP00000364709 | 0 | 0 | 0.423 | 0.897 | 0.419 | 0.27 | 0.8 | 0.927 | 0.92 |
| F7 | TNF | 9606.ENSP00000364731 | 9606.ENSP00000398698 | 0 | 0 | 0 | 0 | 0.062 | 0 | 0 | 0.392 | 0.405 |
| F7 | IL6 | 9606.ENSP00000364731 | 9606.ENSP00000385675 | 0 | 0 | 0 | 0 | 0 | 0 | 0 | 0.469 | 0.469 |
| F7 | THBD | 9606.ENSP00000364731 | 9606.ENSP00000366307 | 0 | 0 | 0 | 0 | 0 | 0 | 0 | 0.681 | 0.681 |
| FASN | CAT | 9606.ENSP00000304592 | 9606.ENSP00000241052 | 0 | 0 | 0 | 0 | 0 | 0.143 | 0 | 0.346 | 0.415 |
| FASN | MAPK3 | 9606.ENSP00000304592 | 9606.ENSP00000263025 | 0 | 0 | 0 | 0 | 0 | 0.27 | 0 | 0.343 | 0.499 |
| FASN | TP53 | 9606.ENSP00000304592 | 9606.ENSP00000269305 | 0 | 0 | 0 | 0 | 0 | 0 | 0 | 0.464 | 0.463 |
| FASN | EGFR | 9606.ENSP00000304592 | 9606.ENSP00000275493 | 0 | 0 | 0 | 0 | 0.066 | 0 | 0 | 0.583 | 0.595 |
| FASN | PPARG | 9606.ENSP00000304592 | 9606.ENSP00000287820 | 0 | 0 | 0 | 0 | 0 | 0 | 0 | 0.829 | 0.829 |
| FASN | HMGCR | 9606.ENSP00000304592 | 9606.ENSP00000287936 | 0 | 0 | 0 | 0 | 0.106 | 0 | 0 | 0.832 | 0.843 |
| FASN | INSR | 9606.ENSP00000304592 | 9606.ENSP00000303830 | 0 | 0 | 0 | 0 | 0 | 0 | 0 | 0.428 | 0.428 |
| FASN | ESR1 | 9606.ENSP00000304592 | 9606.ENSP00000405330 | 0 | 0 | 0 | 0 | 0 | 0.072 | 0 | 0.391 | 0.41 |
| FASN | AR | 9606.ENSP00000304592 | 9606.ENSP00000363822 | 0 | 0 | 0 | 0 | 0 | 0 | 0 | 0.42 | 0.42 |
| FASN | GSK3B | 9606.ENSP00000304592 | 9606.ENSP00000324806 | 0 | 0 | 0 | 0 | 0 | 0.105 | 0 | 0.396 | 0.436 |
| FASN | TNF | 9606.ENSP00000304592 | 9606.ENSP00000398698 | 0 | 0 | 0 | 0 | 0 | 0 | 0 | 0.438 | 0.438 |
| FASN | PPARD | 9606.ENSP00000304592 | 9606.ENSP00000310928 | 0 | 0 | 0 | 0 | 0.062 | 0 | 0 | 0.432 | 0.445 |
| FASN | IL6 | 9606.ENSP00000304592 | 9606.ENSP00000385675 | 0 | 0 | 0 | 0 | 0 | 0 | 0 | 0.459 | 0.459 |
| FASN | LDLR | 9606.ENSP00000304592 | 9606.ENSP00000454071 | 0 | 0 | 0 | 0 | 0.085 | 0.068 | 0 | 0.569 | 0.601 |
| FASN | RXRB | 9606.ENSP00000304592 | 9606.ENSP00000363817 | 0 | 0 | 0 | 0 | 0.065 | 0 | 0.9 | 0.124 | 0.91 |
| FASN | RXRA | 9606.ENSP00000304592 | 9606.ENSP00000419692 | 0 | 0 | 0 | 0 | 0.062 | 0 | 0.9 | 0.331 | 0.931 |
| FASN | ACACA | 9606.ENSP00000304592 | 9606.ENSP00000483300 | 0.056 | 0 | 0 | 0 | 0.684 | 0.068 | 0.9 | 0.922 | 0.997 |
| GJA1 | MAPK1 | 9606.ENSP00000282561 | 9606.ENSP00000215832 | 0 | 0 | 0 | 0 | 0 | 0.462 | 0.8 | 0.209 | 0.907 |
| GJA1 | MMP2 | 9606.ENSP00000282561 | 9606.ENSP00000219070 | 0 | 0 | 0 | 0 | 0.229 | 0 | 0 | 0.45 | 0.557 |
| GJA1 | CCL2 | 9606.ENSP00000282561 | 9606.ENSP00000225831 | 0 | 0 | 0 | 0 | 0.086 | 0 | 0 | 0.413 | 0.44 |
| GJA1 | KCNH2 | 9606.ENSP00000282561 | 9606.ENSP00000262186 | 0 | 0 | 0 | 0 | 0.062 | 0 | 0 | 0.51 | 0.52 |
| GJA1 | MAPK3 | 9606.ENSP00000282561 | 9606.ENSP00000263025 | 0 | 0 | 0 | 0 | 0 | 0.213 | 0.8 | 0.501 | 0.914 |
| GJA1 | IL1B | 9606.ENSP00000282561 | 9606.ENSP00000263341 | 0 | 0 | 0 | 0 | 0 | 0 | 0 | 0.519 | 0.519 |
| GJA1 | KDR | 9606.ENSP00000282561 | 9606.ENSP00000263923 | 0 | 0 | 0 | 0 | 0.188 | 0 | 0 | 0.414 | 0.504 |
| GJA1 | EGF | 9606.ENSP00000282561 | 9606.ENSP00000265171 | 0 | 0 | 0 | 0 | 0.062 | 0 | 0 | 0.503 | 0.513 |
| GJA1 | CDK2 | 9606.ENSP00000282561 | 9606.ENSP00000266970 | 0 | 0 | 0 | 0 | 0 | 0 | 0 | 0.705 | 0.706 |
| GJA1 | TP53 | 9606.ENSP00000282561 | 9606.ENSP00000269305 | 0 | 0 | 0 | 0 | 0 | 0 | 0 | 0.413 | 0.412 |
| GJA1 | EGFR | 9606.ENSP00000282561 | 9606.ENSP00000275493 | 0 | 0 | 0 | 0 | 0.158 | 0 | 0 | 0.39 | 0.464 |
| GJA1 | VCAM1 | 9606.ENSP00000282561 | 9606.ENSP00000294728 | 0 | 0 | 0 | 0 | 0.076 | 0 | 0 | 0.401 | 0.423 |
| GJA1 | ESR1 | 9606.ENSP00000282561 | 9606.ENSP00000405330 | 0 | 0 | 0 | 0 | 0 | 0 | 0 | 0.424 | 0.424 |
| GJA1 | JUN | 9606.ENSP00000282561 | 9606.ENSP00000360266 | 0 | 0 | 0 | 0 | 0 | 0 | 0 | 0.45 | 0.45 |
| GJA1 | PTGS2 | 9606.ENSP00000282561 | 9606.ENSP00000356438 | 0 | 0 | 0 | 0 | 0 | 0 | 0 | 0.459 | 0.459 |
| GJA1 | NOS3 | 9606.ENSP00000282561 | 9606.ENSP00000297494 | 0 | 0 | 0 | 0 | 0 | 0 | 0 | 0.518 | 0.518 |
| GJA1 | TNF | 9606.ENSP00000282561 | 9606.ENSP00000398698 | 0 | 0 | 0 | 0 | 0 | 0 | 0 | 0.535 | 0.535 |
| GJA1 | VEGFA | 9606.ENSP00000282561 | 9606.ENSP00000478570 | 0 | 0 | 0 | 0 | 0 | 0 | 0 | 0.56 | 0.56 |
| GJA1 | IL6 | 9606.ENSP00000282561 | 9606.ENSP00000385675 | 0 | 0 | 0 | 0 | 0 | 0 | 0 | 0.609 | 0.609 |
| GJA1 | CDK1 | 9606.ENSP00000282561 | 9606.ENSP00000378699 | 0 | 0 | 0 | 0 | 0 | 0 | 0.8 | 0.235 | 0.84 |
| GSK3B | MAPK1 | 9606.ENSP00000324806 | 9606.ENSP00000215832 | 0 | 0 | 0.265 | 0.773 | 0.288 | 0.351 | 0 | 0.542 | 0.598 |
| GSK3B | HMOX1 | 9606.ENSP00000324806 | 9606.ENSP00000216117 | 0 | 0 | 0 | 0 | 0 | 0.056 | 0 | 0.46 | 0.468 |
| GSK3B | MMP2 | 9606.ENSP00000324806 | 9606.ENSP00000219070 | 0 | 0 | 0 | 0 | 0 | 0 | 0 | 0.413 | 0.413 |
| GSK3B | NR3C1 | 9606.ENSP00000324806 | 9606.ENSP00000231509 | 0 | 0 | 0 | 0 | 0 | 0.077 | 0.9 | 0.321 | 0.931 |
| GSK3B | CAT | 9606.ENSP00000324806 | 9606.ENSP00000241052 | 0.044 | 0 | 0 | 0 | 0.062 | 0.144 | 0 | 0.429 | 0.504 |
| GSK3B | CDK4 | 9606.ENSP00000324806 | 9606.ENSP00000257904 | 0 | 0 | 0.347 | 0.735 | 0.064 | 0.061 | 0.9 | 0.497 | 0.923 |
| GSK3B | IL1B | 9606.ENSP00000324806 | 9606.ENSP00000263341 | 0 | 0 | 0 | 0 | 0 | 0.056 | 0 | 0.495 | 0.503 |
| GSK3B | EGF | 9606.ENSP00000324806 | 9606.ENSP00000265171 | 0 | 0 | 0 | 0 | 0 | 0.056 | 0 | 0.602 | 0.609 |
| GSK3B | TP53 | 9606.ENSP00000324806 | 9606.ENSP00000269305 | 0 | 0 | 0 | 0 | 0 | 0.866 | 0.9 | 0.973 | 0.999 |
| GSK3B | PPARG | 9606.ENSP00000324806 | 9606.ENSP00000287820 | 0 | 0 | 0 | 0 | 0 | 0.077 | 0 | 0.612 | 0.626 |
| GSK3B | BAX | 9606.ENSP00000324806 | 9606.ENSP00000293288 | 0 | 0 | 0 | 0 | 0.059 | 0.27 | 0 | 0.301 | 0.477 |
| GSK3B | NOS3 | 9606.ENSP00000324806 | 9606.ENSP00000297494 | 0 | 0 | 0 | 0 | 0.062 | 0.077 | 0 | 0.464 | 0.495 |
| GSK3B | ACHE | 9606.ENSP00000324806 | 9606.ENSP00000303211 | 0 | 0 | 0 | 0 | 0.048 | 0.062 | 0 | 0.408 | 0.425 |
| GSK3B | INSR | 9606.ENSP00000324806 | 9606.ENSP00000303830 | 0 | 0 | 0 | 0 | 0 | 0.056 | 0 | 0.399 | 0.409 |
| GSK3B | FASN | 9606.ENSP00000324806 | 9606.ENSP00000304592 | 0 | 0 | 0 | 0 | 0 | 0.105 | 0 | 0.396 | 0.436 |
| GSK3B | PRKACA | 9606.ENSP00000324806 | 9606.ENSP00000309591 | 0 | 0 | 0.251 | 0.625 | 0.079 | 0.721 | 0.8 | 0.276 | 0.953 |
| GSK3B | HSPA5 | 9606.ENSP00000324806 | 9606.ENSP00000324173 | 0 | 0 | 0 | 0 | 0.062 | 0.371 | 0 | 0.335 | 0.574 |
| GSK3B | MAP2 | 9606.ENSP00000324806 | 9606.ENSP00000353508 | 0 | 0 | 0 | 0 | 0.062 | 0.181 | 0 | 0.36 | 0.465 |
| GSK3B | LDLR | 9606.ENSP00000324806 | 9606.ENSP00000454071 | 0 | 0 | 0 | 0 | 0 | 0.056 | 0 | 0.466 | 0.475 |
| GSK3B | PTGS2 | 9606.ENSP00000324806 | 9606.ENSP00000356438 | 0 | 0 | 0 | 0 | 0 | 0.077 | 0 | 0.477 | 0.497 |
| GSK3B | VEGFA | 9606.ENSP00000324806 | 9606.ENSP00000478570 | 0 | 0 | 0 | 0 | 0 | 0 | 0 | 0.504 | 0.504 |
| GSK3B | PTPN1 | 9606.ENSP00000324806 | 9606.ENSP00000360683 | 0 | 0 | 0 | 0 | 0.062 | 0.298 | 0 | 0.33 | 0.521 |
| GSK3B | IL6 | 9606.ENSP00000324806 | 9606.ENSP00000385675 | 0 | 0 | 0 | 0 | 0 | 0 | 0 | 0.581 | 0.581 |
| GSK3B | TNF | 9606.ENSP00000324806 | 9606.ENSP00000398698 | 0 | 0 | 0 | 0 | 0 | 0.056 | 0 | 0.58 | 0.587 |
| GSK3B | ESR1 | 9606.ENSP00000324806 | 9606.ENSP00000405330 | 0 | 0 | 0 | 0 | 0 | 0.077 | 0 | 0.618 | 0.633 |
| GSK3B | PPP3CA | 9606.ENSP00000324806 | 9606.ENSP00000378323 | 0 | 0 | 0 | 0 | 0.083 | 0.244 | 0.8 | 0.379 | 0.902 |
| GSK3B | AR | 9606.ENSP00000324806 | 9606.ENSP00000363822 | 0 | 0 | 0 | 0 | 0 | 0.482 | 0.9 | 0.405 | 0.966 |
| GSK3B | JUN | 9606.ENSP00000324806 | 9606.ENSP00000360266 | 0 | 0 | 0 | 0 | 0 | 0.691 | 0.9 | 0.709 | 0.99 |
| GSR | HMOX1 | 9606.ENSP00000221130 | 9606.ENSP00000216117 | 0 | 0 | 0 | 0 | 0.083 | 0.098 | 0 | 0.742 | 0.768 |
| GSR | ACHE | 9606.ENSP00000221130 | 9606.ENSP00000303211 | 0 | 0 | 0 | 0 | 0 | 0 | 0 | 0.4 | 0.4 |
| GSR | PPARG | 9606.ENSP00000221130 | 9606.ENSP00000287820 | 0 | 0 | 0 | 0 | 0 | 0.057 | 0 | 0.392 | 0.402 |
| GSR | PON1 | 9606.ENSP00000221130 | 9606.ENSP00000222381 | 0 | 0 | 0 | 0 | 0 | 0 | 0 | 0.404 | 0.404 |
| GSR | MAPK3 | 9606.ENSP00000221130 | 9606.ENSP00000263025 | 0 | 0 | 0 | 0 | 0.067 | 0 | 0 | 0.395 | 0.412 |
| GSR | AKR1C1 | 9606.ENSP00000221130 | 9606.ENSP00000370254 | 0 | 0 | 0 | 0 | 0.062 | 0.057 | 0 | 0.414 | 0.436 |
| GSR | ABCC1 | 9606.ENSP00000221130 | 9606.ENSP00000382342 | 0 | 0 | 0 | 0 | 0.094 | 0.137 | 0 | 0.385 | 0.477 |
| GSR | JUN | 9606.ENSP00000221130 | 9606.ENSP00000360266 | 0 | 0 | 0 | 0 | 0 | 0.129 | 0 | 0.458 | 0.507 |
| GSR | TP53 | 9606.ENSP00000221130 | 9606.ENSP00000269305 | 0 | 0 | 0 | 0 | 0 | 0 | 0 | 0.508 | 0.508 |
| GSR | XDH | 9606.ENSP00000221130 | 9606.ENSP00000368727 | 0 | 0 | 0 | 0 | 0 | 0 | 0 | 0.516 | 0.516 |
| GSR | NOS3 | 9606.ENSP00000221130 | 9606.ENSP00000297494 | 0 | 0 | 0 | 0 | 0 | 0 | 0 | 0.525 | 0.525 |
| GSR | PTGS2 | 9606.ENSP00000221130 | 9606.ENSP00000356438 | 0 | 0 | 0 | 0 | 0 | 0.166 | 0 | 0.478 | 0.546 |
| GSR | TNF | 9606.ENSP00000221130 | 9606.ENSP00000398698 | 0 | 0 | 0 | 0 | 0 | 0 | 0 | 0.55 | 0.55 |
| GSR | IL1B | 9606.ENSP00000221130 | 9606.ENSP00000263341 | 0 | 0 | 0 | 0 | 0 | 0 | 0 | 0.556 | 0.556 |
| GSR | MPO | 9606.ENSP00000221130 | 9606.ENSP00000225275 | 0 | 0 | 0 | 0 | 0 | 0.166 | 0 | 0.49 | 0.556 |
| GSR | IL6 | 9606.ENSP00000221130 | 9606.ENSP00000385675 | 0 | 0 | 0 | 0 | 0 | 0 | 0 | 0.563 | 0.563 |
| GSR | NQO1 | 9606.ENSP00000221130 | 9606.ENSP00000319788 | 0 | 0 | 0 | 0 | 0.064 | 0 | 0 | 0.73 | 0.736 |
| GSR | GSTP1 | 9606.ENSP00000221130 | 9606.ENSP00000381607 | 0 | 0 | 0 | 0 | 0.064 | 0 | 0.65 | 0.58 | 0.85 |
| GSR | SOD1 | 9606.ENSP00000221130 | 9606.ENSP00000270142 | 0 | 0 | 0 | 0 | 0.073 | 0.117 | 0 | 0.84 | 0.858 |
| GSR | CAT | 9606.ENSP00000221130 | 9606.ENSP00000241052 | 0 | 0 | 0 | 0 | 0.218 | 0.26 | 0 | 0.953 | 0.97 |
| GSTP1 | HMOX1 | 9606.ENSP00000381607 | 9606.ENSP00000216117 | 0 | 0 | 0 | 0 | 0 | 0 | 0 | 0.556 | 0.556 |
| GSTP1 | GSR | 9606.ENSP00000381607 | 9606.ENSP00000221130 | 0 | 0 | 0 | 0 | 0.064 | 0 | 0.65 | 0.58 | 0.85 |
| GSTP1 | PON1 | 9606.ENSP00000381607 | 9606.ENSP00000222381 | 0 | 0 | 0 | 0 | 0.062 | 0 | 0 | 0.402 | 0.415 |
| GSTP1 | MPO | 9606.ENSP00000381607 | 9606.ENSP00000225275 | 0 | 0 | 0 | 0 | 0 | 0 | 0 | 0.421 | 0.42 |
| GSTP1 | CAT | 9606.ENSP00000381607 | 9606.ENSP00000241052 | 0 | 0 | 0 | 0 | 0.053 | 0.178 | 0 | 0.535 | 0.606 |
| GSTP1 | AHR | 9606.ENSP00000381607 | 9606.ENSP00000242057 | 0 | 0 | 0 | 0 | 0 | 0 | 0 | 0.477 | 0.477 |
| GSTP1 | TP53 | 9606.ENSP00000381607 | 9606.ENSP00000269305 | 0 | 0 | 0 | 0 | 0 | 0 | 0 | 0.665 | 0.665 |
| GSTP1 | SOD1 | 9606.ENSP00000381607 | 9606.ENSP00000270142 | 0 | 0 | 0 | 0 | 0.063 | 0.129 | 0 | 0.47 | 0.53 |
| GSTP1 | EGFR | 9606.ENSP00000381607 | 9606.ENSP00000275493 | 0 | 0 | 0 | 0 | 0.068 | 0.487 | 0 | 0.422 | 0.699 |
| GSTP1 | ADH1B | 9606.ENSP00000381607 | 9606.ENSP00000306606 | 0 | 0 | 0 | 0 | 0 | 0.166 | 0.65 | 0.325 | 0.785 |
| GSTP1 | NQO1 | 9606.ENSP00000381607 | 9606.ENSP00000319788 | 0 | 0 | 0 | 0 | 0.065 | 0 | 0 | 0.714 | 0.721 |
| GSTP1 | PGR | 9606.ENSP00000381607 | 9606.ENSP00000325120 | 0 | 0 | 0 | 0 | 0 | 0.062 | 0 | 0.394 | 0.407 |
| GSTP1 | CYP3A4 | 9606.ENSP00000381607 | 9606.ENSP00000337915 | 0 | 0 | 0 | 0 | 0 | 0.056 | 0.65 | 0.563 | 0.843 |
| GSTP1 | CYP1A2 | 9606.ENSP00000381607 | 9606.ENSP00000342007 | 0 | 0 | 0 | 0 | 0 | 0.057 | 0.65 | 0.563 | 0.843 |
| GSTP1 | ESR2 | 9606.ENSP00000381607 | 9606.ENSP00000343925 | 0 | 0 | 0 | 0 | 0 | 0.062 | 0 | 0.391 | 0.404 |
| GSTP1 | PTGS2 | 9606.ENSP00000381607 | 9606.ENSP00000356438 | 0 | 0 | 0 | 0 | 0 | 0 | 0 | 0.501 | 0.501 |
| GSTP1 | JUN | 9606.ENSP00000381607 | 9606.ENSP00000360266 | 0 | 0 | 0 | 0 | 0 | 0.094 | 0 | 0.985 | 0.986 |
| GSTP1 | AR | 9606.ENSP00000381607 | 9606.ENSP00000363822 | 0 | 0 | 0 | 0 | 0 | 0.062 | 0 | 0.456 | 0.467 |
| GSTP1 | MAPK8 | 9606.ENSP00000381607 | 9606.ENSP00000378974 | 0 | 0 | 0 | 0 | 0 | 0.757 | 0 | 0.982 | 0.995 |
| GSTP1 | ABCC1 | 9606.ENSP00000381607 | 9606.ENSP00000382342 | 0 | 0 | 0 | 0 | 0.054 | 0 | 0 | 0.483 | 0.49 |
| GSTP1 | ESR1 | 9606.ENSP00000381607 | 9606.ENSP00000405330 | 0 | 0 | 0 | 0 | 0 | 0.062 | 0 | 0.56 | 0.569 |
| GSTP1 | HTR2A | 9606.ENSP00000381607 | 9606.ENSP00000437737 | 0 | 0 | 0 | 0 | 0 | 0.063 | 0 | 0.565 | 0.575 |
| HMGCR | PPARG | 9606.ENSP00000287936 | 9606.ENSP00000287820 | 0 | 0 | 0 | 0 | 0.062 | 0 | 0 | 0.568 | 0.577 |
| HMGCR | TNF | 9606.ENSP00000287936 | 9606.ENSP00000398698 | 0 | 0 | 0 | 0 | 0 | 0 | 0 | 0.415 | 0.415 |
| HMGCR | IL6 | 9606.ENSP00000287936 | 9606.ENSP00000385675 | 0 | 0 | 0 | 0 | 0 | 0 | 0 | 0.455 | 0.455 |
| HMGCR | HSPA5 | 9606.ENSP00000287936 | 9606.ENSP00000324173 | 0 | 0 | 0 | 0 | 0.062 | 0.059 | 0 | 0.443 | 0.465 |
| HMGCR | CYP3A4 | 9606.ENSP00000287936 | 9606.ENSP00000337915 | 0 | 0 | 0 | 0 | 0.064 | 0 | 0 | 0.502 | 0.514 |
| HMGCR | NOS3 | 9606.ENSP00000287936 | 9606.ENSP00000297494 | 0 | 0 | 0 | 0 | 0.049 | 0 | 0 | 0.525 | 0.529 |
| HMGCR | SOAT1 | 9606.ENSP00000287936 | 9606.ENSP00000356591 | 0 | 0 | 0 | 0 | 0 | 0 | 0 | 0.639 | 0.639 |
| HMGCR | ACACA | 9606.ENSP00000287936 | 9606.ENSP00000483300 | 0.05 | 0 | 0 | 0 | 0.069 | 0 | 0 | 0.768 | 0.777 |
| HMGCR | LDLR | 9606.ENSP00000287936 | 9606.ENSP00000454071 | 0 | 0 | 0 | 0 | 0.165 | 0.062 | 0 | 0.815 | 0.842 |
| HMGCR | FASN | 9606.ENSP00000287936 | 9606.ENSP00000304592 | 0 | 0 | 0 | 0 | 0.106 | 0 | 0 | 0.832 | 0.843 |
| HMGCR | NCOA2 | 9606.ENSP00000287936 | 9606.ENSP00000399968 | 0 | 0 | 0 | 0 | 0 | 0 | 0.9 | 0.078 | 0.903 |
| HMGCR | NCOA1 | 9606.ENSP00000287936 | 9606.ENSP00000385216 | 0 | 0 | 0 | 0 | 0 | 0 | 0.9 | 0.107 | 0.906 |
| HMGCR | RXRA | 9606.ENSP00000287936 | 9606.ENSP00000419692 | 0 | 0 | 0 | 0 | 0.062 | 0 | 0.9 | 0.313 | 0.929 |
| HMOX1 | EGF | 9606.ENSP00000216117 | 9606.ENSP00000265171 | 0 | 0 | 0 | 0 | 0 | 0 | 0 | 0.4 | 0.4 |
| HMOX1 | THBD | 9606.ENSP00000216117 | 9606.ENSP00000366307 | 0 | 0 | 0 | 0 | 0.088 | 0 | 0 | 0.375 | 0.405 |
| HMOX1 | KCNMA1 | 9606.ENSP00000216117 | 9606.ENSP00000286628 | 0 | 0 | 0 | 0 | 0 | 0 | 0 | 0.409 | 0.409 |
| HMOX1 | MMP3 | 9606.ENSP00000216117 | 9606.ENSP00000299855 | 0 | 0 | 0 | 0 | 0 | 0 | 0 | 0.413 | 0.413 |
| HMOX1 | IL2 | 9606.ENSP00000216117 | 9606.ENSP00000226730 | 0 | 0 | 0 | 0 | 0 | 0 | 0 | 0.414 | 0.414 |
| HMOX1 | ALOX5 | 9606.ENSP00000216117 | 9606.ENSP00000363512 | 0 | 0 | 0 | 0 | 0.06 | 0 | 0 | 0.404 | 0.415 |
| HMOX1 | MMP1 | 9606.ENSP00000216117 | 9606.ENSP00000322788 | 0 | 0 | 0 | 0 | 0.068 | 0 | 0 | 0.415 | 0.431 |
| HMOX1 | GSK3B | 9606.ENSP00000216117 | 9606.ENSP00000324806 | 0 | 0 | 0 | 0 | 0 | 0.056 | 0 | 0.46 | 0.468 |
| HMOX1 | AKR1C1 | 9606.ENSP00000216117 | 9606.ENSP00000370254 | 0 | 0 | 0 | 0 | 0 | 0 | 0 | 0.47 | 0.47 |
| HMOX1 | IFNG | 9606.ENSP00000216117 | 9606.ENSP00000229135 | 0 | 0 | 0 | 0 | 0 | 0 | 0 | 0.475 | 0.475 |
| HMOX1 | MMP2 | 9606.ENSP00000216117 | 9606.ENSP00000219070 | 0 | 0 | 0 | 0 | 0 | 0 | 0 | 0.486 | 0.486 |
| HMOX1 | MAPK8 | 9606.ENSP00000216117 | 9606.ENSP00000378974 | 0 | 0 | 0 | 0 | 0 | 0 | 0 | 0.503 | 0.503 |
| HMOX1 | SELE | 9606.ENSP00000216117 | 9606.ENSP00000331736 | 0 | 0 | 0 | 0 | 0 | 0 | 0 | 0.509 | 0.509 |
| HMOX1 | BAX | 9606.ENSP00000216117 | 9606.ENSP00000293288 | 0 | 0 | 0 | 0 | 0.063 | 0 | 0 | 0.503 | 0.514 |
| HMOX1 | AHR | 9606.ENSP00000216117 | 9606.ENSP00000242057 | 0 | 0 | 0 | 0 | 0.062 | 0 | 0 | 0.51 | 0.52 |
| HMOX1 | GSTP1 | 9606.ENSP00000216117 | 9606.ENSP00000381607 | 0 | 0 | 0 | 0 | 0 | 0 | 0 | 0.556 | 0.556 |
| HMOX1 | MAPK3 | 9606.ENSP00000216117 | 9606.ENSP00000263025 | 0 | 0 | 0 | 0 | 0 | 0 | 0 | 0.611 | 0.611 |
| HMOX1 | TP53 | 9606.ENSP00000216117 | 9606.ENSP00000269305 | 0 | 0 | 0 | 0 | 0.062 | 0 | 0 | 0.611 | 0.619 |
| HMOX1 | VCAM1 | 9606.ENSP00000216117 | 9606.ENSP00000294728 | 0 | 0 | 0 | 0 | 0.062 | 0 | 0 | 0.616 | 0.624 |
| HMOX1 | HSPA5 | 9606.ENSP00000216117 | 9606.ENSP00000324173 | 0 | 0 | 0 | 0 | 0 | 0 | 0 | 0.636 | 0.637 |
| HMOX1 | MPO | 9606.ENSP00000216117 | 9606.ENSP00000225275 | 0 | 0 | 0 | 0 | 0 | 0 | 0 | 0.652 | 0.652 |
| HMOX1 | PPARG | 9606.ENSP00000216117 | 9606.ENSP00000287820 | 0 | 0 | 0 | 0 | 0 | 0 | 0 | 0.654 | 0.654 |
| HMOX1 | VEGFA | 9606.ENSP00000216117 | 9606.ENSP00000478570 | 0 | 0 | 0 | 0 | 0 | 0 | 0 | 0.683 | 0.683 |
| HMOX1 | CCL2 | 9606.ENSP00000216117 | 9606.ENSP00000225831 | 0 | 0 | 0 | 0 | 0.095 | 0 | 0 | 0.668 | 0.686 |
| HMOX1 | SOD1 | 9606.ENSP00000216117 | 9606.ENSP00000270142 | 0 | 0 | 0 | 0 | 0 | 0 | 0 | 0.708 | 0.708 |
| HMOX1 | NOS2 | 9606.ENSP00000216117 | 9606.ENSP00000327251 | 0 | 0 | 0 | 0 | 0 | 0.177 | 0 | 0.667 | 0.714 |
| HMOX1 | PTGS2 | 9606.ENSP00000216117 | 9606.ENSP00000356438 | 0 | 0 | 0 | 0 | 0.076 | 0 | 0 | 0.745 | 0.754 |
| HMOX1 | IL1B | 9606.ENSP00000216117 | 9606.ENSP00000263341 | 0 | 0 | 0 | 0 | 0.07 | 0 | 0 | 0.76 | 0.767 |
| HMOX1 | GSR | 9606.ENSP00000216117 | 9606.ENSP00000221130 | 0 | 0 | 0 | 0 | 0.083 | 0.098 | 0 | 0.742 | 0.768 |
| HMOX1 | TNF | 9606.ENSP00000216117 | 9606.ENSP00000398698 | 0 | 0 | 0 | 0 | 0 | 0 | 0 | 0.775 | 0.775 |
| HMOX1 | NOS3 | 9606.ENSP00000216117 | 9606.ENSP00000297494 | 0 | 0 | 0 | 0 | 0 | 0.177 | 0 | 0.752 | 0.787 |
| HMOX1 | IL6 | 9606.ENSP00000216117 | 9606.ENSP00000385675 | 0 | 0 | 0 | 0 | 0.073 | 0 | 0 | 0.78 | 0.787 |
| HMOX1 | CAT | 9606.ENSP00000216117 | 9606.ENSP00000241052 | 0 | 0 | 0 | 0 | 0.062 | 0.101 | 0 | 0.882 | 0.892 |
| HMOX1 | POR | 9606.ENSP00000216117 | 9606.ENSP00000419970 | 0 | 0 | 0 | 0 | 0.072 | 0.324 | 0 | 0.865 | 0.908 |
| HMOX1 | NQO1 | 9606.ENSP00000216117 | 9606.ENSP00000319788 | 0 | 0 | 0 | 0 | 0.058 | 0 | 0 | 0.915 | 0.916 |
| HMOX1 | MAPK14 | 9606.ENSP00000216117 | 9606.ENSP00000229795 | 0 | 0 | 0 | 0 | 0 | 0 | 0.9 | 0.407 | 0.938 |
| HMOX1 | JUN | 9606.ENSP00000216117 | 9606.ENSP00000360266 | 0 | 0 | 0 | 0 | 0 | 0 | 0.9 | 0.692 | 0.967 |
| HSPA5 | HMOX1 | 9606.ENSP00000324173 | 9606.ENSP00000216117 | 0 | 0 | 0 | 0 | 0 | 0 | 0 | 0.636 | 0.637 |
| HSPA5 | PLAT | 9606.ENSP00000324173 | 9606.ENSP00000220809 | 0 | 0 | 0 | 0 | 0 | 0.058 | 0 | 0.778 | 0.782 |
| HSPA5 | MAPK14 | 9606.ENSP00000324173 | 9606.ENSP00000229795 | 0 | 0 | 0 | 0 | 0.062 | 0.104 | 0 | 0.452 | 0.5 |
| HSPA5 | CTSD | 9606.ENSP00000324173 | 9606.ENSP00000236671 | 0 | 0 | 0 | 0 | 0.062 | 0.153 | 0 | 0.565 | 0.624 |
| HSPA5 | CAT | 9606.ENSP00000324173 | 9606.ENSP00000241052 | 0 | 0 | 0 | 0 | 0.062 | 0 | 0 | 0.618 | 0.626 |
| HSPA5 | MAPK3 | 9606.ENSP00000324173 | 9606.ENSP00000263025 | 0 | 0 | 0 | 0 | 0.062 | 0.288 | 0 | 0.63 | 0.731 |
| HSPA5 | IL1B | 9606.ENSP00000324173 | 9606.ENSP00000263341 | 0 | 0 | 0 | 0 | 0 | 0 | 0 | 0.506 | 0.506 |
| HSPA5 | EGF | 9606.ENSP00000324173 | 9606.ENSP00000265171 | 0 | 0 | 0 | 0 | 0 | 0 | 0 | 0.497 | 0.497 |
| HSPA5 | TP53 | 9606.ENSP00000324173 | 9606.ENSP00000269305 | 0 | 0 | 0 | 0 | 0 | 0.428 | 0 | 0.822 | 0.894 |
| HSPA5 | SOD1 | 9606.ENSP00000324173 | 9606.ENSP00000270142 | 0 | 0 | 0 | 0 | 0 | 0.149 | 0.8 | 0.722 | 0.948 |
| HSPA5 | EGFR | 9606.ENSP00000324173 | 9606.ENSP00000275493 | 0 | 0 | 0 | 0 | 0 | 0.682 | 0 | 0.955 | 0.985 |
| HSPA5 | PPARG | 9606.ENSP00000324173 | 9606.ENSP00000287820 | 0 | 0 | 0 | 0 | 0 | 0.066 | 0 | 0.393 | 0.409 |
| HSPA5 | HMGCR | 9606.ENSP00000324173 | 9606.ENSP00000287936 | 0 | 0 | 0 | 0 | 0.062 | 0.059 | 0 | 0.443 | 0.465 |
| HSPA5 | BAX | 9606.ENSP00000324173 | 9606.ENSP00000293288 | 0 | 0 | 0 | 0 | 0 | 0 | 0 | 0.459 | 0.459 |
| HSPA5 | NOS3 | 9606.ENSP00000324173 | 9606.ENSP00000297494 | 0 | 0 | 0 | 0 | 0.053 | 0.056 | 0 | 0.398 | 0.415 |
| HSPA5 | NQO1 | 9606.ENSP00000324173 | 9606.ENSP00000319788 | 0 | 0 | 0 | 0 | 0.065 | 0.104 | 0 | 0.36 | 0.417 |
| HSPA5 | AR | 9606.ENSP00000324173 | 9606.ENSP00000363822 | 0 | 0 | 0 | 0 | 0 | 0.182 | 0 | 0.301 | 0.403 |
| HSPA5 | PRSS3 | 9606.ENSP00000324173 | 9606.ENSP00000354280 | 0 | 0 | 0 | 0 | 0 | 0.058 | 0 | 0.435 | 0.445 |
| HSPA5 | PTPN1 | 9606.ENSP00000324173 | 9606.ENSP00000360683 | 0 | 0 | 0 | 0 | 0.062 | 0 | 0 | 0.447 | 0.459 |
| HSPA5 | F3 | 9606.ENSP00000324173 | 9606.ENSP00000334145 | 0 | 0 | 0 | 0 | 0 | 0 | 0 | 0.46 | 0.46 |
| HSPA5 | VEGFA | 9606.ENSP00000324173 | 9606.ENSP00000478570 | 0 | 0 | 0 | 0 | 0 | 0 | 0 | 0.467 | 0.467 |
| HSPA5 | IL6 | 9606.ENSP00000324173 | 9606.ENSP00000385675 | 0 | 0 | 0 | 0 | 0 | 0 | 0 | 0.527 | 0.527 |
| HSPA5 | MAPK8 | 9606.ENSP00000324173 | 9606.ENSP00000378974 | 0 | 0 | 0 | 0 | 0 | 0 | 0 | 0.531 | 0.531 |
| HSPA5 | ESR1 | 9606.ENSP00000324173 | 9606.ENSP00000405330 | 0 | 0 | 0 | 0 | 0 | 0.309 | 0 | 0.404 | 0.571 |
| HSPA5 | GSK3B | 9606.ENSP00000324173 | 9606.ENSP00000324806 | 0 | 0 | 0 | 0 | 0.062 | 0.371 | 0 | 0.335 | 0.574 |
| HSPA5 | TNF | 9606.ENSP00000324173 | 9606.ENSP00000398698 | 0 | 0 | 0 | 0 | 0 | 0.123 | 0 | 0.585 | 0.621 |
| HSPA5 | JUN | 9606.ENSP00000324173 | 9606.ENSP00000360266 | 0 | 0 | 0 | 0 | 0.062 | 0.111 | 0 | 0.633 | 0.667 |
| HSPA5 | LDLR | 9606.ENSP00000324173 | 9606.ENSP00000454071 | 0 | 0 | 0 | 0 | 0.06 | 0.673 | 0 | 0.674 | 0.891 |
| HTR2A | NR3C1 | 9606.ENSP00000437737 | 9606.ENSP00000231509 | 0 | 0 | 0 | 0 | 0 | 0.056 | 0 | 0.459 | 0.467 |
| HTR2A | SLC6A4 | 9606.ENSP00000437737 | 9606.ENSP00000261707 | 0 | 0 | 0 | 0 | 0 | 0.057 | 0 | 0.912 | 0.914 |
| HTR2A | KCNH2 | 9606.ENSP00000437737 | 9606.ENSP00000262186 | 0 | 0 | 0 | 0 | 0 | 0 | 0 | 0.42 | 0.42 |
| HTR2A | SLC6A3 | 9606.ENSP00000437737 | 9606.ENSP00000270349 | 0 | 0 | 0 | 0 | 0 | 0.057 | 0 | 0.713 | 0.718 |
| HTR2A | CYP3A4 | 9606.ENSP00000437737 | 9606.ENSP00000337915 | 0 | 0 | 0 | 0 | 0 | 0 | 0 | 0.503 | 0.503 |
| HTR2A | MAOA | 9606.ENSP00000437737 | 9606.ENSP00000340684 | 0 | 0 | 0 | 0 | 0 | 0 | 0 | 0.782 | 0.782 |
| HTR2A | CYP1A2 | 9606.ENSP00000437737 | 9606.ENSP00000342007 | 0 | 0 | 0 | 0 | 0 | 0 | 0 | 0.494 | 0.493 |
| HTR2A | HTR3A | 9606.ENSP00000437737 | 9606.ENSP00000347754 | 0 | 0 | 0 | 0 | 0.062 | 0 | 0 | 0.751 | 0.756 |
| HTR2A | MAOB | 9606.ENSP00000437737 | 9606.ENSP00000367309 | 0 | 0 | 0 | 0 | 0.076 | 0 | 0 | 0.597 | 0.611 |
| HTR2A | GSTP1 | 9606.ENSP00000437737 | 9606.ENSP00000381607 | 0 | 0 | 0 | 0 | 0 | 0.063 | 0 | 0.565 | 0.575 |
| HTR2A | CHRNA7 | 9606.ENSP00000437737 | 9606.ENSP00000407546 | 0 | 0 | 0 | 0 | 0 | 0 | 0 | 0.506 | 0.506 |
| HTR3A | NR3C1 | 9606.ENSP00000347754 | 9606.ENSP00000231509 | 0 | 0 | 0 | 0 | 0 | 0.056 | 0 | 0.414 | 0.424 |
| HTR3A | SLC6A4 | 9606.ENSP00000347754 | 9606.ENSP00000261707 | 0 | 0 | 0 | 0 | 0 | 0 | 0 | 0.71 | 0.71 |
| HTR3A | SLC6A3 | 9606.ENSP00000347754 | 9606.ENSP00000270349 | 0 | 0 | 0 | 0 | 0 | 0 | 0 | 0.405 | 0.405 |
| HTR3A | ADRA2A | 9606.ENSP00000347754 | 9606.ENSP00000280155 | 0 | 0 | 0 | 0 | 0.062 | 0 | 0 | 0.422 | 0.434 |
| HTR3A | MAOA | 9606.ENSP00000347754 | 9606.ENSP00000340684 | 0 | 0 | 0 | 0 | 0 | 0 | 0 | 0.519 | 0.519 |
| HTR3A | ADRA1D | 9606.ENSP00000347754 | 9606.ENSP00000368766 | 0 | 0 | 0 | 0 | 0 | 0 | 0 | 0.411 | 0.411 |
| HTR3A | CHRM2 | 9606.ENSP00000347754 | 9606.ENSP00000399745 | 0 | 0 | 0 | 0 | 0.062 | 0 | 0 | 0.417 | 0.43 |
| HTR3A | DRD1 | 9606.ENSP00000347754 | 9606.ENSP00000377353 | 0 | 0 | 0 | 0 | 0.085 | 0 | 0 | 0.405 | 0.432 |
| HTR3A | MAOB | 9606.ENSP00000347754 | 9606.ENSP00000367309 | 0 | 0 | 0 | 0 | 0 | 0 | 0 | 0.439 | 0.439 |
| HTR3A | OPRM1 | 9606.ENSP00000347754 | 9606.ENSP00000394624 | 0 | 0 | 0 | 0 | 0 | 0 | 0 | 0.456 | 0.456 |
| HTR3A | HTR2A | 9606.ENSP00000347754 | 9606.ENSP00000437737 | 0 | 0 | 0 | 0 | 0.062 | 0 | 0 | 0.751 | 0.756 |
| IFNG | MAPK1 | 9606.ENSP00000229135 | 9606.ENSP00000215832 | 0 | 0 | 0 | 0 | 0 | 0 | 0 | 0.415 | 0.415 |
| IFNG | HMOX1 | 9606.ENSP00000229135 | 9606.ENSP00000216117 | 0 | 0 | 0 | 0 | 0 | 0 | 0 | 0.475 | 0.475 |
| IFNG | MMP2 | 9606.ENSP00000229135 | 9606.ENSP00000219070 | 0 | 0 | 0 | 0 | 0 | 0 | 0 | 0.457 | 0.457 |
| IFNG | MPO | 9606.ENSP00000229135 | 9606.ENSP00000225275 | 0 | 0 | 0 | 0 | 0 | 0 | 0 | 0.593 | 0.593 |
| IFNG | CCL2 | 9606.ENSP00000229135 | 9606.ENSP00000225831 | 0 | 0 | 0 | 0 | 0.063 | 0 | 0 | 0.835 | 0.839 |
| IFNG | IL2 | 9606.ENSP00000229135 | 9606.ENSP00000226730 | 0 | 0 | 0 | 0 | 0.087 | 0 | 0 | 0.902 | 0.907 |
| IFNG | NR3C1 | 9606.ENSP00000229135 | 9606.ENSP00000231509 | 0 | 0 | 0 | 0 | 0 | 0 | 0 | 0.401 | 0.401 |
| IFNG | F2 | 9606.ENSP00000229135 | 9606.ENSP00000308541 | 0 | 0 | 0 | 0 | 0 | 0 | 0 | 0.402 | 0.402 |
| IFNG | DPP4 | 9606.ENSP00000229135 | 9606.ENSP00000353731 | 0 | 0 | 0 | 0 | 0 | 0 | 0 | 0.403 | 0.403 |
| IFNG | ESR1 | 9606.ENSP00000229135 | 9606.ENSP00000405330 | 0 | 0 | 0 | 0 | 0 | 0 | 0 | 0.415 | 0.415 |
| IFNG | MMP1 | 9606.ENSP00000229135 | 9606.ENSP00000322788 | 0 | 0 | 0 | 0 | 0.053 | 0 | 0 | 0.425 | 0.432 |
| IFNG | MMP3 | 9606.ENSP00000229135 | 9606.ENSP00000299855 | 0 | 0 | 0 | 0 | 0.053 | 0 | 0 | 0.453 | 0.46 |
| IFNG | F3 | 9606.ENSP00000229135 | 9606.ENSP00000334145 | 0 | 0 | 0 | 0 | 0 | 0 | 0 | 0.474 | 0.474 |
| IFNG | AHR | 9606.ENSP00000229135 | 9606.ENSP00000242057 | 0 | 0 | 0 | 0 | 0 | 0 | 0 | 0.486 | 0.486 |
| IFNG | NOS3 | 9606.ENSP00000229135 | 9606.ENSP00000297494 | 0 | 0 | 0 | 0 | 0 | 0 | 0 | 0.502 | 0.502 |
| IFNG | CAT | 9606.ENSP00000229135 | 9606.ENSP00000241052 | 0 | 0 | 0 | 0 | 0 | 0 | 0 | 0.514 | 0.514 |
| IFNG | MAPK3 | 9606.ENSP00000229135 | 9606.ENSP00000263025 | 0 | 0 | 0 | 0 | 0 | 0 | 0 | 0.528 | 0.528 |
| IFNG | MAPK14 | 9606.ENSP00000229135 | 9606.ENSP00000229795 | 0 | 0 | 0 | 0 | 0 | 0 | 0 | 0.564 | 0.564 |
| IFNG | PPARG | 9606.ENSP00000229135 | 9606.ENSP00000287820 | 0 | 0 | 0 | 0 | 0 | 0 | 0 | 0.579 | 0.579 |
| IFNG | SELE | 9606.ENSP00000229135 | 9606.ENSP00000331736 | 0 | 0 | 0 | 0 | 0 | 0 | 0 | 0.588 | 0.588 |
| IFNG | EGF | 9606.ENSP00000229135 | 9606.ENSP00000265171 | 0 | 0 | 0 | 0 | 0 | 0 | 0 | 0.607 | 0.607 |
| IFNG | PTGS2 | 9606.ENSP00000229135 | 9606.ENSP00000356438 | 0 | 0 | 0 | 0 | 0 | 0 | 0 | 0.643 | 0.643 |
| IFNG | VCAM1 | 9606.ENSP00000229135 | 9606.ENSP00000294728 | 0 | 0 | 0 | 0 | 0 | 0 | 0 | 0.649 | 0.649 |
| IFNG | TP53 | 9606.ENSP00000229135 | 9606.ENSP00000269305 | 0 | 0 | 0 | 0 | 0 | 0 | 0 | 0.657 | 0.657 |
| IFNG | VEGFA | 9606.ENSP00000229135 | 9606.ENSP00000478570 | 0 | 0 | 0 | 0 | 0 | 0 | 0 | 0.689 | 0.689 |
| IFNG | NOS2 | 9606.ENSP00000229135 | 9606.ENSP00000327251 | 0 | 0 | 0 | 0 | 0 | 0 | 0 | 0.764 | 0.764 |
| IFNG | EGFR | 9606.ENSP00000229135 | 9606.ENSP00000275493 | 0 | 0 | 0 | 0 | 0 | 0 | 0.6 | 0.515 | 0.798 |
| IFNG | IL1B | 9606.ENSP00000229135 | 9606.ENSP00000263341 | 0 | 0 | 0 | 0 | 0.08 | 0 | 0 | 0.903 | 0.907 |
| IFNG | PTPN1 | 9606.ENSP00000229135 | 9606.ENSP00000360683 | 0 | 0 | 0 | 0 | 0 | 0 | 0.9 | 0.177 | 0.914 |
| IFNG | TNF | 9606.ENSP00000229135 | 9606.ENSP00000398698 | 0 | 0 | 0 | 0 | 0.152 | 0.213 | 0 | 0.915 | 0.938 |
| IFNG | IL6 | 9606.ENSP00000229135 | 9606.ENSP00000385675 | 0 | 0 | 0 | 0 | 0.066 | 0 | 0 | 0.942 | 0.943 |
| IFNG | JUN | 9606.ENSP00000229135 | 9606.ENSP00000360266 | 0 | 0 | 0 | 0 | 0 | 0 | 0.9 | 0.601 | 0.958 |
| IL1B | MAPK1 | 9606.ENSP00000263341 | 9606.ENSP00000215832 | 0 | 0 | 0 | 0 | 0 | 0 | 0 | 0.426 | 0.426 |
| IL1B | HMOX1 | 9606.ENSP00000263341 | 9606.ENSP00000216117 | 0 | 0 | 0 | 0 | 0.07 | 0 | 0 | 0.76 | 0.767 |
| IL1B | MMP2 | 9606.ENSP00000263341 | 9606.ENSP00000219070 | 0 | 0 | 0 | 0 | 0 | 0.279 | 0 | 0.703 | 0.776 |
| IL1B | PLAT | 9606.ENSP00000263341 | 9606.ENSP00000220809 | 0 | 0 | 0 | 0 | 0 | 0 | 0 | 0.47 | 0.47 |
| IL1B | GSR | 9606.ENSP00000263341 | 9606.ENSP00000221130 | 0 | 0 | 0 | 0 | 0 | 0 | 0 | 0.556 | 0.556 |
| IL1B | MPO | 9606.ENSP00000263341 | 9606.ENSP00000225275 | 0 | 0 | 0 | 0 | 0.098 | 0 | 0 | 0.833 | 0.843 |
| IL1B | CCL2 | 9606.ENSP00000263341 | 9606.ENSP00000225831 | 0 | 0 | 0 | 0 | 0.127 | 0 | 0.9 | 0.927 | 0.993 |
| IL1B | IL2 | 9606.ENSP00000263341 | 9606.ENSP00000226730 | 0 | 0 | 0 | 0 | 0 | 0 | 0 | 0.876 | 0.876 |
| IL1B | IFNG | 9606.ENSP00000263341 | 9606.ENSP00000229135 | 0 | 0 | 0 | 0 | 0.08 | 0 | 0 | 0.903 | 0.907 |
| IL1B | MAPK14 | 9606.ENSP00000263341 | 9606.ENSP00000229795 | 0 | 0 | 0 | 0 | 0 | 0 | 0.9 | 0.734 | 0.972 |
| IL1B | NR3C1 | 9606.ENSP00000263341 | 9606.ENSP00000231509 | 0 | 0 | 0 | 0 | 0 | 0 | 0 | 0.601 | 0.601 |
| IL1B | CTSD | 9606.ENSP00000263341 | 9606.ENSP00000236671 | 0 | 0 | 0 | 0 | 0.062 | 0 | 0 | 0.395 | 0.408 |
| IL1B | CAT | 9606.ENSP00000263341 | 9606.ENSP00000241052 | 0 | 0 | 0 | 0 | 0 | 0 | 0 | 0.734 | 0.734 |
| IL1B | AHR | 9606.ENSP00000263341 | 9606.ENSP00000242057 | 0 | 0 | 0 | 0 | 0.071 | 0 | 0 | 0.6 | 0.612 |
| IL1B | SLC6A4 | 9606.ENSP00000263341 | 9606.ENSP00000261707 | 0 | 0 | 0 | 0 | 0 | 0 | 0 | 0.43 | 0.43 |
| IL1B | MAPK3 | 9606.ENSP00000263341 | 9606.ENSP00000263025 | 0 | 0 | 0 | 0 | 0 | 0 | 0 | 0.712 | 0.712 |
| IL1B | XDH | 9606.ENSP00000263341 | 9606.ENSP00000368727 | 0 | 0 | 0 | 0 | 0.063 | 0 | 0 | 0.403 | 0.417 |
| IL1B | ESR2 | 9606.ENSP00000263341 | 9606.ENSP00000343925 | 0 | 0 | 0 | 0 | 0 | 0 | 0 | 0.46 | 0.459 |
| IL1B | PTGER3 | 9606.ENSP00000263341 | 9606.ENSP00000349003 | 0 | 0 | 0 | 0 | 0 | 0 | 0 | 0.468 | 0.468 |
| IL1B | BAX | 9606.ENSP00000263341 | 9606.ENSP00000293288 | 0 | 0 | 0 | 0 | 0.049 | 0 | 0 | 0.465 | 0.469 |
| IL1B | LDLR | 9606.ENSP00000263341 | 9606.ENSP00000454071 | 0 | 0 | 0 | 0 | 0 | 0 | 0 | 0.471 | 0.471 |
| IL1B | DPP4 | 9606.ENSP00000263341 | 9606.ENSP00000353731 | 0 | 0 | 0 | 0 | 0.062 | 0 | 0 | 0.465 | 0.476 |
| IL1B | GSK3B | 9606.ENSP00000263341 | 9606.ENSP00000324806 | 0 | 0 | 0 | 0 | 0 | 0.056 | 0 | 0.495 | 0.503 |
| IL1B | HSPA5 | 9606.ENSP00000263341 | 9606.ENSP00000324173 | 0 | 0 | 0 | 0 | 0 | 0 | 0 | 0.506 | 0.506 |
| IL1B | ACHE | 9606.ENSP00000263341 | 9606.ENSP00000303211 | 0 | 0 | 0 | 0 | 0 | 0 | 0 | 0.508 | 0.508 |
| IL1B | PTPN1 | 9606.ENSP00000263341 | 9606.ENSP00000360683 | 0 | 0 | 0 | 0 | 0 | 0.27 | 0 | 0.362 | 0.514 |
| IL1B | GJA1 | 9606.ENSP00000263341 | 9606.ENSP00000282561 | 0 | 0 | 0 | 0 | 0 | 0 | 0 | 0.519 | 0.519 |
| IL1B | ADRB2 | 9606.ENSP00000263341 | 9606.ENSP00000305372 | 0 | 0 | 0 | 0 | 0.088 | 0.213 | 0 | 0.413 | 0.541 |
| IL1B | F2 | 9606.ENSP00000263341 | 9606.ENSP00000308541 | 0 | 0 | 0 | 0 | 0 | 0 | 0 | 0.556 | 0.556 |
| IL1B | EGFR | 9606.ENSP00000263341 | 9606.ENSP00000275493 | 0 | 0 | 0 | 0 | 0 | 0 | 0 | 0.561 | 0.561 |
| IL1B | SOD1 | 9606.ENSP00000263341 | 9606.ENSP00000270142 | 0 | 0 | 0 | 0 | 0 | 0 | 0 | 0.562 | 0.562 |
| IL1B | NQO1 | 9606.ENSP00000263341 | 9606.ENSP00000319788 | 0 | 0 | 0 | 0 | 0 | 0 | 0 | 0.564 | 0.564 |
| IL1B | PLAU | 9606.ENSP00000263341 | 9606.ENSP00000361850 | 0 | 0 | 0 | 0 | 0.076 | 0 | 0 | 0.562 | 0.578 |
| IL1B | THBD | 9606.ENSP00000263341 | 9606.ENSP00000366307 | 0 | 0 | 0 | 0 | 0.153 | 0 | 0 | 0.556 | 0.607 |
| IL1B | KDR | 9606.ENSP00000263341 | 9606.ENSP00000263923 | 0 | 0 | 0 | 0 | 0.062 | 0 | 0 | 0.619 | 0.627 |
| IL1B | ALOX5 | 9606.ENSP00000263341 | 9606.ENSP00000363512 | 0 | 0 | 0 | 0 | 0.098 | 0 | 0 | 0.606 | 0.629 |
| IL1B | PTGS1 | 9606.ENSP00000263341 | 9606.ENSP00000354612 | 0 | 0 | 0 | 0 | 0.157 | 0 | 0 | 0.605 | 0.654 |
| IL1B | MAPK8 | 9606.ENSP00000263341 | 9606.ENSP00000378974 | 0 | 0 | 0 | 0 | 0 | 0 | 0 | 0.658 | 0.658 |
| IL1B | F3 | 9606.ENSP00000263341 | 9606.ENSP00000334145 | 0 | 0 | 0 | 0 | 0.062 | 0 | 0 | 0.653 | 0.66 |
| IL1B | TP53 | 9606.ENSP00000263341 | 9606.ENSP00000269305 | 0 | 0 | 0 | 0 | 0 | 0 | 0 | 0.689 | 0.689 |
| IL1B | NOS3 | 9606.ENSP00000263341 | 9606.ENSP00000297494 | 0 | 0 | 0 | 0 | 0.062 | 0 | 0 | 0.689 | 0.696 |
| IL1B | EGF | 9606.ENSP00000263341 | 9606.ENSP00000265171 | 0 | 0 | 0 | 0 | 0 | 0 | 0 | 0.71 | 0.71 |
| IL1B | ESR1 | 9606.ENSP00000263341 | 9606.ENSP00000405330 | 0 | 0 | 0 | 0 | 0 | 0 | 0 | 0.729 | 0.729 |
| IL1B | PPARG | 9606.ENSP00000263341 | 9606.ENSP00000287820 | 0 | 0 | 0 | 0 | 0.062 | 0 | 0 | 0.739 | 0.745 |
| IL1B | SELE | 9606.ENSP00000263341 | 9606.ENSP00000331736 | 0 | 0 | 0 | 0 | 0.062 | 0 | 0 | 0.753 | 0.758 |
| IL1B | MMP1 | 9606.ENSP00000263341 | 9606.ENSP00000322788 | 0 | 0 | 0 | 0 | 0.128 | 0.058 | 0 | 0.742 | 0.77 |
| IL1B | NOS2 | 9606.ENSP00000263341 | 9606.ENSP00000327251 | 0 | 0 | 0 | 0 | 0.068 | 0 | 0 | 0.807 | 0.813 |
| IL1B | MMP3 | 9606.ENSP00000263341 | 9606.ENSP00000299855 | 0 | 0 | 0 | 0 | 0.107 | 0.058 | 0 | 0.809 | 0.825 |
| IL1B | VCAM1 | 9606.ENSP00000263341 | 9606.ENSP00000294728 | 0 | 0 | 0 | 0 | 0.062 | 0 | 0 | 0.826 | 0.83 |
| IL1B | VEGFA | 9606.ENSP00000263341 | 9606.ENSP00000478570 | 0 | 0 | 0 | 0 | 0 | 0 | 0 | 0.857 | 0.857 |
| IL1B | PTGS2 | 9606.ENSP00000263341 | 9606.ENSP00000356438 | 0 | 0 | 0 | 0 | 0.561 | 0 | 0 | 0.877 | 0.944 |
| IL1B | JUN | 9606.ENSP00000263341 | 9606.ENSP00000360266 | 0 | 0 | 0 | 0 | 0.052 | 0 | 0.8 | 0.792 | 0.957 |
| IL1B | TNF | 9606.ENSP00000263341 | 9606.ENSP00000398698 | 0 | 0 | 0 | 0 | 0.462 | 0 | 0.5 | 0.942 | 0.983 |
| IL1B | IL6 | 9606.ENSP00000263341 | 9606.ENSP00000385675 | 0 | 0 | 0 | 0 | 0.427 | 0 | 0.9 | 0.953 | 0.997 |
| IL2 | MAPK1 | 9606.ENSP00000226730 | 9606.ENSP00000215832 | 0 | 0 | 0 | 0 | 0 | 0 | 0.9 | 0.346 | 0.931 |
| IL2 | HMOX1 | 9606.ENSP00000226730 | 9606.ENSP00000216117 | 0 | 0 | 0 | 0 | 0 | 0 | 0 | 0.414 | 0.414 |
| IL2 | MMP2 | 9606.ENSP00000226730 | 9606.ENSP00000219070 | 0 | 0 | 0 | 0 | 0 | 0 | 0 | 0.424 | 0.424 |
| IL2 | MPO | 9606.ENSP00000226730 | 9606.ENSP00000225275 | 0 | 0 | 0 | 0 | 0 | 0 | 0 | 0.516 | 0.516 |
| IL2 | CCL2 | 9606.ENSP00000226730 | 9606.ENSP00000225831 | 0 | 0 | 0 | 0 | 0 | 0 | 0 | 0.81 | 0.81 |
| IL2 | KDR | 9606.ENSP00000226730 | 9606.ENSP00000263923 | 0 | 0 | 0 | 0 | 0 | 0 | 0 | 0.414 | 0.414 |
| IL2 | F3 | 9606.ENSP00000226730 | 9606.ENSP00000334145 | 0 | 0 | 0 | 0 | 0 | 0 | 0 | 0.414 | 0.414 |
| IL2 | MMP3 | 9606.ENSP00000226730 | 9606.ENSP00000299855 | 0 | 0 | 0 | 0 | 0 | 0 | 0 | 0.422 | 0.422 |
| IL2 | NR3C1 | 9606.ENSP00000226730 | 9606.ENSP00000231509 | 0 | 0 | 0 | 0 | 0 | 0 | 0 | 0.422 | 0.422 |
| IL2 | OPRM1 | 9606.ENSP00000226730 | 9606.ENSP00000394624 | 0 | 0 | 0 | 0 | 0 | 0 | 0 | 0.433 | 0.433 |
| IL2 | PPARG | 9606.ENSP00000226730 | 9606.ENSP00000287820 | 0 | 0 | 0 | 0 | 0 | 0 | 0 | 0.46 | 0.459 |
| IL2 | DPP4 | 9606.ENSP00000226730 | 9606.ENSP00000353731 | 0 | 0 | 0 | 0 | 0 | 0 | 0 | 0.459 | 0.459 |
| IL2 | CAT | 9606.ENSP00000226730 | 9606.ENSP00000241052 | 0 | 0 | 0 | 0 | 0 | 0 | 0 | 0.467 | 0.467 |
| IL2 | NOS2 | 9606.ENSP00000226730 | 9606.ENSP00000327251 | 0 | 0 | 0 | 0 | 0 | 0 | 0 | 0.487 | 0.487 |
| IL2 | AHR | 9606.ENSP00000226730 | 9606.ENSP00000242057 | 0 | 0 | 0 | 0 | 0 | 0 | 0 | 0.503 | 0.503 |
| IL2 | SELE | 9606.ENSP00000226730 | 9606.ENSP00000331736 | 0 | 0 | 0 | 0 | 0 | 0 | 0 | 0.533 | 0.533 |
| IL2 | PTGS2 | 9606.ENSP00000226730 | 9606.ENSP00000356438 | 0 | 0 | 0 | 0 | 0 | 0 | 0 | 0.577 | 0.577 |
| IL2 | VCAM1 | 9606.ENSP00000226730 | 9606.ENSP00000294728 | 0 | 0 | 0 | 0 | 0 | 0 | 0 | 0.603 | 0.603 |
| IL2 | EGF | 9606.ENSP00000226730 | 9606.ENSP00000265171 | 0 | 0 | 0 | 0 | 0 | 0 | 0 | 0.615 | 0.615 |
| IL2 | TP53 | 9606.ENSP00000226730 | 9606.ENSP00000269305 | 0 | 0 | 0 | 0 | 0 | 0 | 0 | 0.667 | 0.667 |
| IL2 | VEGFA | 9606.ENSP00000226730 | 9606.ENSP00000478570 | 0 | 0 | 0 | 0 | 0 | 0 | 0 | 0.753 | 0.753 |
| IL2 | EGFR | 9606.ENSP00000226730 | 9606.ENSP00000275493 | 0 | 0 | 0 | 0 | 0 | 0 | 0.6 | 0.556 | 0.815 |
| IL2 | IL1B | 9606.ENSP00000226730 | 9606.ENSP00000263341 | 0 | 0 | 0 | 0 | 0 | 0 | 0 | 0.876 | 0.876 |
| IL2 | IFNG | 9606.ENSP00000226730 | 9606.ENSP00000229135 | 0 | 0 | 0 | 0 | 0.087 | 0 | 0 | 0.902 | 0.907 |
| IL2 | IL6 | 9606.ENSP00000226730 | 9606.ENSP00000385675 | 0 | 0 | 0 | 0 | 0 | 0 | 0 | 0.914 | 0.914 |
| IL2 | TNF | 9606.ENSP00000226730 | 9606.ENSP00000398698 | 0 | 0 | 0 | 0 | 0 | 0 | 0 | 0.933 | 0.933 |
| IL2 | MAPK8 | 9606.ENSP00000226730 | 9606.ENSP00000378974 | 0 | 0 | 0 | 0 | 0 | 0 | 0.9 | 0.401 | 0.937 |
| IL2 | MAPK14 | 9606.ENSP00000226730 | 9606.ENSP00000229795 | 0 | 0 | 0 | 0 | 0 | 0 | 0.9 | 0.412 | 0.938 |
| IL2 | MAPK3 | 9606.ENSP00000226730 | 9606.ENSP00000263025 | 0 | 0 | 0 | 0 | 0 | 0 | 0.9 | 0.523 | 0.95 |
| IL2 | JUN | 9606.ENSP00000226730 | 9606.ENSP00000360266 | 0 | 0 | 0 | 0 | 0 | 0.27 | 0.9 | 0.619 | 0.969 |
| IL6 | MAPK1 | 9606.ENSP00000385675 | 9606.ENSP00000215832 | 0 | 0 | 0 | 0 | 0 | 0 | 0.9 | 0.589 | 0.957 |
| IL6 | HMOX1 | 9606.ENSP00000385675 | 9606.ENSP00000216117 | 0 | 0 | 0 | 0 | 0.073 | 0 | 0 | 0.78 | 0.787 |
| IL6 | MMP2 | 9606.ENSP00000385675 | 9606.ENSP00000219070 | 0 | 0 | 0 | 0 | 0.076 | 0 | 0.9 | 0.715 | 0.971 |
| IL6 | PLAT | 9606.ENSP00000385675 | 9606.ENSP00000220809 | 0 | 0 | 0 | 0 | 0.076 | 0 | 0 | 0.611 | 0.625 |
| IL6 | GSR | 9606.ENSP00000385675 | 9606.ENSP00000221130 | 0 | 0 | 0 | 0 | 0 | 0 | 0 | 0.563 | 0.563 |
| IL6 | PON1 | 9606.ENSP00000385675 | 9606.ENSP00000222381 | 0 | 0 | 0 | 0 | 0 | 0 | 0 | 0.505 | 0.505 |
| IL6 | MPO | 9606.ENSP00000385675 | 9606.ENSP00000225275 | 0 | 0 | 0 | 0 | 0.062 | 0 | 0 | 0.839 | 0.843 |
| IL6 | CCL2 | 9606.ENSP00000385675 | 9606.ENSP00000225831 | 0 | 0 | 0 | 0 | 0.267 | 0 | 0.9 | 0.93 | 0.994 |
| IL6 | IL2 | 9606.ENSP00000385675 | 9606.ENSP00000226730 | 0 | 0 | 0 | 0 | 0 | 0 | 0 | 0.914 | 0.914 |
| IL6 | IFNG | 9606.ENSP00000385675 | 9606.ENSP00000229135 | 0 | 0 | 0 | 0 | 0.066 | 0 | 0 | 0.942 | 0.943 |
| IL6 | MAPK14 | 9606.ENSP00000385675 | 9606.ENSP00000229795 | 0 | 0 | 0 | 0 | 0 | 0 | 0 | 0.758 | 0.758 |
| IL6 | NR3C1 | 9606.ENSP00000385675 | 9606.ENSP00000231509 | 0 | 0 | 0 | 0 | 0 | 0 | 0 | 0.624 | 0.624 |
| IL6 | CAT | 9606.ENSP00000385675 | 9606.ENSP00000241052 | 0 | 0 | 0 | 0 | 0 | 0 | 0 | 0.756 | 0.756 |
| IL6 | AHR | 9606.ENSP00000385675 | 9606.ENSP00000242057 | 0 | 0 | 0 | 0 | 0.069 | 0 | 0 | 0.655 | 0.665 |
| IL6 | CHRM3 | 9606.ENSP00000385675 | 9606.ENSP00000255380 | 0 | 0 | 0 | 0 | 0 | 0 | 0 | 0.411 | 0.411 |
| IL6 | CDK4 | 9606.ENSP00000385675 | 9606.ENSP00000257904 | 0 | 0 | 0 | 0 | 0 | 0 | 0 | 0.458 | 0.457 |
| IL6 | SLC6A4 | 9606.ENSP00000385675 | 9606.ENSP00000261707 | 0 | 0 | 0 | 0 | 0 | 0 | 0 | 0.598 | 0.598 |
| IL6 | MAPK3 | 9606.ENSP00000385675 | 9606.ENSP00000263025 | 0 | 0 | 0 | 0 | 0 | 0 | 0.9 | 0.741 | 0.973 |
| IL6 | IL1B | 9606.ENSP00000385675 | 9606.ENSP00000263341 | 0 | 0 | 0 | 0 | 0.427 | 0 | 0.9 | 0.953 | 0.997 |
| IL6 | KDR | 9606.ENSP00000385675 | 9606.ENSP00000263923 | 0 | 0 | 0 | 0 | 0 | 0 | 0 | 0.61 | 0.61 |
| IL6 | EGF | 9606.ENSP00000385675 | 9606.ENSP00000265171 | 0 | 0 | 0 | 0 | 0 | 0 | 0 | 0.763 | 0.763 |
| IL6 | CDK2 | 9606.ENSP00000385675 | 9606.ENSP00000266970 | 0 | 0 | 0 | 0 | 0 | 0 | 0 | 0.465 | 0.465 |
| IL6 | TP53 | 9606.ENSP00000385675 | 9606.ENSP00000269305 | 0 | 0 | 0 | 0 | 0.062 | 0 | 0 | 0.738 | 0.744 |
| IL6 | SOD1 | 9606.ENSP00000385675 | 9606.ENSP00000270142 | 0 | 0 | 0 | 0 | 0 | 0 | 0 | 0.535 | 0.535 |
| IL6 | EGFR | 9606.ENSP00000385675 | 9606.ENSP00000275493 | 0 | 0 | 0 | 0 | 0 | 0 | 0.6 | 0.683 | 0.867 |
| IL6 | GJA1 | 9606.ENSP00000385675 | 9606.ENSP00000282561 | 0 | 0 | 0 | 0 | 0 | 0 | 0 | 0.609 | 0.609 |
| IL6 | PPARG | 9606.ENSP00000385675 | 9606.ENSP00000287820 | 0 | 0 | 0 | 0 | 0 | 0 | 0 | 0.792 | 0.792 |
| IL6 | HMGCR | 9606.ENSP00000385675 | 9606.ENSP00000287936 | 0 | 0 | 0 | 0 | 0 | 0 | 0 | 0.455 | 0.455 |
| IL6 | BAX | 9606.ENSP00000385675 | 9606.ENSP00000293288 | 0 | 0 | 0 | 0 | 0 | 0 | 0 | 0.459 | 0.459 |
| IL6 | VCAM1 | 9606.ENSP00000385675 | 9606.ENSP00000294728 | 0 | 0 | 0 | 0 | 0.083 | 0 | 0 | 0.86 | 0.866 |
| IL6 | NOS3 | 9606.ENSP00000385675 | 9606.ENSP00000297494 | 0 | 0 | 0 | 0 | 0.062 | 0 | 0 | 0.717 | 0.724 |
| IL6 | MMP3 | 9606.ENSP00000385675 | 9606.ENSP00000299855 | 0 | 0 | 0 | 0 | 0.112 | 0 | 0.9 | 0.768 | 0.977 |
| IL6 | ACHE | 9606.ENSP00000385675 | 9606.ENSP00000303211 | 0 | 0 | 0 | 0 | 0 | 0 | 0 | 0.536 | 0.536 |
| IL6 | FASN | 9606.ENSP00000385675 | 9606.ENSP00000304592 | 0 | 0 | 0 | 0 | 0 | 0 | 0 | 0.459 | 0.459 |
| IL6 | ADRB2 | 9606.ENSP00000385675 | 9606.ENSP00000305372 | 0 | 0 | 0 | 0 | 0.063 | 0 | 0 | 0.47 | 0.482 |
| IL6 | F2 | 9606.ENSP00000385675 | 9606.ENSP00000308541 | 0 | 0 | 0 | 0 | 0 | 0 | 0 | 0.633 | 0.633 |
| IL6 | PPARD | 9606.ENSP00000385675 | 9606.ENSP00000310928 | 0 | 0 | 0 | 0 | 0 | 0 | 0 | 0.415 | 0.415 |
| IL6 | NQO1 | 9606.ENSP00000385675 | 9606.ENSP00000319788 | 0 | 0 | 0 | 0 | 0 | 0 | 0 | 0.57 | 0.57 |
| IL6 | MMP1 | 9606.ENSP00000385675 | 9606.ENSP00000322788 | 0 | 0 | 0 | 0 | 0.154 | 0 | 0.9 | 0.71 | 0.973 |
| IL6 | HSPA5 | 9606.ENSP00000385675 | 9606.ENSP00000324173 | 0 | 0 | 0 | 0 | 0 | 0 | 0 | 0.527 | 0.527 |
| IL6 | GSK3B | 9606.ENSP00000385675 | 9606.ENSP00000324806 | 0 | 0 | 0 | 0 | 0 | 0 | 0 | 0.581 | 0.581 |
| IL6 | PGR | 9606.ENSP00000385675 | 9606.ENSP00000325120 | 0 | 0 | 0 | 0 | 0 | 0 | 0 | 0.459 | 0.459 |
| IL6 | NOS2 | 9606.ENSP00000385675 | 9606.ENSP00000327251 | 0 | 0 | 0 | 0 | 0.063 | 0 | 0 | 0.784 | 0.789 |
| IL6 | SELE | 9606.ENSP00000385675 | 9606.ENSP00000331736 | 0 | 0 | 0 | 0 | 0.121 | 0 | 0 | 0.803 | 0.819 |
| IL6 | F3 | 9606.ENSP00000385675 | 9606.ENSP00000334145 | 0 | 0 | 0 | 0 | 0.088 | 0 | 0 | 0.705 | 0.719 |
| IL6 | CYP3A4 | 9606.ENSP00000385675 | 9606.ENSP00000337915 | 0 | 0 | 0 | 0 | 0 | 0 | 0 | 0.457 | 0.457 |
| IL6 | ESR2 | 9606.ENSP00000385675 | 9606.ENSP00000343925 | 0 | 0 | 0 | 0 | 0 | 0 | 0 | 0.535 | 0.535 |
| IL6 | PTGER3 | 9606.ENSP00000385675 | 9606.ENSP00000349003 | 0 | 0 | 0 | 0 | 0 | 0 | 0 | 0.462 | 0.462 |
| IL6 | NR3C2 | 9606.ENSP00000385675 | 9606.ENSP00000350815 | 0 | 0 | 0 | 0 | 0 | 0 | 0 | 0.417 | 0.417 |
| IL6 | DPP4 | 9606.ENSP00000385675 | 9606.ENSP00000353731 | 0 | 0 | 0 | 0 | 0 | 0 | 0 | 0.566 | 0.566 |
| IL6 | PTGS1 | 9606.ENSP00000385675 | 9606.ENSP00000354612 | 0 | 0 | 0 | 0 | 0.096 | 0 | 0 | 0.54 | 0.566 |
| IL6 | PTGS2 | 9606.ENSP00000385675 | 9606.ENSP00000356438 | 0 | 0 | 0 | 0 | 0.312 | 0 | 0 | 0.878 | 0.913 |
| IL6 | JUN | 9606.ENSP00000385675 | 9606.ENSP00000360266 | 0 | 0 | 0 | 0 | 0.052 | 0 | 0.9 | 0.822 | 0.981 |
| IL6 | PTPN1 | 9606.ENSP00000385675 | 9606.ENSP00000360683 | 0 | 0 | 0 | 0 | 0.062 | 0 | 0 | 0.431 | 0.443 |
| IL6 | PLAU | 9606.ENSP00000385675 | 9606.ENSP00000361850 | 0 | 0 | 0 | 0 | 0.134 | 0 | 0 | 0.506 | 0.553 |
| IL6 | ALOX5 | 9606.ENSP00000385675 | 9606.ENSP00000363512 | 0 | 0 | 0 | 0 | 0 | 0 | 0 | 0.601 | 0.601 |
| IL6 | AR | 9606.ENSP00000385675 | 9606.ENSP00000363822 | 0 | 0 | 0 | 0 | 0 | 0 | 0 | 0.56 | 0.56 |
| IL6 | F10 | 9606.ENSP00000385675 | 9606.ENSP00000364709 | 0 | 0 | 0 | 0 | 0.062 | 0 | 0 | 0.395 | 0.408 |
| IL6 | F7 | 9606.ENSP00000385675 | 9606.ENSP00000364731 | 0 | 0 | 0 | 0 | 0 | 0 | 0 | 0.469 | 0.469 |
| IL6 | THBD | 9606.ENSP00000385675 | 9606.ENSP00000366307 | 0 | 0 | 0 | 0 | 0.098 | 0 | 0 | 0.617 | 0.639 |
| IL6 | XDH | 9606.ENSP00000385675 | 9606.ENSP00000368727 | 0 | 0 | 0 | 0 | 0 | 0 | 0 | 0.404 | 0.404 |
| IL6 | MAPK8 | 9606.ENSP00000385675 | 9606.ENSP00000378974 | 0 | 0 | 0 | 0 | 0 | 0 | 0 | 0.668 | 0.668 |
| IL6 | LDLR | 9606.ENSP00000385675 | 9606.ENSP00000454071 | 0 | 0 | 0 | 0 | 0 | 0 | 0 | 0.521 | 0.521 |
| IL6 | ESR1 | 9606.ENSP00000385675 | 9606.ENSP00000405330 | 0 | 0 | 0 | 0 | 0 | 0 | 0 | 0.677 | 0.677 |
| IL6 | VEGFA | 9606.ENSP00000385675 | 9606.ENSP00000478570 | 0 | 0 | 0 | 0 | 0.063 | 0 | 0.9 | 0.879 | 0.987 |
| IL6 | TNF | 9606.ENSP00000385675 | 9606.ENSP00000398698 | 0 | 0 | 0 | 0 | 0.125 | 0 | 0.9 | 0.941 | 0.994 |
| INSR | MAPK1 | 9606.ENSP00000303830 | 9606.ENSP00000215832 | 0 | 0 | 0 | 0.571 | 0 | 0.351 | 0 | 0.427 | 0.463 |
| INSR | MAPK3 | 9606.ENSP00000303830 | 9606.ENSP00000263025 | 0 | 0 | 0 | 0.573 | 0 | 0.351 | 0 | 0.445 | 0.468 |
| INSR | EGF | 9606.ENSP00000303830 | 9606.ENSP00000265171 | 0 | 0 | 0 | 0 | 0 | 0.056 | 0.8 | 0.323 | 0.861 |
| INSR | EGFR | 9606.ENSP00000303830 | 9606.ENSP00000275493 | 0 | 0 | 0 | 0.574 | 0.062 | 0.107 | 0.5 | 0.567 | 0.651 |
| INSR | PPARG | 9606.ENSP00000303830 | 9606.ENSP00000287820 | 0 | 0 | 0 | 0 | 0 | 0.077 | 0 | 0.507 | 0.525 |
| INSR | GSK3B | 9606.ENSP00000303830 | 9606.ENSP00000324806 | 0 | 0 | 0 | 0 | 0 | 0.056 | 0 | 0.399 | 0.409 |
| INSR | ESR1 | 9606.ENSP00000303830 | 9606.ENSP00000405330 | 0 | 0 | 0 | 0 | 0 | 0.077 | 0 | 0.391 | 0.413 |
| INSR | FASN | 9606.ENSP00000303830 | 9606.ENSP00000304592 | 0 | 0 | 0 | 0 | 0 | 0 | 0 | 0.428 | 0.428 |
| INSR | PIK3CG | 9606.ENSP00000303830 | 9606.ENSP00000352121 | 0 | 0 | 0 | 0 | 0.063 | 0.143 | 0 | 0.418 | 0.491 |
| INSR | VEGFA | 9606.ENSP00000303830 | 9606.ENSP00000478570 | 0 | 0 | 0 | 0 | 0.062 | 0 | 0.6 | 0.245 | 0.691 |
| INSR | ADRB2 | 9606.ENSP00000303830 | 9606.ENSP00000305372 | 0 | 0 | 0 | 0 | 0 | 0.225 | 0 | 0.703 | 0.76 |
| INSR | PTPN1 | 9606.ENSP00000303830 | 9606.ENSP00000360683 | 0 | 0 | 0 | 0 | 0.062 | 0.985 | 0.9 | 0.547 | 0.999 |
| JUN | MAPK1 | 9606.ENSP00000360266 | 9606.ENSP00000215832 | 0 | 0 | 0 | 0 | 0 | 0.867 | 0.9 | 0.707 | 0.995 |
| JUN | HMOX1 | 9606.ENSP00000360266 | 9606.ENSP00000216117 | 0 | 0 | 0 | 0 | 0 | 0 | 0.9 | 0.692 | 0.967 |
| JUN | MMP2 | 9606.ENSP00000360266 | 9606.ENSP00000219070 | 0 | 0 | 0 | 0 | 0 | 0 | 0 | 0.655 | 0.655 |
| JUN | GSR | 9606.ENSP00000360266 | 9606.ENSP00000221130 | 0 | 0 | 0 | 0 | 0 | 0.129 | 0 | 0.458 | 0.507 |
| JUN | MPO | 9606.ENSP00000360266 | 9606.ENSP00000225275 | 0 | 0 | 0 | 0 | 0 | 0 | 0 | 0.458 | 0.458 |
| JUN | CCL2 | 9606.ENSP00000360266 | 9606.ENSP00000225831 | 0 | 0 | 0 | 0 | 0.062 | 0 | 0.9 | 0.686 | 0.968 |
| JUN | IL2 | 9606.ENSP00000360266 | 9606.ENSP00000226730 | 0 | 0 | 0 | 0 | 0 | 0.27 | 0.9 | 0.619 | 0.969 |
| JUN | IFNG | 9606.ENSP00000360266 | 9606.ENSP00000229135 | 0 | 0 | 0 | 0 | 0 | 0 | 0.9 | 0.601 | 0.958 |
| JUN | MAPK14 | 9606.ENSP00000360266 | 9606.ENSP00000229795 | 0 | 0 | 0 | 0 | 0 | 0.4 | 0.9 | 0.838 | 0.989 |
| JUN | NR3C1 | 9606.ENSP00000360266 | 9606.ENSP00000231509 | 0 | 0 | 0 | 0 | 0 | 0.493 | 0.9 | 0.973 | 0.998 |
| JUN | ODC1 | 9606.ENSP00000360266 | 9606.ENSP00000234111 | 0 | 0 | 0 | 0 | 0 | 0 | 0 | 0.506 | 0.506 |
| JUN | CAT | 9606.ENSP00000360266 | 9606.ENSP00000241052 | 0 | 0 | 0 | 0 | 0 | 0 | 0 | 0.66 | 0.66 |
| JUN | AHR | 9606.ENSP00000360266 | 9606.ENSP00000242057 | 0 | 0 | 0 | 0 | 0 | 0 | 0 | 0.588 | 0.588 |
| JUN | CDK4 | 9606.ENSP00000360266 | 9606.ENSP00000257904 | 0 | 0 | 0 | 0 | 0.062 | 0.239 | 0 | 0.624 | 0.708 |
| JUN | MAPK3 | 9606.ENSP00000360266 | 9606.ENSP00000263025 | 0 | 0 | 0 | 0 | 0 | 0.5 | 0.9 | 0.852 | 0.991 |
| JUN | IL1B | 9606.ENSP00000360266 | 9606.ENSP00000263341 | 0 | 0 | 0 | 0 | 0.052 | 0 | 0.8 | 0.792 | 0.957 |
| JUN | KDR | 9606.ENSP00000360266 | 9606.ENSP00000263923 | 0 | 0 | 0 | 0 | 0 | 0.076 | 0 | 0.513 | 0.531 |
| JUN | EGF | 9606.ENSP00000360266 | 9606.ENSP00000265171 | 0 | 0 | 0 | 0 | 0 | 0 | 0 | 0.701 | 0.701 |
| JUN | CDK2 | 9606.ENSP00000360266 | 9606.ENSP00000266970 | 0 | 0 | 0 | 0 | 0.049 | 0.239 | 0 | 0.7 | 0.765 |
| JUN | RB1 | 9606.ENSP00000360266 | 9606.ENSP00000267163 | 0 | 0 | 0 | 0 | 0 | 0.486 | 0.9 | 0.421 | 0.967 |
| JUN | TP53 | 9606.ENSP00000360266 | 9606.ENSP00000269305 | 0 | 0 | 0 | 0 | 0 | 0.149 | 0.6 | 0.838 | 0.94 |
| JUN | SOD1 | 9606.ENSP00000360266 | 9606.ENSP00000270142 | 0 | 0 | 0 | 0 | 0 | 0 | 0 | 0.439 | 0.439 |
| JUN | CCNA2 | 9606.ENSP00000360266 | 9606.ENSP00000274026 | 0 | 0 | 0 | 0 | 0 | 0 | 0 | 0.559 | 0.559 |
| JUN | EGFR | 9606.ENSP00000360266 | 9606.ENSP00000275493 | 0 | 0 | 0 | 0 | 0.06 | 0.139 | 0 | 0.732 | 0.764 |
| JUN | GJA1 | 9606.ENSP00000360266 | 9606.ENSP00000282561 | 0 | 0 | 0 | 0 | 0 | 0 | 0 | 0.45 | 0.45 |
| JUN | CA2 | 9606.ENSP00000360266 | 9606.ENSP00000285379 | 0 | 0 | 0 | 0 | 0 | 0.129 | 0 | 0.413 | 0.467 |
| JUN | PPARG | 9606.ENSP00000360266 | 9606.ENSP00000287820 | 0 | 0 | 0 | 0 | 0 | 0.066 | 0.9 | 0.842 | 0.984 |
| JUN | BAX | 9606.ENSP00000360266 | 9606.ENSP00000293288 | 0 | 0 | 0 | 0 | 0 | 0 | 0 | 0.558 | 0.558 |
| JUN | VCAM1 | 9606.ENSP00000360266 | 9606.ENSP00000294728 | 0 | 0 | 0 | 0 | 0 | 0 | 0 | 0.565 | 0.565 |
| JUN | NOS3 | 9606.ENSP00000360266 | 9606.ENSP00000297494 | 0 | 0 | 0 | 0 | 0 | 0 | 0.9 | 0.603 | 0.958 |
| JUN | MMP3 | 9606.ENSP00000360266 | 9606.ENSP00000299855 | 0 | 0 | 0 | 0 | 0 | 0 | 0 | 0.57 | 0.57 |
| JUN | CHRM1 | 9606.ENSP00000360266 | 9606.ENSP00000306490 | 0 | 0 | 0 | 0 | 0 | 0 | 0.9 | 0.062 | 0.902 |
| JUN | PRKACA | 9606.ENSP00000360266 | 9606.ENSP00000309591 | 0 | 0 | 0 | 0 | 0 | 0 | 0.8 | 0.16 | 0.824 |
| JUN | PPARD | 9606.ENSP00000360266 | 9606.ENSP00000310928 | 0 | 0 | 0 | 0 | 0 | 0.066 | 0 | 0.394 | 0.41 |
| JUN | NQO1 | 9606.ENSP00000360266 | 9606.ENSP00000319788 | 0 | 0 | 0 | 0 | 0 | 0 | 0 | 0.517 | 0.517 |
| JUN | MMP1 | 9606.ENSP00000360266 | 9606.ENSP00000322788 | 0 | 0 | 0 | 0 | 0 | 0.628 | 0 | 0.621 | 0.853 |
| JUN | HSPA5 | 9606.ENSP00000360266 | 9606.ENSP00000324173 | 0 | 0 | 0 | 0 | 0.062 | 0.111 | 0 | 0.633 | 0.667 |
| JUN | GSK3B | 9606.ENSP00000360266 | 9606.ENSP00000324806 | 0 | 0 | 0 | 0 | 0 | 0.691 | 0.9 | 0.709 | 0.99 |
| JUN | PGR | 9606.ENSP00000360266 | 9606.ENSP00000325120 | 0 | 0 | 0 | 0 | 0 | 0.066 | 0 | 0.627 | 0.637 |
| JUN | NOS2 | 9606.ENSP00000360266 | 9606.ENSP00000327251 | 0 | 0 | 0 | 0 | 0 | 0 | 0.9 | 0.581 | 0.956 |
| JUN | SELE | 9606.ENSP00000360266 | 9606.ENSP00000331736 | 0 | 0 | 0 | 0 | 0 | 0 | 0 | 0.463 | 0.463 |
| JUN | F3 | 9606.ENSP00000360266 | 9606.ENSP00000334145 | 0 | 0 | 0 | 0 | 0 | 0 | 0 | 0.416 | 0.416 |
| JUN | ESR2 | 9606.ENSP00000360266 | 9606.ENSP00000343925 | 0 | 0 | 0 | 0 | 0 | 0.066 | 0.8 | 0.795 | 0.958 |
| JUN | MAPK10 | 9606.ENSP00000360266 | 9606.ENSP00000352157 | 0 | 0 | 0 | 0 | 0 | 0.961 | 0.9 | 0.864 | 0.999 |
| JUN | TOP1 | 9606.ENSP00000360266 | 9606.ENSP00000354522 | 0 | 0 | 0 | 0 | 0 | 0.284 | 0 | 0.633 | 0.726 |
| JUN | PTGS2 | 9606.ENSP00000360266 | 9606.ENSP00000356438 | 0 | 0 | 0 | 0 | 0.066 | 0 | 0 | 0.738 | 0.745 |
| JUN | NCOA2 | 9606.ENSP00000360266 | 9606.ENSP00000399968 | 0 | 0 | 0 | 0 | 0 | 0.213 | 0 | 0.307 | 0.432 |
| JUN | TOP2A | 9606.ENSP00000360266 | 9606.ENSP00000411532 | 0 | 0 | 0 | 0 | 0 | 0.298 | 0 | 0.233 | 0.438 |
| JUN | PTPN1 | 9606.ENSP00000360266 | 9606.ENSP00000360683 | 0 | 0 | 0 | 0 | 0 | 0.129 | 0 | 0.422 | 0.475 |
| JUN | CHEK1 | 9606.ENSP00000360266 | 9606.ENSP00000388648 | 0 | 0 | 0 | 0 | 0 | 0.129 | 0 | 0.433 | 0.485 |
| JUN | PLAU | 9606.ENSP00000360266 | 9606.ENSP00000361850 | 0 | 0 | 0 | 0 | 0 | 0 | 0 | 0.502 | 0.502 |
| JUN | PPP3CA | 9606.ENSP00000360266 | 9606.ENSP00000378323 | 0 | 0 | 0 | 0 | 0 | 0.387 | 0 | 0.256 | 0.524 |
| JUN | CDK1 | 9606.ENSP00000360266 | 9606.ENSP00000378699 | 0 | 0 | 0 | 0 | 0.049 | 0.281 | 0 | 0.583 | 0.69 |
| JUN | POR | 9606.ENSP00000360266 | 9606.ENSP00000419970 | 0 | 0 | 0 | 0 | 0 | 0 | 0.9 | 0.157 | 0.912 |
| JUN | AR | 9606.ENSP00000360266 | 9606.ENSP00000363822 | 0 | 0 | 0 | 0 | 0 | 0.499 | 0 | 0.928 | 0.962 |
| JUN | NCOA1 | 9606.ENSP00000360266 | 9606.ENSP00000385216 | 0 | 0 | 0 | 0 | 0 | 0.675 | 0.8 | 0.515 | 0.965 |
| JUN | VEGFA | 9606.ENSP00000360266 | 9606.ENSP00000478570 | 0 | 0 | 0 | 0 | 0.066 | 0.182 | 0.9 | 0.683 | 0.972 |
| JUN | IL6 | 9606.ENSP00000360266 | 9606.ENSP00000385675 | 0 | 0 | 0 | 0 | 0.052 | 0 | 0.9 | 0.822 | 0.981 |
| JUN | TNF | 9606.ENSP00000360266 | 9606.ENSP00000398698 | 0 | 0 | 0 | 0 | 0 | 0 | 0.9 | 0.847 | 0.984 |
| JUN | GSTP1 | 9606.ENSP00000360266 | 9606.ENSP00000381607 | 0 | 0 | 0 | 0 | 0 | 0.094 | 0 | 0.985 | 0.986 |
| JUN | MAPK8 | 9606.ENSP00000360266 | 9606.ENSP00000378974 | 0 | 0 | 0 | 0 | 0 | 0.998 | 0.9 | 0.979 | 0.999 |
| JUN | ESR1 | 9606.ENSP00000360266 | 9606.ENSP00000405330 | 0 | 0 | 0 | 0 | 0 | 0.684 | 0.9 | 0.988 | 0.999 |
| KCNH2 | HTR2A | 9606.ENSP00000262186 | 9606.ENSP00000437737 | 0 | 0 | 0 | 0 | 0 | 0 | 0 | 0.42 | 0.42 |
| KCNH2 | CYP1A2 | 9606.ENSP00000262186 | 9606.ENSP00000342007 | 0 | 0 | 0 | 0 | 0 | 0 | 0 | 0.475 | 0.475 |
| KCNH2 | GJA1 | 9606.ENSP00000262186 | 9606.ENSP00000282561 | 0 | 0 | 0 | 0 | 0.062 | 0 | 0 | 0.51 | 0.52 |
| KCNH2 | CYP3A4 | 9606.ENSP00000262186 | 9606.ENSP00000337915 | 0 | 0 | 0 | 0 | 0 | 0 | 0 | 0.557 | 0.557 |
| KCNH2 | KCNMA1 | 9606.ENSP00000262186 | 9606.ENSP00000286628 | 0 | 0 | 0 | 0 | 0.063 | 0.142 | 0 | 0.657 | 0.7 |
| KCNMA1 | HMOX1 | 9606.ENSP00000286628 | 9606.ENSP00000216117 | 0 | 0 | 0 | 0 | 0 | 0 | 0 | 0.409 | 0.409 |
| KCNMA1 | KCNH2 | 9606.ENSP00000286628 | 9606.ENSP00000262186 | 0 | 0 | 0 | 0 | 0.063 | 0.142 | 0 | 0.657 | 0.7 |
| KCNMA1 | AR | 9606.ENSP00000286628 | 9606.ENSP00000363822 | 0 | 0 | 0 | 0 | 0 | 0 | 0 | 0.422 | 0.422 |
| KCNMA1 | NOS3 | 9606.ENSP00000286628 | 9606.ENSP00000297494 | 0 | 0 | 0 | 0 | 0.062 | 0 | 0 | 0.431 | 0.443 |
| KCNMA1 | PRKACA | 9606.ENSP00000286628 | 9606.ENSP00000309591 | 0 | 0 | 0 | 0 | 0.067 | 0.243 | 0.8 | 0.125 | 0.859 |
| KDR | MAPK1 | 9606.ENSP00000263923 | 9606.ENSP00000215832 | 0 | 0 | 0 | 0.601 | 0 | 0.3 | 0 | 0.434 | 0.415 |
| KDR | MMP2 | 9606.ENSP00000263923 | 9606.ENSP00000219070 | 0 | 0 | 0 | 0 | 0.098 | 0 | 0 | 0.617 | 0.639 |
| KDR | CCL2 | 9606.ENSP00000263923 | 9606.ENSP00000225831 | 0 | 0 | 0 | 0 | 0.065 | 0 | 0 | 0.52 | 0.532 |
| KDR | IL2 | 9606.ENSP00000263923 | 9606.ENSP00000226730 | 0 | 0 | 0 | 0 | 0 | 0 | 0 | 0.414 | 0.414 |
| KDR | MAPK14 | 9606.ENSP00000263923 | 9606.ENSP00000229795 | 0 | 0 | 0 | 0.579 | 0 | 0.081 | 0.9 | 0.528 | 0.924 |
| KDR | IL1B | 9606.ENSP00000263923 | 9606.ENSP00000263341 | 0 | 0 | 0 | 0 | 0.062 | 0 | 0 | 0.619 | 0.627 |
| KDR | PLAU | 9606.ENSP00000263923 | 9606.ENSP00000361850 | 0 | 0 | 0 | 0 | 0.062 | 0 | 0 | 0.404 | 0.417 |
| KDR | F3 | 9606.ENSP00000263923 | 9606.ENSP00000334145 | 0 | 0 | 0 | 0 | 0 | 0 | 0 | 0.421 | 0.421 |
| KDR | MMP3 | 9606.ENSP00000263923 | 9606.ENSP00000299855 | 0 | 0 | 0 | 0 | 0 | 0 | 0 | 0.461 | 0.461 |
| KDR | MMP1 | 9606.ENSP00000263923 | 9606.ENSP00000322788 | 0 | 0 | 0 | 0 | 0 | 0 | 0 | 0.466 | 0.466 |
| KDR | PPARG | 9606.ENSP00000263923 | 9606.ENSP00000287820 | 0 | 0 | 0 | 0 | 0.062 | 0.057 | 0 | 0.456 | 0.476 |
| KDR | ESR1 | 9606.ENSP00000263923 | 9606.ENSP00000405330 | 0 | 0 | 0 | 0 | 0 | 0.057 | 0 | 0.468 | 0.476 |
| KDR | PTGS2 | 9606.ENSP00000263923 | 9606.ENSP00000356438 | 0 | 0 | 0 | 0 | 0 | 0 | 0 | 0.477 | 0.477 |
| KDR | GJA1 | 9606.ENSP00000263923 | 9606.ENSP00000282561 | 0 | 0 | 0 | 0 | 0.188 | 0 | 0 | 0.414 | 0.504 |
| KDR | PIK3CG | 9606.ENSP00000263923 | 9606.ENSP00000352121 | 0 | 0 | 0 | 0 | 0 | 0.306 | 0 | 0.337 | 0.52 |
| KDR | JUN | 9606.ENSP00000263923 | 9606.ENSP00000360266 | 0 | 0 | 0 | 0 | 0 | 0.076 | 0 | 0.513 | 0.531 |
| KDR | IL6 | 9606.ENSP00000263923 | 9606.ENSP00000385675 | 0 | 0 | 0 | 0 | 0 | 0 | 0 | 0.61 | 0.61 |
| KDR | TNF | 9606.ENSP00000263923 | 9606.ENSP00000398698 | 0 | 0 | 0 | 0 | 0 | 0 | 0 | 0.623 | 0.623 |
| KDR | EGFR | 9606.ENSP00000263923 | 9606.ENSP00000275493 | 0 | 0 | 0 | 0.559 | 0.077 | 0.356 | 0 | 0.913 | 0.629 |
| KDR | SELE | 9606.ENSP00000263923 | 9606.ENSP00000331736 | 0 | 0 | 0 | 0 | 0.128 | 0.076 | 0 | 0.601 | 0.65 |
| KDR | TP53 | 9606.ENSP00000263923 | 9606.ENSP00000269305 | 0 | 0 | 0 | 0 | 0 | 0.209 | 0 | 0.633 | 0.697 |
| KDR | PTPN1 | 9606.ENSP00000263923 | 9606.ENSP00000360683 | 0 | 0 | 0 | 0 | 0 | 0.06 | 0 | 0.847 | 0.85 |
| KDR | EGF | 9606.ENSP00000263923 | 9606.ENSP00000265171 | 0 | 0 | 0 | 0 | 0 | 0.057 | 0.6 | 0.734 | 0.891 |
| KDR | PRKACA | 9606.ENSP00000263923 | 9606.ENSP00000309591 | 0 | 0 | 0 | 0.578 | 0 | 0 | 0.9 | 0.101 | 0.902 |
| KDR | NOS3 | 9606.ENSP00000263923 | 9606.ENSP00000297494 | 0 | 0 | 0 | 0 | 0.062 | 0 | 0.9 | 0.835 | 0.983 |
| KDR | VEGFA | 9606.ENSP00000263923 | 9606.ENSP00000478570 | 0 | 0 | 0 | 0 | 0.062 | 0.984 | 0.9 | 0.992 | 0.999 |
| LDLR | MMP2 | 9606.ENSP00000454071 | 9606.ENSP00000219070 | 0 | 0 | 0 | 0 | 0 | 0.385 | 0 | 0.294 | 0.547 |
| LDLR | PLAT | 9606.ENSP00000454071 | 9606.ENSP00000220809 | 0 | 0 | 0 | 0 | 0 | 0.056 | 0 | 0.477 | 0.485 |
| LDLR | PON1 | 9606.ENSP00000454071 | 9606.ENSP00000222381 | 0 | 0 | 0 | 0 | 0 | 0 | 0.54 | 0.344 | 0.685 |
| LDLR | CCL2 | 9606.ENSP00000454071 | 9606.ENSP00000225831 | 0 | 0 | 0 | 0 | 0 | 0 | 0 | 0.517 | 0.517 |
| LDLR | IL1B | 9606.ENSP00000454071 | 9606.ENSP00000263341 | 0 | 0 | 0 | 0 | 0 | 0 | 0 | 0.471 | 0.471 |
| LDLR | EGFR | 9606.ENSP00000454071 | 9606.ENSP00000275493 | 0 | 0 | 0 | 0 | 0.063 | 0.072 | 0 | 0.406 | 0.439 |
| LDLR | PPARG | 9606.ENSP00000454071 | 9606.ENSP00000287820 | 0 | 0 | 0 | 0 | 0 | 0.056 | 0 | 0.595 | 0.601 |
| LDLR | HMGCR | 9606.ENSP00000454071 | 9606.ENSP00000287936 | 0 | 0 | 0 | 0 | 0.165 | 0.062 | 0 | 0.815 | 0.842 |
| LDLR | VCAM1 | 9606.ENSP00000454071 | 9606.ENSP00000294728 | 0 | 0 | 0 | 0 | 0 | 0 | 0 | 0.464 | 0.464 |
| LDLR | NOS3 | 9606.ENSP00000454071 | 9606.ENSP00000297494 | 0 | 0 | 0 | 0 | 0 | 0 | 0 | 0.456 | 0.456 |
| LDLR | FASN | 9606.ENSP00000454071 | 9606.ENSP00000304592 | 0 | 0 | 0 | 0 | 0.085 | 0.068 | 0 | 0.569 | 0.601 |
| LDLR | HSPA5 | 9606.ENSP00000454071 | 9606.ENSP00000324173 | 0 | 0 | 0 | 0 | 0.06 | 0.673 | 0 | 0.674 | 0.891 |
| LDLR | GSK3B | 9606.ENSP00000454071 | 9606.ENSP00000324806 | 0 | 0 | 0 | 0 | 0 | 0.056 | 0 | 0.466 | 0.475 |
| LDLR | PTGER3 | 9606.ENSP00000454071 | 9606.ENSP00000349003 | 0 | 0 | 0 | 0 | 0 | 0.056 | 0 | 0.435 | 0.443 |
| LDLR | SOAT1 | 9606.ENSP00000454071 | 9606.ENSP00000356591 | 0 | 0 | 0 | 0 | 0.062 | 0 | 0 | 0.504 | 0.515 |
| LDLR | PLAU | 9606.ENSP00000454071 | 9606.ENSP00000361850 | 0 | 0 | 0 | 0 | 0.065 | 0.056 | 0 | 0.41 | 0.434 |
| LDLR | IL6 | 9606.ENSP00000454071 | 9606.ENSP00000385675 | 0 | 0 | 0 | 0 | 0 | 0 | 0 | 0.521 | 0.521 |
| LDLR | TNF | 9606.ENSP00000454071 | 9606.ENSP00000398698 | 0 | 0 | 0 | 0 | 0 | 0.062 | 0 | 0.519 | 0.53 |
| LDLR | VEGFA | 9606.ENSP00000454071 | 9606.ENSP00000478570 | 0 | 0 | 0 | 0 | 0.06 | 0 | 0 | 0.393 | 0.405 |
| LDLR | ACACA | 9606.ENSP00000454071 | 9606.ENSP00000483300 | 0 | 0 | 0 | 0 | 0 | 0 | 0 | 0.465 | 0.465 |
| LTA4H | NOS2 | 9606.ENSP00000228740 | 9606.ENSP00000327251 | 0 | 0 | 0 | 0 | 0.062 | 0 | 0 | 0.416 | 0.429 |
| LTA4H | PTGS1 | 9606.ENSP00000228740 | 9606.ENSP00000354612 | 0 | 0 | 0 | 0 | 0.051 | 0 | 0 | 0.444 | 0.45 |
| LTA4H | TNF | 9606.ENSP00000228740 | 9606.ENSP00000398698 | 0 | 0 | 0 | 0 | 0 | 0 | 0 | 0.471 | 0.471 |
| LTA4H | ALOX5 | 9606.ENSP00000228740 | 9606.ENSP00000363512 | 0 | 0 | 0 | 0 | 0 | 0 | 0.9 | 0.79 | 0.978 |
| MAOA | NR3C1 | 9606.ENSP00000340684 | 9606.ENSP00000231509 | 0 | 0 | 0 | 0 | 0 | 0.062 | 0 | 0.556 | 0.565 |
| MAOA | CAT | 9606.ENSP00000340684 | 9606.ENSP00000241052 | 0 | 0 | 0 | 0 | 0.062 | 0 | 0 | 0.446 | 0.459 |
| MAOA | SLC6A4 | 9606.ENSP00000340684 | 9606.ENSP00000261707 | 0 | 0 | 0 | 0 | 0.062 | 0 | 0 | 0.884 | 0.887 |
| MAOA | SLC6A3 | 9606.ENSP00000340684 | 9606.ENSP00000270349 | 0 | 0 | 0 | 0 | 0 | 0 | 0 | 0.804 | 0.804 |
| MAOA | ADRA2A | 9606.ENSP00000340684 | 9606.ENSP00000280155 | 0 | 0 | 0 | 0 | 0.065 | 0 | 0 | 0.426 | 0.44 |
| MAOA | ACHE | 9606.ENSP00000340684 | 9606.ENSP00000303211 | 0.047 | 0 | 0 | 0 | 0.096 | 0 | 0 | 0.603 | 0.628 |
| MAOA | ADH1B | 9606.ENSP00000340684 | 9606.ENSP00000306606 | 0 | 0 | 0 | 0 | 0.104 | 0 | 0.9 | 0.409 | 0.942 |
| MAOA | CYP3A4 | 9606.ENSP00000340684 | 9606.ENSP00000337915 | 0 | 0 | 0 | 0 | 0.064 | 0 | 0.9 | 0.366 | 0.935 |
| MAOA | CYP1A2 | 9606.ENSP00000340684 | 9606.ENSP00000342007 | 0 | 0 | 0 | 0 | 0.063 | 0 | 0 | 0.423 | 0.436 |
| MAOA | HTR3A | 9606.ENSP00000340684 | 9606.ENSP00000347754 | 0 | 0 | 0 | 0 | 0 | 0 | 0 | 0.519 | 0.519 |
| MAOA | OPRM1 | 9606.ENSP00000340684 | 9606.ENSP00000394624 | 0 | 0 | 0 | 0 | 0 | 0 | 0 | 0.52 | 0.52 |
| MAOA | DRD1 | 9606.ENSP00000340684 | 9606.ENSP00000377353 | 0 | 0 | 0 | 0 | 0 | 0 | 0 | 0.614 | 0.614 |
| MAOA | HTR2A | 9606.ENSP00000340684 | 9606.ENSP00000437737 | 0 | 0 | 0 | 0 | 0 | 0 | 0 | 0.782 | 0.782 |
| MAOA | MAOB | 9606.ENSP00000340684 | 9606.ENSP00000367309 | 0 | 0 | 0.448 | 0.976 | 0.18 | 0.307 | 0.8 | 0.918 | 0.88 |
| MAOB | CAT | 9606.ENSP00000367309 | 9606.ENSP00000241052 | 0 | 0 | 0 | 0 | 0.062 | 0 | 0 | 0.503 | 0.514 |
| MAOB | SLC6A4 | 9606.ENSP00000367309 | 9606.ENSP00000261707 | 0 | 0 | 0 | 0 | 0.062 | 0 | 0 | 0.688 | 0.695 |
| MAOB | SLC6A3 | 9606.ENSP00000367309 | 9606.ENSP00000270349 | 0 | 0 | 0 | 0 | 0 | 0 | 0 | 0.784 | 0.785 |
| MAOB | ACHE | 9606.ENSP00000367309 | 9606.ENSP00000303211 | 0.047 | 0 | 0 | 0 | 0.107 | 0 | 0 | 0.662 | 0.687 |
| MAOB | ADH1B | 9606.ENSP00000367309 | 9606.ENSP00000306606 | 0 | 0 | 0 | 0 | 0.104 | 0 | 0.65 | 0.203 | 0.728 |
| MAOB | CYP3A4 | 9606.ENSP00000367309 | 9606.ENSP00000337915 | 0 | 0 | 0 | 0 | 0.064 | 0 | 0.9 | 0.285 | 0.927 |
| MAOB | MAOA | 9606.ENSP00000367309 | 9606.ENSP00000340684 | 0 | 0 | 0.448 | 0.976 | 0.18 | 0.307 | 0.8 | 0.918 | 0.88 |
| MAOB | CYP1A2 | 9606.ENSP00000367309 | 9606.ENSP00000342007 | 0 | 0 | 0 | 0 | 0.063 | 0 | 0 | 0.391 | 0.405 |
| MAOB | HTR3A | 9606.ENSP00000367309 | 9606.ENSP00000347754 | 0 | 0 | 0 | 0 | 0 | 0 | 0 | 0.439 | 0.439 |
| MAOB | CHRM2 | 9606.ENSP00000367309 | 9606.ENSP00000399745 | 0 | 0 | 0 | 0 | 0.06 | 0 | 0 | 0.398 | 0.409 |
| MAOB | DRD1 | 9606.ENSP00000367309 | 9606.ENSP00000377353 | 0 | 0 | 0 | 0 | 0.123 | 0 | 0 | 0.525 | 0.566 |
| MAOB | HTR2A | 9606.ENSP00000367309 | 9606.ENSP00000437737 | 0 | 0 | 0 | 0 | 0.076 | 0 | 0 | 0.597 | 0.611 |
| MAP2 | EGF | 9606.ENSP00000353508 | 9606.ENSP00000265171 | 0 | 0 | 0 | 0 | 0.065 | 0 | 0 | 0.491 | 0.503 |
| MAP2 | PRKACA | 9606.ENSP00000353508 | 9606.ENSP00000309591 | 0 | 0 | 0 | 0 | 0 | 0.391 | 0 | 0.07 | 0.409 |
| MAP2 | GSK3B | 9606.ENSP00000353508 | 9606.ENSP00000324806 | 0 | 0 | 0 | 0 | 0.062 | 0.181 | 0 | 0.36 | 0.465 |
| MAP2 | CHRM2 | 9606.ENSP00000353508 | 9606.ENSP00000399745 | 0 | 0 | 0 | 0 | 0.078 | 0 | 0 | 0.56 | 0.577 |
| MAPK1 | MMP3 | 9606.ENSP00000215832 | 9606.ENSP00000299855 | 0 | 0 | 0 | 0 | 0.049 | 0 | 0 | 0.396 | 0.401 |
| MAPK1 | CCNA2 | 9606.ENSP00000215832 | 9606.ENSP00000274026 | 0 | 0.002 | 0 | 0 | 0.064 | 0.07 | 0 | 0.375 | 0.408 |
| MAPK1 | KDR | 9606.ENSP00000215832 | 9606.ENSP00000263923 | 0 | 0 | 0 | 0.601 | 0 | 0.3 | 0 | 0.434 | 0.415 |
| MAPK1 | IFNG | 9606.ENSP00000215832 | 9606.ENSP00000229135 | 0 | 0 | 0 | 0 | 0 | 0 | 0 | 0.415 | 0.415 |
| MAPK1 | IL1B | 9606.ENSP00000215832 | 9606.ENSP00000263341 | 0 | 0 | 0 | 0 | 0 | 0 | 0 | 0.426 | 0.426 |
| MAPK1 | TOP2A | 9606.ENSP00000215832 | 9606.ENSP00000411532 | 0 | 0 | 0 | 0 | 0.064 | 0.345 | 0 | 0.15 | 0.433 |
| MAPK1 | PIK3CG | 9606.ENSP00000215832 | 9606.ENSP00000352121 | 0 | 0 | 0 | 0 | 0.062 | 0.114 | 0 | 0.401 | 0.459 |
| MAPK1 | INSR | 9606.ENSP00000215832 | 9606.ENSP00000303830 | 0 | 0 | 0 | 0.571 | 0 | 0.351 | 0 | 0.427 | 0.463 |
| MAPK1 | CDK4 | 9606.ENSP00000215832 | 9606.ENSP00000257904 | 0 | 0 | 0.389 | 0.832 | 0.062 | 0.373 | 0 | 0.442 | 0.465 |
| MAPK1 | VEGFA | 9606.ENSP00000215832 | 9606.ENSP00000478570 | 0 | 0 | 0 | 0 | 0 | 0 | 0 | 0.469 | 0.469 |
| MAPK1 | PTGS2 | 9606.ENSP00000215832 | 9606.ENSP00000356438 | 0 | 0 | 0 | 0 | 0 | 0.176 | 0 | 0.393 | 0.478 |
| MAPK1 | CAT | 9606.ENSP00000215832 | 9606.ENSP00000241052 | 0 | 0 | 0 | 0 | 0 | 0.1 | 0 | 0.455 | 0.488 |
| MAPK1 | CHEK1 | 9606.ENSP00000215832 | 9606.ENSP00000388648 | 0 | 0 | 0.303 | 0.633 | 0.062 | 0.355 | 0 | 0.365 | 0.503 |
| MAPK1 | PPP3CA | 9606.ENSP00000215832 | 9606.ENSP00000378323 | 0 | 0 | 0 | 0 | 0.064 | 0.25 | 0 | 0.394 | 0.537 |
| MAPK1 | TNF | 9606.ENSP00000215832 | 9606.ENSP00000398698 | 0 | 0 | 0 | 0 | 0 | 0 | 0 | 0.538 | 0.538 |
| MAPK1 | MAPK10 | 9606.ENSP00000215832 | 9606.ENSP00000352157 | 0 | 0 | 0.368 | 0.892 | 0.081 | 0.056 | 0.5 | 0.604 | 0.574 |
| MAPK1 | PTPN1 | 9606.ENSP00000215832 | 9606.ENSP00000360683 | 0 | 0 | 0 | 0 | 0.092 | 0.382 | 0 | 0.326 | 0.589 |
| MAPK1 | GSK3B | 9606.ENSP00000215832 | 9606.ENSP00000324806 | 0 | 0 | 0.265 | 0.773 | 0.288 | 0.351 | 0 | 0.542 | 0.598 |
| MAPK1 | EGF | 9606.ENSP00000215832 | 9606.ENSP00000265171 | 0 | 0 | 0 | 0 | 0 | 0 | 0 | 0.625 | 0.625 |
| MAPK1 | AR | 9606.ENSP00000215832 | 9606.ENSP00000363822 | 0 | 0 | 0 | 0 | 0 | 0.497 | 0 | 0.324 | 0.645 |
| MAPK1 | PPARG | 9606.ENSP00000215832 | 9606.ENSP00000287820 | 0 | 0 | 0 | 0 | 0 | 0.699 | 0 | 0.403 | 0.812 |
| MAPK1 | PRKACA | 9606.ENSP00000215832 | 9606.ENSP00000309591 | 0 | 0 | 0.357 | 0.611 | 0.065 | 0.18 | 0.8 | 0.257 | 0.867 |
| MAPK1 | ESR2 | 9606.ENSP00000215832 | 9606.ENSP00000343925 | 0 | 0 | 0 | 0 | 0 | 0.317 | 0.8 | 0.304 | 0.896 |
| MAPK1 | GJA1 | 9606.ENSP00000215832 | 9606.ENSP00000282561 | 0 | 0 | 0 | 0 | 0 | 0.462 | 0.8 | 0.209 | 0.907 |
| MAPK1 | RXRB | 9606.ENSP00000215832 | 9606.ENSP00000363817 | 0 | 0 | 0 | 0 | 0 | 0.104 | 0.9 | 0.05 | 0.907 |
| MAPK1 | BAD | 9606.ENSP00000215832 | 9606.ENSP00000378040 | 0 | 0 | 0 | 0 | 0.062 | 0 | 0.9 | 0.169 | 0.915 |
| MAPK1 | F2 | 9606.ENSP00000215832 | 9606.ENSP00000308541 | 0 | 0 | 0 | 0 | 0 | 0.056 | 0.9 | 0.204 | 0.918 |
| MAPK1 | MAPK8 | 9606.ENSP00000215832 | 9606.ENSP00000378974 | 0 | 0 | 0.386 | 0.886 | 0.081 | 0.225 | 0.9 | 0.735 | 0.931 |
| MAPK1 | IL2 | 9606.ENSP00000215832 | 9606.ENSP00000226730 | 0 | 0 | 0 | 0 | 0 | 0 | 0.9 | 0.346 | 0.931 |
| MAPK1 | CDK2 | 9606.ENSP00000215832 | 9606.ENSP00000266970 | 0 | 0 | 0.399 | 0.863 | 0.049 | 0.235 | 0.9 | 0.615 | 0.931 |
| MAPK1 | RB1 | 9606.ENSP00000215832 | 9606.ENSP00000267163 | 0 | 0 | 0 | 0 | 0.062 | 0.213 | 0.9 | 0.296 | 0.941 |
| MAPK1 | NR3C1 | 9606.ENSP00000215832 | 9606.ENSP00000231509 | 0 | 0 | 0 | 0 | 0 | 0.264 | 0.9 | 0.296 | 0.943 |
| MAPK1 | PGR | 9606.ENSP00000215832 | 9606.ENSP00000325120 | 0 | 0 | 0 | 0 | 0 | 0.317 | 0.9 | 0.253 | 0.944 |
| MAPK1 | RXRA | 9606.ENSP00000215832 | 9606.ENSP00000419692 | 0 | 0 | 0 | 0 | 0 | 0.498 | 0.9 | 0.134 | 0.952 |
| MAPK1 | BCL2 | 9606.ENSP00000215832 | 9606.ENSP00000381185 | 0 | 0 | 0 | 0 | 0 | 0.462 | 0.9 | 0.222 | 0.954 |
| MAPK1 | IL6 | 9606.ENSP00000215832 | 9606.ENSP00000385675 | 0 | 0 | 0 | 0 | 0 | 0 | 0.9 | 0.589 | 0.957 |
| MAPK1 | EGFR | 9606.ENSP00000215832 | 9606.ENSP00000275493 | 0 | 0 | 0 | 0.577 | 0 | 0.44 | 0.9 | 0.832 | 0.962 |
| MAPK1 | MAPK14 | 9606.ENSP00000215832 | 9606.ENSP00000229795 | 0 | 0 | 0.412 | 0.926 | 0.106 | 0.874 | 0.8 | 0.776 | 0.977 |
| MAPK1 | ESR1 | 9606.ENSP00000215832 | 9606.ENSP00000405330 | 0 | 0 | 0 | 0 | 0 | 0.699 | 0.9 | 0.532 | 0.984 |
| MAPK1 | MAPK3 | 9606.ENSP00000215832 | 9606.ENSP00000263025 | 0 | 0 | 0.446 | 0.983 | 0 | 0.887 | 0.9 | 0.923 | 0.988 |
| MAPK1 | TP53 | 9606.ENSP00000215832 | 9606.ENSP00000269305 | 0 | 0 | 0 | 0 | 0.055 | 0.876 | 0.8 | 0.695 | 0.991 |
| MAPK1 | JUN | 9606.ENSP00000215832 | 9606.ENSP00000360266 | 0 | 0 | 0 | 0 | 0 | 0.867 | 0.9 | 0.707 | 0.995 |
| MAPK10 | MAPK1 | 9606.ENSP00000352157 | 9606.ENSP00000215832 | 0 | 0 | 0.368 | 0.892 | 0.081 | 0.056 | 0.5 | 0.604 | 0.574 |
| MAPK10 | MAPK14 | 9606.ENSP00000352157 | 9606.ENSP00000229795 | 0 | 0 | 0.347 | 0.921 | 0.066 | 0.056 | 0.6 | 0.711 | 0.646 |
| MAPK10 | NR3C1 | 9606.ENSP00000352157 | 9606.ENSP00000231509 | 0 | 0 | 0 | 0 | 0.062 | 0 | 0.9 | 0.114 | 0.909 |
| MAPK10 | MAPK3 | 9606.ENSP00000352157 | 9606.ENSP00000263025 | 0 | 0 | 0.366 | 0.881 | 0.081 | 0.056 | 0.5 | 0.556 | 0.576 |
| MAPK10 | TP53 | 9606.ENSP00000352157 | 9606.ENSP00000269305 | 0 | 0 | 0 | 0 | 0 | 0.333 | 0.8 | 0.398 | 0.912 |
| MAPK10 | PGR | 9606.ENSP00000352157 | 9606.ENSP00000325120 | 0 | 0 | 0 | 0 | 0.08 | 0 | 0.8 | 0.067 | 0.813 |
| MAPK10 | ESR2 | 9606.ENSP00000352157 | 9606.ENSP00000343925 | 0 | 0 | 0 | 0 | 0.062 | 0 | 0.8 | 0.07 | 0.81 |
| MAPK10 | BCL2 | 9606.ENSP00000352157 | 9606.ENSP00000381185 | 0 | 0 | 0 | 0 | 0 | 0.056 | 0.8 | 0.083 | 0.811 |
| MAPK10 | ESR1 | 9606.ENSP00000352157 | 9606.ENSP00000405330 | 0 | 0 | 0 | 0 | 0 | 0 | 0.8 | 0.158 | 0.824 |
| MAPK10 | MAPK8 | 9606.ENSP00000352157 | 9606.ENSP00000378974 | 0 | 0 | 0.445 | 0.984 | 0.062 | 0 | 0.9 | 0.917 | 0.904 |
| MAPK10 | BAD | 9606.ENSP00000352157 | 9606.ENSP00000378040 | 0 | 0 | 0 | 0 | 0 | 0 | 0.9 | 0.093 | 0.905 |
| MAPK10 | JUN | 9606.ENSP00000352157 | 9606.ENSP00000360266 | 0 | 0 | 0 | 0 | 0 | 0.961 | 0.9 | 0.864 | 0.999 |
| MAPK14 | MAPK1 | 9606.ENSP00000229795 | 9606.ENSP00000215832 | 0 | 0 | 0.412 | 0.926 | 0.106 | 0.874 | 0.8 | 0.776 | 0.977 |
| MAPK14 | HMOX1 | 9606.ENSP00000229795 | 9606.ENSP00000216117 | 0 | 0 | 0 | 0 | 0 | 0 | 0.9 | 0.407 | 0.938 |
| MAPK14 | CCL2 | 9606.ENSP00000229795 | 9606.ENSP00000225831 | 0 | 0 | 0 | 0 | 0 | 0 | 0 | 0.465 | 0.465 |
| MAPK14 | IL2 | 9606.ENSP00000229795 | 9606.ENSP00000226730 | 0 | 0 | 0 | 0 | 0 | 0 | 0.9 | 0.412 | 0.938 |
| MAPK14 | IFNG | 9606.ENSP00000229795 | 9606.ENSP00000229135 | 0 | 0 | 0 | 0 | 0 | 0 | 0 | 0.564 | 0.564 |
| MAPK14 | PIK3CG | 9606.ENSP00000229795 | 9606.ENSP00000352121 | 0 | 0 | 0 | 0 | 0.062 | 0.114 | 0 | 0.346 | 0.409 |
| MAPK14 | CAMKK2 | 9606.ENSP00000229795 | 9606.ENSP00000312741 | 0 | 0 | 0 | 0.63 | 0.063 | 0.396 | 0 | 0.195 | 0.445 |
| MAPK14 | F2 | 9606.ENSP00000229795 | 9606.ENSP00000308541 | 0 | 0 | 0 | 0 | 0 | 0.056 | 0 | 0.444 | 0.452 |
| MAPK14 | PPP3CA | 9606.ENSP00000229795 | 9606.ENSP00000378323 | 0 | 0 | 0 | 0 | 0.103 | 0.25 | 0 | 0.261 | 0.46 |
| MAPK14 | NOS3 | 9606.ENSP00000229795 | 9606.ENSP00000297494 | 0 | 0 | 0 | 0 | 0.049 | 0 | 0 | 0.46 | 0.465 |
| MAPK14 | VCAM1 | 9606.ENSP00000229795 | 9606.ENSP00000294728 | 0 | 0 | 0 | 0 | 0 | 0 | 0 | 0.487 | 0.488 |
| MAPK14 | PTPN1 | 9606.ENSP00000229795 | 9606.ENSP00000360683 | 0 | 0 | 0 | 0 | 0.065 | 0.247 | 0 | 0.341 | 0.495 |
| MAPK14 | NOS2 | 9606.ENSP00000229795 | 9606.ENSP00000327251 | 0 | 0 | 0 | 0 | 0.049 | 0 | 0 | 0.496 | 0.5 |
| MAPK14 | HSPA5 | 9606.ENSP00000229795 | 9606.ENSP00000324173 | 0 | 0 | 0 | 0 | 0.062 | 0.104 | 0 | 0.452 | 0.5 |
| MAPK14 | CAT | 9606.ENSP00000229795 | 9606.ENSP00000241052 | 0 | 0 | 0 | 0 | 0 | 0.117 | 0 | 0.507 | 0.546 |
| MAPK14 | EGF | 9606.ENSP00000229795 | 9606.ENSP00000265171 | 0 | 0 | 0 | 0 | 0 | 0 | 0 | 0.562 | 0.562 |
| MAPK14 | PTGS2 | 9606.ENSP00000229795 | 9606.ENSP00000356438 | 0 | 0 | 0 | 0 | 0 | 0.065 | 0 | 0.624 | 0.633 |
| MAPK14 | MAPK10 | 9606.ENSP00000229795 | 9606.ENSP00000352157 | 0 | 0 | 0.347 | 0.921 | 0.066 | 0.056 | 0.6 | 0.711 | 0.646 |
| MAPK14 | PPARG | 9606.ENSP00000229795 | 9606.ENSP00000287820 | 0 | 0 | 0 | 0 | 0 | 0.104 | 0 | 0.655 | 0.678 |
| MAPK14 | IL6 | 9606.ENSP00000229795 | 9606.ENSP00000385675 | 0 | 0 | 0 | 0 | 0 | 0 | 0 | 0.758 | 0.758 |
| MAPK14 | MAPK8 | 9606.ENSP00000229795 | 9606.ENSP00000378974 | 0 | 0 | 0.396 | 0.924 | 0.066 | 0.47 | 0.6 | 0.814 | 0.803 |
| MAPK14 | EGFR | 9606.ENSP00000229795 | 9606.ENSP00000275493 | 0 | 0 | 0 | 0.577 | 0 | 0.736 | 0 | 0.647 | 0.807 |
| MAPK14 | PGR | 9606.ENSP00000229795 | 9606.ENSP00000325120 | 0 | 0 | 0 | 0 | 0 | 0.104 | 0.8 | 0.232 | 0.85 |
| MAPK14 | ESR2 | 9606.ENSP00000229795 | 9606.ENSP00000343925 | 0 | 0 | 0 | 0 | 0.062 | 0.104 | 0.8 | 0.244 | 0.855 |
| MAPK14 | KDR | 9606.ENSP00000229795 | 9606.ENSP00000263923 | 0 | 0 | 0 | 0.579 | 0 | 0.081 | 0.9 | 0.528 | 0.924 |
| MAPK14 | AR | 9606.ENSP00000229795 | 9606.ENSP00000363822 | 0 | 0 | 0 | 0 | 0 | 0.104 | 0.9 | 0.289 | 0.93 |
| MAPK14 | NR3C1 | 9606.ENSP00000229795 | 9606.ENSP00000231509 | 0 | 0 | 0 | 0 | 0 | 0.104 | 0.9 | 0.334 | 0.935 |
| MAPK14 | VEGFA | 9606.ENSP00000229795 | 9606.ENSP00000478570 | 0 | 0 | 0 | 0 | 0 | 0 | 0.9 | 0.539 | 0.951 |
| MAPK14 | ESR1 | 9606.ENSP00000229795 | 9606.ENSP00000405330 | 0 | 0 | 0 | 0 | 0 | 0.264 | 0.9 | 0.458 | 0.956 |
| MAPK14 | MAPK3 | 9606.ENSP00000229795 | 9606.ENSP00000263025 | 0 | 0 | 0.394 | 0.924 | 0.048 | 0.777 | 0.8 | 0.831 | 0.957 |
| MAPK14 | IL1B | 9606.ENSP00000229795 | 9606.ENSP00000263341 | 0 | 0 | 0 | 0 | 0 | 0 | 0.9 | 0.734 | 0.972 |
| MAPK14 | TNF | 9606.ENSP00000229795 | 9606.ENSP00000398698 | 0 | 0 | 0 | 0 | 0 | 0 | 0.9 | 0.768 | 0.975 |
| MAPK14 | RB1 | 9606.ENSP00000229795 | 9606.ENSP00000267163 | 0 | 0 | 0 | 0 | 0.062 | 0.839 | 0.9 | 0.208 | 0.986 |
| MAPK14 | JUN | 9606.ENSP00000229795 | 9606.ENSP00000360266 | 0 | 0 | 0 | 0 | 0 | 0.4 | 0.9 | 0.838 | 0.989 |
| MAPK14 | TP53 | 9606.ENSP00000229795 | 9606.ENSP00000269305 | 0 | 0 | 0 | 0 | 0 | 0.878 | 0.9 | 0.655 | 0.995 |
| MAPK3 | MAPK1 | 9606.ENSP00000263025 | 9606.ENSP00000215832 | 0 | 0 | 0.446 | 0.983 | 0 | 0.887 | 0.9 | 0.923 | 0.988 |
| MAPK3 | HMOX1 | 9606.ENSP00000263025 | 9606.ENSP00000216117 | 0 | 0 | 0 | 0 | 0 | 0 | 0 | 0.611 | 0.611 |
| MAPK3 | MMP2 | 9606.ENSP00000263025 | 9606.ENSP00000219070 | 0 | 0 | 0 | 0 | 0.049 | 0 | 0 | 0.681 | 0.683 |
| MAPK3 | PLAT | 9606.ENSP00000263025 | 9606.ENSP00000220809 | 0 | 0 | 0 | 0 | 0.049 | 0.235 | 0 | 0.339 | 0.477 |
| MAPK3 | GSR | 9606.ENSP00000263025 | 9606.ENSP00000221130 | 0 | 0 | 0 | 0 | 0.067 | 0 | 0 | 0.395 | 0.412 |
| MAPK3 | MPO | 9606.ENSP00000263025 | 9606.ENSP00000225275 | 0 | 0 | 0 | 0 | 0 | 0.058 | 0 | 0.416 | 0.427 |
| MAPK3 | CCL2 | 9606.ENSP00000263025 | 9606.ENSP00000225831 | 0 | 0 | 0 | 0 | 0 | 0 | 0 | 0.634 | 0.634 |
| MAPK3 | IL2 | 9606.ENSP00000263025 | 9606.ENSP00000226730 | 0 | 0 | 0 | 0 | 0 | 0 | 0.9 | 0.523 | 0.95 |
| MAPK3 | IFNG | 9606.ENSP00000263025 | 9606.ENSP00000229135 | 0 | 0 | 0 | 0 | 0 | 0 | 0 | 0.528 | 0.528 |
| MAPK3 | MAPK14 | 9606.ENSP00000263025 | 9606.ENSP00000229795 | 0 | 0 | 0.394 | 0.924 | 0.048 | 0.777 | 0.8 | 0.831 | 0.957 |
| MAPK3 | NR3C1 | 9606.ENSP00000263025 | 9606.ENSP00000231509 | 0 | 0 | 0 | 0 | 0 | 0.104 | 0.9 | 0.627 | 0.963 |
| MAPK3 | CAT | 9606.ENSP00000263025 | 9606.ENSP00000241052 | 0 | 0 | 0 | 0 | 0 | 0.1 | 0 | 0.673 | 0.693 |
| MAPK3 | SELE | 9606.ENSP00000263025 | 9606.ENSP00000331736 | 0 | 0 | 0 | 0 | 0 | 0 | 0 | 0.401 | 0.401 |
| MAPK3 | PPP3CA | 9606.ENSP00000263025 | 9606.ENSP00000378323 | 0 | 0 | 0 | 0 | 0.064 | 0.25 | 0 | 0.295 | 0.462 |
| MAPK3 | PLAU | 9606.ENSP00000263025 | 9606.ENSP00000361850 | 0 | 0 | 0 | 0 | 0 | 0.056 | 0 | 0.457 | 0.465 |
| MAPK3 | INSR | 9606.ENSP00000263025 | 9606.ENSP00000303830 | 0 | 0 | 0 | 0.573 | 0 | 0.351 | 0 | 0.445 | 0.468 |
| MAPK3 | SOD1 | 9606.ENSP00000263025 | 9606.ENSP00000270142 | 0 | 0 | 0 | 0 | 0.049 | 0.127 | 0 | 0.416 | 0.473 |
| MAPK3 | NOS2 | 9606.ENSP00000263025 | 9606.ENSP00000327251 | 0 | 0 | 0 | 0 | 0.049 | 0 | 0 | 0.478 | 0.483 |
| MAPK3 | FASN | 9606.ENSP00000263025 | 9606.ENSP00000304592 | 0 | 0 | 0 | 0 | 0 | 0.27 | 0 | 0.343 | 0.499 |
| MAPK3 | ADRB2 | 9606.ENSP00000263025 | 9606.ENSP00000305372 | 0 | 0 | 0 | 0 | 0 | 0 | 0 | 0.505 | 0.505 |
| MAPK3 | MMP1 | 9606.ENSP00000263025 | 9606.ENSP00000322788 | 0 | 0 | 0 | 0 | 0.049 | 0 | 0 | 0.506 | 0.51 |
| MAPK3 | MMP3 | 9606.ENSP00000263025 | 9606.ENSP00000299855 | 0 | 0 | 0 | 0 | 0.049 | 0 | 0 | 0.513 | 0.517 |
| MAPK3 | PIK3CG | 9606.ENSP00000263025 | 9606.ENSP00000352121 | 0 | 0 | 0 | 0 | 0.062 | 0.114 | 0 | 0.492 | 0.541 |
| MAPK3 | AR | 9606.ENSP00000263025 | 9606.ENSP00000363822 | 0 | 0 | 0 | 0 | 0 | 0.104 | 0 | 0.517 | 0.548 |
| MAPK3 | CCNA2 | 9606.ENSP00000263025 | 9606.ENSP00000274026 | 0 | 0.001 | 0 | 0 | 0.064 | 0.07 | 0 | 0.526 | 0.551 |
| MAPK3 | VCAM1 | 9606.ENSP00000263025 | 9606.ENSP00000294728 | 0 | 0 | 0 | 0 | 0 | 0 | 0 | 0.564 | 0.564 |
| MAPK3 | MAPK10 | 9606.ENSP00000263025 | 9606.ENSP00000352157 | 0 | 0 | 0.366 | 0.881 | 0.081 | 0.056 | 0.5 | 0.556 | 0.576 |
| MAPK3 | PTPN1 | 9606.ENSP00000263025 | 9606.ENSP00000360683 | 0 | 0 | 0 | 0 | 0.062 | 0.247 | 0 | 0.527 | 0.637 |
| MAPK3 | PPARG | 9606.ENSP00000263025 | 9606.ENSP00000287820 | 0 | 0 | 0 | 0 | 0 | 0.104 | 0 | 0.627 | 0.651 |
| MAPK3 | NOS3 | 9606.ENSP00000263025 | 9606.ENSP00000297494 | 0 | 0 | 0 | 0 | 0.049 | 0 | 0 | 0.681 | 0.683 |
| MAPK3 | IL1B | 9606.ENSP00000263025 | 9606.ENSP00000263341 | 0 | 0 | 0 | 0 | 0 | 0 | 0 | 0.712 | 0.712 |
| MAPK3 | HSPA5 | 9606.ENSP00000263025 | 9606.ENSP00000324173 | 0 | 0 | 0 | 0 | 0.062 | 0.288 | 0 | 0.63 | 0.731 |
| MAPK3 | VEGFA | 9606.ENSP00000263025 | 9606.ENSP00000478570 | 0 | 0 | 0 | 0 | 0 | 0 | 0 | 0.754 | 0.754 |
| MAPK3 | TNF | 9606.ENSP00000263025 | 9606.ENSP00000398698 | 0 | 0 | 0 | 0 | 0 | 0 | 0 | 0.765 | 0.765 |
| MAPK3 | PTGS2 | 9606.ENSP00000263025 | 9606.ENSP00000356438 | 0 | 0 | 0 | 0 | 0 | 0.334 | 0 | 0.682 | 0.78 |
| MAPK3 | EGF | 9606.ENSP00000263025 | 9606.ENSP00000265171 | 0 | 0 | 0 | 0 | 0 | 0 | 0 | 0.82 | 0.82 |
| MAPK3 | PRKACA | 9606.ENSP00000263025 | 9606.ENSP00000309591 | 0 | 0 | 0.369 | 0.611 | 0.106 | 0.18 | 0.8 | 0.189 | 0.87 |
| MAPK3 | RXRB | 9606.ENSP00000263025 | 9606.ENSP00000363817 | 0 | 0 | 0 | 0 | 0 | 0.104 | 0.9 | 0.05 | 0.907 |
| MAPK3 | GJA1 | 9606.ENSP00000263025 | 9606.ENSP00000282561 | 0 | 0 | 0 | 0 | 0 | 0.213 | 0.8 | 0.501 | 0.914 |
| MAPK3 | ESR2 | 9606.ENSP00000263025 | 9606.ENSP00000343925 | 0 | 0 | 0 | 0 | 0 | 0.104 | 0.8 | 0.57 | 0.916 |
| MAPK3 | BAD | 9606.ENSP00000263025 | 9606.ENSP00000378040 | 0 | 0 | 0 | 0 | 0.062 | 0 | 0.9 | 0.298 | 0.928 |
| MAPK3 | CDK2 | 9606.ENSP00000263025 | 9606.ENSP00000266970 | 0 | 0 | 0.405 | 0.872 | 0.049 | 0.235 | 0.9 | 0.664 | 0.931 |
| MAPK3 | F2 | 9606.ENSP00000263025 | 9606.ENSP00000308541 | 0 | 0 | 0 | 0 | 0 | 0.056 | 0.9 | 0.348 | 0.933 |
| MAPK3 | MAPK8 | 9606.ENSP00000263025 | 9606.ENSP00000378974 | 0 | 0 | 0.39 | 0.874 | 0.083 | 0.225 | 0.9 | 0.837 | 0.934 |
| MAPK3 | RXRA | 9606.ENSP00000263025 | 9606.ENSP00000419692 | 0 | 0 | 0 | 0 | 0 | 0.317 | 0.9 | 0.152 | 0.937 |
| MAPK3 | BCL2 | 9606.ENSP00000263025 | 9606.ENSP00000381185 | 0 | 0 | 0 | 0 | 0 | 0.213 | 0.9 | 0.297 | 0.939 |
| MAPK3 | RB1 | 9606.ENSP00000263025 | 9606.ENSP00000267163 | 0 | 0 | 0 | 0 | 0.062 | 0.156 | 0.9 | 0.404 | 0.946 |
| MAPK3 | PGR | 9606.ENSP00000263025 | 9606.ENSP00000325120 | 0 | 0 | 0 | 0 | 0 | 0.104 | 0.9 | 0.588 | 0.959 |
| MAPK3 | EGFR | 9606.ENSP00000263025 | 9606.ENSP00000275493 | 0 | 0 | 0 | 0.58 | 0 | 0.413 | 0.9 | 0.859 | 0.96 |
| MAPK3 | IL6 | 9606.ENSP00000263025 | 9606.ENSP00000385675 | 0 | 0 | 0 | 0 | 0 | 0 | 0.9 | 0.741 | 0.973 |
| MAPK3 | ESR1 | 9606.ENSP00000263025 | 9606.ENSP00000405330 | 0 | 0 | 0 | 0 | 0 | 0.264 | 0.9 | 0.683 | 0.974 |
| MAPK3 | TP53 | 9606.ENSP00000263025 | 9606.ENSP00000269305 | 0 | 0 | 0 | 0 | 0.062 | 0.68 | 0.8 | 0.721 | 0.981 |
| MAPK3 | JUN | 9606.ENSP00000263025 | 9606.ENSP00000360266 | 0 | 0 | 0 | 0 | 0 | 0.5 | 0.9 | 0.852 | 0.991 |
| MAPK8 | MAPK1 | 9606.ENSP00000378974 | 9606.ENSP00000215832 | 0 | 0 | 0.386 | 0.886 | 0.081 | 0.225 | 0.9 | 0.735 | 0.931 |
| MAPK8 | HMOX1 | 9606.ENSP00000378974 | 9606.ENSP00000216117 | 0 | 0 | 0 | 0 | 0 | 0 | 0 | 0.503 | 0.503 |
| MAPK8 | MMP2 | 9606.ENSP00000378974 | 9606.ENSP00000219070 | 0 | 0 | 0 | 0 | 0 | 0 | 0 | 0.511 | 0.511 |
| MAPK8 | CCL2 | 9606.ENSP00000378974 | 9606.ENSP00000225831 | 0 | 0 | 0 | 0 | 0 | 0 | 0 | 0.518 | 0.518 |
| MAPK8 | IL2 | 9606.ENSP00000378974 | 9606.ENSP00000226730 | 0 | 0 | 0 | 0 | 0 | 0 | 0.9 | 0.401 | 0.937 |
| MAPK8 | MAPK14 | 9606.ENSP00000378974 | 9606.ENSP00000229795 | 0 | 0 | 0.396 | 0.924 | 0.066 | 0.47 | 0.6 | 0.814 | 0.803 |
| MAPK8 | NR3C1 | 9606.ENSP00000378974 | 9606.ENSP00000231509 | 0 | 0 | 0 | 0 | 0.065 | 0.213 | 0.9 | 0.292 | 0.94 |
| MAPK8 | CAT | 9606.ENSP00000378974 | 9606.ENSP00000241052 | 0 | 0 | 0 | 0 | 0 | 0 | 0 | 0.501 | 0.501 |
| MAPK8 | MAPK3 | 9606.ENSP00000378974 | 9606.ENSP00000263025 | 0 | 0 | 0.39 | 0.874 | 0.083 | 0.225 | 0.9 | 0.837 | 0.934 |
| MAPK8 | IL1B | 9606.ENSP00000378974 | 9606.ENSP00000263341 | 0 | 0 | 0 | 0 | 0 | 0 | 0 | 0.658 | 0.658 |
| MAPK8 | EGF | 9606.ENSP00000378974 | 9606.ENSP00000265171 | 0 | 0 | 0 | 0 | 0.061 | 0.076 | 0 | 0.523 | 0.549 |
| MAPK8 | RB1 | 9606.ENSP00000378974 | 9606.ENSP00000267163 | 0 | 0 | 0 | 0 | 0 | 0.308 | 0 | 0.198 | 0.422 |
| MAPK8 | TP53 | 9606.ENSP00000378974 | 9606.ENSP00000269305 | 0 | 0 | 0 | 0 | 0 | 0.884 | 0.9 | 0.827 | 0.997 |
| MAPK8 | EGFR | 9606.ENSP00000378974 | 9606.ENSP00000275493 | 0 | 0 | 0 | 0.563 | 0 | 0.642 | 0 | 0.591 | 0.731 |
| MAPK8 | PPARG | 9606.ENSP00000378974 | 9606.ENSP00000287820 | 0 | 0 | 0 | 0 | 0 | 0.213 | 0 | 0.477 | 0.571 |
| MAPK8 | BAX | 9606.ENSP00000378974 | 9606.ENSP00000293288 | 0 | 0 | 0 | 0 | 0 | 0.281 | 0.9 | 0.394 | 0.952 |
| MAPK8 | NOS3 | 9606.ENSP00000378974 | 9606.ENSP00000297494 | 0 | 0 | 0 | 0 | 0.053 | 0 | 0 | 0.466 | 0.472 |
| MAPK8 | MMP1 | 9606.ENSP00000378974 | 9606.ENSP00000322788 | 0 | 0 | 0 | 0 | 0 | 0 | 0 | 0.408 | 0.408 |
| MAPK8 | HSPA5 | 9606.ENSP00000378974 | 9606.ENSP00000324173 | 0 | 0 | 0 | 0 | 0 | 0 | 0 | 0.531 | 0.531 |
| MAPK8 | PGR | 9606.ENSP00000378974 | 9606.ENSP00000325120 | 0 | 0 | 0 | 0 | 0.062 | 0 | 0.8 | 0.246 | 0.846 |
| MAPK8 | NOS2 | 9606.ENSP00000378974 | 9606.ENSP00000327251 | 0 | 0 | 0 | 0 | 0.053 | 0 | 0 | 0.397 | 0.405 |
| MAPK8 | ESR2 | 9606.ENSP00000378974 | 9606.ENSP00000343925 | 0 | 0 | 0 | 0 | 0.062 | 0 | 0.8 | 0.232 | 0.843 |
| MAPK8 | MAPK10 | 9606.ENSP00000378974 | 9606.ENSP00000352157 | 0 | 0 | 0.445 | 0.984 | 0.062 | 0 | 0.9 | 0.917 | 0.904 |
| MAPK8 | PTGS2 | 9606.ENSP00000378974 | 9606.ENSP00000356438 | 0 | 0 | 0 | 0 | 0 | 0 | 0 | 0.564 | 0.564 |
| MAPK8 | JUN | 9606.ENSP00000378974 | 9606.ENSP00000360266 | 0 | 0 | 0 | 0 | 0 | 0.998 | 0.9 | 0.979 | 0.999 |
| MAPK8 | AR | 9606.ENSP00000378974 | 9606.ENSP00000363822 | 0 | 0 | 0 | 0 | 0 | 0 | 0.9 | 0.285 | 0.925 |
| MAPK8 | BAD | 9606.ENSP00000378974 | 9606.ENSP00000378040 | 0 | 0 | 0 | 0 | 0 | 0.682 | 0.9 | 0.244 | 0.973 |
| MAPK8 | PPP3CA | 9606.ENSP00000378974 | 9606.ENSP00000378323 | 0 | 0 | 0 | 0 | 0.052 | 0.064 | 0 | 0.418 | 0.438 |
| MAPK8 | VEGFA | 9606.ENSP00000378974 | 9606.ENSP00000478570 | 0 | 0 | 0 | 0 | 0 | 0 | 0 | 0.517 | 0.517 |
| MAPK8 | IL6 | 9606.ENSP00000378974 | 9606.ENSP00000385675 | 0 | 0 | 0 | 0 | 0 | 0 | 0 | 0.668 | 0.668 |
| MAPK8 | TNF | 9606.ENSP00000378974 | 9606.ENSP00000398698 | 0 | 0 | 0 | 0 | 0.052 | 0 | 0 | 0.696 | 0.7 |
| MAPK8 | ESR1 | 9606.ENSP00000378974 | 9606.ENSP00000405330 | 0 | 0 | 0 | 0 | 0 | 0 | 0.8 | 0.456 | 0.886 |
| MAPK8 | RXRA | 9606.ENSP00000378974 | 9606.ENSP00000419692 | 0 | 0 | 0 | 0 | 0 | 0 | 0.9 | 0.143 | 0.91 |
| MAPK8 | BCL2 | 9606.ENSP00000378974 | 9606.ENSP00000381185 | 0 | 0 | 0 | 0 | 0 | 0.471 | 0.9 | 0.258 | 0.957 |
| MAPK8 | GSTP1 | 9606.ENSP00000378974 | 9606.ENSP00000381607 | 0 | 0 | 0 | 0 | 0 | 0.757 | 0 | 0.982 | 0.995 |
| MMP1 | HMOX1 | 9606.ENSP00000322788 | 9606.ENSP00000216117 | 0 | 0 | 0 | 0 | 0.068 | 0 | 0 | 0.415 | 0.431 |
| MMP1 | MMP2 | 9606.ENSP00000322788 | 9606.ENSP00000219070 | 0 | 0 | 0 | 0.869 | 0.154 | 0 | 0.9 | 0.883 | 0.921 |
| MMP1 | PLAT | 9606.ENSP00000322788 | 9606.ENSP00000220809 | 0 | 0 | 0 | 0 | 0.137 | 0 | 0 | 0.466 | 0.52 |
| MMP1 | MPO | 9606.ENSP00000322788 | 9606.ENSP00000225275 | 0 | 0 | 0 | 0 | 0 | 0 | 0 | 0.405 | 0.405 |
| MMP1 | CCL2 | 9606.ENSP00000322788 | 9606.ENSP00000225831 | 0 | 0 | 0 | 0 | 0.109 | 0.213 | 0 | 0.614 | 0.705 |
| MMP1 | IFNG | 9606.ENSP00000322788 | 9606.ENSP00000229135 | 0 | 0 | 0 | 0 | 0.053 | 0 | 0 | 0.425 | 0.432 |
| MMP1 | MAPK3 | 9606.ENSP00000322788 | 9606.ENSP00000263025 | 0 | 0 | 0 | 0 | 0.049 | 0 | 0 | 0.506 | 0.51 |
| MMP1 | IL1B | 9606.ENSP00000322788 | 9606.ENSP00000263341 | 0 | 0 | 0 | 0 | 0.128 | 0.058 | 0 | 0.742 | 0.77 |
| MMP1 | KDR | 9606.ENSP00000322788 | 9606.ENSP00000263923 | 0 | 0 | 0 | 0 | 0 | 0 | 0 | 0.466 | 0.466 |
| MMP1 | EGF | 9606.ENSP00000322788 | 9606.ENSP00000265171 | 0 | 0 | 0 | 0 | 0 | 0 | 0 | 0.626 | 0.626 |
| MMP1 | TP53 | 9606.ENSP00000322788 | 9606.ENSP00000269305 | 0 | 0 | 0 | 0 | 0 | 0 | 0 | 0.524 | 0.524 |
| MMP1 | EGFR | 9606.ENSP00000322788 | 9606.ENSP00000275493 | 0 | 0 | 0 | 0 | 0.078 | 0 | 0 | 0.516 | 0.535 |
| MMP1 | VCAM1 | 9606.ENSP00000322788 | 9606.ENSP00000294728 | 0 | 0 | 0 | 0 | 0.065 | 0 | 0 | 0.526 | 0.537 |
| MMP1 | MMP3 | 9606.ENSP00000322788 | 9606.ENSP00000299855 | 0 | 0 | 0.298 | 0.953 | 0.529 | 0 | 0.9 | 0.912 | 0.953 |
| MMP1 | PRSS1 | 9606.ENSP00000322788 | 9606.ENSP00000308720 | 0 | 0 | 0 | 0 | 0.066 | 0 | 0.9 | 0.086 | 0.907 |
| MMP1 | MAPK8 | 9606.ENSP00000322788 | 9606.ENSP00000378974 | 0 | 0 | 0 | 0 | 0 | 0 | 0 | 0.408 | 0.408 |
| MMP1 | ESR1 | 9606.ENSP00000322788 | 9606.ENSP00000405330 | 0 | 0 | 0 | 0 | 0 | 0 | 0 | 0.418 | 0.418 |
| MMP1 | SELE | 9606.ENSP00000322788 | 9606.ENSP00000331736 | 0 | 0 | 0 | 0 | 0.052 | 0 | 0 | 0.46 | 0.466 |
| MMP1 | PTGS2 | 9606.ENSP00000322788 | 9606.ENSP00000356438 | 0 | 0 | 0 | 0 | 0.138 | 0 | 0 | 0.618 | 0.657 |
| MMP1 | PLAU | 9606.ENSP00000322788 | 9606.ENSP00000361850 | 0 | 0 | 0 | 0 | 0.198 | 0 | 0 | 0.603 | 0.668 |
| MMP1 | TNF | 9606.ENSP00000322788 | 9606.ENSP00000398698 | 0 | 0 | 0 | 0 | 0.064 | 0 | 0 | 0.7 | 0.707 |
| MMP1 | JUN | 9606.ENSP00000322788 | 9606.ENSP00000360266 | 0 | 0 | 0 | 0 | 0 | 0.628 | 0 | 0.621 | 0.853 |
| MMP1 | VEGFA | 9606.ENSP00000322788 | 9606.ENSP00000478570 | 0 | 0 | 0 | 0 | 0.069 | 0 | 0.9 | 0.683 | 0.967 |
| MMP1 | IL6 | 9606.ENSP00000322788 | 9606.ENSP00000385675 | 0 | 0 | 0 | 0 | 0.154 | 0 | 0.9 | 0.71 | 0.973 |
| MMP2 | HMOX1 | 9606.ENSP00000219070 | 9606.ENSP00000216117 | 0 | 0 | 0 | 0 | 0 | 0 | 0 | 0.486 | 0.486 |
| MMP2 | DPP4 | 9606.ENSP00000219070 | 9606.ENSP00000353731 | 0 | 0 | 0 | 0 | 0.063 | 0 | 0 | 0.391 | 0.404 |
| MMP2 | GSK3B | 9606.ENSP00000219070 | 9606.ENSP00000324806 | 0 | 0 | 0 | 0 | 0 | 0 | 0 | 0.413 | 0.413 |
| MMP2 | AR | 9606.ENSP00000219070 | 9606.ENSP00000363822 | 0 | 0 | 0 | 0 | 0.061 | 0 | 0 | 0.403 | 0.415 |
| MMP2 | IL2 | 9606.ENSP00000219070 | 9606.ENSP00000226730 | 0 | 0 | 0 | 0 | 0 | 0 | 0 | 0.424 | 0.424 |
| MMP2 | PGR | 9606.ENSP00000219070 | 9606.ENSP00000325120 | 0 | 0 | 0 | 0 | 0 | 0 | 0 | 0.426 | 0.426 |
| MMP2 | F3 | 9606.ENSP00000219070 | 9606.ENSP00000334145 | 0 | 0 | 0 | 0 | 0.062 | 0 | 0 | 0.421 | 0.433 |
| MMP2 | IFNG | 9606.ENSP00000219070 | 9606.ENSP00000229135 | 0 | 0 | 0 | 0 | 0 | 0 | 0 | 0.457 | 0.457 |
| MMP2 | CAT | 9606.ENSP00000219070 | 9606.ENSP00000241052 | 0 | 0 | 0 | 0 | 0 | 0 | 0 | 0.46 | 0.459 |
| MMP2 | MMP3 | 9606.ENSP00000219070 | 9606.ENSP00000299855 | 0 | 0 | 0 | 0.88 | 0.18 | 0 | 0.3 | 0.878 | 0.464 |
| MMP2 | CDK2 | 9606.ENSP00000219070 | 9606.ENSP00000266970 | 0 | 0 | 0 | 0 | 0 | 0 | 0 | 0.469 | 0.469 |
| MMP2 | CTSD | 9606.ENSP00000219070 | 9606.ENSP00000236671 | 0 | 0 | 0 | 0 | 0.052 | 0 | 0 | 0.476 | 0.482 |
| MMP2 | CDK4 | 9606.ENSP00000219070 | 9606.ENSP00000257904 | 0 | 0 | 0 | 0 | 0 | 0 | 0 | 0.503 | 0.503 |
| MMP2 | ALOX5 | 9606.ENSP00000219070 | 9606.ENSP00000363512 | 0 | 0 | 0 | 0 | 0 | 0 | 0 | 0.503 | 0.503 |
| MMP2 | SELE | 9606.ENSP00000219070 | 9606.ENSP00000331736 | 0 | 0 | 0 | 0 | 0 | 0 | 0 | 0.504 | 0.504 |
| MMP2 | MPO | 9606.ENSP00000219070 | 9606.ENSP00000225275 | 0 | 0 | 0 | 0 | 0 | 0 | 0 | 0.505 | 0.505 |
| MMP2 | MAPK8 | 9606.ENSP00000219070 | 9606.ENSP00000378974 | 0 | 0 | 0 | 0 | 0 | 0 | 0 | 0.511 | 0.511 |
| MMP2 | PPARG | 9606.ENSP00000219070 | 9606.ENSP00000287820 | 0 | 0 | 0 | 0 | 0.062 | 0 | 0 | 0.504 | 0.514 |
| MMP2 | LDLR | 9606.ENSP00000219070 | 9606.ENSP00000454071 | 0 | 0 | 0 | 0 | 0 | 0.385 | 0 | 0.294 | 0.547 |
| MMP2 | GJA1 | 9606.ENSP00000219070 | 9606.ENSP00000282561 | 0 | 0 | 0 | 0 | 0.229 | 0 | 0 | 0.45 | 0.557 |
| MMP2 | ESR1 | 9606.ENSP00000219070 | 9606.ENSP00000405330 | 0 | 0 | 0 | 0 | 0 | 0 | 0 | 0.56 | 0.56 |
| MMP2 | NOS3 | 9606.ENSP00000219070 | 9606.ENSP00000297494 | 0 | 0 | 0 | 0 | 0 | 0 | 0 | 0.602 | 0.602 |
| MMP2 | PLAT | 9606.ENSP00000219070 | 9606.ENSP00000220809 | 0 | 0 | 0 | 0 | 0.158 | 0 | 0 | 0.575 | 0.627 |
| MMP2 | VCAM1 | 9606.ENSP00000219070 | 9606.ENSP00000294728 | 0 | 0 | 0 | 0 | 0.109 | 0 | 0 | 0.602 | 0.63 |
| MMP2 | KDR | 9606.ENSP00000219070 | 9606.ENSP00000263923 | 0 | 0 | 0 | 0 | 0.098 | 0 | 0 | 0.617 | 0.639 |
| MMP2 | JUN | 9606.ENSP00000219070 | 9606.ENSP00000360266 | 0 | 0 | 0 | 0 | 0 | 0 | 0 | 0.655 | 0.655 |
| MMP2 | PTGS2 | 9606.ENSP00000219070 | 9606.ENSP00000356438 | 0 | 0 | 0 | 0 | 0.066 | 0 | 0 | 0.654 | 0.663 |
| MMP2 | EGF | 9606.ENSP00000219070 | 9606.ENSP00000265171 | 0 | 0 | 0 | 0 | 0 | 0 | 0 | 0.671 | 0.671 |
| MMP2 | TP53 | 9606.ENSP00000219070 | 9606.ENSP00000269305 | 0 | 0 | 0 | 0 | 0.062 | 0 | 0 | 0.669 | 0.676 |
| MMP2 | MAPK3 | 9606.ENSP00000219070 | 9606.ENSP00000263025 | 0 | 0 | 0 | 0 | 0.049 | 0 | 0 | 0.681 | 0.683 |
| MMP2 | CCL2 | 9606.ENSP00000219070 | 9606.ENSP00000225831 | 0 | 0 | 0 | 0 | 0.155 | 0 | 0 | 0.667 | 0.706 |
| MMP2 | EGFR | 9606.ENSP00000219070 | 9606.ENSP00000275493 | 0 | 0 | 0 | 0 | 0.11 | 0 | 0 | 0.684 | 0.707 |
| MMP2 | PLAU | 9606.ENSP00000219070 | 9606.ENSP00000361850 | 0 | 0 | 0 | 0 | 0.152 | 0 | 0 | 0.688 | 0.724 |
| MMP2 | TNF | 9606.ENSP00000219070 | 9606.ENSP00000398698 | 0 | 0 | 0 | 0 | 0 | 0 | 0 | 0.734 | 0.734 |
| MMP2 | IL1B | 9606.ENSP00000219070 | 9606.ENSP00000263341 | 0 | 0 | 0 | 0 | 0 | 0.279 | 0 | 0.703 | 0.776 |
| MMP2 | MMP1 | 9606.ENSP00000219070 | 9606.ENSP00000322788 | 0 | 0 | 0 | 0.869 | 0.154 | 0 | 0.9 | 0.883 | 0.921 |
| MMP2 | IL6 | 9606.ENSP00000219070 | 9606.ENSP00000385675 | 0 | 0 | 0 | 0 | 0.076 | 0 | 0.9 | 0.715 | 0.971 |
| MMP2 | VEGFA | 9606.ENSP00000219070 | 9606.ENSP00000478570 | 0 | 0 | 0 | 0 | 0.065 | 0 | 0.9 | 0.848 | 0.984 |
| MMP3 | MAPK1 | 9606.ENSP00000299855 | 9606.ENSP00000215832 | 0 | 0 | 0 | 0 | 0.049 | 0 | 0 | 0.396 | 0.401 |
| MMP3 | HMOX1 | 9606.ENSP00000299855 | 9606.ENSP00000216117 | 0 | 0 | 0 | 0 | 0 | 0 | 0 | 0.413 | 0.413 |
| MMP3 | MMP2 | 9606.ENSP00000299855 | 9606.ENSP00000219070 | 0 | 0 | 0 | 0.88 | 0.18 | 0 | 0.3 | 0.878 | 0.464 |
| MMP3 | PLAT | 9606.ENSP00000299855 | 9606.ENSP00000220809 | 0 | 0 | 0 | 0 | 0.092 | 0 | 0 | 0.578 | 0.601 |
| MMP3 | MPO | 9606.ENSP00000299855 | 9606.ENSP00000225275 | 0 | 0 | 0 | 0 | 0 | 0 | 0 | 0.455 | 0.455 |
| MMP3 | CCL2 | 9606.ENSP00000299855 | 9606.ENSP00000225831 | 0 | 0 | 0 | 0 | 0.107 | 0.213 | 0 | 0.731 | 0.795 |
| MMP3 | IL2 | 9606.ENSP00000299855 | 9606.ENSP00000226730 | 0 | 0 | 0 | 0 | 0 | 0 | 0 | 0.422 | 0.422 |
| MMP3 | IFNG | 9606.ENSP00000299855 | 9606.ENSP00000229135 | 0 | 0 | 0 | 0 | 0.053 | 0 | 0 | 0.453 | 0.46 |
| MMP3 | CTSD | 9606.ENSP00000299855 | 9606.ENSP00000236671 | 0 | 0 | 0 | 0 | 0.052 | 0 | 0 | 0.419 | 0.426 |
| MMP3 | MAPK3 | 9606.ENSP00000299855 | 9606.ENSP00000263025 | 0 | 0 | 0 | 0 | 0.049 | 0 | 0 | 0.513 | 0.517 |
| MMP3 | IL1B | 9606.ENSP00000299855 | 9606.ENSP00000263341 | 0 | 0 | 0 | 0 | 0.107 | 0.058 | 0 | 0.809 | 0.825 |
| MMP3 | KDR | 9606.ENSP00000299855 | 9606.ENSP00000263923 | 0 | 0 | 0 | 0 | 0 | 0 | 0 | 0.461 | 0.461 |
| MMP3 | EGF | 9606.ENSP00000299855 | 9606.ENSP00000265171 | 0 | 0 | 0 | 0 | 0 | 0 | 0 | 0.548 | 0.548 |
| MMP3 | TP53 | 9606.ENSP00000299855 | 9606.ENSP00000269305 | 0 | 0 | 0 | 0 | 0 | 0 | 0 | 0.523 | 0.523 |
| MMP3 | EGFR | 9606.ENSP00000299855 | 9606.ENSP00000275493 | 0 | 0 | 0 | 0 | 0.076 | 0 | 0.65 | 0.541 | 0.838 |
| MMP3 | VCAM1 | 9606.ENSP00000299855 | 9606.ENSP00000294728 | 0 | 0 | 0 | 0 | 0.062 | 0 | 0 | 0.543 | 0.553 |
| MMP3 | ESR1 | 9606.ENSP00000299855 | 9606.ENSP00000405330 | 0 | 0 | 0 | 0 | 0 | 0 | 0 | 0.435 | 0.435 |
| MMP3 | SELE | 9606.ENSP00000299855 | 9606.ENSP00000331736 | 0 | 0 | 0 | 0 | 0 | 0 | 0 | 0.481 | 0.481 |
| MMP3 | JUN | 9606.ENSP00000299855 | 9606.ENSP00000360266 | 0 | 0 | 0 | 0 | 0 | 0 | 0 | 0.57 | 0.57 |
| MMP3 | PLAU | 9606.ENSP00000299855 | 9606.ENSP00000361850 | 0 | 0 | 0 | 0 | 0.098 | 0 | 0 | 0.605 | 0.628 |
| MMP3 | PTGS2 | 9606.ENSP00000299855 | 9606.ENSP00000356438 | 0 | 0 | 0 | 0 | 0.095 | 0 | 0 | 0.672 | 0.691 |
| MMP3 | TNF | 9606.ENSP00000299855 | 9606.ENSP00000398698 | 0 | 0 | 0 | 0 | 0.064 | 0 | 0 | 0.762 | 0.767 |
| MMP3 | PRSS1 | 9606.ENSP00000299855 | 9606.ENSP00000308720 | 0 | 0 | 0 | 0 | 0 | 0 | 0.9 | 0.047 | 0.9 |
| MMP3 | MMP1 | 9606.ENSP00000299855 | 9606.ENSP00000322788 | 0 | 0 | 0.298 | 0.953 | 0.529 | 0 | 0.9 | 0.912 | 0.953 |
| MMP3 | VEGFA | 9606.ENSP00000299855 | 9606.ENSP00000478570 | 0 | 0 | 0 | 0 | 0 | 0 | 0.9 | 0.671 | 0.965 |
| MMP3 | IL6 | 9606.ENSP00000299855 | 9606.ENSP00000385675 | 0 | 0 | 0 | 0 | 0.112 | 0 | 0.9 | 0.768 | 0.977 |
| MPO | HMOX1 | 9606.ENSP00000225275 | 9606.ENSP00000216117 | 0 | 0 | 0 | 0 | 0 | 0 | 0 | 0.652 | 0.652 |
| MPO | MMP2 | 9606.ENSP00000225275 | 9606.ENSP00000219070 | 0 | 0 | 0 | 0 | 0 | 0 | 0 | 0.505 | 0.505 |
| MPO | GSR | 9606.ENSP00000225275 | 9606.ENSP00000221130 | 0 | 0 | 0 | 0 | 0 | 0.166 | 0 | 0.49 | 0.556 |
| MPO | PON1 | 9606.ENSP00000225275 | 9606.ENSP00000222381 | 0 | 0 | 0 | 0 | 0 | 0 | 0 | 0.923 | 0.923 |
| MPO | MMP1 | 9606.ENSP00000225275 | 9606.ENSP00000322788 | 0 | 0 | 0 | 0 | 0 | 0 | 0 | 0.405 | 0.405 |
| MPO | EGF | 9606.ENSP00000225275 | 9606.ENSP00000265171 | 0 | 0 | 0 | 0 | 0.062 | 0.069 | 0 | 0.386 | 0.417 |
| MPO | GSTP1 | 9606.ENSP00000225275 | 9606.ENSP00000381607 | 0 | 0 | 0 | 0 | 0 | 0 | 0 | 0.421 | 0.42 |
| MPO | TP53 | 9606.ENSP00000225275 | 9606.ENSP00000269305 | 0 | 0 | 0 | 0 | 0 | 0 | 0 | 0.424 | 0.424 |
| MPO | MAPK3 | 9606.ENSP00000225275 | 9606.ENSP00000263025 | 0 | 0 | 0 | 0 | 0 | 0.058 | 0 | 0.416 | 0.427 |
| MPO | XDH | 9606.ENSP00000225275 | 9606.ENSP00000368727 | 0 | 0 | 0 | 0 | 0 | 0 | 0 | 0.448 | 0.448 |
| MPO | MMP3 | 9606.ENSP00000225275 | 9606.ENSP00000299855 | 0 | 0 | 0 | 0 | 0 | 0 | 0 | 0.455 | 0.455 |
| MPO | JUN | 9606.ENSP00000225275 | 9606.ENSP00000360266 | 0 | 0 | 0 | 0 | 0 | 0 | 0 | 0.458 | 0.458 |
| MPO | PPARG | 9606.ENSP00000225275 | 9606.ENSP00000287820 | 0 | 0 | 0 | 0 | 0.062 | 0.056 | 0 | 0.467 | 0.486 |
| MPO | NQO1 | 9606.ENSP00000225275 | 9606.ENSP00000319788 | 0 | 0 | 0 | 0 | 0 | 0 | 0 | 0.505 | 0.505 |
| MPO | F2 | 9606.ENSP00000225275 | 9606.ENSP00000308541 | 0 | 0 | 0 | 0 | 0 | 0.056 | 0 | 0.503 | 0.51 |
| MPO | IL2 | 9606.ENSP00000225275 | 9606.ENSP00000226730 | 0 | 0 | 0 | 0 | 0 | 0 | 0 | 0.516 | 0.516 |
| MPO | ALOX5 | 9606.ENSP00000225275 | 9606.ENSP00000363512 | 0 | 0 | 0 | 0 | 0.121 | 0 | 0 | 0.478 | 0.522 |
| MPO | NOS2 | 9606.ENSP00000225275 | 9606.ENSP00000327251 | 0 | 0 | 0 | 0 | 0 | 0.09 | 0 | 0.535 | 0.559 |
| MPO | VEGFA | 9606.ENSP00000225275 | 9606.ENSP00000478570 | 0 | 0 | 0 | 0 | 0.062 | 0 | 0 | 0.562 | 0.571 |
| MPO | NOS3 | 9606.ENSP00000225275 | 9606.ENSP00000297494 | 0 | 0 | 0 | 0 | 0 | 0.09 | 0 | 0.565 | 0.587 |
| MPO | IFNG | 9606.ENSP00000225275 | 9606.ENSP00000229135 | 0 | 0 | 0 | 0 | 0 | 0 | 0 | 0.593 | 0.593 |
| MPO | F3 | 9606.ENSP00000225275 | 9606.ENSP00000334145 | 0 | 0 | 0 | 0 | 0 | 0 | 0 | 0.594 | 0.594 |
| MPO | VCAM1 | 9606.ENSP00000225275 | 9606.ENSP00000294728 | 0 | 0 | 0 | 0 | 0 | 0 | 0 | 0.633 | 0.633 |
| MPO | SELE | 9606.ENSP00000225275 | 9606.ENSP00000331736 | 0 | 0 | 0 | 0 | 0 | 0.056 | 0 | 0.636 | 0.641 |
| MPO | PTGS2 | 9606.ENSP00000225275 | 9606.ENSP00000356438 | 0 | 0 | 0 | 0 | 0.065 | 0 | 0 | 0.644 | 0.653 |
| MPO | CCL2 | 9606.ENSP00000225275 | 9606.ENSP00000225831 | 0 | 0 | 0 | 0 | 0 | 0 | 0 | 0.682 | 0.682 |
| MPO | CAT | 9606.ENSP00000225275 | 9606.ENSP00000241052 | 0 | 0 | 0 | 0 | 0.061 | 0.241 | 0 | 0.702 | 0.769 |
| MPO | IL1B | 9606.ENSP00000225275 | 9606.ENSP00000263341 | 0 | 0 | 0 | 0 | 0.098 | 0 | 0 | 0.833 | 0.843 |
| MPO | IL6 | 9606.ENSP00000225275 | 9606.ENSP00000385675 | 0 | 0 | 0 | 0 | 0.062 | 0 | 0 | 0.839 | 0.843 |
| MPO | TNF | 9606.ENSP00000225275 | 9606.ENSP00000398698 | 0 | 0 | 0 | 0 | 0.074 | 0 | 0 | 0.861 | 0.866 |
| MPO | PTGS1 | 9606.ENSP00000225275 | 9606.ENSP00000354612 | 0 | 0 | 0 | 0 | 0.076 | 0 | 0.9 | 0.34 | 0.933 |
| NCOA1 | NR3C1 | 9606.ENSP00000385216 | 9606.ENSP00000231509 | 0 | 0 | 0 | 0 | 0.062 | 0.977 | 0.9 | 0.893 | 0.999 |
| NCOA1 | CTSD | 9606.ENSP00000385216 | 9606.ENSP00000236671 | 0 | 0 | 0 | 0 | 0 | 0 | 0.9 | 0.089 | 0.905 |
| NCOA1 | AHR | 9606.ENSP00000385216 | 9606.ENSP00000242057 | 0 | 0 | 0 | 0.547 | 0.062 | 0.599 | 0 | 0.579 | 0.707 |
| NCOA1 | RB1 | 9606.ENSP00000385216 | 9606.ENSP00000267163 | 0 | 0 | 0 | 0 | 0 | 0.105 | 0 | 0.851 | 0.861 |
| NCOA1 | TP53 | 9606.ENSP00000385216 | 9606.ENSP00000269305 | 0 | 0 | 0 | 0 | 0 | 0.27 | 0 | 0.51 | 0.627 |
| NCOA1 | PPARG | 9606.ENSP00000385216 | 9606.ENSP00000287820 | 0 | 0 | 0 | 0 | 0 | 0.984 | 0.9 | 0.987 | 0.999 |
| NCOA1 | HMGCR | 9606.ENSP00000385216 | 9606.ENSP00000287936 | 0 | 0 | 0 | 0 | 0 | 0 | 0.9 | 0.107 | 0.906 |
| NCOA1 | PPARD | 9606.ENSP00000385216 | 9606.ENSP00000310928 | 0 | 0 | 0 | 0 | 0 | 0.828 | 0 | 0.382 | 0.889 |
| NCOA1 | PGR | 9606.ENSP00000385216 | 9606.ENSP00000325120 | 0 | 0 | 0 | 0 | 0 | 0.894 | 0.8 | 0.851 | 0.996 |
| NCOA1 | CYP3A4 | 9606.ENSP00000385216 | 9606.ENSP00000337915 | 0 | 0 | 0 | 0 | 0 | 0 | 0 | 0.458 | 0.457 |
| NCOA1 | ESR2 | 9606.ENSP00000385216 | 9606.ENSP00000343925 | 0 | 0 | 0 | 0 | 0 | 0.977 | 0.9 | 0.971 | 0.999 |
| NCOA1 | NR3C2 | 9606.ENSP00000385216 | 9606.ENSP00000350815 | 0 | 0 | 0 | 0 | 0.072 | 0.881 | 0 | 0.396 | 0.928 |
| NCOA1 | JUN | 9606.ENSP00000385216 | 9606.ENSP00000360266 | 0 | 0 | 0 | 0 | 0 | 0.675 | 0.8 | 0.515 | 0.965 |
| NCOA1 | RXRB | 9606.ENSP00000385216 | 9606.ENSP00000363817 | 0 | 0 | 0 | 0 | 0 | 0.417 | 0.6 | 0.305 | 0.824 |
| NCOA1 | AR | 9606.ENSP00000385216 | 9606.ENSP00000363822 | 0 | 0 | 0 | 0 | 0 | 0.895 | 0.9 | 0.964 | 0.999 |
| NCOA1 | NCOA2 | 9606.ENSP00000385216 | 9606.ENSP00000399968 | 0 | 0 | 0 | 0.817 | 0.083 | 0.836 | 0.9 | 0.887 | 0.986 |
| NCOA1 | RXRA | 9606.ENSP00000385216 | 9606.ENSP00000419692 | 0 | 0 | 0 | 0 | 0 | 0.988 | 0.9 | 0.626 | 0.999 |
| NCOA1 | ESR1 | 9606.ENSP00000385216 | 9606.ENSP00000405330 | 0 | 0 | 0 | 0 | 0 | 0.984 | 0.9 | 0.989 | 0.999 |
| NCOA2 | NR3C1 | 9606.ENSP00000399968 | 9606.ENSP00000231509 | 0 | 0 | 0 | 0 | 0.062 | 0.978 | 0.9 | 0.817 | 0.999 |
| NCOA2 | CTSD | 9606.ENSP00000399968 | 9606.ENSP00000236671 | 0 | 0 | 0 | 0 | 0 | 0 | 0.9 | 0.114 | 0.907 |
| NCOA2 | AHR | 9606.ENSP00000399968 | 9606.ENSP00000242057 | 0 | 0 | 0 | 0.546 | 0 | 0.599 | 0 | 0.334 | 0.654 |
| NCOA2 | TP53 | 9606.ENSP00000399968 | 9606.ENSP00000269305 | 0 | 0 | 0 | 0 | 0 | 0.068 | 0 | 0.493 | 0.508 |
| NCOA2 | EGFR | 9606.ENSP00000399968 | 9606.ENSP00000275493 | 0 | 0 | 0 | 0 | 0.062 | 0 | 0 | 0.643 | 0.65 |
| NCOA2 | PPARG | 9606.ENSP00000399968 | 9606.ENSP00000287820 | 0 | 0 | 0 | 0 | 0.062 | 0.954 | 0.9 | 0.843 | 0.999 |
| NCOA2 | HMGCR | 9606.ENSP00000399968 | 9606.ENSP00000287936 | 0 | 0 | 0 | 0 | 0 | 0 | 0.9 | 0.078 | 0.903 |
| NCOA2 | PPARD | 9606.ENSP00000399968 | 9606.ENSP00000310928 | 0 | 0 | 0 | 0 | 0.049 | 0.713 | 0 | 0.357 | 0.809 |
| NCOA2 | PGR | 9606.ENSP00000399968 | 9606.ENSP00000325120 | 0 | 0 | 0 | 0 | 0 | 0.578 | 0 | 0.569 | 0.81 |
| NCOA2 | ESR2 | 9606.ENSP00000399968 | 9606.ENSP00000343925 | 0 | 0 | 0 | 0 | 0 | 0.847 | 0.9 | 0.952 | 0.999 |
| NCOA2 | NR3C2 | 9606.ENSP00000399968 | 9606.ENSP00000350815 | 0 | 0 | 0 | 0 | 0 | 0.213 | 0 | 0.309 | 0.432 |
| NCOA2 | JUN | 9606.ENSP00000399968 | 9606.ENSP00000360266 | 0 | 0 | 0 | 0 | 0 | 0.213 | 0 | 0.307 | 0.432 |
| NCOA2 | RXRB | 9606.ENSP00000399968 | 9606.ENSP00000363817 | 0 | 0 | 0 | 0 | 0 | 0.705 | 0.6 | 0.509 | 0.937 |
| NCOA2 | AR | 9606.ENSP00000399968 | 9606.ENSP00000363822 | 0 | 0 | 0 | 0 | 0 | 0.977 | 0.9 | 0.954 | 0.999 |
| NCOA2 | NCOA1 | 9606.ENSP00000399968 | 9606.ENSP00000385216 | 0 | 0 | 0 | 0.817 | 0.083 | 0.836 | 0.9 | 0.887 | 0.986 |
| NCOA2 | RXRA | 9606.ENSP00000399968 | 9606.ENSP00000419692 | 0 | 0 | 0 | 0 | 0.062 | 0.983 | 0.9 | 0.505 | 0.999 |
| NCOA2 | ESR1 | 9606.ENSP00000399968 | 9606.ENSP00000405330 | 0 | 0 | 0 | 0 | 0.062 | 0.989 | 0.9 | 0.965 | 0.999 |
| NOS2 | HMOX1 | 9606.ENSP00000327251 | 9606.ENSP00000216117 | 0 | 0 | 0 | 0 | 0 | 0.177 | 0 | 0.667 | 0.714 |
| NOS2 | MPO | 9606.ENSP00000327251 | 9606.ENSP00000225275 | 0 | 0 | 0 | 0 | 0 | 0.09 | 0 | 0.535 | 0.559 |
| NOS2 | CCL2 | 9606.ENSP00000327251 | 9606.ENSP00000225831 | 0 | 0 | 0 | 0 | 0 | 0 | 0 | 0.624 | 0.624 |
| NOS2 | IL2 | 9606.ENSP00000327251 | 9606.ENSP00000226730 | 0 | 0 | 0 | 0 | 0 | 0 | 0 | 0.487 | 0.487 |
| NOS2 | LTA4H | 9606.ENSP00000327251 | 9606.ENSP00000228740 | 0 | 0 | 0 | 0 | 0.062 | 0 | 0 | 0.416 | 0.429 |
| NOS2 | IFNG | 9606.ENSP00000327251 | 9606.ENSP00000229135 | 0 | 0 | 0 | 0 | 0 | 0 | 0 | 0.764 | 0.764 |
| NOS2 | MAPK14 | 9606.ENSP00000327251 | 9606.ENSP00000229795 | 0 | 0 | 0 | 0 | 0.049 | 0 | 0 | 0.496 | 0.5 |
| NOS2 | ODC1 | 9606.ENSP00000327251 | 9606.ENSP00000234111 | 0.043 | 0 | 0 | 0 | 0.062 | 0 | 0 | 0.456 | 0.469 |
| NOS2 | CAT | 9606.ENSP00000327251 | 9606.ENSP00000241052 | 0 | 0 | 0 | 0 | 0.049 | 0 | 0.9 | 0.558 | 0.954 |
| NOS2 | MAPK3 | 9606.ENSP00000327251 | 9606.ENSP00000263025 | 0 | 0 | 0 | 0 | 0.049 | 0 | 0 | 0.478 | 0.483 |
| NOS2 | IL1B | 9606.ENSP00000327251 | 9606.ENSP00000263341 | 0 | 0 | 0 | 0 | 0.068 | 0 | 0 | 0.807 | 0.813 |
| NOS2 | TP53 | 9606.ENSP00000327251 | 9606.ENSP00000269305 | 0 | 0 | 0 | 0 | 0 | 0 | 0 | 0.417 | 0.417 |
| NOS2 | SOD1 | 9606.ENSP00000327251 | 9606.ENSP00000270142 | 0 | 0 | 0 | 0 | 0 | 0.115 | 0 | 0.406 | 0.452 |
| NOS2 | PPARG | 9606.ENSP00000327251 | 9606.ENSP00000287820 | 0 | 0 | 0 | 0 | 0.062 | 0.067 | 0 | 0.526 | 0.549 |
| NOS2 | VCAM1 | 9606.ENSP00000327251 | 9606.ENSP00000294728 | 0 | 0 | 0 | 0 | 0 | 0 | 0 | 0.481 | 0.481 |
| NOS2 | NOS3 | 9606.ENSP00000327251 | 9606.ENSP00000297494 | 0 | 0 | 0.447 | 0.944 | 0 | 0.164 | 0.8 | 0.82 | 0.837 |
| NOS2 | MAPK8 | 9606.ENSP00000327251 | 9606.ENSP00000378974 | 0 | 0 | 0 | 0 | 0.053 | 0 | 0 | 0.397 | 0.405 |
| NOS2 | OPRM1 | 9606.ENSP00000327251 | 9606.ENSP00000394624 | 0 | 0 | 0 | 0 | 0 | 0 | 0 | 0.416 | 0.416 |
| NOS2 | PTGS1 | 9606.ENSP00000327251 | 9606.ENSP00000354612 | 0 | 0 | 0 | 0 | 0.048 | 0.142 | 0 | 0.343 | 0.416 |
| NOS2 | SELE | 9606.ENSP00000327251 | 9606.ENSP00000331736 | 0 | 0 | 0 | 0 | 0.063 | 0 | 0 | 0.405 | 0.419 |
| NOS2 | VEGFA | 9606.ENSP00000327251 | 9606.ENSP00000478570 | 0 | 0 | 0 | 0 | 0.065 | 0 | 0 | 0.518 | 0.53 |
| NOS2 | IL6 | 9606.ENSP00000327251 | 9606.ENSP00000385675 | 0 | 0 | 0 | 0 | 0.063 | 0 | 0 | 0.784 | 0.789 |
| NOS2 | PTGS2 | 9606.ENSP00000327251 | 9606.ENSP00000356438 | 0 | 0 | 0 | 0 | 0.063 | 0.182 | 0 | 0.805 | 0.838 |
| NOS2 | RXRA | 9606.ENSP00000327251 | 9606.ENSP00000419692 | 0 | 0 | 0 | 0 | 0 | 0.067 | 0.9 | 0.047 | 0.903 |
| NOS2 | JUN | 9606.ENSP00000327251 | 9606.ENSP00000360266 | 0 | 0 | 0 | 0 | 0 | 0 | 0.9 | 0.581 | 0.956 |
| NOS2 | TNF | 9606.ENSP00000327251 | 9606.ENSP00000398698 | 0 | 0 | 0 | 0 | 0.063 | 0 | 0.9 | 0.796 | 0.979 |
| NOS3 | HMOX1 | 9606.ENSP00000297494 | 9606.ENSP00000216117 | 0 | 0 | 0 | 0 | 0 | 0.177 | 0 | 0.752 | 0.787 |
| NOS3 | MMP2 | 9606.ENSP00000297494 | 9606.ENSP00000219070 | 0 | 0 | 0 | 0 | 0 | 0 | 0 | 0.602 | 0.602 |
| NOS3 | PLAT | 9606.ENSP00000297494 | 9606.ENSP00000220809 | 0 | 0 | 0 | 0 | 0 | 0 | 0 | 0.458 | 0.457 |
| NOS3 | GSR | 9606.ENSP00000297494 | 9606.ENSP00000221130 | 0 | 0 | 0 | 0 | 0 | 0 | 0 | 0.525 | 0.525 |
| NOS3 | PON1 | 9606.ENSP00000297494 | 9606.ENSP00000222381 | 0 | 0 | 0 | 0 | 0 | 0 | 0 | 0.553 | 0.553 |
| NOS3 | MPO | 9606.ENSP00000297494 | 9606.ENSP00000225275 | 0 | 0 | 0 | 0 | 0 | 0.09 | 0 | 0.565 | 0.587 |
| NOS3 | CCL2 | 9606.ENSP00000297494 | 9606.ENSP00000225831 | 0 | 0 | 0 | 0 | 0 | 0 | 0 | 0.681 | 0.681 |
| NOS3 | IFNG | 9606.ENSP00000297494 | 9606.ENSP00000229135 | 0 | 0 | 0 | 0 | 0 | 0 | 0 | 0.502 | 0.502 |
| NOS3 | MAPK14 | 9606.ENSP00000297494 | 9606.ENSP00000229795 | 0 | 0 | 0 | 0 | 0.049 | 0 | 0 | 0.46 | 0.465 |
| NOS3 | CAT | 9606.ENSP00000297494 | 9606.ENSP00000241052 | 0 | 0 | 0 | 0 | 0.049 | 0 | 0 | 0.744 | 0.746 |
| NOS3 | MAPK3 | 9606.ENSP00000297494 | 9606.ENSP00000263025 | 0 | 0 | 0 | 0 | 0.049 | 0 | 0 | 0.681 | 0.683 |
| NOS3 | IL1B | 9606.ENSP00000297494 | 9606.ENSP00000263341 | 0 | 0 | 0 | 0 | 0.062 | 0 | 0 | 0.689 | 0.696 |
| NOS3 | KDR | 9606.ENSP00000297494 | 9606.ENSP00000263923 | 0 | 0 | 0 | 0 | 0.062 | 0 | 0.9 | 0.835 | 0.983 |
| NOS3 | EGF | 9606.ENSP00000297494 | 9606.ENSP00000265171 | 0 | 0 | 0 | 0 | 0.062 | 0 | 0 | 0.517 | 0.527 |
| NOS3 | TP53 | 9606.ENSP00000297494 | 9606.ENSP00000269305 | 0 | 0 | 0 | 0 | 0 | 0 | 0 | 0.564 | 0.564 |
| NOS3 | SOD1 | 9606.ENSP00000297494 | 9606.ENSP00000270142 | 0 | 0 | 0 | 0 | 0 | 0 | 0 | 0.523 | 0.523 |
| NOS3 | EGFR | 9606.ENSP00000297494 | 9606.ENSP00000275493 | 0 | 0 | 0 | 0 | 0 | 0 | 0 | 0.459 | 0.459 |
| NOS3 | GJA1 | 9606.ENSP00000297494 | 9606.ENSP00000282561 | 0 | 0 | 0 | 0 | 0 | 0 | 0 | 0.518 | 0.518 |
| NOS3 | KCNMA1 | 9606.ENSP00000297494 | 9606.ENSP00000286628 | 0 | 0 | 0 | 0 | 0.062 | 0 | 0 | 0.431 | 0.443 |
| NOS3 | PPARG | 9606.ENSP00000297494 | 9606.ENSP00000287820 | 0 | 0 | 0 | 0 | 0.052 | 0.067 | 0 | 0.673 | 0.686 |
| NOS3 | HMGCR | 9606.ENSP00000297494 | 9606.ENSP00000287936 | 0 | 0 | 0 | 0 | 0.049 | 0 | 0 | 0.525 | 0.529 |
| NOS3 | VCAM1 | 9606.ENSP00000297494 | 9606.ENSP00000294728 | 0 | 0 | 0 | 0 | 0 | 0 | 0 | 0.757 | 0.757 |
| NOS3 | F2 | 9606.ENSP00000297494 | 9606.ENSP00000308541 | 0 | 0 | 0 | 0 | 0 | 0 | 0 | 0.405 | 0.405 |
| NOS3 | HSPA5 | 9606.ENSP00000297494 | 9606.ENSP00000324173 | 0 | 0 | 0 | 0 | 0.053 | 0.056 | 0 | 0.398 | 0.415 |
| NOS3 | ADRB1 | 9606.ENSP00000297494 | 9606.ENSP00000358301 | 0 | 0 | 0 | 0 | 0.062 | 0 | 0 | 0.413 | 0.426 |
| NOS3 | PTPN1 | 9606.ENSP00000297494 | 9606.ENSP00000360683 | 0 | 0 | 0 | 0 | 0.048 | 0.066 | 0 | 0.425 | 0.444 |
| NOS3 | LDLR | 9606.ENSP00000297494 | 9606.ENSP00000454071 | 0 | 0 | 0 | 0 | 0 | 0 | 0 | 0.456 | 0.456 |
| NOS3 | MAPK8 | 9606.ENSP00000297494 | 9606.ENSP00000378974 | 0 | 0 | 0 | 0 | 0.053 | 0 | 0 | 0.466 | 0.472 |
| NOS3 | NR3C2 | 9606.ENSP00000297494 | 9606.ENSP00000350815 | 0 | 0 | 0 | 0 | 0.062 | 0.067 | 0 | 0.451 | 0.477 |
| NOS3 | GSK3B | 9606.ENSP00000297494 | 9606.ENSP00000324806 | 0 | 0 | 0 | 0 | 0.062 | 0.077 | 0 | 0.464 | 0.495 |
| NOS3 | DPP4 | 9606.ENSP00000297494 | 9606.ENSP00000353731 | 0.048 | 0 | 0 | 0 | 0 | 0 | 0 | 0.501 | 0.504 |
| NOS3 | CAMKK2 | 9606.ENSP00000297494 | 9606.ENSP00000312741 | 0 | 0 | 0 | 0 | 0 | 0.214 | 0 | 0.402 | 0.509 |
| NOS3 | ADRB2 | 9606.ENSP00000297494 | 9606.ENSP00000305372 | 0 | 0 | 0 | 0 | 0.062 | 0 | 0 | 0.508 | 0.518 |
| NOS3 | PTGS1 | 9606.ENSP00000297494 | 9606.ENSP00000354612 | 0 | 0 | 0 | 0 | 0.048 | 0.142 | 0 | 0.501 | 0.556 |
| NOS3 | XDH | 9606.ENSP00000297494 | 9606.ENSP00000368727 | 0 | 0 | 0 | 0 | 0 | 0 | 0 | 0.578 | 0.578 |
| NOS3 | F3 | 9606.ENSP00000297494 | 9606.ENSP00000334145 | 0 | 0 | 0 | 0 | 0 | 0 | 0 | 0.6 | 0.6 |
| NOS3 | THBD | 9606.ENSP00000297494 | 9606.ENSP00000366307 | 0 | 0 | 0 | 0 | 0.062 | 0 | 0 | 0.603 | 0.611 |
| NOS3 | SELE | 9606.ENSP00000297494 | 9606.ENSP00000331736 | 0 | 0 | 0 | 0 | 0.079 | 0 | 0 | 0.681 | 0.693 |
| NOS3 | PTGS2 | 9606.ENSP00000297494 | 9606.ENSP00000356438 | 0 | 0 | 0 | 0 | 0.048 | 0.159 | 0 | 0.662 | 0.705 |
| NOS3 | IL6 | 9606.ENSP00000297494 | 9606.ENSP00000385675 | 0 | 0 | 0 | 0 | 0.062 | 0 | 0 | 0.717 | 0.724 |
| NOS3 | TNF | 9606.ENSP00000297494 | 9606.ENSP00000398698 | 0 | 0 | 0 | 0 | 0.062 | 0 | 0 | 0.75 | 0.756 |
| NOS3 | NOS2 | 9606.ENSP00000297494 | 9606.ENSP00000327251 | 0 | 0 | 0.447 | 0.944 | 0 | 0.164 | 0.8 | 0.82 | 0.837 |
| NOS3 | PRKACA | 9606.ENSP00000297494 | 9606.ENSP00000309591 | 0 | 0 | 0 | 0 | 0 | 0.154 | 0.9 | 0.043 | 0.911 |
| NOS3 | JUN | 9606.ENSP00000297494 | 9606.ENSP00000360266 | 0 | 0 | 0 | 0 | 0 | 0 | 0.9 | 0.603 | 0.958 |
| NOS3 | ESR2 | 9606.ENSP00000297494 | 9606.ENSP00000343925 | 0 | 0 | 0 | 0 | 0 | 0.067 | 0.9 | 0.851 | 0.984 |
| NOS3 | VEGFA | 9606.ENSP00000297494 | 9606.ENSP00000478570 | 0 | 0 | 0 | 0 | 0.064 | 0 | 0.9 | 0.857 | 0.985 |
| NOS3 | ESR1 | 9606.ENSP00000297494 | 9606.ENSP00000405330 | 0 | 0 | 0 | 0 | 0 | 0.345 | 0.9 | 0.985 | 0.998 |
| NQO1 | HMOX1 | 9606.ENSP00000319788 | 9606.ENSP00000216117 | 0 | 0 | 0 | 0 | 0.058 | 0 | 0 | 0.915 | 0.916 |
| NQO1 | GSR | 9606.ENSP00000319788 | 9606.ENSP00000221130 | 0 | 0 | 0 | 0 | 0.064 | 0 | 0 | 0.73 | 0.736 |
| NQO1 | MPO | 9606.ENSP00000319788 | 9606.ENSP00000225275 | 0 | 0 | 0 | 0 | 0 | 0 | 0 | 0.505 | 0.505 |
| NQO1 | ODC1 | 9606.ENSP00000319788 | 9606.ENSP00000234111 | 0 | 0 | 0 | 0 | 0 | 0 | 0.8 | 0.475 | 0.89 |
| NQO1 | CAT | 9606.ENSP00000319788 | 9606.ENSP00000241052 | 0 | 0 | 0 | 0 | 0.062 | 0 | 0 | 0.755 | 0.76 |
| NQO1 | AHR | 9606.ENSP00000319788 | 9606.ENSP00000242057 | 0 | 0 | 0 | 0 | 0 | 0 | 0 | 0.688 | 0.688 |
| NQO1 | IL1B | 9606.ENSP00000319788 | 9606.ENSP00000263341 | 0 | 0 | 0 | 0 | 0 | 0 | 0 | 0.564 | 0.564 |
| NQO1 | TP53 | 9606.ENSP00000319788 | 9606.ENSP00000269305 | 0 | 0 | 0 | 0 | 0 | 0.835 | 0 | 0.978 | 0.996 |
| NQO1 | SOD1 | 9606.ENSP00000319788 | 9606.ENSP00000270142 | 0 | 0 | 0 | 0 | 0.062 | 0 | 0 | 0.638 | 0.646 |
| NQO1 | PPARG | 9606.ENSP00000319788 | 9606.ENSP00000287820 | 0 | 0 | 0 | 0 | 0.062 | 0 | 0 | 0.406 | 0.419 |
| NQO1 | HSPA5 | 9606.ENSP00000319788 | 9606.ENSP00000324173 | 0 | 0 | 0 | 0 | 0.065 | 0.104 | 0 | 0.36 | 0.417 |
| NQO1 | CYP3A4 | 9606.ENSP00000319788 | 9606.ENSP00000337915 | 0 | 0 | 0 | 0 | 0 | 0 | 0 | 0.454 | 0.454 |
| NQO1 | POR | 9606.ENSP00000319788 | 9606.ENSP00000419970 | 0 | 0 | 0 | 0 | 0 | 0 | 0 | 0.469 | 0.469 |
| NQO1 | PLAU | 9606.ENSP00000319788 | 9606.ENSP00000361850 | 0 | 0 | 0 | 0 | 0.077 | 0 | 0 | 0.464 | 0.484 |
| NQO1 | ESR1 | 9606.ENSP00000319788 | 9606.ENSP00000405330 | 0 | 0 | 0 | 0 | 0 | 0 | 0 | 0.486 | 0.486 |
| NQO1 | PTGS2 | 9606.ENSP00000319788 | 9606.ENSP00000356438 | 0 | 0 | 0 | 0 | 0 | 0 | 0 | 0.506 | 0.506 |
| NQO1 | JUN | 9606.ENSP00000319788 | 9606.ENSP00000360266 | 0 | 0 | 0 | 0 | 0 | 0 | 0 | 0.517 | 0.517 |
| NQO1 | AKR1B10 | 9606.ENSP00000319788 | 9606.ENSP00000352584 | 0.072 | 0 | 0 | 0 | 0.169 | 0 | 0 | 0.427 | 0.52 |
| NQO1 | CYP1A2 | 9606.ENSP00000319788 | 9606.ENSP00000342007 | 0 | 0 | 0 | 0 | 0 | 0 | 0 | 0.529 | 0.529 |
| NQO1 | AKR1C3 | 9606.ENSP00000319788 | 9606.ENSP00000369927 | 0.072 | 0 | 0 | 0 | 0.163 | 0 | 0 | 0.469 | 0.552 |
| NQO1 | IL6 | 9606.ENSP00000319788 | 9606.ENSP00000385675 | 0 | 0 | 0 | 0 | 0 | 0 | 0 | 0.57 | 0.57 |
| NQO1 | TNF | 9606.ENSP00000319788 | 9606.ENSP00000398698 | 0 | 0 | 0 | 0 | 0 | 0.114 | 0 | 0.577 | 0.609 |
| NQO1 | AKR1C1 | 9606.ENSP00000319788 | 9606.ENSP00000370254 | 0.072 | 0 | 0 | 0 | 0.163 | 0 | 0 | 0.612 | 0.673 |
| NQO1 | GSTP1 | 9606.ENSP00000319788 | 9606.ENSP00000381607 | 0 | 0 | 0 | 0 | 0.065 | 0 | 0 | 0.714 | 0.721 |
| NR3C1 | MAPK1 | 9606.ENSP00000231509 | 9606.ENSP00000215832 | 0 | 0 | 0 | 0 | 0 | 0.264 | 0.9 | 0.296 | 0.943 |
| NR3C1 | CCL2 | 9606.ENSP00000231509 | 9606.ENSP00000225831 | 0 | 0 | 0 | 0 | 0.055 | 0 | 0 | 0.406 | 0.414 |
| NR3C1 | IL2 | 9606.ENSP00000231509 | 9606.ENSP00000226730 | 0 | 0 | 0 | 0 | 0 | 0 | 0 | 0.422 | 0.422 |
| NR3C1 | IFNG | 9606.ENSP00000231509 | 9606.ENSP00000229135 | 0 | 0 | 0 | 0 | 0 | 0 | 0 | 0.401 | 0.401 |
| NR3C1 | MAPK14 | 9606.ENSP00000231509 | 9606.ENSP00000229795 | 0 | 0 | 0 | 0 | 0 | 0.104 | 0.9 | 0.334 | 0.935 |
| NR3C1 | SLC6A3 | 9606.ENSP00000231509 | 9606.ENSP00000270349 | 0 | 0 | 0 | 0 | 0 | 0.057 | 0 | 0.391 | 0.401 |
| NR3C1 | RB1 | 9606.ENSP00000231509 | 9606.ENSP00000267163 | 0 | 0 | 0 | 0 | 0 | 0.077 | 0 | 0.387 | 0.41 |
| NR3C1 | VEGFA | 9606.ENSP00000231509 | 9606.ENSP00000478570 | 0 | 0 | 0 | 0 | 0 | 0 | 0 | 0.42 | 0.42 |
| NR3C1 | HTR3A | 9606.ENSP00000231509 | 9606.ENSP00000347754 | 0 | 0 | 0 | 0 | 0 | 0.056 | 0 | 0.414 | 0.424 |
| NR3C1 | HTR2A | 9606.ENSP00000231509 | 9606.ENSP00000437737 | 0 | 0 | 0 | 0 | 0 | 0.056 | 0 | 0.459 | 0.467 |
| NR3C1 | PTGS2 | 9606.ENSP00000231509 | 9606.ENSP00000356438 | 0 | 0 | 0 | 0 | 0.069 | 0.056 | 0 | 0.465 | 0.488 |
| NR3C1 | PPARG | 9606.ENSP00000231509 | 9606.ENSP00000287820 | 0 | 0 | 0 | 0.578 | 0.062 | 0.27 | 0 | 0.745 | 0.507 |
| NR3C1 | ADRB2 | 9606.ENSP00000231509 | 9606.ENSP00000305372 | 0 | 0 | 0 | 0 | 0 | 0.056 | 0 | 0.543 | 0.55 |
| NR3C1 | MAOA | 9606.ENSP00000231509 | 9606.ENSP00000340684 | 0 | 0 | 0 | 0 | 0 | 0.062 | 0 | 0.556 | 0.565 |
| NR3C1 | CYP3A4 | 9606.ENSP00000231509 | 9606.ENSP00000337915 | 0 | 0 | 0 | 0 | 0 | 0.073 | 0 | 0.582 | 0.596 |
| NR3C1 | IL1B | 9606.ENSP00000231509 | 9606.ENSP00000263341 | 0 | 0 | 0 | 0 | 0 | 0 | 0 | 0.601 | 0.601 |
| NR3C1 | IL6 | 9606.ENSP00000231509 | 9606.ENSP00000385675 | 0 | 0 | 0 | 0 | 0 | 0 | 0 | 0.624 | 0.624 |
| NR3C1 | PGR | 9606.ENSP00000231509 | 9606.ENSP00000325120 | 0 | 0 | 0 | 0.839 | 0 | 0 | 0.6 | 0.862 | 0.655 |
| NR3C1 | SLC6A4 | 9606.ENSP00000231509 | 9606.ENSP00000261707 | 0 | 0 | 0 | 0 | 0 | 0.057 | 0 | 0.681 | 0.686 |
| NR3C1 | AHR | 9606.ENSP00000231509 | 9606.ENSP00000242057 | 0 | 0 | 0 | 0 | 0.059 | 0.057 | 0 | 0.724 | 0.733 |
| NR3C1 | EGFR | 9606.ENSP00000231509 | 9606.ENSP00000275493 | 0 | 0 | 0 | 0 | 0 | 0.664 | 0 | 0.394 | 0.788 |
| NR3C1 | NR3C2 | 9606.ENSP00000231509 | 9606.ENSP00000350815 | 0 | 0 | 0 | 0.861 | 0 | 0.486 | 0.6 | 0.968 | 0.814 |
| NR3C1 | MAPK10 | 9606.ENSP00000231509 | 9606.ENSP00000352157 | 0 | 0 | 0 | 0 | 0.062 | 0 | 0.9 | 0.114 | 0.909 |
| NR3C1 | PRKACA | 9606.ENSP00000231509 | 9606.ENSP00000309591 | 0 | 0 | 0 | 0 | 0.049 | 0.213 | 0.9 | 0.049 | 0.919 |
| NR3C1 | RXRA | 9606.ENSP00000231509 | 9606.ENSP00000419692 | 0 | 0 | 0 | 0.632 | 0 | 0 | 0.9 | 0.643 | 0.923 |
| NR3C1 | ESR1 | 9606.ENSP00000231509 | 9606.ENSP00000405330 | 0 | 0 | 0 | 0.66 | 0 | 0 | 0.9 | 0.89 | 0.93 |
| NR3C1 | AR | 9606.ENSP00000231509 | 9606.ENSP00000363822 | 0 | 0 | 0 | 0.816 | 0 | 0.213 | 0.9 | 0.834 | 0.93 |
| NR3C1 | GSK3B | 9606.ENSP00000231509 | 9606.ENSP00000324806 | 0 | 0 | 0 | 0 | 0 | 0.077 | 0.9 | 0.321 | 0.931 |
| NR3C1 | MAPK8 | 9606.ENSP00000231509 | 9606.ENSP00000378974 | 0 | 0 | 0 | 0 | 0.065 | 0.213 | 0.9 | 0.292 | 0.94 |
| NR3C1 | TNF | 9606.ENSP00000231509 | 9606.ENSP00000398698 | 0 | 0 | 0 | 0 | 0 | 0 | 0.9 | 0.621 | 0.96 |
| NR3C1 | MAPK3 | 9606.ENSP00000231509 | 9606.ENSP00000263025 | 0 | 0 | 0 | 0 | 0 | 0.104 | 0.9 | 0.627 | 0.963 |
| NR3C1 | TP53 | 9606.ENSP00000231509 | 9606.ENSP00000269305 | 0 | 0 | 0 | 0 | 0 | 0.486 | 0.9 | 0.637 | 0.979 |
| NR3C1 | JUN | 9606.ENSP00000231509 | 9606.ENSP00000360266 | 0 | 0 | 0 | 0 | 0 | 0.493 | 0.9 | 0.973 | 0.998 |
| NR3C1 | NCOA1 | 9606.ENSP00000231509 | 9606.ENSP00000385216 | 0 | 0 | 0 | 0 | 0.062 | 0.977 | 0.9 | 0.893 | 0.999 |
| NR3C1 | NCOA2 | 9606.ENSP00000231509 | 9606.ENSP00000399968 | 0 | 0 | 0 | 0 | 0.062 | 0.978 | 0.9 | 0.817 | 0.999 |
| NR3C2 | NR3C1 | 9606.ENSP00000350815 | 9606.ENSP00000231509 | 0 | 0 | 0 | 0.861 | 0 | 0.486 | 0.6 | 0.968 | 0.814 |
| NR3C2 | SLC6A4 | 9606.ENSP00000350815 | 9606.ENSP00000261707 | 0 | 0 | 0 | 0 | 0 | 0.057 | 0 | 0.404 | 0.414 |
| NR3C2 | NOS3 | 9606.ENSP00000350815 | 9606.ENSP00000297494 | 0 | 0 | 0 | 0 | 0.062 | 0.067 | 0 | 0.451 | 0.477 |
| NR3C2 | PGR | 9606.ENSP00000350815 | 9606.ENSP00000325120 | 0 | 0 | 0 | 0.809 | 0.062 | 0 | 0.6 | 0.678 | 0.658 |
| NR3C2 | IL6 | 9606.ENSP00000350815 | 9606.ENSP00000385675 | 0 | 0 | 0 | 0 | 0 | 0 | 0 | 0.417 | 0.417 |
| NR3C2 | NCOA2 | 9606.ENSP00000350815 | 9606.ENSP00000399968 | 0 | 0 | 0 | 0 | 0 | 0.213 | 0 | 0.309 | 0.432 |
| NR3C2 | AR | 9606.ENSP00000350815 | 9606.ENSP00000363822 | 0 | 0 | 0 | 0.779 | 0.062 | 0.213 | 0.6 | 0.705 | 0.728 |
| NR3C2 | NCOA1 | 9606.ENSP00000350815 | 9606.ENSP00000385216 | 0 | 0 | 0 | 0 | 0.072 | 0.881 | 0 | 0.396 | 0.928 |
| ODC1 | EGF | 9606.ENSP00000234111 | 9606.ENSP00000265171 | 0 | 0 | 0 | 0 | 0 | 0 | 0 | 0.424 | 0.424 |
| ODC1 | PTGS2 | 9606.ENSP00000234111 | 9606.ENSP00000356438 | 0 | 0 | 0 | 0 | 0 | 0 | 0 | 0.468 | 0.468 |
| ODC1 | NOS2 | 9606.ENSP00000234111 | 9606.ENSP00000327251 | 0.043 | 0 | 0 | 0 | 0.062 | 0 | 0 | 0.456 | 0.469 |
| ODC1 | AR | 9606.ENSP00000234111 | 9606.ENSP00000363822 | 0 | 0 | 0 | 0 | 0 | 0 | 0 | 0.474 | 0.474 |
| ODC1 | CAT | 9606.ENSP00000234111 | 9606.ENSP00000241052 | 0.043 | 0 | 0 | 0 | 0 | 0 | 0 | 0.481 | 0.482 |
| ODC1 | JUN | 9606.ENSP00000234111 | 9606.ENSP00000360266 | 0 | 0 | 0 | 0 | 0 | 0 | 0 | 0.506 | 0.506 |
| ODC1 | TP53 | 9606.ENSP00000234111 | 9606.ENSP00000269305 | 0 | 0 | 0 | 0 | 0.054 | 0 | 0 | 0.504 | 0.51 |
| ODC1 | NQO1 | 9606.ENSP00000234111 | 9606.ENSP00000319788 | 0 | 0 | 0 | 0 | 0 | 0 | 0.8 | 0.475 | 0.89 |
| OPRD1 | SLC6A3 | 9606.ENSP00000234961 | 9606.ENSP00000270349 | 0 | 0 | 0 | 0 | 0 | 0.057 | 0 | 0.392 | 0.402 |
| OPRD1 | ADRB2 | 9606.ENSP00000234961 | 9606.ENSP00000305372 | 0 | 0 | 0 | 0.674 | 0 | 0.312 | 0 | 0.487 | 0.416 |
| OPRD1 | CHRNA7 | 9606.ENSP00000234961 | 9606.ENSP00000407546 | 0 | 0 | 0 | 0 | 0.074 | 0 | 0 | 0.444 | 0.464 |
| OPRD1 | ADH1B | 9606.ENSP00000234961 | 9606.ENSP00000306606 | 0 | 0 | 0 | 0 | 0 | 0 | 0 | 0.49 | 0.489 |
| OPRD1 | SLC6A4 | 9606.ENSP00000234961 | 9606.ENSP00000261707 | 0 | 0 | 0 | 0 | 0 | 0.057 | 0 | 0.501 | 0.509 |
| OPRD1 | OPRM1 | 9606.ENSP00000234961 | 9606.ENSP00000394624 | 0 | 0 | 0 | 0.961 | 0.069 | 0.501 | 0.3 | 0.859 | 0.658 |
| OPRM1 | IL2 | 9606.ENSP00000394624 | 9606.ENSP00000226730 | 0 | 0 | 0 | 0 | 0 | 0 | 0 | 0.433 | 0.433 |
| OPRM1 | OPRD1 | 9606.ENSP00000394624 | 9606.ENSP00000234961 | 0 | 0 | 0 | 0.961 | 0.069 | 0.501 | 0.3 | 0.859 | 0.658 |
| OPRM1 | SLC6A4 | 9606.ENSP00000394624 | 9606.ENSP00000261707 | 0 | 0 | 0 | 0 | 0 | 0.057 | 0 | 0.681 | 0.686 |
| OPRM1 | SLC6A3 | 9606.ENSP00000394624 | 9606.ENSP00000270349 | 0 | 0 | 0 | 0 | 0 | 0.057 | 0 | 0.681 | 0.686 |
| OPRM1 | ADH1B | 9606.ENSP00000394624 | 9606.ENSP00000306606 | 0 | 0 | 0 | 0 | 0 | 0 | 0 | 0.46 | 0.459 |
| OPRM1 | PGR | 9606.ENSP00000394624 | 9606.ENSP00000325120 | 0 | 0 | 0 | 0 | 0 | 0.056 | 0 | 0.649 | 0.654 |
| OPRM1 | NOS2 | 9606.ENSP00000394624 | 9606.ENSP00000327251 | 0 | 0 | 0 | 0 | 0 | 0 | 0 | 0.416 | 0.416 |
| OPRM1 | CYP3A4 | 9606.ENSP00000394624 | 9606.ENSP00000337915 | 0 | 0 | 0 | 0 | 0 | 0 | 0 | 0.518 | 0.518 |
| OPRM1 | MAOA | 9606.ENSP00000394624 | 9606.ENSP00000340684 | 0 | 0 | 0 | 0 | 0 | 0 | 0 | 0.52 | 0.52 |
| OPRM1 | HTR3A | 9606.ENSP00000394624 | 9606.ENSP00000347754 | 0 | 0 | 0 | 0 | 0 | 0 | 0 | 0.456 | 0.456 |
| OPRM1 | PTGER3 | 9606.ENSP00000394624 | 9606.ENSP00000349003 | 0 | 0 | 0 | 0 | 0 | 0 | 0 | 0.409 | 0.409 |
| PDE3A | PRKACA | 9606.ENSP00000351957 | 9606.ENSP00000309591 | 0 | 0 | 0 | 0 | 0.066 | 0.325 | 0.6 | 0.113 | 0.746 |
| PDE3A | PIK3CG | 9606.ENSP00000351957 | 9606.ENSP00000352121 | 0 | 0 | 0 | 0 | 0 | 0 | 0 | 0.712 | 0.712 |
| PGR | MAPK1 | 9606.ENSP00000325120 | 9606.ENSP00000215832 | 0 | 0 | 0 | 0 | 0 | 0.317 | 0.9 | 0.253 | 0.944 |
| PGR | MMP2 | 9606.ENSP00000325120 | 9606.ENSP00000219070 | 0 | 0 | 0 | 0 | 0 | 0 | 0 | 0.426 | 0.426 |
| PGR | MAPK14 | 9606.ENSP00000325120 | 9606.ENSP00000229795 | 0 | 0 | 0 | 0 | 0 | 0.104 | 0.8 | 0.232 | 0.85 |
| PGR | NR3C1 | 9606.ENSP00000325120 | 9606.ENSP00000231509 | 0 | 0 | 0 | 0.839 | 0 | 0 | 0.6 | 0.862 | 0.655 |
| PGR | CTSD | 9606.ENSP00000325120 | 9606.ENSP00000236671 | 0 | 0 | 0 | 0 | 0 | 0.056 | 0 | 0.602 | 0.608 |
| PGR | AHR | 9606.ENSP00000325120 | 9606.ENSP00000242057 | 0 | 0 | 0 | 0 | 0 | 0.057 | 0 | 0.43 | 0.44 |
| PGR | CDK4 | 9606.ENSP00000325120 | 9606.ENSP00000257904 | 0 | 0 | 0 | 0 | 0 | 0.057 | 0 | 0.468 | 0.476 |
| PGR | MAPK3 | 9606.ENSP00000325120 | 9606.ENSP00000263025 | 0 | 0 | 0 | 0 | 0 | 0.104 | 0.9 | 0.588 | 0.959 |
| PGR | EGF | 9606.ENSP00000325120 | 9606.ENSP00000265171 | 0 | 0 | 0 | 0 | 0 | 0.056 | 0 | 0.687 | 0.692 |
| PGR | CDK2 | 9606.ENSP00000325120 | 9606.ENSP00000266970 | 0 | 0 | 0 | 0 | 0 | 0.494 | 0 | 0.677 | 0.83 |
| PGR | RB1 | 9606.ENSP00000325120 | 9606.ENSP00000267163 | 0 | 0 | 0 | 0 | 0 | 0.077 | 0 | 0.688 | 0.7 |
| PGR | TP53 | 9606.ENSP00000325120 | 9606.ENSP00000269305 | 0 | 0 | 0 | 0 | 0.063 | 0 | 0 | 0.805 | 0.81 |
| PGR | CCNA2 | 9606.ENSP00000325120 | 9606.ENSP00000274026 | 0 | 0 | 0 | 0 | 0 | 0.225 | 0 | 0.405 | 0.519 |
| PGR | EGFR | 9606.ENSP00000325120 | 9606.ENSP00000275493 | 0 | 0 | 0 | 0 | 0 | 0.102 | 0 | 0.833 | 0.844 |
| PGR | PRKACA | 9606.ENSP00000325120 | 9606.ENSP00000309591 | 0 | 0 | 0 | 0 | 0 | 0 | 0.9 | 0 | 0.9 |
| PGR | AKR1C3 | 9606.ENSP00000325120 | 9606.ENSP00000369927 | 0 | 0 | 0 | 0 | 0 | 0.056 | 0 | 0.392 | 0.401 |
| PGR | PLAU | 9606.ENSP00000325120 | 9606.ENSP00000361850 | 0 | 0 | 0 | 0 | 0 | 0 | 0 | 0.402 | 0.402 |
| PGR | TNF | 9606.ENSP00000325120 | 9606.ENSP00000398698 | 0 | 0 | 0 | 0 | 0 | 0 | 0 | 0.406 | 0.406 |
| PGR | GSTP1 | 9606.ENSP00000325120 | 9606.ENSP00000381607 | 0 | 0 | 0 | 0 | 0 | 0.062 | 0 | 0.394 | 0.407 |
| PGR | CYP3A4 | 9606.ENSP00000325120 | 9606.ENSP00000337915 | 0 | 0 | 0 | 0 | 0 | 0.073 | 0 | 0.398 | 0.418 |
| PGR | IL6 | 9606.ENSP00000325120 | 9606.ENSP00000385675 | 0 | 0 | 0 | 0 | 0 | 0 | 0 | 0.459 | 0.459 |
| PGR | TOP2A | 9606.ENSP00000325120 | 9606.ENSP00000411532 | 0 | 0 | 0 | 0 | 0.062 | 0 | 0 | 0.457 | 0.468 |
| PGR | PTGS2 | 9606.ENSP00000325120 | 9606.ENSP00000356438 | 0 | 0 | 0 | 0 | 0.062 | 0.056 | 0 | 0.57 | 0.586 |
| PGR | VEGFA | 9606.ENSP00000325120 | 9606.ENSP00000478570 | 0 | 0 | 0 | 0 | 0 | 0 | 0 | 0.606 | 0.606 |
| PGR | JUN | 9606.ENSP00000325120 | 9606.ENSP00000360266 | 0 | 0 | 0 | 0 | 0 | 0.066 | 0 | 0.627 | 0.637 |
| PGR | OPRM1 | 9606.ENSP00000325120 | 9606.ENSP00000394624 | 0 | 0 | 0 | 0 | 0 | 0.056 | 0 | 0.649 | 0.654 |
| PGR | NR3C2 | 9606.ENSP00000325120 | 9606.ENSP00000350815 | 0 | 0 | 0 | 0.809 | 0.062 | 0 | 0.6 | 0.678 | 0.658 |
| PGR | AR | 9606.ENSP00000325120 | 9606.ENSP00000363822 | 0 | 0 | 0 | 0.813 | 0.069 | 0 | 0.6 | 0.908 | 0.677 |
| PGR | NCOA2 | 9606.ENSP00000325120 | 9606.ENSP00000399968 | 0 | 0 | 0 | 0 | 0 | 0.578 | 0 | 0.569 | 0.81 |
| PGR | MAPK10 | 9606.ENSP00000325120 | 9606.ENSP00000352157 | 0 | 0 | 0 | 0 | 0.08 | 0 | 0.8 | 0.067 | 0.813 |
| PGR | MAPK8 | 9606.ENSP00000325120 | 9606.ENSP00000378974 | 0 | 0 | 0 | 0 | 0.062 | 0 | 0.8 | 0.246 | 0.846 |
| PGR | ESR1 | 9606.ENSP00000325120 | 9606.ENSP00000405330 | 0 | 0 | 0 | 0.679 | 0.102 | 0.825 | 0.6 | 0.965 | 0.952 |
| PGR | NCOA1 | 9606.ENSP00000325120 | 9606.ENSP00000385216 | 0 | 0 | 0 | 0 | 0 | 0.894 | 0.8 | 0.851 | 0.996 |
| PIK3CG | MAPK1 | 9606.ENSP00000352121 | 9606.ENSP00000215832 | 0 | 0 | 0 | 0 | 0.062 | 0.114 | 0 | 0.401 | 0.459 |
| PIK3CG | MAPK14 | 9606.ENSP00000352121 | 9606.ENSP00000229795 | 0 | 0 | 0 | 0 | 0.062 | 0.114 | 0 | 0.346 | 0.409 |
| PIK3CG | MAPK3 | 9606.ENSP00000352121 | 9606.ENSP00000263025 | 0 | 0 | 0 | 0 | 0.062 | 0.114 | 0 | 0.492 | 0.541 |
| PIK3CG | KDR | 9606.ENSP00000352121 | 9606.ENSP00000263923 | 0 | 0 | 0 | 0 | 0 | 0.306 | 0 | 0.337 | 0.52 |
| PIK3CG | TP53 | 9606.ENSP00000352121 | 9606.ENSP00000269305 | 0 | 0 | 0 | 0 | 0 | 0.056 | 0 | 0.475 | 0.483 |
| PIK3CG | EGFR | 9606.ENSP00000352121 | 9606.ENSP00000275493 | 0 | 0 | 0 | 0 | 0 | 0.058 | 0 | 0.537 | 0.545 |
| PIK3CG | INSR | 9606.ENSP00000352121 | 9606.ENSP00000303830 | 0 | 0 | 0 | 0 | 0.063 | 0.143 | 0 | 0.418 | 0.491 |
| PIK3CG | CHRM1 | 9606.ENSP00000352121 | 9606.ENSP00000306490 | 0 | 0 | 0 | 0 | 0 | 0 | 0.6 | 0.05 | 0.604 |
| PIK3CG | PDE3A | 9606.ENSP00000352121 | 9606.ENSP00000351957 | 0 | 0 | 0 | 0 | 0 | 0 | 0 | 0.712 | 0.712 |
| PIK3CG | CHEK1 | 9606.ENSP00000352121 | 9606.ENSP00000388648 | 0 | 0 | 0 | 0 | 0 | 0.245 | 0 | 0.264 | 0.421 |
| PIK3CG | VEGFA | 9606.ENSP00000352121 | 9606.ENSP00000478570 | 0 | 0 | 0 | 0 | 0 | 0 | 0 | 0.451 | 0.451 |
| PIK3CG | TNF | 9606.ENSP00000352121 | 9606.ENSP00000398698 | 0 | 0 | 0 | 0 | 0.129 | 0 | 0 | 0.42 | 0.474 |
| PIK3CG | CHRM2 | 9606.ENSP00000352121 | 9606.ENSP00000399745 | 0 | 0 | 0 | 0 | 0 | 0 | 0.6 | 0.073 | 0.613 |
| PIM1 | EGFR | 9606.ENSP00000362608 | 9606.ENSP00000275493 | 0 | 0 | 0 | 0.582 | 0.048 | 0.642 | 0 | 0.164 | 0.663 |
| PIM1 | PRKACA | 9606.ENSP00000362608 | 9606.ENSP00000309591 | 0 | 0 | 0.373 | 0.651 | 0.103 | 0.347 | 0 | 0.219 | 0.498 |
| PIM1 | BAD | 9606.ENSP00000362608 | 9606.ENSP00000378040 | 0 | 0 | 0 | 0 | 0 | 0.463 | 0 | 0.292 | 0.603 |
| PLAT | MMP2 | 9606.ENSP00000220809 | 9606.ENSP00000219070 | 0 | 0 | 0 | 0 | 0.158 | 0 | 0 | 0.575 | 0.627 |
| PLAT | PLAU | 9606.ENSP00000220809 | 9606.ENSP00000361850 | 0 | 0 | 0.442 | 0.883 | 0.112 | 0.27 | 0 | 0.935 | 0.427 |
| PLAT | F7 | 9606.ENSP00000220809 | 9606.ENSP00000364731 | 0 | 0 | 0.416 | 0.62 | 0 | 0.091 | 0 | 0.703 | 0.429 |
| PLAT | CCL2 | 9606.ENSP00000220809 | 9606.ENSP00000225831 | 0 | 0 | 0 | 0 | 0.073 | 0 | 0 | 0.415 | 0.434 |
| PLAT | NOS3 | 9606.ENSP00000220809 | 9606.ENSP00000297494 | 0 | 0 | 0 | 0 | 0 | 0 | 0 | 0.458 | 0.457 |
| PLAT | IL1B | 9606.ENSP00000220809 | 9606.ENSP00000263341 | 0 | 0 | 0 | 0 | 0 | 0 | 0 | 0.47 | 0.47 |
| PLAT | MAPK3 | 9606.ENSP00000220809 | 9606.ENSP00000263025 | 0 | 0 | 0 | 0 | 0.049 | 0.235 | 0 | 0.339 | 0.477 |
| PLAT | LDLR | 9606.ENSP00000220809 | 9606.ENSP00000454071 | 0 | 0 | 0 | 0 | 0 | 0.056 | 0 | 0.477 | 0.485 |
| PLAT | EGF | 9606.ENSP00000220809 | 9606.ENSP00000265171 | 0 | 0 | 0 | 0 | 0.062 | 0.056 | 0 | 0.473 | 0.493 |
| PLAT | VCAM1 | 9606.ENSP00000220809 | 9606.ENSP00000294728 | 0 | 0 | 0 | 0 | 0.086 | 0 | 0 | 0.476 | 0.501 |
| PLAT | SELE | 9606.ENSP00000220809 | 9606.ENSP00000331736 | 0 | 0 | 0 | 0 | 0 | 0 | 0 | 0.506 | 0.506 |
| PLAT | MMP1 | 9606.ENSP00000220809 | 9606.ENSP00000322788 | 0 | 0 | 0 | 0 | 0.137 | 0 | 0 | 0.466 | 0.52 |
| PLAT | VEGFA | 9606.ENSP00000220809 | 9606.ENSP00000478570 | 0 | 0 | 0 | 0 | 0.085 | 0 | 0 | 0.508 | 0.53 |
| PLAT | TNF | 9606.ENSP00000220809 | 9606.ENSP00000398698 | 0 | 0 | 0 | 0 | 0 | 0 | 0 | 0.537 | 0.537 |
| PLAT | F10 | 9606.ENSP00000220809 | 9606.ENSP00000364709 | 0 | 0 | 0.408 | 0.632 | 0.061 | 0.298 | 0 | 0.67 | 0.552 |
| PLAT | MMP3 | 9606.ENSP00000220809 | 9606.ENSP00000299855 | 0 | 0 | 0 | 0 | 0.092 | 0 | 0 | 0.578 | 0.601 |
| PLAT | IL6 | 9606.ENSP00000220809 | 9606.ENSP00000385675 | 0 | 0 | 0 | 0 | 0.076 | 0 | 0 | 0.611 | 0.625 |
| PLAT | THBD | 9606.ENSP00000220809 | 9606.ENSP00000366307 | 0 | 0 | 0 | 0 | 0.062 | 0 | 0 | 0.711 | 0.717 |
| PLAT | HSPA5 | 9606.ENSP00000220809 | 9606.ENSP00000324173 | 0 | 0 | 0 | 0 | 0 | 0.058 | 0 | 0.778 | 0.782 |
| PLAT | F3 | 9606.ENSP00000220809 | 9606.ENSP00000334145 | 0 | 0 | 0 | 0 | 0.076 | 0 | 0 | 0.801 | 0.808 |
| PLAT | F2 | 9606.ENSP00000220809 | 9606.ENSP00000308541 | 0 | 0 | 0.395 | 0.642 | 0 | 0.653 | 0.9 | 0.739 | 0.976 |
| PLAU | MMP2 | 9606.ENSP00000361850 | 9606.ENSP00000219070 | 0 | 0 | 0 | 0 | 0.152 | 0 | 0 | 0.688 | 0.724 |
| PLAU | PLAT | 9606.ENSP00000361850 | 9606.ENSP00000220809 | 0 | 0 | 0.442 | 0.883 | 0.112 | 0.27 | 0 | 0.935 | 0.427 |
| PLAU | CCL2 | 9606.ENSP00000361850 | 9606.ENSP00000225831 | 0 | 0 | 0 | 0 | 0.122 | 0 | 0 | 0.475 | 0.519 |
| PLAU | CTSD | 9606.ENSP00000361850 | 9606.ENSP00000236671 | 0 | 0 | 0 | 0 | 0.065 | 0 | 0 | 0.459 | 0.472 |
| PLAU | MAPK3 | 9606.ENSP00000361850 | 9606.ENSP00000263025 | 0 | 0 | 0 | 0 | 0 | 0.056 | 0 | 0.457 | 0.465 |
| PLAU | IL1B | 9606.ENSP00000361850 | 9606.ENSP00000263341 | 0 | 0 | 0 | 0 | 0.076 | 0 | 0 | 0.562 | 0.578 |
| PLAU | KDR | 9606.ENSP00000361850 | 9606.ENSP00000263923 | 0 | 0 | 0 | 0 | 0.062 | 0 | 0 | 0.404 | 0.417 |
| PLAU | EGF | 9606.ENSP00000361850 | 9606.ENSP00000265171 | 0 | 0 | 0 | 0 | 0 | 0.056 | 0 | 0.56 | 0.566 |
| PLAU | TP53 | 9606.ENSP00000361850 | 9606.ENSP00000269305 | 0 | 0 | 0 | 0 | 0 | 0 | 0 | 0.56 | 0.56 |
| PLAU | EGFR | 9606.ENSP00000361850 | 9606.ENSP00000275493 | 0 | 0 | 0 | 0 | 0.088 | 0 | 0.9 | 0.626 | 0.962 |
| PLAU | VCAM1 | 9606.ENSP00000361850 | 9606.ENSP00000294728 | 0 | 0 | 0 | 0 | 0.062 | 0 | 0 | 0.391 | 0.404 |
| PLAU | MMP3 | 9606.ENSP00000361850 | 9606.ENSP00000299855 | 0 | 0 | 0 | 0 | 0.098 | 0 | 0 | 0.605 | 0.628 |
| PLAU | F2 | 9606.ENSP00000361850 | 9606.ENSP00000308541 | 0 | 0 | 0.415 | 0.645 | 0 | 0.213 | 0.9 | 0.467 | 0.94 |
| PLAU | NQO1 | 9606.ENSP00000361850 | 9606.ENSP00000319788 | 0 | 0 | 0 | 0 | 0.077 | 0 | 0 | 0.464 | 0.484 |
| PLAU | MMP1 | 9606.ENSP00000361850 | 9606.ENSP00000322788 | 0 | 0 | 0 | 0 | 0.198 | 0 | 0 | 0.603 | 0.668 |
| PLAU | PGR | 9606.ENSP00000361850 | 9606.ENSP00000325120 | 0 | 0 | 0 | 0 | 0 | 0 | 0 | 0.402 | 0.402 |
| PLAU | F3 | 9606.ENSP00000361850 | 9606.ENSP00000334145 | 0 | 0 | 0 | 0 | 0.076 | 0 | 0 | 0.556 | 0.572 |
| PLAU | PTGS2 | 9606.ENSP00000361850 | 9606.ENSP00000356438 | 0 | 0 | 0 | 0 | 0.103 | 0.056 | 0 | 0.469 | 0.511 |
| PLAU | JUN | 9606.ENSP00000361850 | 9606.ENSP00000360266 | 0 | 0 | 0 | 0 | 0 | 0 | 0 | 0.502 | 0.502 |
| PLAU | LDLR | 9606.ENSP00000361850 | 9606.ENSP00000454071 | 0 | 0 | 0 | 0 | 0.065 | 0.056 | 0 | 0.41 | 0.434 |
| PLAU | ESR1 | 9606.ENSP00000361850 | 9606.ENSP00000405330 | 0 | 0 | 0 | 0 | 0.062 | 0 | 0 | 0.452 | 0.463 |
| PLAU | TNF | 9606.ENSP00000361850 | 9606.ENSP00000398698 | 0 | 0 | 0 | 0 | 0.062 | 0 | 0 | 0.491 | 0.502 |
| PLAU | THBD | 9606.ENSP00000361850 | 9606.ENSP00000366307 | 0 | 0 | 0 | 0 | 0.098 | 0 | 0 | 0.519 | 0.548 |
| PLAU | IL6 | 9606.ENSP00000361850 | 9606.ENSP00000385675 | 0 | 0 | 0 | 0 | 0.134 | 0 | 0 | 0.506 | 0.553 |
| PLAU | VEGFA | 9606.ENSP00000361850 | 9606.ENSP00000478570 | 0 | 0 | 0 | 0 | 0.069 | 0 | 0 | 0.652 | 0.662 |
| PON1 | GSR | 9606.ENSP00000222381 | 9606.ENSP00000221130 | 0 | 0 | 0 | 0 | 0 | 0 | 0 | 0.404 | 0.404 |
| PON1 | VCAM1 | 9606.ENSP00000222381 | 9606.ENSP00000294728 | 0 | 0 | 0 | 0 | 0.058 | 0 | 0 | 0.392 | 0.402 |
| PON1 | GSTP1 | 9606.ENSP00000222381 | 9606.ENSP00000381607 | 0 | 0 | 0 | 0 | 0.062 | 0 | 0 | 0.402 | 0.415 |
| PON1 | CCL2 | 9606.ENSP00000222381 | 9606.ENSP00000225831 | 0 | 0 | 0 | 0 | 0 | 0 | 0 | 0.452 | 0.452 |
| PON1 | CYP1A2 | 9606.ENSP00000222381 | 9606.ENSP00000342007 | 0 | 0 | 0 | 0 | 0 | 0 | 0 | 0.459 | 0.459 |
| PON1 | TNF | 9606.ENSP00000222381 | 9606.ENSP00000398698 | 0 | 0 | 0 | 0 | 0 | 0 | 0 | 0.468 | 0.468 |
| PON1 | PPARG | 9606.ENSP00000222381 | 9606.ENSP00000287820 | 0 | 0 | 0 | 0 | 0.062 | 0 | 0 | 0.46 | 0.471 |
| PON1 | CYP3A4 | 9606.ENSP00000222381 | 9606.ENSP00000337915 | 0 | 0 | 0 | 0 | 0.098 | 0 | 0 | 0.451 | 0.483 |
| PON1 | IL6 | 9606.ENSP00000222381 | 9606.ENSP00000385675 | 0 | 0 | 0 | 0 | 0 | 0 | 0 | 0.505 | 0.505 |
| PON1 | NOS3 | 9606.ENSP00000222381 | 9606.ENSP00000297494 | 0 | 0 | 0 | 0 | 0 | 0 | 0 | 0.553 | 0.553 |
| PON1 | CAT | 9606.ENSP00000222381 | 9606.ENSP00000241052 | 0 | 0 | 0 | 0 | 0.062 | 0 | 0 | 0.596 | 0.604 |
| PON1 | ACHE | 9606.ENSP00000222381 | 9606.ENSP00000303211 | 0 | 0 | 0 | 0 | 0.063 | 0 | 0 | 0.659 | 0.667 |
| PON1 | LDLR | 9606.ENSP00000222381 | 9606.ENSP00000454071 | 0 | 0 | 0 | 0 | 0 | 0 | 0.54 | 0.344 | 0.685 |
| PON1 | MPO | 9606.ENSP00000222381 | 9606.ENSP00000225275 | 0 | 0 | 0 | 0 | 0 | 0 | 0 | 0.923 | 0.923 |
| POR | HMOX1 | 9606.ENSP00000419970 | 9606.ENSP00000216117 | 0 | 0 | 0 | 0 | 0.072 | 0.324 | 0 | 0.865 | 0.908 |
| POR | CAT | 9606.ENSP00000419970 | 9606.ENSP00000241052 | 0 | 0 | 0 | 0 | 0.049 | 0 | 0 | 0.452 | 0.457 |
| POR | NQO1 | 9606.ENSP00000419970 | 9606.ENSP00000319788 | 0 | 0 | 0 | 0 | 0 | 0 | 0 | 0.469 | 0.469 |
| POR | CYP3A4 | 9606.ENSP00000419970 | 9606.ENSP00000337915 | 0.042 | 0.384 | 0 | 0 | 0 | 0.282 | 0 | 0.941 | 0.972 |
| POR | CYP1A2 | 9606.ENSP00000419970 | 9606.ENSP00000342007 | 0.042 | 0 | 0 | 0 | 0 | 0.494 | 0 | 0.77 | 0.879 |
| POR | JUN | 9606.ENSP00000419970 | 9606.ENSP00000360266 | 0 | 0 | 0 | 0 | 0 | 0 | 0.9 | 0.157 | 0.912 |
| POR | AKR1C3 | 9606.ENSP00000419970 | 9606.ENSP00000369927 | 0 | 0 | 0 | 0 | 0 | 0 | 0 | 0.402 | 0.402 |
| PPARD | FASN | 9606.ENSP00000310928 | 9606.ENSP00000304592 | 0 | 0 | 0 | 0 | 0.062 | 0 | 0 | 0.432 | 0.445 |
| PPARD | JUN | 9606.ENSP00000310928 | 9606.ENSP00000360266 | 0 | 0 | 0 | 0 | 0 | 0.066 | 0 | 0.394 | 0.41 |
| PPARD | IL6 | 9606.ENSP00000310928 | 9606.ENSP00000385675 | 0 | 0 | 0 | 0 | 0 | 0 | 0 | 0.415 | 0.415 |
| PPARD | TNF | 9606.ENSP00000310928 | 9606.ENSP00000398698 | 0 | 0 | 0 | 0 | 0 | 0 | 0 | 0.451 | 0.451 |
| PPARD | PTGS2 | 9606.ENSP00000310928 | 9606.ENSP00000356438 | 0 | 0 | 0 | 0 | 0 | 0.056 | 0 | 0.442 | 0.451 |
| PPARD | NCOA2 | 9606.ENSP00000310928 | 9606.ENSP00000399968 | 0 | 0 | 0 | 0 | 0.049 | 0.713 | 0 | 0.357 | 0.809 |
| PPARD | NCOA1 | 9606.ENSP00000310928 | 9606.ENSP00000385216 | 0 | 0 | 0 | 0 | 0 | 0.828 | 0 | 0.382 | 0.889 |
| PPARD | RXRB | 9606.ENSP00000310928 | 9606.ENSP00000363817 | 0 | 0 | 0 | 0.712 | 0.072 | 0.417 | 0.9 | 0.749 | 0.953 |
| PPARD | RXRA | 9606.ENSP00000310928 | 9606.ENSP00000419692 | 0 | 0 | 0 | 0.705 | 0.062 | 0.684 | 0.9 | 0.852 | 0.975 |
| PPARG | MAPK1 | 9606.ENSP00000287820 | 9606.ENSP00000215832 | 0 | 0 | 0 | 0 | 0 | 0.699 | 0 | 0.403 | 0.812 |
| PPARG | HMOX1 | 9606.ENSP00000287820 | 9606.ENSP00000216117 | 0 | 0 | 0 | 0 | 0 | 0 | 0 | 0.654 | 0.654 |
| PPARG | MMP2 | 9606.ENSP00000287820 | 9606.ENSP00000219070 | 0 | 0 | 0 | 0 | 0.062 | 0 | 0 | 0.504 | 0.514 |
| PPARG | GSR | 9606.ENSP00000287820 | 9606.ENSP00000221130 | 0 | 0 | 0 | 0 | 0 | 0.057 | 0 | 0.392 | 0.402 |
| PPARG | PON1 | 9606.ENSP00000287820 | 9606.ENSP00000222381 | 0 | 0 | 0 | 0 | 0.062 | 0 | 0 | 0.46 | 0.471 |
| PPARG | MPO | 9606.ENSP00000287820 | 9606.ENSP00000225275 | 0 | 0 | 0 | 0 | 0.062 | 0.056 | 0 | 0.467 | 0.486 |
| PPARG | CCL2 | 9606.ENSP00000287820 | 9606.ENSP00000225831 | 0 | 0 | 0 | 0 | 0.055 | 0 | 0 | 0.705 | 0.709 |
| PPARG | IL2 | 9606.ENSP00000287820 | 9606.ENSP00000226730 | 0 | 0 | 0 | 0 | 0 | 0 | 0 | 0.46 | 0.459 |
| PPARG | IFNG | 9606.ENSP00000287820 | 9606.ENSP00000229135 | 0 | 0 | 0 | 0 | 0 | 0 | 0 | 0.579 | 0.579 |
| PPARG | MAPK14 | 9606.ENSP00000287820 | 9606.ENSP00000229795 | 0 | 0 | 0 | 0 | 0 | 0.104 | 0 | 0.655 | 0.678 |
| PPARG | NR3C1 | 9606.ENSP00000287820 | 9606.ENSP00000231509 | 0 | 0 | 0 | 0.578 | 0.062 | 0.27 | 0 | 0.745 | 0.507 |
| PPARG | CAT | 9606.ENSP00000287820 | 9606.ENSP00000241052 | 0 | 0 | 0 | 0 | 0 | 0 | 0 | 0.655 | 0.655 |
| PPARG | AHR | 9606.ENSP00000287820 | 9606.ENSP00000242057 | 0 | 0 | 0 | 0 | 0 | 0.057 | 0 | 0.565 | 0.573 |
| PPARG | CDK4 | 9606.ENSP00000287820 | 9606.ENSP00000257904 | 0 | 0 | 0 | 0 | 0 | 0.057 | 0 | 0.414 | 0.423 |
| PPARG | MAPK3 | 9606.ENSP00000287820 | 9606.ENSP00000263025 | 0 | 0 | 0 | 0 | 0 | 0.104 | 0 | 0.627 | 0.651 |
| PPARG | IL1B | 9606.ENSP00000287820 | 9606.ENSP00000263341 | 0 | 0 | 0 | 0 | 0.062 | 0 | 0 | 0.739 | 0.745 |
| PPARG | KDR | 9606.ENSP00000287820 | 9606.ENSP00000263923 | 0 | 0 | 0 | 0 | 0.062 | 0.057 | 0 | 0.456 | 0.476 |
| PPARG | EGF | 9606.ENSP00000287820 | 9606.ENSP00000265171 | 0 | 0 | 0 | 0 | 0 | 0.056 | 0 | 0.522 | 0.529 |
| PPARG | CDK2 | 9606.ENSP00000287820 | 9606.ENSP00000266970 | 0 | 0 | 0 | 0 | 0 | 0.057 | 0 | 0.41 | 0.42 |
| PPARG | RB1 | 9606.ENSP00000287820 | 9606.ENSP00000267163 | 0 | 0 | 0 | 0 | 0 | 0.297 | 0.9 | 0.285 | 0.945 |
| PPARG | TP53 | 9606.ENSP00000287820 | 9606.ENSP00000269305 | 0 | 0 | 0 | 0 | 0 | 0.292 | 0.65 | 0.719 | 0.924 |
| PPARG | SOD1 | 9606.ENSP00000287820 | 9606.ENSP00000270142 | 0 | 0 | 0 | 0 | 0 | 0 | 0 | 0.459 | 0.459 |
| PPARG | EGFR | 9606.ENSP00000287820 | 9606.ENSP00000275493 | 0 | 0 | 0 | 0 | 0.062 | 0.316 | 0 | 0.517 | 0.663 |
| PPARG | HSPA5 | 9606.ENSP00000287820 | 9606.ENSP00000324173 | 0 | 0 | 0 | 0 | 0 | 0.066 | 0 | 0.393 | 0.409 |
| PPARG | AKR1C3 | 9606.ENSP00000287820 | 9606.ENSP00000369927 | 0 | 0 | 0 | 0 | 0 | 0.056 | 0 | 0.4 | 0.41 |
| PPARG | NQO1 | 9606.ENSP00000287820 | 9606.ENSP00000319788 | 0 | 0 | 0 | 0 | 0.062 | 0 | 0 | 0.406 | 0.419 |
| PPARG | XDH | 9606.ENSP00000287820 | 9606.ENSP00000368727 | 0 | 0 | 0 | 0 | 0.063 | 0.133 | 0 | 0.364 | 0.438 |
| PPARG | CYP3A4 | 9606.ENSP00000287820 | 9606.ENSP00000337915 | 0 | 0 | 0 | 0 | 0.053 | 0.073 | 0 | 0.413 | 0.439 |
| PPARG | ADRB2 | 9606.ENSP00000287820 | 9606.ENSP00000305372 | 0 | 0 | 0 | 0 | 0 | 0.056 | 0 | 0.457 | 0.465 |
| PPARG | SELE | 9606.ENSP00000287820 | 9606.ENSP00000331736 | 0 | 0 | 0 | 0 | 0.051 | 0.076 | 0 | 0.459 | 0.484 |
| PPARG | INSR | 9606.ENSP00000287820 | 9606.ENSP00000303830 | 0 | 0 | 0 | 0 | 0 | 0.077 | 0 | 0.507 | 0.525 |
| PPARG | PTGS1 | 9606.ENSP00000287820 | 9606.ENSP00000354612 | 0 | 0 | 0 | 0 | 0.062 | 0.056 | 0 | 0.507 | 0.525 |
| PPARG | NOS2 | 9606.ENSP00000287820 | 9606.ENSP00000327251 | 0 | 0 | 0 | 0 | 0.062 | 0.067 | 0 | 0.526 | 0.549 |
| PPARG | MAPK8 | 9606.ENSP00000287820 | 9606.ENSP00000378974 | 0 | 0 | 0 | 0 | 0 | 0.213 | 0 | 0.477 | 0.571 |
| PPARG | PTPN1 | 9606.ENSP00000287820 | 9606.ENSP00000360683 | 0 | 0 | 0 | 0 | 0 | 0.069 | 0 | 0.559 | 0.572 |
| PPARG | HMGCR | 9606.ENSP00000287820 | 9606.ENSP00000287936 | 0 | 0 | 0 | 0 | 0.062 | 0 | 0 | 0.568 | 0.577 |
| PPARG | DPP4 | 9606.ENSP00000287820 | 9606.ENSP00000353731 | 0 | 0 | 0 | 0 | 0.062 | 0.078 | 0 | 0.566 | 0.591 |
| PPARG | LDLR | 9606.ENSP00000287820 | 9606.ENSP00000454071 | 0 | 0 | 0 | 0 | 0 | 0.056 | 0 | 0.595 | 0.601 |
| PPARG | VCAM1 | 9606.ENSP00000287820 | 9606.ENSP00000294728 | 0 | 0 | 0 | 0 | 0.062 | 0 | 0 | 0.606 | 0.615 |
| PPARG | GSK3B | 9606.ENSP00000287820 | 9606.ENSP00000324806 | 0 | 0 | 0 | 0 | 0 | 0.077 | 0 | 0.612 | 0.626 |
| PPARG | VEGFA | 9606.ENSP00000287820 | 9606.ENSP00000478570 | 0 | 0 | 0 | 0 | 0 | 0 | 0 | 0.662 | 0.662 |
| PPARG | ALOX5 | 9606.ENSP00000287820 | 9606.ENSP00000363512 | 0 | 0 | 0 | 0 | 0 | 0 | 0 | 0.676 | 0.676 |
| PPARG | ACACA | 9606.ENSP00000287820 | 9606.ENSP00000483300 | 0 | 0 | 0 | 0 | 0.049 | 0 | 0 | 0.682 | 0.685 |
| PPARG | NOS3 | 9606.ENSP00000287820 | 9606.ENSP00000297494 | 0 | 0 | 0 | 0 | 0.052 | 0.067 | 0 | 0.673 | 0.686 |
| PPARG | IL6 | 9606.ENSP00000287820 | 9606.ENSP00000385675 | 0 | 0 | 0 | 0 | 0 | 0 | 0 | 0.792 | 0.792 |
| PPARG | FASN | 9606.ENSP00000287820 | 9606.ENSP00000304592 | 0 | 0 | 0 | 0 | 0 | 0 | 0 | 0.829 | 0.829 |
| PPARG | PTGS2 | 9606.ENSP00000287820 | 9606.ENSP00000356438 | 0 | 0 | 0 | 0 | 0 | 0.056 | 0 | 0.86 | 0.862 |
| PPARG | RXRB | 9606.ENSP00000287820 | 9606.ENSP00000363817 | 0 | 0 | 0 | 0.662 | 0.049 | 0.483 | 0.9 | 0.536 | 0.955 |
| PPARG | TNF | 9606.ENSP00000287820 | 9606.ENSP00000398698 | 0 | 0 | 0 | 0 | 0 | 0 | 0.9 | 0.807 | 0.979 |
| PPARG | JUN | 9606.ENSP00000287820 | 9606.ENSP00000360266 | 0 | 0 | 0 | 0 | 0 | 0.066 | 0.9 | 0.842 | 0.984 |
| PPARG | RXRA | 9606.ENSP00000287820 | 9606.ENSP00000419692 | 0 | 0 | 0 | 0.694 | 0.062 | 0.981 | 0.9 | 0.99 | 0.998 |
| PPARG | NCOA2 | 9606.ENSP00000287820 | 9606.ENSP00000399968 | 0 | 0 | 0 | 0 | 0.062 | 0.954 | 0.9 | 0.843 | 0.999 |
| PPARG | NCOA1 | 9606.ENSP00000287820 | 9606.ENSP00000385216 | 0 | 0 | 0 | 0 | 0 | 0.984 | 0.9 | 0.987 | 0.999 |
| PPP3CA | MAPK1 | 9606.ENSP00000378323 | 9606.ENSP00000215832 | 0 | 0 | 0 | 0 | 0.064 | 0.25 | 0 | 0.394 | 0.537 |
| PPP3CA | MAPK14 | 9606.ENSP00000378323 | 9606.ENSP00000229795 | 0 | 0 | 0 | 0 | 0.103 | 0.25 | 0 | 0.261 | 0.46 |
| PPP3CA | CDK4 | 9606.ENSP00000378323 | 9606.ENSP00000257904 | 0 | 0 | 0 | 0 | 0.063 | 0.149 | 0 | 0.374 | 0.457 |
| PPP3CA | MAPK3 | 9606.ENSP00000378323 | 9606.ENSP00000263025 | 0 | 0 | 0 | 0 | 0.064 | 0.25 | 0 | 0.295 | 0.462 |
| PPP3CA | SOD1 | 9606.ENSP00000378323 | 9606.ENSP00000270142 | 0 | 0 | 0 | 0 | 0 | 0.271 | 0.8 | 0.104 | 0.858 |
| PPP3CA | GSK3B | 9606.ENSP00000378323 | 9606.ENSP00000324806 | 0 | 0 | 0 | 0 | 0.083 | 0.244 | 0.8 | 0.379 | 0.902 |
| PPP3CA | JUN | 9606.ENSP00000378323 | 9606.ENSP00000360266 | 0 | 0 | 0 | 0 | 0 | 0.387 | 0 | 0.256 | 0.524 |
| PPP3CA | BAD | 9606.ENSP00000378323 | 9606.ENSP00000378040 | 0 | 0 | 0 | 0 | 0 | 0.213 | 0.8 | 0 | 0.835 |
| PPP3CA | MAPK8 | 9606.ENSP00000378323 | 9606.ENSP00000378974 | 0 | 0 | 0 | 0 | 0.052 | 0.064 | 0 | 0.418 | 0.438 |
| PPP3CA | BCL2 | 9606.ENSP00000378323 | 9606.ENSP00000381185 | 0 | 0 | 0 | 0 | 0 | 0.687 | 0 | 0.073 | 0.698 |
| PRKACA | MAPK1 | 9606.ENSP00000309591 | 9606.ENSP00000215832 | 0 | 0 | 0.357 | 0.611 | 0.065 | 0.18 | 0.8 | 0.257 | 0.867 |
| PRKACA | NR3C1 | 9606.ENSP00000309591 | 9606.ENSP00000231509 | 0 | 0 | 0 | 0 | 0.049 | 0.213 | 0.9 | 0.049 | 0.919 |
| PRKACA | MAPK3 | 9606.ENSP00000309591 | 9606.ENSP00000263025 | 0 | 0 | 0.369 | 0.611 | 0.106 | 0.18 | 0.8 | 0.189 | 0.87 |
| PRKACA | KDR | 9606.ENSP00000309591 | 9606.ENSP00000263923 | 0 | 0 | 0 | 0.578 | 0 | 0 | 0.9 | 0.101 | 0.902 |
| PRKACA | EGFR | 9606.ENSP00000309591 | 9606.ENSP00000275493 | 0 | 0 | 0 | 0.594 | 0 | 0.487 | 0 | 0.132 | 0.506 |
| PRKACA | KCNMA1 | 9606.ENSP00000309591 | 9606.ENSP00000286628 | 0 | 0 | 0 | 0 | 0.067 | 0.243 | 0.8 | 0.125 | 0.859 |
| PRKACA | BAX | 9606.ENSP00000309591 | 9606.ENSP00000293288 | 0 | 0 | 0 | 0 | 0.045 | 0 | 0.8 | 0 | 0.8 |
| PRKACA | NOS3 | 9606.ENSP00000309591 | 9606.ENSP00000297494 | 0 | 0 | 0 | 0 | 0 | 0.154 | 0.9 | 0.043 | 0.911 |
| PRKACA | MAP2 | 9606.ENSP00000309591 | 9606.ENSP00000353508 | 0 | 0 | 0 | 0 | 0 | 0.391 | 0 | 0.07 | 0.409 |
| PRKACA | PIM1 | 9606.ENSP00000309591 | 9606.ENSP00000362608 | 0 | 0 | 0.373 | 0.651 | 0.103 | 0.347 | 0 | 0.219 | 0.498 |
| PRKACA | PDE3A | 9606.ENSP00000309591 | 9606.ENSP00000351957 | 0 | 0 | 0 | 0 | 0.066 | 0.325 | 0.6 | 0.113 | 0.746 |
| PRKACA | ESR2 | 9606.ENSP00000309591 | 9606.ENSP00000343925 | 0 | 0 | 0 | 0 | 0 | 0 | 0.8 | 0.06 | 0.804 |
| PRKACA | JUN | 9606.ENSP00000309591 | 9606.ENSP00000360266 | 0 | 0 | 0 | 0 | 0 | 0 | 0.8 | 0.16 | 0.824 |
| PRKACA | ESR1 | 9606.ENSP00000309591 | 9606.ENSP00000405330 | 0 | 0 | 0 | 0 | 0 | 0.209 | 0.8 | 0.073 | 0.84 |
| PRKACA | PGR | 9606.ENSP00000309591 | 9606.ENSP00000325120 | 0 | 0 | 0 | 0 | 0 | 0 | 0.9 | 0 | 0.9 |
| PRKACA | VEGFA | 9606.ENSP00000309591 | 9606.ENSP00000478570 | 0 | 0 | 0 | 0 | 0 | 0 | 0.9 | 0.063 | 0.902 |
| PRKACA | AR | 9606.ENSP00000309591 | 9606.ENSP00000363822 | 0 | 0 | 0 | 0 | 0 | 0 | 0.9 | 0.094 | 0.905 |
| PRKACA | GSK3B | 9606.ENSP00000309591 | 9606.ENSP00000324806 | 0 | 0 | 0.251 | 0.625 | 0.079 | 0.721 | 0.8 | 0.276 | 0.953 |
| PRKACA | BAD | 9606.ENSP00000309591 | 9606.ENSP00000378040 | 0 | 0 | 0 | 0 | 0 | 0.729 | 0.9 | 0.232 | 0.977 |
| PRSS1 | MMP3 | 9606.ENSP00000308720 | 9606.ENSP00000299855 | 0 | 0 | 0 | 0 | 0 | 0 | 0.9 | 0.047 | 0.9 |
| PRSS1 | PRSS3 | 9606.ENSP00000308720 | 9606.ENSP00000354280 | 0 | 0 | 0.449 | 0.98 | 0.131 | 0 | 0.8 | 0.767 | 0.823 |
| PRSS1 | MMP1 | 9606.ENSP00000308720 | 9606.ENSP00000322788 | 0 | 0 | 0 | 0 | 0.066 | 0 | 0.9 | 0.086 | 0.907 |
| PRSS3 | PRSS1 | 9606.ENSP00000354280 | 9606.ENSP00000308720 | 0 | 0 | 0.449 | 0.98 | 0.131 | 0 | 0.8 | 0.767 | 0.823 |
| PRSS3 | HSPA5 | 9606.ENSP00000354280 | 9606.ENSP00000324173 | 0 | 0 | 0 | 0 | 0 | 0.058 | 0 | 0.435 | 0.445 |
| PTGER3 | IL1B | 9606.ENSP00000349003 | 9606.ENSP00000263341 | 0 | 0 | 0 | 0 | 0 | 0 | 0 | 0.468 | 0.468 |
| PTGER3 | ADRB2 | 9606.ENSP00000349003 | 9606.ENSP00000305372 | 0 | 0 | 0 | 0 | 0 | 0 | 0 | 0.475 | 0.475 |
| PTGER3 | VEGFA | 9606.ENSP00000349003 | 9606.ENSP00000478570 | 0 | 0 | 0 | 0 | 0 | 0 | 0 | 0.4 | 0.4 |
| PTGER3 | OPRM1 | 9606.ENSP00000349003 | 9606.ENSP00000394624 | 0 | 0 | 0 | 0 | 0 | 0 | 0 | 0.409 | 0.409 |
| PTGER3 | TNF | 9606.ENSP00000349003 | 9606.ENSP00000398698 | 0 | 0 | 0 | 0 | 0 | 0 | 0 | 0.428 | 0.428 |
| PTGER3 | LDLR | 9606.ENSP00000349003 | 9606.ENSP00000454071 | 0 | 0 | 0 | 0 | 0 | 0.056 | 0 | 0.435 | 0.443 |
| PTGER3 | IL6 | 9606.ENSP00000349003 | 9606.ENSP00000385675 | 0 | 0 | 0 | 0 | 0 | 0 | 0 | 0.462 | 0.462 |
| PTGER3 | ALOX5 | 9606.ENSP00000349003 | 9606.ENSP00000363512 | 0 | 0 | 0 | 0 | 0 | 0 | 0 | 0.47 | 0.47 |
| PTGER3 | PTGS1 | 9606.ENSP00000349003 | 9606.ENSP00000354612 | 0 | 0 | 0 | 0 | 0 | 0.056 | 0 | 0.618 | 0.624 |
| PTGER3 | PTGS2 | 9606.ENSP00000349003 | 9606.ENSP00000356438 | 0 | 0 | 0 | 0 | 0 | 0.056 | 0 | 0.71 | 0.714 |
| PTGS1 | MPO | 9606.ENSP00000354612 | 9606.ENSP00000225275 | 0 | 0 | 0 | 0 | 0.076 | 0 | 0.9 | 0.34 | 0.933 |
| PTGS1 | CCL2 | 9606.ENSP00000354612 | 9606.ENSP00000225831 | 0 | 0 | 0 | 0 | 0.097 | 0 | 0 | 0.389 | 0.424 |
| PTGS1 | LTA4H | 9606.ENSP00000354612 | 9606.ENSP00000228740 | 0 | 0 | 0 | 0 | 0.051 | 0 | 0 | 0.444 | 0.45 |
| PTGS1 | CAT | 9606.ENSP00000354612 | 9606.ENSP00000241052 | 0 | 0 | 0 | 0 | 0.065 | 0.178 | 0 | 0.342 | 0.45 |
| PTGS1 | IL1B | 9606.ENSP00000354612 | 9606.ENSP00000263341 | 0 | 0 | 0 | 0 | 0.157 | 0 | 0 | 0.605 | 0.654 |
| PTGS1 | EGF | 9606.ENSP00000354612 | 9606.ENSP00000265171 | 0 | 0 | 0 | 0 | 0.062 | 0.069 | 0 | 0.376 | 0.408 |
| PTGS1 | PPARG | 9606.ENSP00000354612 | 9606.ENSP00000287820 | 0 | 0 | 0 | 0 | 0.062 | 0.056 | 0 | 0.507 | 0.525 |
| PTGS1 | NOS3 | 9606.ENSP00000354612 | 9606.ENSP00000297494 | 0 | 0 | 0 | 0 | 0.048 | 0.142 | 0 | 0.501 | 0.556 |
| PTGS1 | NOS2 | 9606.ENSP00000354612 | 9606.ENSP00000327251 | 0 | 0 | 0 | 0 | 0.048 | 0.142 | 0 | 0.343 | 0.416 |
| PTGS1 | F3 | 9606.ENSP00000354612 | 9606.ENSP00000334145 | 0 | 0 | 0 | 0 | 0.062 | 0 | 0 | 0.39 | 0.403 |
| PTGS1 | PTGER3 | 9606.ENSP00000354612 | 9606.ENSP00000349003 | 0 | 0 | 0 | 0 | 0 | 0.056 | 0 | 0.618 | 0.624 |
| PTGS1 | VEGFA | 9606.ENSP00000354612 | 9606.ENSP00000478570 | 0 | 0 | 0 | 0 | 0.064 | 0 | 0 | 0.419 | 0.432 |
| PTGS1 | AKR1C3 | 9606.ENSP00000354612 | 9606.ENSP00000369927 | 0 | 0 | 0 | 0 | 0.061 | 0 | 0 | 0.471 | 0.482 |
| PTGS1 | IL6 | 9606.ENSP00000354612 | 9606.ENSP00000385675 | 0 | 0 | 0 | 0 | 0.096 | 0 | 0 | 0.54 | 0.566 |
| PTGS1 | TNF | 9606.ENSP00000354612 | 9606.ENSP00000398698 | 0 | 0 | 0 | 0 | 0.085 | 0 | 0 | 0.548 | 0.569 |
| PTGS1 | PTGS2 | 9606.ENSP00000354612 | 9606.ENSP00000356438 | 0 | 0 | 0.432 | 0.967 | 0.14 | 0.27 | 0.8 | 0.901 | 0.869 |
| PTGS1 | ALOX5 | 9606.ENSP00000354612 | 9606.ENSP00000363512 | 0 | 0 | 0 | 0 | 0.096 | 0 | 0.9 | 0.728 | 0.973 |
| PTGS2 | MAPK1 | 9606.ENSP00000356438 | 9606.ENSP00000215832 | 0 | 0 | 0 | 0 | 0 | 0.176 | 0 | 0.393 | 0.478 |
| PTGS2 | HMOX1 | 9606.ENSP00000356438 | 9606.ENSP00000216117 | 0 | 0 | 0 | 0 | 0.076 | 0 | 0 | 0.745 | 0.754 |
| PTGS2 | MMP2 | 9606.ENSP00000356438 | 9606.ENSP00000219070 | 0 | 0 | 0 | 0 | 0.066 | 0 | 0 | 0.654 | 0.663 |
| PTGS2 | GSR | 9606.ENSP00000356438 | 9606.ENSP00000221130 | 0 | 0 | 0 | 0 | 0 | 0.166 | 0 | 0.478 | 0.546 |
| PTGS2 | MPO | 9606.ENSP00000356438 | 9606.ENSP00000225275 | 0 | 0 | 0 | 0 | 0.065 | 0 | 0 | 0.644 | 0.653 |
| PTGS2 | CCL2 | 9606.ENSP00000356438 | 9606.ENSP00000225831 | 0 | 0 | 0 | 0 | 0.117 | 0 | 0 | 0.754 | 0.774 |
| PTGS2 | IL2 | 9606.ENSP00000356438 | 9606.ENSP00000226730 | 0 | 0 | 0 | 0 | 0 | 0 | 0 | 0.577 | 0.577 |
| PTGS2 | IFNG | 9606.ENSP00000356438 | 9606.ENSP00000229135 | 0 | 0 | 0 | 0 | 0 | 0 | 0 | 0.643 | 0.643 |
| PTGS2 | MAPK14 | 9606.ENSP00000356438 | 9606.ENSP00000229795 | 0 | 0 | 0 | 0 | 0 | 0.065 | 0 | 0.624 | 0.633 |
| PTGS2 | NR3C1 | 9606.ENSP00000356438 | 9606.ENSP00000231509 | 0 | 0 | 0 | 0 | 0.069 | 0.056 | 0 | 0.465 | 0.488 |
| PTGS2 | ODC1 | 9606.ENSP00000356438 | 9606.ENSP00000234111 | 0 | 0 | 0 | 0 | 0 | 0 | 0 | 0.468 | 0.468 |
| PTGS2 | CAT | 9606.ENSP00000356438 | 9606.ENSP00000241052 | 0 | 0 | 0 | 0 | 0 | 0.178 | 0 | 0.675 | 0.721 |
| PTGS2 | AHR | 9606.ENSP00000356438 | 9606.ENSP00000242057 | 0 | 0 | 0 | 0 | 0.098 | 0 | 0 | 0.469 | 0.5 |
| PTGS2 | MAPK3 | 9606.ENSP00000356438 | 9606.ENSP00000263025 | 0 | 0 | 0 | 0 | 0 | 0.334 | 0 | 0.682 | 0.78 |
| PTGS2 | IL1B | 9606.ENSP00000356438 | 9606.ENSP00000263341 | 0 | 0 | 0 | 0 | 0.561 | 0 | 0 | 0.877 | 0.944 |
| PTGS2 | KDR | 9606.ENSP00000356438 | 9606.ENSP00000263923 | 0 | 0 | 0 | 0 | 0 | 0 | 0 | 0.477 | 0.477 |
| PTGS2 | EGF | 9606.ENSP00000356438 | 9606.ENSP00000265171 | 0 | 0 | 0 | 0 | 0.062 | 0.069 | 0 | 0.623 | 0.642 |
| PTGS2 | CDK2 | 9606.ENSP00000356438 | 9606.ENSP00000266970 | 0 | 0 | 0 | 0 | 0 | 0 | 0 | 0.403 | 0.403 |
| PTGS2 | TP53 | 9606.ENSP00000356438 | 9606.ENSP00000269305 | 0 | 0 | 0 | 0 | 0 | 0.874 | 0 | 0.958 | 0.994 |
| PTGS2 | SOD1 | 9606.ENSP00000356438 | 9606.ENSP00000270142 | 0 | 0 | 0 | 0 | 0.062 | 0 | 0 | 0.457 | 0.468 |
| PTGS2 | EGFR | 9606.ENSP00000356438 | 9606.ENSP00000275493 | 0 | 0 | 0 | 0 | 0.062 | 0 | 0 | 0.7 | 0.706 |
| PTGS2 | GJA1 | 9606.ENSP00000356438 | 9606.ENSP00000282561 | 0 | 0 | 0 | 0 | 0 | 0 | 0 | 0.459 | 0.459 |
| PTGS2 | PPARG | 9606.ENSP00000356438 | 9606.ENSP00000287820 | 0 | 0 | 0 | 0 | 0 | 0.056 | 0 | 0.86 | 0.862 |
| PTGS2 | BAX | 9606.ENSP00000356438 | 9606.ENSP00000293288 | 0 | 0 | 0 | 0 | 0 | 0 | 0 | 0.458 | 0.457 |
| PTGS2 | VCAM1 | 9606.ENSP00000356438 | 9606.ENSP00000294728 | 0 | 0 | 0 | 0 | 0.062 | 0 | 0 | 0.66 | 0.667 |
| PTGS2 | NOS3 | 9606.ENSP00000356438 | 9606.ENSP00000297494 | 0 | 0 | 0 | 0 | 0.048 | 0.159 | 0 | 0.662 | 0.705 |
| PTGS2 | MMP3 | 9606.ENSP00000356438 | 9606.ENSP00000299855 | 0 | 0 | 0 | 0 | 0.095 | 0 | 0 | 0.672 | 0.691 |
| PTGS2 | ACHE | 9606.ENSP00000356438 | 9606.ENSP00000303211 | 0 | 0 | 0 | 0 | 0 | 0.059 | 0 | 0.467 | 0.477 |
| PTGS2 | PPARD | 9606.ENSP00000356438 | 9606.ENSP00000310928 | 0 | 0 | 0 | 0 | 0 | 0.056 | 0 | 0.442 | 0.451 |
| PTGS2 | NQO1 | 9606.ENSP00000356438 | 9606.ENSP00000319788 | 0 | 0 | 0 | 0 | 0 | 0 | 0 | 0.506 | 0.506 |
| PTGS2 | MMP1 | 9606.ENSP00000356438 | 9606.ENSP00000322788 | 0 | 0 | 0 | 0 | 0.138 | 0 | 0 | 0.618 | 0.657 |
| PTGS2 | GSK3B | 9606.ENSP00000356438 | 9606.ENSP00000324806 | 0 | 0 | 0 | 0 | 0 | 0.077 | 0 | 0.477 | 0.497 |
| PTGS2 | PGR | 9606.ENSP00000356438 | 9606.ENSP00000325120 | 0 | 0 | 0 | 0 | 0.062 | 0.056 | 0 | 0.57 | 0.586 |
| PTGS2 | NOS2 | 9606.ENSP00000356438 | 9606.ENSP00000327251 | 0 | 0 | 0 | 0 | 0.063 | 0.182 | 0 | 0.805 | 0.838 |
| PTGS2 | SELE | 9606.ENSP00000356438 | 9606.ENSP00000331736 | 0 | 0 | 0 | 0 | 0.065 | 0.056 | 0 | 0.531 | 0.55 |
| PTGS2 | F3 | 9606.ENSP00000356438 | 9606.ENSP00000334145 | 0 | 0 | 0 | 0 | 0.085 | 0 | 0 | 0.463 | 0.487 |
| PTGS2 | ESR2 | 9606.ENSP00000356438 | 9606.ENSP00000343925 | 0 | 0 | 0 | 0 | 0 | 0.056 | 0 | 0.52 | 0.527 |
| PTGS2 | PTGER3 | 9606.ENSP00000356438 | 9606.ENSP00000349003 | 0 | 0 | 0 | 0 | 0 | 0.056 | 0 | 0.71 | 0.714 |
| PTGS2 | PTGS1 | 9606.ENSP00000356438 | 9606.ENSP00000354612 | 0 | 0 | 0.432 | 0.967 | 0.14 | 0.27 | 0.8 | 0.901 | 0.869 |
| PTGS2 | AR | 9606.ENSP00000356438 | 9606.ENSP00000363822 | 0 | 0 | 0 | 0 | 0 | 0.056 | 0 | 0.412 | 0.421 |
| PTGS2 | GSTP1 | 9606.ENSP00000356438 | 9606.ENSP00000381607 | 0 | 0 | 0 | 0 | 0 | 0 | 0 | 0.501 | 0.501 |
| PTGS2 | PLAU | 9606.ENSP00000356438 | 9606.ENSP00000361850 | 0 | 0 | 0 | 0 | 0.103 | 0.056 | 0 | 0.469 | 0.511 |
| PTGS2 | AKR1C3 | 9606.ENSP00000356438 | 9606.ENSP00000369927 | 0 | 0 | 0 | 0 | 0 | 0 | 0 | 0.548 | 0.548 |
| PTGS2 | MAPK8 | 9606.ENSP00000356438 | 9606.ENSP00000378974 | 0 | 0 | 0 | 0 | 0 | 0 | 0 | 0.564 | 0.564 |
| PTGS2 | ESR1 | 9606.ENSP00000356438 | 9606.ENSP00000405330 | 0 | 0 | 0 | 0 | 0 | 0.056 | 0 | 0.632 | 0.637 |
| PTGS2 | JUN | 9606.ENSP00000356438 | 9606.ENSP00000360266 | 0 | 0 | 0 | 0 | 0.066 | 0 | 0 | 0.738 | 0.745 |
| PTGS2 | TNF | 9606.ENSP00000356438 | 9606.ENSP00000398698 | 0 | 0 | 0 | 0 | 0.096 | 0 | 0 | 0.855 | 0.863 |
| PTGS2 | VEGFA | 9606.ENSP00000356438 | 9606.ENSP00000478570 | 0 | 0 | 0 | 0 | 0.083 | 0 | 0 | 0.892 | 0.896 |
| PTGS2 | IL6 | 9606.ENSP00000356438 | 9606.ENSP00000385675 | 0 | 0 | 0 | 0 | 0.312 | 0 | 0 | 0.878 | 0.913 |
| PTGS2 | ALOX5 | 9606.ENSP00000356438 | 9606.ENSP00000363512 | 0 | 0 | 0 | 0 | 0.081 | 0 | 0.9 | 0.828 | 0.982 |
| PTPN1 | MAPK1 | 9606.ENSP00000360683 | 9606.ENSP00000215832 | 0 | 0 | 0 | 0 | 0.092 | 0.382 | 0 | 0.326 | 0.589 |
| PTPN1 | IFNG | 9606.ENSP00000360683 | 9606.ENSP00000229135 | 0 | 0 | 0 | 0 | 0 | 0 | 0.9 | 0.177 | 0.914 |
| PTPN1 | MAPK14 | 9606.ENSP00000360683 | 9606.ENSP00000229795 | 0 | 0 | 0 | 0 | 0.065 | 0.247 | 0 | 0.341 | 0.495 |
| PTPN1 | CAT | 9606.ENSP00000360683 | 9606.ENSP00000241052 | 0 | 0 | 0 | 0 | 0.053 | 0 | 0 | 0.468 | 0.475 |
| PTPN1 | MAPK3 | 9606.ENSP00000360683 | 9606.ENSP00000263025 | 0 | 0 | 0 | 0 | 0.062 | 0.247 | 0 | 0.527 | 0.637 |
| PTPN1 | IL1B | 9606.ENSP00000360683 | 9606.ENSP00000263341 | 0 | 0 | 0 | 0 | 0 | 0.27 | 0 | 0.362 | 0.514 |
| PTPN1 | KDR | 9606.ENSP00000360683 | 9606.ENSP00000263923 | 0 | 0 | 0 | 0 | 0 | 0.06 | 0 | 0.847 | 0.85 |
| PTPN1 | EGF | 9606.ENSP00000360683 | 9606.ENSP00000265171 | 0 | 0 | 0 | 0 | 0 | 0.06 | 0.9 | 0.576 | 0.956 |
| PTPN1 | TP53 | 9606.ENSP00000360683 | 9606.ENSP00000269305 | 0 | 0 | 0 | 0 | 0.054 | 0 | 0 | 0.405 | 0.413 |
| PTPN1 | EGFR | 9606.ENSP00000360683 | 9606.ENSP00000275493 | 0 | 0 | 0 | 0 | 0 | 0.893 | 0.9 | 0.988 | 0.999 |
| PTPN1 | PPARG | 9606.ENSP00000360683 | 9606.ENSP00000287820 | 0 | 0 | 0 | 0 | 0 | 0.069 | 0 | 0.559 | 0.572 |
| PTPN1 | NOS3 | 9606.ENSP00000360683 | 9606.ENSP00000297494 | 0 | 0 | 0 | 0 | 0.048 | 0.066 | 0 | 0.425 | 0.444 |
| PTPN1 | INSR | 9606.ENSP00000360683 | 9606.ENSP00000303830 | 0 | 0 | 0 | 0 | 0.062 | 0.985 | 0.9 | 0.547 | 0.999 |
| PTPN1 | HSPA5 | 9606.ENSP00000360683 | 9606.ENSP00000324173 | 0 | 0 | 0 | 0 | 0.062 | 0 | 0 | 0.447 | 0.459 |
| PTPN1 | GSK3B | 9606.ENSP00000360683 | 9606.ENSP00000324806 | 0 | 0 | 0 | 0 | 0.062 | 0.298 | 0 | 0.33 | 0.521 |
| PTPN1 | DPP4 | 9606.ENSP00000360683 | 9606.ENSP00000353731 | 0 | 0 | 0 | 0 | 0 | 0 | 0 | 0.472 | 0.472 |
| PTPN1 | JUN | 9606.ENSP00000360683 | 9606.ENSP00000360266 | 0 | 0 | 0 | 0 | 0 | 0.129 | 0 | 0.422 | 0.475 |
| PTPN1 | IL6 | 9606.ENSP00000360683 | 9606.ENSP00000385675 | 0 | 0 | 0 | 0 | 0.062 | 0 | 0 | 0.431 | 0.443 |
| PTPN1 | TNF | 9606.ENSP00000360683 | 9606.ENSP00000398698 | 0 | 0 | 0 | 0 | 0 | 0.057 | 0 | 0.447 | 0.456 |
| PTPN1 | CDK1 | 9606.ENSP00000360683 | 9606.ENSP00000378699 | 0 | 0 | 0 | 0 | 0.062 | 0.3 | 0 | 0.248 | 0.464 |
| PTPN1 | ESR1 | 9606.ENSP00000360683 | 9606.ENSP00000405330 | 0 | 0 | 0 | 0 | 0 | 0.291 | 0 | 0.3 | 0.482 |
| PTPN1 | VEGFA | 9606.ENSP00000360683 | 9606.ENSP00000478570 | 0 | 0 | 0 | 0 | 0 | 0 | 0 | 0.539 | 0.539 |
| RB1 | MAPK1 | 9606.ENSP00000267163 | 9606.ENSP00000215832 | 0 | 0 | 0 | 0 | 0.062 | 0.213 | 0.9 | 0.296 | 0.941 |
| RB1 | MAPK14 | 9606.ENSP00000267163 | 9606.ENSP00000229795 | 0 | 0 | 0 | 0 | 0.062 | 0.839 | 0.9 | 0.208 | 0.986 |
| RB1 | NR3C1 | 9606.ENSP00000267163 | 9606.ENSP00000231509 | 0 | 0 | 0 | 0 | 0 | 0.077 | 0 | 0.387 | 0.41 |
| RB1 | AHR | 9606.ENSP00000267163 | 9606.ENSP00000242057 | 0 | 0 | 0 | 0 | 0 | 0.857 | 0 | 0.134 | 0.871 |
| RB1 | CDK4 | 9606.ENSP00000267163 | 9606.ENSP00000257904 | 0 | 0 | 0 | 0 | 0.064 | 0.993 | 0.9 | 0.924 | 0.999 |
| RB1 | MAPK3 | 9606.ENSP00000267163 | 9606.ENSP00000263025 | 0 | 0 | 0 | 0 | 0.062 | 0.156 | 0.9 | 0.404 | 0.946 |
| RB1 | CDK2 | 9606.ENSP00000267163 | 9606.ENSP00000266970 | 0 | 0 | 0 | 0 | 0.082 | 0.929 | 0.9 | 0.71 | 0.997 |
| RB1 | TOP2A | 9606.ENSP00000267163 | 9606.ENSP00000411532 | 0 | 0 | 0 | 0 | 0.122 | 0.27 | 0 | 0.141 | 0.401 |
| RB1 | MAPK8 | 9606.ENSP00000267163 | 9606.ENSP00000378974 | 0 | 0 | 0 | 0 | 0 | 0.308 | 0 | 0.198 | 0.422 |
| RB1 | EGFR | 9606.ENSP00000267163 | 9606.ENSP00000275493 | 0 | 0 | 0 | 0 | 0 | 0.056 | 0 | 0.513 | 0.52 |
| RB1 | ESR2 | 9606.ENSP00000267163 | 9606.ENSP00000343925 | 0 | 0 | 0 | 0 | 0 | 0.077 | 0 | 0.519 | 0.537 |
| RB1 | CHEK1 | 9606.ENSP00000267163 | 9606.ENSP00000388648 | 0 | 0 | 0 | 0 | 0.062 | 0.27 | 0 | 0.402 | 0.554 |
| RB1 | PGR | 9606.ENSP00000267163 | 9606.ENSP00000325120 | 0 | 0 | 0 | 0 | 0 | 0.077 | 0 | 0.688 | 0.7 |
| RB1 | ESR1 | 9606.ENSP00000267163 | 9606.ENSP00000405330 | 0 | 0 | 0 | 0 | 0 | 0.077 | 0 | 0.782 | 0.79 |
| RB1 | NCOA1 | 9606.ENSP00000267163 | 9606.ENSP00000385216 | 0 | 0 | 0 | 0 | 0 | 0.105 | 0 | 0.851 | 0.861 |
| RB1 | AR | 9606.ENSP00000267163 | 9606.ENSP00000363822 | 0 | 0 | 0 | 0 | 0 | 0.687 | 0.65 | 0.489 | 0.939 |
| RB1 | PPARG | 9606.ENSP00000267163 | 9606.ENSP00000287820 | 0 | 0 | 0 | 0 | 0 | 0.297 | 0.9 | 0.285 | 0.945 |
| RB1 | JUN | 9606.ENSP00000267163 | 9606.ENSP00000360266 | 0 | 0 | 0 | 0 | 0 | 0.486 | 0.9 | 0.421 | 0.967 |
| RB1 | TP53 | 9606.ENSP00000267163 | 9606.ENSP00000269305 | 0 | 0 | 0 | 0 | 0.056 | 0.486 | 0.9 | 0.823 | 0.99 |
| RB1 | CCNA2 | 9606.ENSP00000267163 | 9606.ENSP00000274026 | 0 | 0 | 0 | 0 | 0.108 | 0.885 | 0.9 | 0.733 | 0.996 |
| RB1 | CDK1 | 9606.ENSP00000267163 | 9606.ENSP00000378699 | 0 | 0 | 0 | 0 | 0.064 | 0.895 | 0.9 | 0.709 | 0.996 |
| RXRA | MAPK1 | 9606.ENSP00000419692 | 9606.ENSP00000215832 | 0 | 0 | 0 | 0 | 0 | 0.498 | 0.9 | 0.134 | 0.952 |
| RXRA | NR3C1 | 9606.ENSP00000419692 | 9606.ENSP00000231509 | 0 | 0 | 0 | 0.632 | 0 | 0 | 0.9 | 0.643 | 0.923 |
| RXRA | MAPK3 | 9606.ENSP00000419692 | 9606.ENSP00000263025 | 0 | 0 | 0 | 0 | 0 | 0.317 | 0.9 | 0.152 | 0.937 |
| RXRA | PPARG | 9606.ENSP00000419692 | 9606.ENSP00000287820 | 0 | 0 | 0 | 0.694 | 0.062 | 0.981 | 0.9 | 0.99 | 0.998 |
| RXRA | HMGCR | 9606.ENSP00000419692 | 9606.ENSP00000287936 | 0 | 0 | 0 | 0 | 0.062 | 0 | 0.9 | 0.313 | 0.929 |
| RXRA | FASN | 9606.ENSP00000419692 | 9606.ENSP00000304592 | 0 | 0 | 0 | 0 | 0.062 | 0 | 0.9 | 0.331 | 0.931 |
| RXRA | PPARD | 9606.ENSP00000419692 | 9606.ENSP00000310928 | 0 | 0 | 0 | 0.705 | 0.062 | 0.684 | 0.9 | 0.852 | 0.975 |
| RXRA | NOS2 | 9606.ENSP00000419692 | 9606.ENSP00000327251 | 0 | 0 | 0 | 0 | 0 | 0.067 | 0.9 | 0.047 | 0.903 |
| RXRA | CYP3A4 | 9606.ENSP00000419692 | 9606.ENSP00000337915 | 0 | 0 | 0 | 0 | 0 | 0.073 | 0 | 0.42 | 0.439 |
| RXRA | RXRB | 9606.ENSP00000419692 | 9606.ENSP00000363817 | 0 | 0 | 0 | 0.968 | 0.07 | 0 | 0.9 | 0.838 | 0.905 |
| RXRA | CDK1 | 9606.ENSP00000419692 | 9606.ENSP00000378699 | 0 | 0 | 0 | 0 | 0 | 0.057 | 0.9 | 0.099 | 0.907 |
| RXRA | MAPK8 | 9606.ENSP00000419692 | 9606.ENSP00000378974 | 0 | 0 | 0 | 0 | 0 | 0 | 0.9 | 0.143 | 0.91 |
| RXRA | BCL2 | 9606.ENSP00000419692 | 9606.ENSP00000381185 | 0 | 0 | 0 | 0 | 0 | 0.057 | 0.9 | 0 | 0.901 |
| RXRA | NCOA1 | 9606.ENSP00000419692 | 9606.ENSP00000385216 | 0 | 0 | 0 | 0 | 0 | 0.988 | 0.9 | 0.626 | 0.999 |
| RXRA | TNF | 9606.ENSP00000419692 | 9606.ENSP00000398698 | 0 | 0 | 0 | 0 | 0 | 0 | 0.9 | 0.328 | 0.93 |
| RXRA | NCOA2 | 9606.ENSP00000419692 | 9606.ENSP00000399968 | 0 | 0 | 0 | 0 | 0.062 | 0.983 | 0.9 | 0.505 | 0.999 |
| RXRB | MAPK1 | 9606.ENSP00000363817 | 9606.ENSP00000215832 | 0 | 0 | 0 | 0 | 0 | 0.104 | 0.9 | 0.05 | 0.907 |
| RXRB | MAPK3 | 9606.ENSP00000363817 | 9606.ENSP00000263025 | 0 | 0 | 0 | 0 | 0 | 0.104 | 0.9 | 0.05 | 0.907 |
| RXRB | PPARG | 9606.ENSP00000363817 | 9606.ENSP00000287820 | 0 | 0 | 0 | 0.662 | 0.049 | 0.483 | 0.9 | 0.536 | 0.955 |
| RXRB | FASN | 9606.ENSP00000363817 | 9606.ENSP00000304592 | 0 | 0 | 0 | 0 | 0.065 | 0 | 0.9 | 0.124 | 0.91 |
| RXRB | PPARD | 9606.ENSP00000363817 | 9606.ENSP00000310928 | 0 | 0 | 0 | 0.712 | 0.072 | 0.417 | 0.9 | 0.749 | 0.953 |
| RXRB | NCOA1 | 9606.ENSP00000363817 | 9606.ENSP00000385216 | 0 | 0 | 0 | 0 | 0 | 0.417 | 0.6 | 0.305 | 0.824 |
| RXRB | BCL2 | 9606.ENSP00000363817 | 9606.ENSP00000381185 | 0 | 0 | 0 | 0 | 0.062 | 0.057 | 0.9 | 0 | 0.903 |
| RXRB | RXRA | 9606.ENSP00000363817 | 9606.ENSP00000419692 | 0 | 0 | 0 | 0.968 | 0.07 | 0 | 0.9 | 0.838 | 0.905 |
| RXRB | NCOA2 | 9606.ENSP00000363817 | 9606.ENSP00000399968 | 0 | 0 | 0 | 0 | 0 | 0.705 | 0.6 | 0.509 | 0.937 |
| SELE | HMOX1 | 9606.ENSP00000331736 | 9606.ENSP00000216117 | 0 | 0 | 0 | 0 | 0 | 0 | 0 | 0.509 | 0.509 |
| SELE | MMP2 | 9606.ENSP00000331736 | 9606.ENSP00000219070 | 0 | 0 | 0 | 0 | 0 | 0 | 0 | 0.504 | 0.504 |
| SELE | PLAT | 9606.ENSP00000331736 | 9606.ENSP00000220809 | 0 | 0 | 0 | 0 | 0 | 0 | 0 | 0.506 | 0.506 |
| SELE | MPO | 9606.ENSP00000331736 | 9606.ENSP00000225275 | 0 | 0 | 0 | 0 | 0 | 0.056 | 0 | 0.636 | 0.641 |
| SELE | CCL2 | 9606.ENSP00000331736 | 9606.ENSP00000225831 | 0 | 0 | 0 | 0 | 0.109 | 0 | 0 | 0.767 | 0.784 |
| SELE | IL2 | 9606.ENSP00000331736 | 9606.ENSP00000226730 | 0 | 0 | 0 | 0 | 0 | 0 | 0 | 0.533 | 0.533 |
| SELE | IFNG | 9606.ENSP00000331736 | 9606.ENSP00000229135 | 0 | 0 | 0 | 0 | 0 | 0 | 0 | 0.588 | 0.588 |
| SELE | CAT | 9606.ENSP00000331736 | 9606.ENSP00000241052 | 0 | 0 | 0 | 0 | 0.049 | 0 | 0 | 0.41 | 0.415 |
| SELE | MAPK3 | 9606.ENSP00000331736 | 9606.ENSP00000263025 | 0 | 0 | 0 | 0 | 0 | 0 | 0 | 0.401 | 0.401 |
| SELE | IL1B | 9606.ENSP00000331736 | 9606.ENSP00000263341 | 0 | 0 | 0 | 0 | 0.062 | 0 | 0 | 0.753 | 0.758 |
| SELE | KDR | 9606.ENSP00000331736 | 9606.ENSP00000263923 | 0 | 0 | 0 | 0 | 0.128 | 0.076 | 0 | 0.601 | 0.65 |
| SELE | EGF | 9606.ENSP00000331736 | 9606.ENSP00000265171 | 0 | 0 | 0 | 0 | 0 | 0 | 0 | 0.505 | 0.505 |
| SELE | EGFR | 9606.ENSP00000331736 | 9606.ENSP00000275493 | 0 | 0 | 0 | 0 | 0 | 0 | 0 | 0.425 | 0.425 |
| SELE | PPARG | 9606.ENSP00000331736 | 9606.ENSP00000287820 | 0 | 0 | 0 | 0 | 0.051 | 0.076 | 0 | 0.459 | 0.484 |
| SELE | VCAM1 | 9606.ENSP00000331736 | 9606.ENSP00000294728 | 0 | 0 | 0 | 0 | 0.122 | 0 | 0 | 0.916 | 0.924 |
| SELE | NOS3 | 9606.ENSP00000331736 | 9606.ENSP00000297494 | 0 | 0 | 0 | 0 | 0.079 | 0 | 0 | 0.681 | 0.693 |
| SELE | MMP3 | 9606.ENSP00000331736 | 9606.ENSP00000299855 | 0 | 0 | 0 | 0 | 0 | 0 | 0 | 0.481 | 0.481 |
| SELE | F2 | 9606.ENSP00000331736 | 9606.ENSP00000308541 | 0 | 0 | 0 | 0 | 0.049 | 0 | 0 | 0.553 | 0.556 |
| SELE | MMP1 | 9606.ENSP00000331736 | 9606.ENSP00000322788 | 0 | 0 | 0 | 0 | 0.052 | 0 | 0 | 0.46 | 0.466 |
| SELE | NOS2 | 9606.ENSP00000331736 | 9606.ENSP00000327251 | 0 | 0 | 0 | 0 | 0.063 | 0 | 0 | 0.405 | 0.419 |
| SELE | JUN | 9606.ENSP00000331736 | 9606.ENSP00000360266 | 0 | 0 | 0 | 0 | 0 | 0 | 0 | 0.463 | 0.463 |
| SELE | PTGS2 | 9606.ENSP00000331736 | 9606.ENSP00000356438 | 0 | 0 | 0 | 0 | 0.065 | 0.056 | 0 | 0.531 | 0.55 |
| SELE | VEGFA | 9606.ENSP00000331736 | 9606.ENSP00000478570 | 0 | 0 | 0 | 0 | 0 | 0 | 0 | 0.685 | 0.685 |
| SELE | THBD | 9606.ENSP00000331736 | 9606.ENSP00000366307 | 0 | 0 | 0 | 0 | 0.076 | 0 | 0 | 0.702 | 0.712 |
| SELE | F3 | 9606.ENSP00000331736 | 9606.ENSP00000334145 | 0 | 0 | 0 | 0 | 0 | 0 | 0 | 0.715 | 0.715 |
| SELE | IL6 | 9606.ENSP00000331736 | 9606.ENSP00000385675 | 0 | 0 | 0 | 0 | 0.121 | 0 | 0 | 0.803 | 0.819 |
| SELE | TNF | 9606.ENSP00000331736 | 9606.ENSP00000398698 | 0 | 0 | 0 | 0 | 0.06 | 0 | 0 | 0.835 | 0.838 |
| SLC6A3 | NR3C1 | 9606.ENSP00000270349 | 9606.ENSP00000231509 | 0 | 0 | 0 | 0 | 0 | 0.057 | 0 | 0.391 | 0.401 |
| SLC6A3 | OPRD1 | 9606.ENSP00000270349 | 9606.ENSP00000234961 | 0 | 0 | 0 | 0 | 0 | 0.057 | 0 | 0.392 | 0.402 |
| SLC6A3 | HTR3A | 9606.ENSP00000270349 | 9606.ENSP00000347754 | 0 | 0 | 0 | 0 | 0 | 0 | 0 | 0.405 | 0.405 |
| SLC6A3 | ADRA1B | 9606.ENSP00000270349 | 9606.ENSP00000306662 | 0 | 0 | 0 | 0 | 0 | 0.057 | 0 | 0.41 | 0.42 |
| SLC6A3 | CHRNA7 | 9606.ENSP00000270349 | 9606.ENSP00000407546 | 0 | 0 | 0 | 0 | 0 | 0 | 0 | 0.427 | 0.427 |
| SLC6A3 | ACHE | 9606.ENSP00000270349 | 9606.ENSP00000303211 | 0 | 0 | 0 | 0 | 0.062 | 0 | 0 | 0.442 | 0.454 |
| SLC6A3 | ADRA1A | 9606.ENSP00000270349 | 9606.ENSP00000369960 | 0 | 0 | 0 | 0 | 0 | 0.057 | 0 | 0.457 | 0.466 |
| SLC6A3 | ESR1 | 9606.ENSP00000270349 | 9606.ENSP00000405330 | 0 | 0 | 0 | 0 | 0.053 | 0.057 | 0 | 0.453 | 0.469 |
| SLC6A3 | ADRA1D | 9606.ENSP00000270349 | 9606.ENSP00000368766 | 0 | 0 | 0 | 0 | 0 | 0.057 | 0 | 0.469 | 0.478 |
| SLC6A3 | ADRA2A | 9606.ENSP00000270349 | 9606.ENSP00000280155 | 0 | 0 | 0 | 0 | 0 | 0.057 | 0 | 0.556 | 0.563 |
| SLC6A3 | OPRM1 | 9606.ENSP00000270349 | 9606.ENSP00000394624 | 0 | 0 | 0 | 0 | 0 | 0.057 | 0 | 0.681 | 0.686 |
| SLC6A3 | HTR2A | 9606.ENSP00000270349 | 9606.ENSP00000437737 | 0 | 0 | 0 | 0 | 0 | 0.057 | 0 | 0.713 | 0.718 |
| SLC6A3 | MAOB | 9606.ENSP00000270349 | 9606.ENSP00000367309 | 0 | 0 | 0 | 0 | 0 | 0 | 0 | 0.784 | 0.785 |
| SLC6A3 | MAOA | 9606.ENSP00000270349 | 9606.ENSP00000340684 | 0 | 0 | 0 | 0 | 0 | 0 | 0 | 0.804 | 0.804 |
| SLC6A3 | DRD1 | 9606.ENSP00000270349 | 9606.ENSP00000377353 | 0 | 0 | 0 | 0 | 0.057 | 0.057 | 0 | 0.823 | 0.829 |
| SLC6A4 | NR3C1 | 9606.ENSP00000261707 | 9606.ENSP00000231509 | 0 | 0 | 0 | 0 | 0 | 0.057 | 0 | 0.681 | 0.686 |
| SLC6A4 | OPRD1 | 9606.ENSP00000261707 | 9606.ENSP00000234961 | 0 | 0 | 0 | 0 | 0 | 0.057 | 0 | 0.501 | 0.509 |
| SLC6A4 | NR3C2 | 9606.ENSP00000261707 | 9606.ENSP00000350815 | 0 | 0 | 0 | 0 | 0 | 0.057 | 0 | 0.404 | 0.414 |
| SLC6A4 | ESR1 | 9606.ENSP00000261707 | 9606.ENSP00000405330 | 0 | 0 | 0 | 0 | 0.062 | 0.057 | 0 | 0.391 | 0.414 |
| SLC6A4 | ACHE | 9606.ENSP00000261707 | 9606.ENSP00000303211 | 0 | 0 | 0 | 0 | 0 | 0 | 0 | 0.417 | 0.417 |
| SLC6A4 | ADH1B | 9606.ENSP00000261707 | 9606.ENSP00000306606 | 0 | 0 | 0 | 0 | 0 | 0 | 0 | 0.423 | 0.422 |
| SLC6A4 | IL1B | 9606.ENSP00000261707 | 9606.ENSP00000263341 | 0 | 0 | 0 | 0 | 0 | 0 | 0 | 0.43 | 0.43 |
| SLC6A4 | ADRA1B | 9606.ENSP00000261707 | 9606.ENSP00000306662 | 0 | 0 | 0 | 0 | 0.062 | 0.057 | 0 | 0.429 | 0.451 |
| SLC6A4 | CYP1A2 | 9606.ENSP00000261707 | 9606.ENSP00000342007 | 0 | 0 | 0 | 0 | 0 | 0 | 0 | 0.46 | 0.459 |
| SLC6A4 | CHRNA7 | 9606.ENSP00000261707 | 9606.ENSP00000407546 | 0 | 0 | 0 | 0 | 0 | 0 | 0 | 0.461 | 0.461 |
| SLC6A4 | ADRB2 | 9606.ENSP00000261707 | 9606.ENSP00000305372 | 0 | 0 | 0 | 0 | 0 | 0.057 | 0 | 0.456 | 0.465 |
| SLC6A4 | ADRA1A | 9606.ENSP00000261707 | 9606.ENSP00000369960 | 0 | 0 | 0 | 0 | 0 | 0.057 | 0 | 0.471 | 0.48 |
| SLC6A4 | CHRM2 | 9606.ENSP00000261707 | 9606.ENSP00000399745 | 0 | 0 | 0 | 0 | 0.062 | 0 | 0 | 0.471 | 0.483 |
| SLC6A4 | ADRA1D | 9606.ENSP00000261707 | 9606.ENSP00000368766 | 0 | 0 | 0 | 0 | 0 | 0.057 | 0 | 0.477 | 0.485 |
| SLC6A4 | ADRA2A | 9606.ENSP00000261707 | 9606.ENSP00000280155 | 0 | 0 | 0 | 0 | 0 | 0.057 | 0 | 0.564 | 0.571 |
| SLC6A4 | IL6 | 9606.ENSP00000261707 | 9606.ENSP00000385675 | 0 | 0 | 0 | 0 | 0 | 0 | 0 | 0.598 | 0.598 |
| SLC6A4 | OPRM1 | 9606.ENSP00000261707 | 9606.ENSP00000394624 | 0 | 0 | 0 | 0 | 0 | 0.057 | 0 | 0.681 | 0.686 |
| SLC6A4 | MAOB | 9606.ENSP00000261707 | 9606.ENSP00000367309 | 0 | 0 | 0 | 0 | 0.062 | 0 | 0 | 0.688 | 0.695 |
| SLC6A4 | HTR3A | 9606.ENSP00000261707 | 9606.ENSP00000347754 | 0 | 0 | 0 | 0 | 0 | 0 | 0 | 0.71 | 0.71 |
| SLC6A4 | DRD1 | 9606.ENSP00000261707 | 9606.ENSP00000377353 | 0 | 0 | 0 | 0 | 0.057 | 0.057 | 0 | 0.722 | 0.731 |
| SLC6A4 | MAOA | 9606.ENSP00000261707 | 9606.ENSP00000340684 | 0 | 0 | 0 | 0 | 0.062 | 0 | 0 | 0.884 | 0.887 |
| SLC6A4 | HTR2A | 9606.ENSP00000261707 | 9606.ENSP00000437737 | 0 | 0 | 0 | 0 | 0 | 0.057 | 0 | 0.912 | 0.914 |
| SOAT1 | HMGCR | 9606.ENSP00000356591 | 9606.ENSP00000287936 | 0 | 0 | 0 | 0 | 0 | 0 | 0 | 0.639 | 0.639 |
| SOAT1 | LDLR | 9606.ENSP00000356591 | 9606.ENSP00000454071 | 0 | 0 | 0 | 0 | 0.062 | 0 | 0 | 0.504 | 0.515 |
| SOD1 | HMOX1 | 9606.ENSP00000270142 | 9606.ENSP00000216117 | 0 | 0 | 0 | 0 | 0 | 0 | 0 | 0.708 | 0.708 |
| SOD1 | GSR | 9606.ENSP00000270142 | 9606.ENSP00000221130 | 0 | 0 | 0 | 0 | 0.073 | 0.117 | 0 | 0.84 | 0.858 |
| SOD1 | CCL2 | 9606.ENSP00000270142 | 9606.ENSP00000225831 | 0 | 0 | 0 | 0 | 0 | 0 | 0 | 0.427 | 0.426 |
| SOD1 | CAT | 9606.ENSP00000270142 | 9606.ENSP00000241052 | 0.111 | 0 | 0 | 0 | 0.124 | 0.52 | 0.9 | 0.923 | 0.996 |
| SOD1 | MAPK3 | 9606.ENSP00000270142 | 9606.ENSP00000263025 | 0 | 0 | 0 | 0 | 0.049 | 0.127 | 0 | 0.416 | 0.473 |
| SOD1 | IL1B | 9606.ENSP00000270142 | 9606.ENSP00000263341 | 0 | 0 | 0 | 0 | 0 | 0 | 0 | 0.562 | 0.562 |
| SOD1 | TP53 | 9606.ENSP00000270142 | 9606.ENSP00000269305 | 0 | 0 | 0 | 0 | 0 | 0 | 0 | 0.553 | 0.553 |
| SOD1 | JUN | 9606.ENSP00000270142 | 9606.ENSP00000360266 | 0 | 0 | 0 | 0 | 0 | 0 | 0 | 0.439 | 0.439 |
| SOD1 | ABCC1 | 9606.ENSP00000270142 | 9606.ENSP00000382342 | 0 | 0 | 0 | 0 | 0 | 0.131 | 0 | 0.387 | 0.445 |
| SOD1 | NOS2 | 9606.ENSP00000270142 | 9606.ENSP00000327251 | 0 | 0 | 0 | 0 | 0 | 0.115 | 0 | 0.406 | 0.452 |
| SOD1 | PPARG | 9606.ENSP00000270142 | 9606.ENSP00000287820 | 0 | 0 | 0 | 0 | 0 | 0 | 0 | 0.459 | 0.459 |
| SOD1 | PTGS2 | 9606.ENSP00000270142 | 9606.ENSP00000356438 | 0 | 0 | 0 | 0 | 0.062 | 0 | 0 | 0.457 | 0.468 |
| SOD1 | VEGFA | 9606.ENSP00000270142 | 9606.ENSP00000478570 | 0 | 0 | 0 | 0 | 0.063 | 0 | 0 | 0.503 | 0.514 |
| SOD1 | NOS3 | 9606.ENSP00000270142 | 9606.ENSP00000297494 | 0 | 0 | 0 | 0 | 0 | 0 | 0 | 0.523 | 0.523 |
| SOD1 | GSTP1 | 9606.ENSP00000270142 | 9606.ENSP00000381607 | 0 | 0 | 0 | 0 | 0.063 | 0.129 | 0 | 0.47 | 0.53 |
| SOD1 | XDH | 9606.ENSP00000270142 | 9606.ENSP00000368727 | 0 | 0 | 0 | 0 | 0 | 0 | 0 | 0.535 | 0.535 |
| SOD1 | IL6 | 9606.ENSP00000270142 | 9606.ENSP00000385675 | 0 | 0 | 0 | 0 | 0 | 0 | 0 | 0.535 | 0.535 |
| SOD1 | TNF | 9606.ENSP00000270142 | 9606.ENSP00000398698 | 0 | 0 | 0 | 0 | 0.049 | 0 | 0 | 0.583 | 0.587 |
| SOD1 | NQO1 | 9606.ENSP00000270142 | 9606.ENSP00000319788 | 0 | 0 | 0 | 0 | 0.062 | 0 | 0 | 0.638 | 0.646 |
| SOD1 | ESR1 | 9606.ENSP00000270142 | 9606.ENSP00000405330 | 0 | 0 | 0 | 0 | 0.062 | 0 | 0 | 0.703 | 0.71 |
| SOD1 | PPP3CA | 9606.ENSP00000270142 | 9606.ENSP00000378323 | 0 | 0 | 0 | 0 | 0 | 0.271 | 0.8 | 0.104 | 0.858 |
| SOD1 | BCL2 | 9606.ENSP00000270142 | 9606.ENSP00000381185 | 0 | 0 | 0 | 0 | 0 | 0.297 | 0.8 | 0.15 | 0.87 |
| SOD1 | HSPA5 | 9606.ENSP00000270142 | 9606.ENSP00000324173 | 0 | 0 | 0 | 0 | 0 | 0.149 | 0.8 | 0.722 | 0.948 |
| THBD | HMOX1 | 9606.ENSP00000366307 | 9606.ENSP00000216117 | 0 | 0 | 0 | 0 | 0.088 | 0 | 0 | 0.375 | 0.405 |
| THBD | PLAT | 9606.ENSP00000366307 | 9606.ENSP00000220809 | 0 | 0 | 0 | 0 | 0.062 | 0 | 0 | 0.711 | 0.717 |
| THBD | CCL2 | 9606.ENSP00000366307 | 9606.ENSP00000225831 | 0 | 0 | 0 | 0 | 0.095 | 0 | 0 | 0.504 | 0.532 |
| THBD | IL1B | 9606.ENSP00000366307 | 9606.ENSP00000263341 | 0 | 0 | 0 | 0 | 0.153 | 0 | 0 | 0.556 | 0.607 |
| THBD | VCAM1 | 9606.ENSP00000366307 | 9606.ENSP00000294728 | 0 | 0 | 0 | 0 | 0.062 | 0 | 0 | 0.666 | 0.673 |
| THBD | NOS3 | 9606.ENSP00000366307 | 9606.ENSP00000297494 | 0 | 0 | 0 | 0 | 0.062 | 0 | 0 | 0.603 | 0.611 |
| THBD | F2 | 9606.ENSP00000366307 | 9606.ENSP00000308541 | 0 | 0 | 0 | 0 | 0 | 0.962 | 0.8 | 0.925 | 0.999 |
| THBD | SELE | 9606.ENSP00000366307 | 9606.ENSP00000331736 | 0 | 0 | 0 | 0 | 0.076 | 0 | 0 | 0.702 | 0.712 |
| THBD | F3 | 9606.ENSP00000366307 | 9606.ENSP00000334145 | 0 | 0 | 0 | 0 | 0.062 | 0 | 0 | 0.864 | 0.867 |
| THBD | PLAU | 9606.ENSP00000366307 | 9606.ENSP00000361850 | 0 | 0 | 0 | 0 | 0.098 | 0 | 0 | 0.519 | 0.548 |
| THBD | F10 | 9606.ENSP00000366307 | 9606.ENSP00000364709 | 0 | 0 | 0 | 0 | 0 | 0 | 0.65 | 0.639 | 0.868 |
| THBD | F7 | 9606.ENSP00000366307 | 9606.ENSP00000364731 | 0 | 0 | 0 | 0 | 0 | 0 | 0 | 0.681 | 0.681 |
| THBD | VEGFA | 9606.ENSP00000366307 | 9606.ENSP00000478570 | 0 | 0 | 0 | 0 | 0.065 | 0 | 0 | 0.527 | 0.538 |
| THBD | TNF | 9606.ENSP00000366307 | 9606.ENSP00000398698 | 0 | 0 | 0 | 0 | 0.062 | 0 | 0 | 0.619 | 0.627 |
| THBD | IL6 | 9606.ENSP00000366307 | 9606.ENSP00000385675 | 0 | 0 | 0 | 0 | 0.098 | 0 | 0 | 0.617 | 0.639 |
| TNF | MAPK1 | 9606.ENSP00000398698 | 9606.ENSP00000215832 | 0 | 0 | 0 | 0 | 0 | 0 | 0 | 0.538 | 0.538 |
| TNF | HMOX1 | 9606.ENSP00000398698 | 9606.ENSP00000216117 | 0 | 0 | 0 | 0 | 0 | 0 | 0 | 0.775 | 0.775 |
| TNF | MMP2 | 9606.ENSP00000398698 | 9606.ENSP00000219070 | 0 | 0 | 0 | 0 | 0 | 0 | 0 | 0.734 | 0.734 |
| TNF | PLAT | 9606.ENSP00000398698 | 9606.ENSP00000220809 | 0 | 0 | 0 | 0 | 0 | 0 | 0 | 0.537 | 0.537 |
| TNF | GSR | 9606.ENSP00000398698 | 9606.ENSP00000221130 | 0 | 0 | 0 | 0 | 0 | 0 | 0 | 0.55 | 0.55 |
| TNF | PON1 | 9606.ENSP00000398698 | 9606.ENSP00000222381 | 0 | 0 | 0 | 0 | 0 | 0 | 0 | 0.468 | 0.468 |
| TNF | MPO | 9606.ENSP00000398698 | 9606.ENSP00000225275 | 0 | 0 | 0 | 0 | 0.074 | 0 | 0 | 0.861 | 0.866 |
| TNF | CCL2 | 9606.ENSP00000398698 | 9606.ENSP00000225831 | 0 | 0 | 0 | 0 | 0.062 | 0 | 0.9 | 0.93 | 0.992 |
| TNF | IL2 | 9606.ENSP00000398698 | 9606.ENSP00000226730 | 0 | 0 | 0 | 0 | 0 | 0 | 0 | 0.933 | 0.933 |
| TNF | LTA4H | 9606.ENSP00000398698 | 9606.ENSP00000228740 | 0 | 0 | 0 | 0 | 0 | 0 | 0 | 0.471 | 0.471 |
| TNF | IFNG | 9606.ENSP00000398698 | 9606.ENSP00000229135 | 0 | 0 | 0 | 0 | 0.152 | 0.213 | 0 | 0.915 | 0.938 |
| TNF | MAPK14 | 9606.ENSP00000398698 | 9606.ENSP00000229795 | 0 | 0 | 0 | 0 | 0 | 0 | 0.9 | 0.768 | 0.975 |
| TNF | NR3C1 | 9606.ENSP00000398698 | 9606.ENSP00000231509 | 0 | 0 | 0 | 0 | 0 | 0 | 0.9 | 0.621 | 0.96 |
| TNF | CTSD | 9606.ENSP00000398698 | 9606.ENSP00000236671 | 0 | 0 | 0 | 0 | 0.062 | 0 | 0 | 0.536 | 0.547 |
| TNF | CAT | 9606.ENSP00000398698 | 9606.ENSP00000241052 | 0 | 0 | 0 | 0 | 0 | 0 | 0 | 0.779 | 0.779 |
| TNF | AHR | 9606.ENSP00000398698 | 9606.ENSP00000242057 | 0 | 0 | 0 | 0 | 0 | 0 | 0 | 0.673 | 0.674 |
| TNF | CDK4 | 9606.ENSP00000398698 | 9606.ENSP00000257904 | 0 | 0 | 0 | 0 | 0 | 0.065 | 0 | 0.429 | 0.443 |
| TNF | MAPK3 | 9606.ENSP00000398698 | 9606.ENSP00000263025 | 0 | 0 | 0 | 0 | 0 | 0 | 0 | 0.765 | 0.765 |
| TNF | IL1B | 9606.ENSP00000398698 | 9606.ENSP00000263341 | 0 | 0 | 0 | 0 | 0.462 | 0 | 0.5 | 0.942 | 0.983 |
| TNF | KDR | 9606.ENSP00000398698 | 9606.ENSP00000263923 | 0 | 0 | 0 | 0 | 0 | 0 | 0 | 0.623 | 0.623 |
| TNF | EGF | 9606.ENSP00000398698 | 9606.ENSP00000265171 | 0 | 0 | 0 | 0 | 0 | 0.059 | 0 | 0.783 | 0.787 |
| TNF | CDK2 | 9606.ENSP00000398698 | 9606.ENSP00000266970 | 0 | 0 | 0 | 0 | 0 | 0.065 | 0 | 0.474 | 0.487 |
| TNF | TP53 | 9606.ENSP00000398698 | 9606.ENSP00000269305 | 0 | 0 | 0 | 0 | 0 | 0 | 0 | 0.784 | 0.784 |
| TNF | SOD1 | 9606.ENSP00000398698 | 9606.ENSP00000270142 | 0 | 0 | 0 | 0 | 0.049 | 0 | 0 | 0.583 | 0.587 |
| TNF | EGFR | 9606.ENSP00000398698 | 9606.ENSP00000275493 | 0 | 0 | 0 | 0 | 0.063 | 0 | 0 | 0.704 | 0.712 |
| TNF | GJA1 | 9606.ENSP00000398698 | 9606.ENSP00000282561 | 0 | 0 | 0 | 0 | 0 | 0 | 0 | 0.535 | 0.535 |
| TNF | PPARG | 9606.ENSP00000398698 | 9606.ENSP00000287820 | 0 | 0 | 0 | 0 | 0 | 0 | 0.9 | 0.807 | 0.979 |
| TNF | HMGCR | 9606.ENSP00000398698 | 9606.ENSP00000287936 | 0 | 0 | 0 | 0 | 0 | 0 | 0 | 0.415 | 0.415 |
| TNF | BAX | 9606.ENSP00000398698 | 9606.ENSP00000293288 | 0 | 0 | 0 | 0 | 0 | 0.059 | 0 | 0.457 | 0.467 |
| TNF | VCAM1 | 9606.ENSP00000398698 | 9606.ENSP00000294728 | 0 | 0 | 0 | 0 | 0 | 0 | 0.9 | 0.884 | 0.987 |
| TNF | NOS3 | 9606.ENSP00000398698 | 9606.ENSP00000297494 | 0 | 0 | 0 | 0 | 0.062 | 0 | 0 | 0.75 | 0.756 |
| TNF | MMP3 | 9606.ENSP00000398698 | 9606.ENSP00000299855 | 0 | 0 | 0 | 0 | 0.064 | 0 | 0 | 0.762 | 0.767 |
| TNF | ACHE | 9606.ENSP00000398698 | 9606.ENSP00000303211 | 0 | 0 | 0 | 0 | 0 | 0 | 0 | 0.521 | 0.521 |
| TNF | FASN | 9606.ENSP00000398698 | 9606.ENSP00000304592 | 0 | 0 | 0 | 0 | 0 | 0 | 0 | 0.438 | 0.438 |
| TNF | ADRB2 | 9606.ENSP00000398698 | 9606.ENSP00000305372 | 0 | 0 | 0 | 0 | 0.065 | 0 | 0 | 0.554 | 0.566 |
| TNF | F2 | 9606.ENSP00000398698 | 9606.ENSP00000308541 | 0 | 0 | 0 | 0 | 0 | 0 | 0 | 0.627 | 0.627 |
| TNF | PPARD | 9606.ENSP00000398698 | 9606.ENSP00000310928 | 0 | 0 | 0 | 0 | 0 | 0 | 0 | 0.451 | 0.451 |
| TNF | NQO1 | 9606.ENSP00000398698 | 9606.ENSP00000319788 | 0 | 0 | 0 | 0 | 0 | 0.114 | 0 | 0.577 | 0.609 |
| TNF | MMP1 | 9606.ENSP00000398698 | 9606.ENSP00000322788 | 0 | 0 | 0 | 0 | 0.064 | 0 | 0 | 0.7 | 0.707 |
| TNF | HSPA5 | 9606.ENSP00000398698 | 9606.ENSP00000324173 | 0 | 0 | 0 | 0 | 0 | 0.123 | 0 | 0.585 | 0.621 |
| TNF | GSK3B | 9606.ENSP00000398698 | 9606.ENSP00000324806 | 0 | 0 | 0 | 0 | 0 | 0.056 | 0 | 0.58 | 0.587 |
| TNF | PGR | 9606.ENSP00000398698 | 9606.ENSP00000325120 | 0 | 0 | 0 | 0 | 0 | 0 | 0 | 0.406 | 0.406 |
| TNF | NOS2 | 9606.ENSP00000398698 | 9606.ENSP00000327251 | 0 | 0 | 0 | 0 | 0.063 | 0 | 0.9 | 0.796 | 0.979 |
| TNF | SELE | 9606.ENSP00000398698 | 9606.ENSP00000331736 | 0 | 0 | 0 | 0 | 0.06 | 0 | 0 | 0.835 | 0.838 |
| TNF | F3 | 9606.ENSP00000398698 | 9606.ENSP00000334145 | 0 | 0 | 0 | 0 | 0.063 | 0 | 0 | 0.698 | 0.705 |
| TNF | ESR2 | 9606.ENSP00000398698 | 9606.ENSP00000343925 | 0 | 0 | 0 | 0 | 0 | 0 | 0 | 0.514 | 0.514 |
| TNF | PTGER3 | 9606.ENSP00000398698 | 9606.ENSP00000349003 | 0 | 0 | 0 | 0 | 0 | 0 | 0 | 0.428 | 0.428 |
| TNF | PIK3CG | 9606.ENSP00000398698 | 9606.ENSP00000352121 | 0 | 0 | 0 | 0 | 0.129 | 0 | 0 | 0.42 | 0.474 |
| TNF | DPP4 | 9606.ENSP00000398698 | 9606.ENSP00000353731 | 0 | 0 | 0 | 0 | 0 | 0 | 0 | 0.532 | 0.532 |
| TNF | PTGS1 | 9606.ENSP00000398698 | 9606.ENSP00000354612 | 0 | 0 | 0 | 0 | 0.085 | 0 | 0 | 0.548 | 0.569 |
| TNF | PTGS2 | 9606.ENSP00000398698 | 9606.ENSP00000356438 | 0 | 0 | 0 | 0 | 0.096 | 0 | 0 | 0.855 | 0.863 |
| TNF | JUN | 9606.ENSP00000398698 | 9606.ENSP00000360266 | 0 | 0 | 0 | 0 | 0 | 0 | 0.9 | 0.847 | 0.984 |
| TNF | PTPN1 | 9606.ENSP00000398698 | 9606.ENSP00000360683 | 0 | 0 | 0 | 0 | 0 | 0.057 | 0 | 0.447 | 0.456 |
| TNF | PLAU | 9606.ENSP00000398698 | 9606.ENSP00000361850 | 0 | 0 | 0 | 0 | 0.062 | 0 | 0 | 0.491 | 0.502 |
| TNF | ALOX5 | 9606.ENSP00000398698 | 9606.ENSP00000363512 | 0 | 0 | 0 | 0 | 0.121 | 0 | 0 | 0.62 | 0.652 |
| TNF | AR | 9606.ENSP00000398698 | 9606.ENSP00000363822 | 0 | 0 | 0 | 0 | 0 | 0 | 0 | 0.469 | 0.469 |
| TNF | F10 | 9606.ENSP00000398698 | 9606.ENSP00000364709 | 0 | 0 | 0 | 0 | 0.062 | 0 | 0 | 0.513 | 0.524 |
| TNF | F7 | 9606.ENSP00000398698 | 9606.ENSP00000364731 | 0 | 0 | 0 | 0 | 0.062 | 0 | 0 | 0.392 | 0.405 |
| TNF | THBD | 9606.ENSP00000398698 | 9606.ENSP00000366307 | 0 | 0 | 0 | 0 | 0.062 | 0 | 0 | 0.619 | 0.627 |
| TNF | XDH | 9606.ENSP00000398698 | 9606.ENSP00000368727 | 0 | 0 | 0 | 0 | 0.062 | 0 | 0 | 0.494 | 0.505 |
| TNF | MAPK8 | 9606.ENSP00000398698 | 9606.ENSP00000378974 | 0 | 0 | 0 | 0 | 0.052 | 0 | 0 | 0.696 | 0.7 |
| TNF | ABCC1 | 9606.ENSP00000398698 | 9606.ENSP00000382342 | 0 | 0 | 0 | 0 | 0 | 0 | 0 | 0.447 | 0.447 |
| TNF | IL6 | 9606.ENSP00000398698 | 9606.ENSP00000385675 | 0 | 0 | 0 | 0 | 0.125 | 0 | 0.9 | 0.941 | 0.994 |
| TNF | LDLR | 9606.ENSP00000398698 | 9606.ENSP00000454071 | 0 | 0 | 0 | 0 | 0 | 0.062 | 0 | 0.519 | 0.53 |
| TNF | ESR1 | 9606.ENSP00000398698 | 9606.ENSP00000405330 | 0 | 0 | 0 | 0 | 0 | 0 | 0 | 0.622 | 0.622 |
| TNF | VEGFA | 9606.ENSP00000398698 | 9606.ENSP00000478570 | 0 | 0 | 0 | 0 | 0 | 0 | 0 | 0.88 | 0.88 |
| TNF | RXRA | 9606.ENSP00000398698 | 9606.ENSP00000419692 | 0 | 0 | 0 | 0 | 0 | 0 | 0.9 | 0.328 | 0.93 |
| TOP1 | CDK4 | 9606.ENSP00000354522 | 9606.ENSP00000257904 | 0 | 0 | 0 | 0 | 0.063 | 0.192 | 0 | 0.361 | 0.474 |
| TOP1 | CDK2 | 9606.ENSP00000354522 | 9606.ENSP00000266970 | 0 | 0 | 0 | 0 | 0.091 | 0.078 | 0 | 0.513 | 0.557 |
| TOP1 | TP53 | 9606.ENSP00000354522 | 9606.ENSP00000269305 | 0 | 0 | 0 | 0 | 0.065 | 0.735 | 0 | 0.97 | 0.991 |
| TOP1 | CCNA2 | 9606.ENSP00000354522 | 9606.ENSP00000274026 | 0 | 0 | 0 | 0 | 0.086 | 0.061 | 0 | 0.39 | 0.431 |
| TOP1 | EGFR | 9606.ENSP00000354522 | 9606.ENSP00000275493 | 0 | 0 | 0 | 0 | 0 | 0.27 | 0 | 0.602 | 0.697 |
| TOP1 | ABCC1 | 9606.ENSP00000354522 | 9606.ENSP00000382342 | 0 | 0 | 0 | 0 | 0 | 0.076 | 0 | 0.408 | 0.43 |
| TOP1 | AR | 9606.ENSP00000354522 | 9606.ENSP00000363822 | 0 | 0 | 0 | 0 | 0.062 | 0 | 0 | 0.436 | 0.448 |
| TOP1 | VEGFA | 9606.ENSP00000354522 | 9606.ENSP00000478570 | 0 | 0 | 0 | 0 | 0 | 0 | 0 | 0.468 | 0.468 |
| TOP1 | CDK1 | 9606.ENSP00000354522 | 9606.ENSP00000378699 | 0 | 0 | 0 | 0 | 0.09 | 0.056 | 0 | 0.49 | 0.524 |
| TOP1 | JUN | 9606.ENSP00000354522 | 9606.ENSP00000360266 | 0 | 0 | 0 | 0 | 0 | 0.284 | 0 | 0.633 | 0.726 |
| TOP1 | CHEK1 | 9606.ENSP00000354522 | 9606.ENSP00000388648 | 0 | 0 | 0 | 0 | 0.062 | 0.205 | 0 | 0.82 | 0.854 |
| TOP1 | TOP2A | 9606.ENSP00000354522 | 9606.ENSP00000411532 | 0 | 0 | 0 | 0 | 0.111 | 0.919 | 0 | 0.919 | 0.993 |
| TOP2A | MAPK1 | 9606.ENSP00000411532 | 9606.ENSP00000215832 | 0 | 0 | 0 | 0 | 0.064 | 0.345 | 0 | 0.15 | 0.433 |
| TOP2A | CDK4 | 9606.ENSP00000411532 | 9606.ENSP00000257904 | 0 | 0 | 0 | 0 | 0.212 | 0.056 | 0 | 0.418 | 0.53 |
| TOP2A | CDK2 | 9606.ENSP00000411532 | 9606.ENSP00000266970 | 0 | 0 | 0 | 0 | 0.376 | 0.056 | 0 | 0.454 | 0.65 |
| TOP2A | RB1 | 9606.ENSP00000411532 | 9606.ENSP00000267163 | 0 | 0 | 0 | 0 | 0.122 | 0.27 | 0 | 0.141 | 0.401 |
| TOP2A | TP53 | 9606.ENSP00000411532 | 9606.ENSP00000269305 | 0 | 0 | 0 | 0 | 0.107 | 0.382 | 0 | 0.668 | 0.801 |
| TOP2A | CCNA2 | 9606.ENSP00000411532 | 9606.ENSP00000274026 | 0 | 0 | 0 | 0 | 0.967 | 0.056 | 0 | 0.575 | 0.985 |
| TOP2A | EGFR | 9606.ENSP00000411532 | 9606.ENSP00000275493 | 0 | 0 | 0 | 0 | 0 | 0 | 0 | 0.515 | 0.515 |
| TOP2A | PGR | 9606.ENSP00000411532 | 9606.ENSP00000325120 | 0 | 0 | 0 | 0 | 0.062 | 0 | 0 | 0.457 | 0.468 |
| TOP2A | TOP1 | 9606.ENSP00000411532 | 9606.ENSP00000354522 | 0 | 0 | 0 | 0 | 0.111 | 0.919 | 0 | 0.919 | 0.993 |
| TOP2A | JUN | 9606.ENSP00000411532 | 9606.ENSP00000360266 | 0 | 0 | 0 | 0 | 0 | 0.298 | 0 | 0.233 | 0.438 |
| TOP2A | CDK1 | 9606.ENSP00000411532 | 9606.ENSP00000378699 | 0 | 0 | 0 | 0 | 0.988 | 0.225 | 0 | 0.672 | 0.996 |
| TOP2A | CHEK1 | 9606.ENSP00000411532 | 9606.ENSP00000388648 | 0 | 0 | 0 | 0 | 0.803 | 0.205 | 0 | 0.678 | 0.945 |
| TOP2A | ESR1 | 9606.ENSP00000411532 | 9606.ENSP00000405330 | 0 | 0 | 0 | 0 | 0.062 | 0.044 | 0 | 0.469 | 0.482 |
| TP53 | MAPK1 | 9606.ENSP00000269305 | 9606.ENSP00000215832 | 0 | 0 | 0 | 0 | 0.055 | 0.876 | 0.8 | 0.695 | 0.991 |
| TP53 | HMOX1 | 9606.ENSP00000269305 | 9606.ENSP00000216117 | 0 | 0 | 0 | 0 | 0.062 | 0 | 0 | 0.611 | 0.619 |
| TP53 | MMP2 | 9606.ENSP00000269305 | 9606.ENSP00000219070 | 0 | 0 | 0 | 0 | 0.062 | 0 | 0 | 0.669 | 0.676 |
| TP53 | GSR | 9606.ENSP00000269305 | 9606.ENSP00000221130 | 0 | 0 | 0 | 0 | 0 | 0 | 0 | 0.508 | 0.508 |
| TP53 | MPO | 9606.ENSP00000269305 | 9606.ENSP00000225275 | 0 | 0 | 0 | 0 | 0 | 0 | 0 | 0.424 | 0.424 |
| TP53 | CCL2 | 9606.ENSP00000269305 | 9606.ENSP00000225831 | 0 | 0 | 0 | 0 | 0 | 0 | 0 | 0.644 | 0.644 |
| TP53 | IL2 | 9606.ENSP00000269305 | 9606.ENSP00000226730 | 0 | 0 | 0 | 0 | 0 | 0 | 0 | 0.667 | 0.667 |
| TP53 | IFNG | 9606.ENSP00000269305 | 9606.ENSP00000229135 | 0 | 0 | 0 | 0 | 0 | 0 | 0 | 0.657 | 0.657 |
| TP53 | MAPK14 | 9606.ENSP00000269305 | 9606.ENSP00000229795 | 0 | 0 | 0 | 0 | 0 | 0.878 | 0.9 | 0.655 | 0.995 |
| TP53 | NR3C1 | 9606.ENSP00000269305 | 9606.ENSP00000231509 | 0 | 0 | 0 | 0 | 0 | 0.486 | 0.9 | 0.637 | 0.979 |
| TP53 | ODC1 | 9606.ENSP00000269305 | 9606.ENSP00000234111 | 0 | 0 | 0 | 0 | 0.054 | 0 | 0 | 0.504 | 0.51 |
| TP53 | CTSD | 9606.ENSP00000269305 | 9606.ENSP00000236671 | 0 | 0 | 0 | 0 | 0 | 0 | 0 | 0.518 | 0.518 |
| TP53 | CAT | 9606.ENSP00000269305 | 9606.ENSP00000241052 | 0 | 0 | 0 | 0 | 0 | 0 | 0 | 0.689 | 0.689 |
| TP53 | AHR | 9606.ENSP00000269305 | 9606.ENSP00000242057 | 0 | 0 | 0 | 0 | 0 | 0 | 0 | 0.518 | 0.518 |
| TP53 | CDK4 | 9606.ENSP00000269305 | 9606.ENSP00000257904 | 0 | 0 | 0 | 0 | 0.093 | 0.498 | 0 | 0.871 | 0.936 |
| TP53 | MAPK3 | 9606.ENSP00000269305 | 9606.ENSP00000263025 | 0 | 0 | 0 | 0 | 0.062 | 0.68 | 0.8 | 0.721 | 0.981 |
| TP53 | IL1B | 9606.ENSP00000269305 | 9606.ENSP00000263341 | 0 | 0 | 0 | 0 | 0 | 0 | 0 | 0.689 | 0.689 |
| TP53 | KDR | 9606.ENSP00000269305 | 9606.ENSP00000263923 | 0 | 0 | 0 | 0 | 0 | 0.209 | 0 | 0.633 | 0.697 |
| TP53 | EGF | 9606.ENSP00000269305 | 9606.ENSP00000265171 | 0 | 0 | 0 | 0 | 0 | 0 | 0 | 0.766 | 0.766 |
| TP53 | CDK2 | 9606.ENSP00000269305 | 9606.ENSP00000266970 | 0 | 0 | 0 | 0 | 0.095 | 0.79 | 0.9 | 0.97 | 0.999 |
| TP53 | RB1 | 9606.ENSP00000269305 | 9606.ENSP00000267163 | 0 | 0 | 0 | 0 | 0.056 | 0.486 | 0.9 | 0.823 | 0.99 |
| TP53 | GJA1 | 9606.ENSP00000269305 | 9606.ENSP00000282561 | 0 | 0 | 0 | 0 | 0 | 0 | 0 | 0.413 | 0.412 |
| TP53 | PTPN1 | 9606.ENSP00000269305 | 9606.ENSP00000360683 | 0 | 0 | 0 | 0 | 0.054 | 0 | 0 | 0.405 | 0.413 |
| TP53 | NOS2 | 9606.ENSP00000269305 | 9606.ENSP00000327251 | 0 | 0 | 0 | 0 | 0 | 0 | 0 | 0.417 | 0.417 |
| TP53 | CYP3A4 | 9606.ENSP00000269305 | 9606.ENSP00000337915 | 0 | 0 | 0 | 0 | 0 | 0.058 | 0 | 0.417 | 0.427 |
| TP53 | ABCC1 | 9606.ENSP00000269305 | 9606.ENSP00000382342 | 0 | 0 | 0 | 0 | 0 | 0 | 0 | 0.46 | 0.459 |
| TP53 | VCAM1 | 9606.ENSP00000269305 | 9606.ENSP00000294728 | 0 | 0 | 0 | 0 | 0 | 0 | 0 | 0.459 | 0.459 |
| TP53 | FASN | 9606.ENSP00000269305 | 9606.ENSP00000304592 | 0 | 0 | 0 | 0 | 0 | 0 | 0 | 0.464 | 0.463 |
| TP53 | ALOX5 | 9606.ENSP00000269305 | 9606.ENSP00000363512 | 0 | 0 | 0 | 0 | 0 | 0 | 0 | 0.475 | 0.475 |
| TP53 | PIK3CG | 9606.ENSP00000269305 | 9606.ENSP00000352121 | 0 | 0 | 0 | 0 | 0 | 0.056 | 0 | 0.475 | 0.483 |
| TP53 | NCOA2 | 9606.ENSP00000269305 | 9606.ENSP00000399968 | 0 | 0 | 0 | 0 | 0 | 0.068 | 0 | 0.493 | 0.508 |
| TP53 | MMP3 | 9606.ENSP00000269305 | 9606.ENSP00000299855 | 0 | 0 | 0 | 0 | 0 | 0 | 0 | 0.523 | 0.523 |
| TP53 | MMP1 | 9606.ENSP00000269305 | 9606.ENSP00000322788 | 0 | 0 | 0 | 0 | 0 | 0 | 0 | 0.524 | 0.524 |
| TP53 | SOD1 | 9606.ENSP00000269305 | 9606.ENSP00000270142 | 0 | 0 | 0 | 0 | 0 | 0 | 0 | 0.553 | 0.553 |
| TP53 | PLAU | 9606.ENSP00000269305 | 9606.ENSP00000361850 | 0 | 0 | 0 | 0 | 0 | 0 | 0 | 0.56 | 0.56 |
| TP53 | NOS3 | 9606.ENSP00000269305 | 9606.ENSP00000297494 | 0 | 0 | 0 | 0 | 0 | 0 | 0 | 0.564 | 0.564 |
| TP53 | NCOA1 | 9606.ENSP00000269305 | 9606.ENSP00000385216 | 0 | 0 | 0 | 0 | 0 | 0.27 | 0 | 0.51 | 0.627 |
| TP53 | BAD | 9606.ENSP00000269305 | 9606.ENSP00000378040 | 0 | 0 | 0 | 0 | 0 | 0 | 0 | 0.64 | 0.64 |
| TP53 | GSTP1 | 9606.ENSP00000269305 | 9606.ENSP00000381607 | 0 | 0 | 0 | 0 | 0 | 0 | 0 | 0.665 | 0.665 |
| TP53 | IL6 | 9606.ENSP00000269305 | 9606.ENSP00000385675 | 0 | 0 | 0 | 0 | 0.062 | 0 | 0 | 0.738 | 0.744 |
| TP53 | TNF | 9606.ENSP00000269305 | 9606.ENSP00000398698 | 0 | 0 | 0 | 0 | 0 | 0 | 0 | 0.784 | 0.784 |
| TP53 | TOP2A | 9606.ENSP00000269305 | 9606.ENSP00000411532 | 0 | 0 | 0 | 0 | 0.107 | 0.382 | 0 | 0.668 | 0.801 |
| TP53 | PGR | 9606.ENSP00000269305 | 9606.ENSP00000325120 | 0 | 0 | 0 | 0 | 0.063 | 0 | 0 | 0.805 | 0.81 |
| TP53 | VEGFA | 9606.ENSP00000269305 | 9606.ENSP00000478570 | 0 | 0 | 0 | 0 | 0 | 0.161 | 0 | 0.81 | 0.834 |
| TP53 | AR | 9606.ENSP00000269305 | 9606.ENSP00000363822 | 0 | 0 | 0 | 0 | 0 | 0.474 | 0 | 0.776 | 0.877 |
| TP53 | ESR2 | 9606.ENSP00000269305 | 9606.ENSP00000343925 | 0 | 0 | 0 | 0 | 0 | 0 | 0 | 0.881 | 0.881 |
| TP53 | HSPA5 | 9606.ENSP00000269305 | 9606.ENSP00000324173 | 0 | 0 | 0 | 0 | 0 | 0.428 | 0 | 0.822 | 0.894 |
| TP53 | EGFR | 9606.ENSP00000269305 | 9606.ENSP00000275493 | 0 | 0 | 0 | 0 | 0 | 0.279 | 0 | 0.877 | 0.908 |
| TP53 | MAPK10 | 9606.ENSP00000269305 | 9606.ENSP00000352157 | 0 | 0 | 0 | 0 | 0 | 0.333 | 0.8 | 0.398 | 0.912 |
| TP53 | PPARG | 9606.ENSP00000269305 | 9606.ENSP00000287820 | 0 | 0 | 0 | 0 | 0 | 0.292 | 0.65 | 0.719 | 0.924 |
| TP53 | DPP4 | 9606.ENSP00000269305 | 9606.ENSP00000353731 | 0 | 0 | 0 | 0 | 0 | 0 | 0 | 0.928 | 0.928 |
| TP53 | JUN | 9606.ENSP00000269305 | 9606.ENSP00000360266 | 0 | 0 | 0 | 0 | 0 | 0.149 | 0.6 | 0.838 | 0.94 |
| TP53 | CDK1 | 9606.ENSP00000269305 | 9606.ENSP00000378699 | 0 | 0 | 0 | 0 | 0.096 | 0.68 | 0 | 0.927 | 0.977 |
| TP53 | BAX | 9606.ENSP00000269305 | 9606.ENSP00000293288 | 0 | 0 | 0 | 0 | 0.051 | 0.738 | 0.9 | 0.681 | 0.991 |
| TP53 | TOP1 | 9606.ENSP00000269305 | 9606.ENSP00000354522 | 0 | 0 | 0 | 0 | 0.065 | 0.735 | 0 | 0.97 | 0.991 |
| TP53 | PTGS2 | 9606.ENSP00000269305 | 9606.ENSP00000356438 | 0 | 0 | 0 | 0 | 0 | 0.874 | 0 | 0.958 | 0.994 |
| TP53 | ESR1 | 9606.ENSP00000269305 | 9606.ENSP00000405330 | 0 | 0 | 0 | 0 | 0 | 0.835 | 0 | 0.968 | 0.994 |
| TP53 | BCL2 | 9606.ENSP00000269305 | 9606.ENSP00000381185 | 0 | 0 | 0 | 0 | 0.051 | 0.884 | 0.9 | 0.628 | 0.995 |
| TP53 | CCNA2 | 9606.ENSP00000269305 | 9606.ENSP00000274026 | 0 | 0 | 0 | 0 | 0.092 | 0.283 | 0.9 | 0.944 | 0.995 |
| TP53 | NQO1 | 9606.ENSP00000269305 | 9606.ENSP00000319788 | 0 | 0 | 0 | 0 | 0 | 0.835 | 0 | 0.978 | 0.996 |
| TP53 | MAPK8 | 9606.ENSP00000269305 | 9606.ENSP00000378974 | 0 | 0 | 0 | 0 | 0 | 0.884 | 0.9 | 0.827 | 0.997 |
| TP53 | CHEK1 | 9606.ENSP00000269305 | 9606.ENSP00000388648 | 0 | 0 | 0 | 0 | 0.096 | 0.875 | 0.9 | 0.861 | 0.998 |
| TP53 | GSK3B | 9606.ENSP00000269305 | 9606.ENSP00000324806 | 0 | 0 | 0 | 0 | 0 | 0.866 | 0.9 | 0.973 | 0.999 |
| VCAM1 | HMOX1 | 9606.ENSP00000294728 | 9606.ENSP00000216117 | 0 | 0 | 0 | 0 | 0.062 | 0 | 0 | 0.616 | 0.624 |
| VCAM1 | MMP2 | 9606.ENSP00000294728 | 9606.ENSP00000219070 | 0 | 0 | 0 | 0 | 0.109 | 0 | 0 | 0.602 | 0.63 |
| VCAM1 | PLAT | 9606.ENSP00000294728 | 9606.ENSP00000220809 | 0 | 0 | 0 | 0 | 0.086 | 0 | 0 | 0.476 | 0.501 |
| VCAM1 | PON1 | 9606.ENSP00000294728 | 9606.ENSP00000222381 | 0 | 0 | 0 | 0 | 0.058 | 0 | 0 | 0.392 | 0.402 |
| VCAM1 | MPO | 9606.ENSP00000294728 | 9606.ENSP00000225275 | 0 | 0 | 0 | 0 | 0 | 0 | 0 | 0.633 | 0.633 |
| VCAM1 | CCL2 | 9606.ENSP00000294728 | 9606.ENSP00000225831 | 0 | 0 | 0 | 0 | 0.202 | 0 | 0 | 0.879 | 0.899 |
| VCAM1 | IL2 | 9606.ENSP00000294728 | 9606.ENSP00000226730 | 0 | 0 | 0 | 0 | 0 | 0 | 0 | 0.603 | 0.603 |
| VCAM1 | IFNG | 9606.ENSP00000294728 | 9606.ENSP00000229135 | 0 | 0 | 0 | 0 | 0 | 0 | 0 | 0.649 | 0.649 |
| VCAM1 | MAPK14 | 9606.ENSP00000294728 | 9606.ENSP00000229795 | 0 | 0 | 0 | 0 | 0 | 0 | 0 | 0.487 | 0.488 |
| VCAM1 | CAT | 9606.ENSP00000294728 | 9606.ENSP00000241052 | 0 | 0 | 0 | 0 | 0 | 0 | 0 | 0.522 | 0.522 |
| VCAM1 | MAPK3 | 9606.ENSP00000294728 | 9606.ENSP00000263025 | 0 | 0 | 0 | 0 | 0 | 0 | 0 | 0.564 | 0.564 |
| VCAM1 | IL1B | 9606.ENSP00000294728 | 9606.ENSP00000263341 | 0 | 0 | 0 | 0 | 0.062 | 0 | 0 | 0.826 | 0.83 |
| VCAM1 | EGF | 9606.ENSP00000294728 | 9606.ENSP00000265171 | 0 | 0 | 0 | 0 | 0 | 0 | 0 | 0.557 | 0.557 |
| VCAM1 | TP53 | 9606.ENSP00000294728 | 9606.ENSP00000269305 | 0 | 0 | 0 | 0 | 0 | 0 | 0 | 0.459 | 0.459 |
| VCAM1 | EGFR | 9606.ENSP00000294728 | 9606.ENSP00000275493 | 0 | 0 | 0 | 0 | 0.076 | 0 | 0 | 0.453 | 0.472 |
| VCAM1 | GJA1 | 9606.ENSP00000294728 | 9606.ENSP00000282561 | 0 | 0 | 0 | 0 | 0.076 | 0 | 0 | 0.401 | 0.423 |
| VCAM1 | PPARG | 9606.ENSP00000294728 | 9606.ENSP00000287820 | 0 | 0 | 0 | 0 | 0.062 | 0 | 0 | 0.606 | 0.615 |
| VCAM1 | PLAU | 9606.ENSP00000294728 | 9606.ENSP00000361850 | 0 | 0 | 0 | 0 | 0.062 | 0 | 0 | 0.391 | 0.404 |
| VCAM1 | ALOX5 | 9606.ENSP00000294728 | 9606.ENSP00000363512 | 0 | 0 | 0 | 0 | 0.062 | 0 | 0 | 0.392 | 0.405 |
| VCAM1 | DPP4 | 9606.ENSP00000294728 | 9606.ENSP00000353731 | 0 | 0 | 0 | 0 | 0 | 0 | 0 | 0.428 | 0.428 |
| VCAM1 | LDLR | 9606.ENSP00000294728 | 9606.ENSP00000454071 | 0 | 0 | 0 | 0 | 0 | 0 | 0 | 0.464 | 0.464 |
| VCAM1 | NOS2 | 9606.ENSP00000294728 | 9606.ENSP00000327251 | 0 | 0 | 0 | 0 | 0 | 0 | 0 | 0.481 | 0.481 |
| VCAM1 | F2 | 9606.ENSP00000294728 | 9606.ENSP00000308541 | 0 | 0 | 0 | 0 | 0 | 0 | 0 | 0.508 | 0.508 |
| VCAM1 | MMP1 | 9606.ENSP00000294728 | 9606.ENSP00000322788 | 0 | 0 | 0 | 0 | 0.065 | 0 | 0 | 0.526 | 0.537 |
| VCAM1 | MMP3 | 9606.ENSP00000294728 | 9606.ENSP00000299855 | 0 | 0 | 0 | 0 | 0.062 | 0 | 0 | 0.543 | 0.553 |
| VCAM1 | JUN | 9606.ENSP00000294728 | 9606.ENSP00000360266 | 0 | 0 | 0 | 0 | 0 | 0 | 0 | 0.565 | 0.565 |
| VCAM1 | PTGS2 | 9606.ENSP00000294728 | 9606.ENSP00000356438 | 0 | 0 | 0 | 0 | 0.062 | 0 | 0 | 0.66 | 0.667 |
| VCAM1 | THBD | 9606.ENSP00000294728 | 9606.ENSP00000366307 | 0 | 0 | 0 | 0 | 0.062 | 0 | 0 | 0.666 | 0.673 |
| VCAM1 | F3 | 9606.ENSP00000294728 | 9606.ENSP00000334145 | 0 | 0 | 0 | 0 | 0.062 | 0 | 0 | 0.707 | 0.713 |
| VCAM1 | NOS3 | 9606.ENSP00000294728 | 9606.ENSP00000297494 | 0 | 0 | 0 | 0 | 0 | 0 | 0 | 0.757 | 0.757 |
| VCAM1 | VEGFA | 9606.ENSP00000294728 | 9606.ENSP00000478570 | 0 | 0 | 0 | 0 | 0.062 | 0 | 0 | 0.756 | 0.761 |
| VCAM1 | IL6 | 9606.ENSP00000294728 | 9606.ENSP00000385675 | 0 | 0 | 0 | 0 | 0.083 | 0 | 0 | 0.86 | 0.866 |
| VCAM1 | SELE | 9606.ENSP00000294728 | 9606.ENSP00000331736 | 0 | 0 | 0 | 0 | 0.122 | 0 | 0 | 0.916 | 0.924 |
| VCAM1 | TNF | 9606.ENSP00000294728 | 9606.ENSP00000398698 | 0 | 0 | 0 | 0 | 0 | 0 | 0.9 | 0.884 | 0.987 |
| VEGFA | MAPK1 | 9606.ENSP00000478570 | 9606.ENSP00000215832 | 0 | 0 | 0 | 0 | 0 | 0 | 0 | 0.469 | 0.469 |
| VEGFA | HMOX1 | 9606.ENSP00000478570 | 9606.ENSP00000216117 | 0 | 0 | 0 | 0 | 0 | 0 | 0 | 0.683 | 0.683 |
| VEGFA | MMP2 | 9606.ENSP00000478570 | 9606.ENSP00000219070 | 0 | 0 | 0 | 0 | 0.065 | 0 | 0.9 | 0.848 | 0.984 |
| VEGFA | PLAT | 9606.ENSP00000478570 | 9606.ENSP00000220809 | 0 | 0 | 0 | 0 | 0.085 | 0 | 0 | 0.508 | 0.53 |
| VEGFA | MPO | 9606.ENSP00000478570 | 9606.ENSP00000225275 | 0 | 0 | 0 | 0 | 0.062 | 0 | 0 | 0.562 | 0.571 |
| VEGFA | CCL2 | 9606.ENSP00000478570 | 9606.ENSP00000225831 | 0 | 0 | 0 | 0 | 0 | 0 | 0 | 0.856 | 0.856 |
| VEGFA | IL2 | 9606.ENSP00000478570 | 9606.ENSP00000226730 | 0 | 0 | 0 | 0 | 0 | 0 | 0 | 0.753 | 0.753 |
| VEGFA | IFNG | 9606.ENSP00000478570 | 9606.ENSP00000229135 | 0 | 0 | 0 | 0 | 0 | 0 | 0 | 0.689 | 0.689 |
| VEGFA | MAPK14 | 9606.ENSP00000478570 | 9606.ENSP00000229795 | 0 | 0 | 0 | 0 | 0 | 0 | 0.9 | 0.539 | 0.951 |
| VEGFA | NR3C1 | 9606.ENSP00000478570 | 9606.ENSP00000231509 | 0 | 0 | 0 | 0 | 0 | 0 | 0 | 0.42 | 0.42 |
| VEGFA | CTSD | 9606.ENSP00000478570 | 9606.ENSP00000236671 | 0 | 0 | 0 | 0 | 0 | 0 | 0 | 0.405 | 0.405 |
| VEGFA | CAT | 9606.ENSP00000478570 | 9606.ENSP00000241052 | 0 | 0 | 0 | 0 | 0 | 0 | 0 | 0.601 | 0.601 |
| VEGFA | AHR | 9606.ENSP00000478570 | 9606.ENSP00000242057 | 0 | 0 | 0 | 0 | 0 | 0 | 0 | 0.457 | 0.457 |
| VEGFA | CDK4 | 9606.ENSP00000478570 | 9606.ENSP00000257904 | 0 | 0 | 0 | 0 | 0 | 0.155 | 0 | 0.565 | 0.616 |
| VEGFA | MAPK3 | 9606.ENSP00000478570 | 9606.ENSP00000263025 | 0 | 0 | 0 | 0 | 0 | 0 | 0 | 0.754 | 0.754 |
| VEGFA | IL1B | 9606.ENSP00000478570 | 9606.ENSP00000263341 | 0 | 0 | 0 | 0 | 0 | 0 | 0 | 0.857 | 0.857 |
| VEGFA | KDR | 9606.ENSP00000478570 | 9606.ENSP00000263923 | 0 | 0 | 0 | 0 | 0.062 | 0.984 | 0.9 | 0.992 | 0.999 |
| VEGFA | EGF | 9606.ENSP00000478570 | 9606.ENSP00000265171 | 0 | 0 | 0 | 0 | 0 | 0 | 0 | 0.878 | 0.878 |
| VEGFA | CDK2 | 9606.ENSP00000478570 | 9606.ENSP00000266970 | 0 | 0 | 0 | 0 | 0 | 0.105 | 0 | 0.522 | 0.554 |
| VEGFA | TP53 | 9606.ENSP00000478570 | 9606.ENSP00000269305 | 0 | 0 | 0 | 0 | 0 | 0.161 | 0 | 0.81 | 0.834 |
| VEGFA | SOD1 | 9606.ENSP00000478570 | 9606.ENSP00000270142 | 0 | 0 | 0 | 0 | 0.063 | 0 | 0 | 0.503 | 0.514 |
| VEGFA | CCNA2 | 9606.ENSP00000478570 | 9606.ENSP00000274026 | 0 | 0 | 0 | 0 | 0 | 0 | 0 | 0.47 | 0.47 |
| VEGFA | EGFR | 9606.ENSP00000478570 | 9606.ENSP00000275493 | 0 | 0 | 0 | 0 | 0.089 | 0 | 0.6 | 0.861 | 0.944 |
| VEGFA | GJA1 | 9606.ENSP00000478570 | 9606.ENSP00000282561 | 0 | 0 | 0 | 0 | 0 | 0 | 0 | 0.56 | 0.56 |
| VEGFA | PPARG | 9606.ENSP00000478570 | 9606.ENSP00000287820 | 0 | 0 | 0 | 0 | 0 | 0 | 0 | 0.662 | 0.662 |
| VEGFA | BAX | 9606.ENSP00000478570 | 9606.ENSP00000293288 | 0 | 0 | 0 | 0 | 0 | 0 | 0 | 0.467 | 0.467 |
| VEGFA | VCAM1 | 9606.ENSP00000478570 | 9606.ENSP00000294728 | 0 | 0 | 0 | 0 | 0.062 | 0 | 0 | 0.756 | 0.761 |
| VEGFA | NOS3 | 9606.ENSP00000478570 | 9606.ENSP00000297494 | 0 | 0 | 0 | 0 | 0.064 | 0 | 0.9 | 0.857 | 0.985 |
| VEGFA | MMP3 | 9606.ENSP00000478570 | 9606.ENSP00000299855 | 0 | 0 | 0 | 0 | 0 | 0 | 0.9 | 0.671 | 0.965 |
| VEGFA | INSR | 9606.ENSP00000478570 | 9606.ENSP00000303830 | 0 | 0 | 0 | 0 | 0.062 | 0 | 0.6 | 0.245 | 0.691 |
| VEGFA | F2 | 9606.ENSP00000478570 | 9606.ENSP00000308541 | 0 | 0 | 0 | 0 | 0 | 0 | 0 | 0.583 | 0.583 |
| VEGFA | PRKACA | 9606.ENSP00000478570 | 9606.ENSP00000309591 | 0 | 0 | 0 | 0 | 0 | 0 | 0.9 | 0.063 | 0.902 |
| VEGFA | MMP1 | 9606.ENSP00000478570 | 9606.ENSP00000322788 | 0 | 0 | 0 | 0 | 0.069 | 0 | 0.9 | 0.683 | 0.967 |
| VEGFA | HSPA5 | 9606.ENSP00000478570 | 9606.ENSP00000324173 | 0 | 0 | 0 | 0 | 0 | 0 | 0 | 0.467 | 0.467 |
| VEGFA | GSK3B | 9606.ENSP00000478570 | 9606.ENSP00000324806 | 0 | 0 | 0 | 0 | 0 | 0 | 0 | 0.504 | 0.504 |
| VEGFA | PGR | 9606.ENSP00000478570 | 9606.ENSP00000325120 | 0 | 0 | 0 | 0 | 0 | 0 | 0 | 0.606 | 0.606 |
| VEGFA | NOS2 | 9606.ENSP00000478570 | 9606.ENSP00000327251 | 0 | 0 | 0 | 0 | 0.065 | 0 | 0 | 0.518 | 0.53 |
| VEGFA | SELE | 9606.ENSP00000478570 | 9606.ENSP00000331736 | 0 | 0 | 0 | 0 | 0 | 0 | 0 | 0.685 | 0.685 |
| VEGFA | F3 | 9606.ENSP00000478570 | 9606.ENSP00000334145 | 0 | 0 | 0 | 0 | 0.062 | 0 | 0 | 0.681 | 0.687 |
| VEGFA | CYP3A4 | 9606.ENSP00000478570 | 9606.ENSP00000337915 | 0 | 0 | 0 | 0 | 0 | 0 | 0 | 0.404 | 0.404 |
| VEGFA | ESR2 | 9606.ENSP00000478570 | 9606.ENSP00000343925 | 0 | 0 | 0 | 0 | 0 | 0 | 0 | 0.556 | 0.556 |
| VEGFA | PTGER3 | 9606.ENSP00000478570 | 9606.ENSP00000349003 | 0 | 0 | 0 | 0 | 0 | 0 | 0 | 0.4 | 0.4 |
| VEGFA | PIK3CG | 9606.ENSP00000478570 | 9606.ENSP00000352121 | 0 | 0 | 0 | 0 | 0 | 0 | 0 | 0.451 | 0.451 |
| VEGFA | DPP4 | 9606.ENSP00000478570 | 9606.ENSP00000353731 | 0 | 0 | 0 | 0 | 0 | 0 | 0 | 0.451 | 0.451 |
| VEGFA | TOP1 | 9606.ENSP00000478570 | 9606.ENSP00000354522 | 0 | 0 | 0 | 0 | 0 | 0 | 0 | 0.468 | 0.468 |
| VEGFA | PTGS1 | 9606.ENSP00000478570 | 9606.ENSP00000354612 | 0 | 0 | 0 | 0 | 0.064 | 0 | 0 | 0.419 | 0.432 |
| VEGFA | PTGS2 | 9606.ENSP00000478570 | 9606.ENSP00000356438 | 0 | 0 | 0 | 0 | 0.083 | 0 | 0 | 0.892 | 0.896 |
| VEGFA | JUN | 9606.ENSP00000478570 | 9606.ENSP00000360266 | 0 | 0 | 0 | 0 | 0.066 | 0.182 | 0.9 | 0.683 | 0.972 |
| VEGFA | PTPN1 | 9606.ENSP00000478570 | 9606.ENSP00000360683 | 0 | 0 | 0 | 0 | 0 | 0 | 0 | 0.539 | 0.539 |
| VEGFA | PLAU | 9606.ENSP00000478570 | 9606.ENSP00000361850 | 0 | 0 | 0 | 0 | 0.069 | 0 | 0 | 0.652 | 0.662 |
| VEGFA | ALOX5 | 9606.ENSP00000478570 | 9606.ENSP00000363512 | 0 | 0 | 0 | 0 | 0 | 0 | 0 | 0.454 | 0.454 |
| VEGFA | AR | 9606.ENSP00000478570 | 9606.ENSP00000363822 | 0 | 0 | 0 | 0 | 0 | 0 | 0 | 0.6 | 0.6 |
| VEGFA | THBD | 9606.ENSP00000478570 | 9606.ENSP00000366307 | 0 | 0 | 0 | 0 | 0.065 | 0 | 0 | 0.527 | 0.538 |
| VEGFA | CDK1 | 9606.ENSP00000478570 | 9606.ENSP00000378699 | 0 | 0 | 0 | 0 | 0 | 0.105 | 0 | 0.413 | 0.452 |
| VEGFA | MAPK8 | 9606.ENSP00000478570 | 9606.ENSP00000378974 | 0 | 0 | 0 | 0 | 0 | 0 | 0 | 0.517 | 0.517 |
| VEGFA | IL6 | 9606.ENSP00000478570 | 9606.ENSP00000385675 | 0 | 0 | 0 | 0 | 0.063 | 0 | 0.9 | 0.879 | 0.987 |
| VEGFA | CHEK1 | 9606.ENSP00000478570 | 9606.ENSP00000388648 | 0 | 0 | 0 | 0 | 0 | 0.129 | 0 | 0.366 | 0.424 |
| VEGFA | TNF | 9606.ENSP00000478570 | 9606.ENSP00000398698 | 0 | 0 | 0 | 0 | 0 | 0 | 0 | 0.88 | 0.88 |
| VEGFA | ESR1 | 9606.ENSP00000478570 | 9606.ENSP00000405330 | 0 | 0 | 0 | 0 | 0.062 | 0 | 0 | 0.685 | 0.691 |
| VEGFA | LDLR | 9606.ENSP00000478570 | 9606.ENSP00000454071 | 0 | 0 | 0 | 0 | 0.06 | 0 | 0 | 0.393 | 0.405 |
| XDH | GSR | 9606.ENSP00000368727 | 9606.ENSP00000221130 | 0 | 0 | 0 | 0 | 0 | 0 | 0 | 0.516 | 0.516 |
| XDH | MPO | 9606.ENSP00000368727 | 9606.ENSP00000225275 | 0 | 0 | 0 | 0 | 0 | 0 | 0 | 0.448 | 0.448 |
| XDH | CAT | 9606.ENSP00000368727 | 9606.ENSP00000241052 | 0 | 0 | 0 | 0 | 0.062 | 0 | 0 | 0.697 | 0.703 |
| XDH | IL1B | 9606.ENSP00000368727 | 9606.ENSP00000263341 | 0 | 0 | 0 | 0 | 0.063 | 0 | 0 | 0.403 | 0.417 |
| XDH | SOD1 | 9606.ENSP00000368727 | 9606.ENSP00000270142 | 0 | 0 | 0 | 0 | 0 | 0 | 0 | 0.535 | 0.535 |
| XDH | PPARG | 9606.ENSP00000368727 | 9606.ENSP00000287820 | 0 | 0 | 0 | 0 | 0.063 | 0.133 | 0 | 0.364 | 0.438 |
| XDH | NOS3 | 9606.ENSP00000368727 | 9606.ENSP00000297494 | 0 | 0 | 0 | 0 | 0 | 0 | 0 | 0.578 | 0.578 |
| XDH | CYP1A2 | 9606.ENSP00000368727 | 9606.ENSP00000342007 | 0 | 0 | 0 | 0 | 0.051 | 0 | 0.9 | 0.134 | 0.91 |
| XDH | IL6 | 9606.ENSP00000368727 | 9606.ENSP00000385675 | 0 | 0 | 0 | 0 | 0 | 0 | 0 | 0.404 | 0.404 |
| XDH | TNF | 9606.ENSP00000368727 | 9606.ENSP00000398698 | 0 | 0 | 0 | 0 | 0.062 | 0 | 0 | 0.494 | 0.505 |
